# Stereoselective benzylic C(sp^3^)–H alkenylation enabled by metallaphotoredox catalysis†

**DOI:** 10.1039/d4sc02830a

**Published:** 2024-07-08

**Authors:** Yantao Li, Haonan Bai, Qi Gao, Kai Liu, Jie Han, Weipeng Li, Chengjian Zhu, Jin Xie

**Affiliations:** a State Key Laboratory of Coordination Chemistry, Jiangsu Key Laboratory of Advanced Organic Materials, Chemistry and Biomedicine Innovation Center (ChemBIC), School of Chemistry and Chemical Engineering, Nanjing University Nanjing 210023 China; b Green Catalysis Center, and College of Chemistry, Zhengzhou University Zhengzhou 450001 China; c State Key Laboratory of Natural Medicines, China Pharmaceutical University Nanjing 211198 China; d State Key Laboratory of Organometallic Chemistry, Shanghai Institute of Organic Chemistry Shanghai 200032 China

## Abstract

Selective activation of the benzylic C(sp^3^)–H bond is pivotal for the construction of complex organic frameworks. Achieving precise selectivity among C–H bonds with comparable energetic and steric profiles remains a profound synthetic challenge. Herein, we unveil a site- and stereoselective benzylic C(sp^3^)–H alkenylation utilizing metallaphotoredox catalysis. Various linear and cyclic (*Z*)-all-carbon tri- and tetrasubstituted olefins can be smoothly obtained. This strategy can be applied to complex substrates with multiple benzylic sites, previously deemed unsuitable due to the uncontrollable site-selectivity. In addition, sensitive functional groups such as terminal alkenyl and TMS groups are compatible under the mild conditions. The exceptional site-selectivity and broad substrate compatibility are attributed to the visible-light catalyzed relay electron transfer–proton transfer process. More importantly, we have extended this methodology to achieve enantioselective benzylic C(sp^3^)–H alkenylation, producing highly enantioenriched products. The applicability and scalability of our protocol are further validated through late-stage functionalization of complex structures and gram-scale operations, underscoring its practicality and robustness.

## Abstract 2

We report a site- and stereoselective benzylic C(sp^3^)–H alkenylation utilizing metallaphotoredox catalysis. In addition, enantioselective benzylic C(sp^3^)–H alkenylation can successfully produce highly enantioenriched tetrasubstituted olefins.

## Graphical abstract

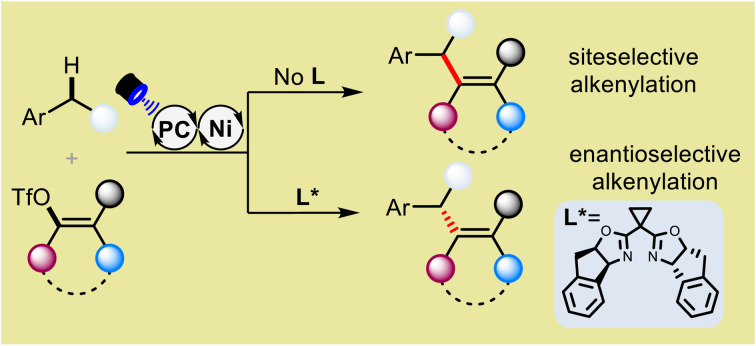

## Introduction

**Scheme 1:**
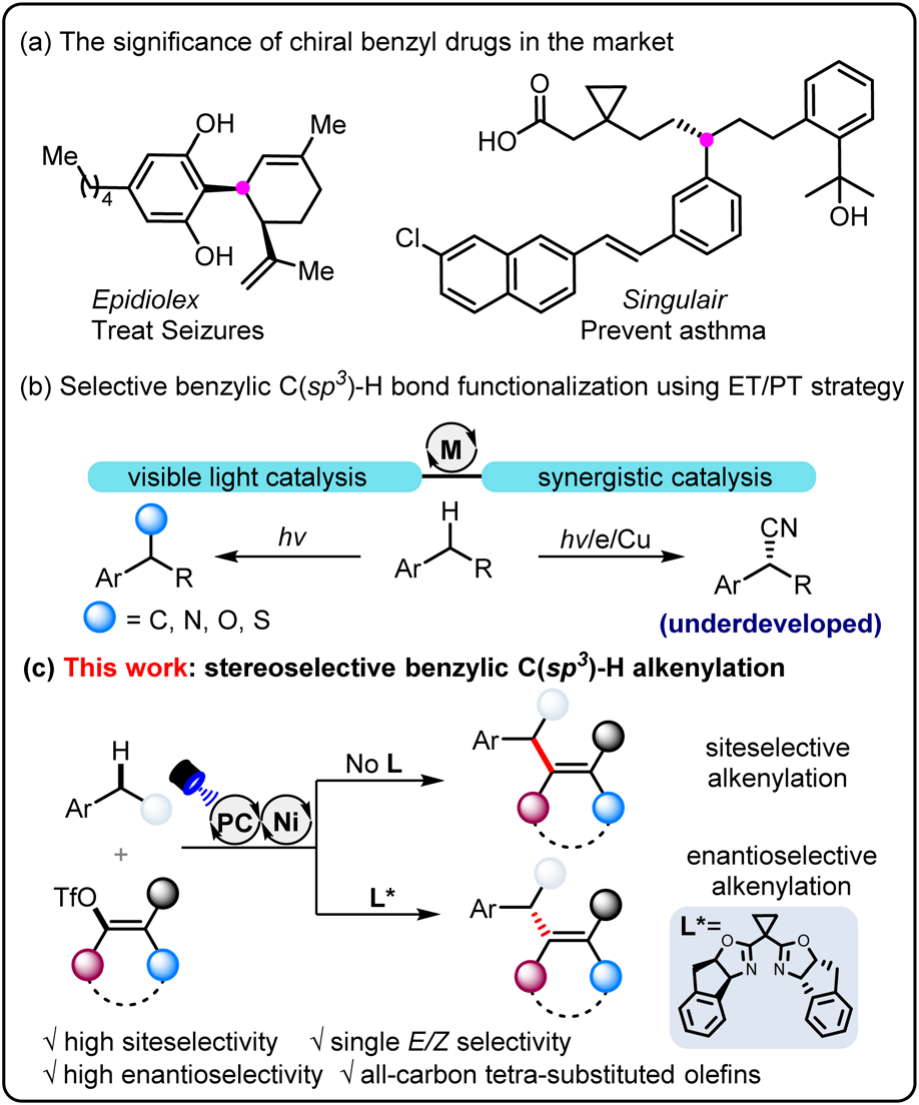
The state of the art for light-induced benzylic C(sp^3^)–H functionalization. ET/PT = electron transfer/proton transfer.

Arene derivatives bearing the benzylic stereogenic center are one of the most prevalent functional motifs among pharmaceuticals,^1^ and consequently, considerable efforts have been dedicated to developing highly direct and efficient synthetic methods (Scheme 1a). In this context, transformations of native benzylic C(sp^3^)–H bonds to muti-functionalized target molecules represent one of the most attractive strategies in organic synthesis.^2^ Over the past decade, metallaphotocatalysis^3^ has blossomed as a powerful strategy to construct different kinds of chemical bonds, offering a complementary toolbox to traditional transition metal catalysis.^4^ In general, the photocatalytic hydrogen atom transfer (HAT) process is the dominant method to produce benzylic radicals owing to the weak BDE of benzylic C–H bonds,^5^ which can be further captured by a series of transition metals, such as Cu,^6^ Ni,^7^ Co^8^ and Pd^9^ to facilitate benzylic C(sp^3^)–H functionalization. However, the reliance on diverse HAT reagents (*e.g.*, NSFI, DTBP, TBADT, and thiol) presents a challenge in achieving regioselectivity, particularly for substrates with intricate structures. Very recently, the electron transfer–proton transfer (ET/PT) strategy has gained increasing attention and offers a promising way to address the C–H selectivity issue (Scheme 1b).^10^ With this strategy, copper-catalyzed selective C–C bond formation has been realized directly from benzylic C–H bonds.^11^ Despite these efforts, photo/nickel-catalyzed carbofunctionalization with the ET/PT process of toluene derivatives has not been disclosed. The main challenges might stem from the mismatched reaction rate, owing to the rapid oxidation addition process of organohalides to the nickel catalyst while relatively slow rate to give rise to benzylic radicals.

On the other hand, all-carbon tetrasubstituted olefins have attracted enormous attention, since the structural motifs widely exist in a broad spectrum of significant biologically active compounds^12^ and versatile organic intermediates.^13^ The traditional double bond-forming methods,^14^ including Wittig olefination, Peterson olefination, and olefin metathesis are effective, but the *Z*/*E* stereoselectivity remains a great challenge. Difunctionalization of alkyne and cross-coupling reactions usually cannot avoid the involvement of the organometallic reagents.^15^ Despite recent efforts, the development of efficient methods to construct all-carbon tetrasubstituted olefins in a stereoselective manner is synthetically challenging. With our continual work in photo/metal synergistic catalysis,^16^ we questioned if we could realize stereoselective alkenylation of benzylic C(sp^3^)–H bonds in a series of readily available alkylarenes (Scheme 1c). The electron transfer–proton transfer pathway can avoid other side reactions with interfering C(sp^3^)–H bonds. By employing a chiral bisoxazoline ligand, enantioselective benzylic C(sp^3^)–H alkenylation has also been achieved. Notably, in this catalytic system, we are able to overcome the significant challenge of steric hindrance posed by the synthesis of specific (*Z*)-all-carbon tetrasubstituted alkenes.

**Scheme 2:**
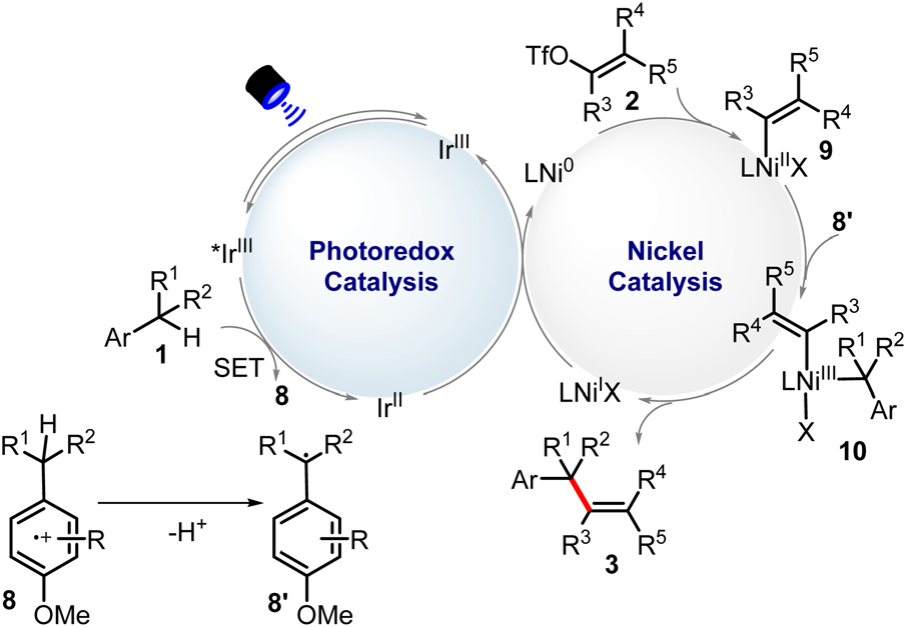
Mechanistic studies of benzylic C(sp^3^)–H alkenylation reaction.

The radical inhibition experiments with TEMPO indicated the reaction probably proceeded *via* a radical process. In some examples, homocoupling of substrate 1 (toluene derivatives) could also be detected. The luminescence quenching experiment shows that [Ir(dF(CF_3_)ppy)_2_(4,4′-dCF_3_bpy)]PF_6_* can be quenched by 1a. Based on our mechanistic experiments (see details in the ESI†) and previous work,^17^ a possible mechanism was proposed in Scheme 2. The electronic-rich arene (1) is first oxidized by the excited-state photocatalyst [Ir^III^]*, experiencing the key electron transfer–deprotonation process, to finally produce radical 8′ and simultaneously the [Ir^II^] complex. Then, oxidation addition of substrate 2 to Ni^0^ leads to intermediate 9, which can be captured by 8′ through radical addition to produce 10 species. Next, this Ni^III^ complex would undergo reductive elimination to deliver the final product 3 and Ni^I^-complex. Finally, [Ir^II^] complex can donate one electron to Ni^I^ to generate Ir^III^ and Ni^0^, completing two catalytic cycles, respectively.

## Results and discussion

We initiated our investigation by achieving benzylic C(sp^3^)–H alkenylation of 1-ethyl-4-methoxybenzene (1a) with ethyl (*Z*)-2-benzyl-3-(((trifluoromethyl)sulfonyl)oxy)but-2-enoate (2a) (Table 1). Several allylic or benzylic C(sp^3^)–H bonds in the model substrates (1a and 2a) make it generally challenging to differentiate through the HAT manner. The optimized reaction conditions include 2 mol% [Ir(dF(CF_3_)ppy)_2_(4,4′-d(CF_3_)bpy)]PF_6_ (^1/2^*E*_red_(*Ir^III^/Ir^II^) = +1.65 V *vs.* SCE) as a photocatalyst, which is sufficiently positive to oxidize 1a (^1/2^*E* = +1.52 V *vs.* SCE), 10% mol NiCl_2_·6H_2_O as the transition metal catalyst, Li_2_CO_3_ as the base and DMF as the solvent under blue LED radiation at rt for 24 h. The desired product (3a) was obtained in 85% isolated yield (entry 1). When PC-1 was replaced with PC-2 (^1/2^*E*_red_(*Ir^III^/Ir^II^) = +1.21 V *vs.* SCE), 62% of 3a was obtained (entry 2). Although PC-2 holds lower oxidation potential, the potential overlap between the excited PC-2 and 1a could promote the single electron oxidation process for fast deprotonation and benzylic radical generation as the driving force. Other nickel sources like Ni(acac)_2_ or NiBr_2_ could lead to a diminished yield (entries 3 and 4). The screening of other solvents revealed that DMF led to the best reaction efficiency (entries 5–7). The use of Cs_2_CO_3_ leads to a slightly decreased yield of 79% (entry 8). The control experiments suggested that PC-1, [Ni], base, and light were necessary factors to deliver product 3a (entry 9).

**Table 1:** Optimization of the reaction conditions^a^

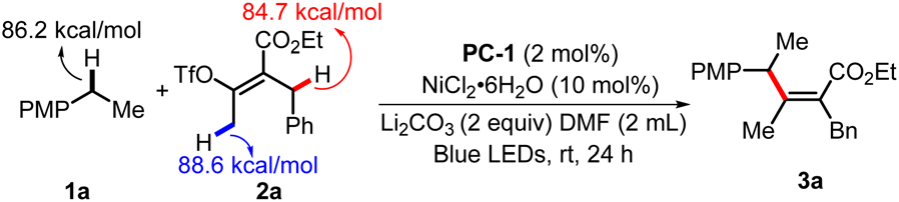		
Entry	Variation of standard conditions	Yield^b^ [%]
**1**	**None**	**85** c
2	PC-2 instead of PC-1	62
3	Ni(acac)_2_ instead of NiCl_2_·6H_2_O	22
4	NiBr_2_ instead of NiCl_2_·6H_2_O	0
5	MeCN as solvent	6
6	DCM as the solvent	ND
7	THF as the solvent	20
8	Cs_2_CO_3_ as base	79
9	Without PC-1 or [Ni] or base or light	ND
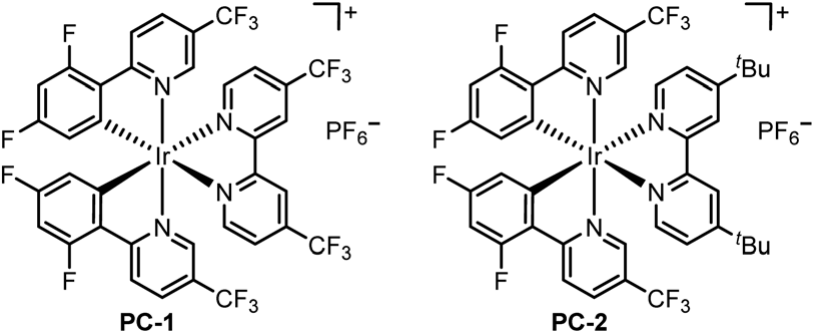		

a Standard conditions: PC-1 (2 mol%), NiCl_2_·6H_2_O (10 mol%), 1a (0.4 mmol), 2a (0.2 mmol), Li_2_CO_3_ (0.4 mmol), DMF (2 mL), blue LEDs, 24 h.

b Isolated yields. DMF = *N*,*N*-dimethylformamide; ND = not detected. PMP = *p*-methoxyphenyl. BDE results were calculated using DFT calculations.

c 79% yield on a 3 mmol scale.

**Scheme 3:**
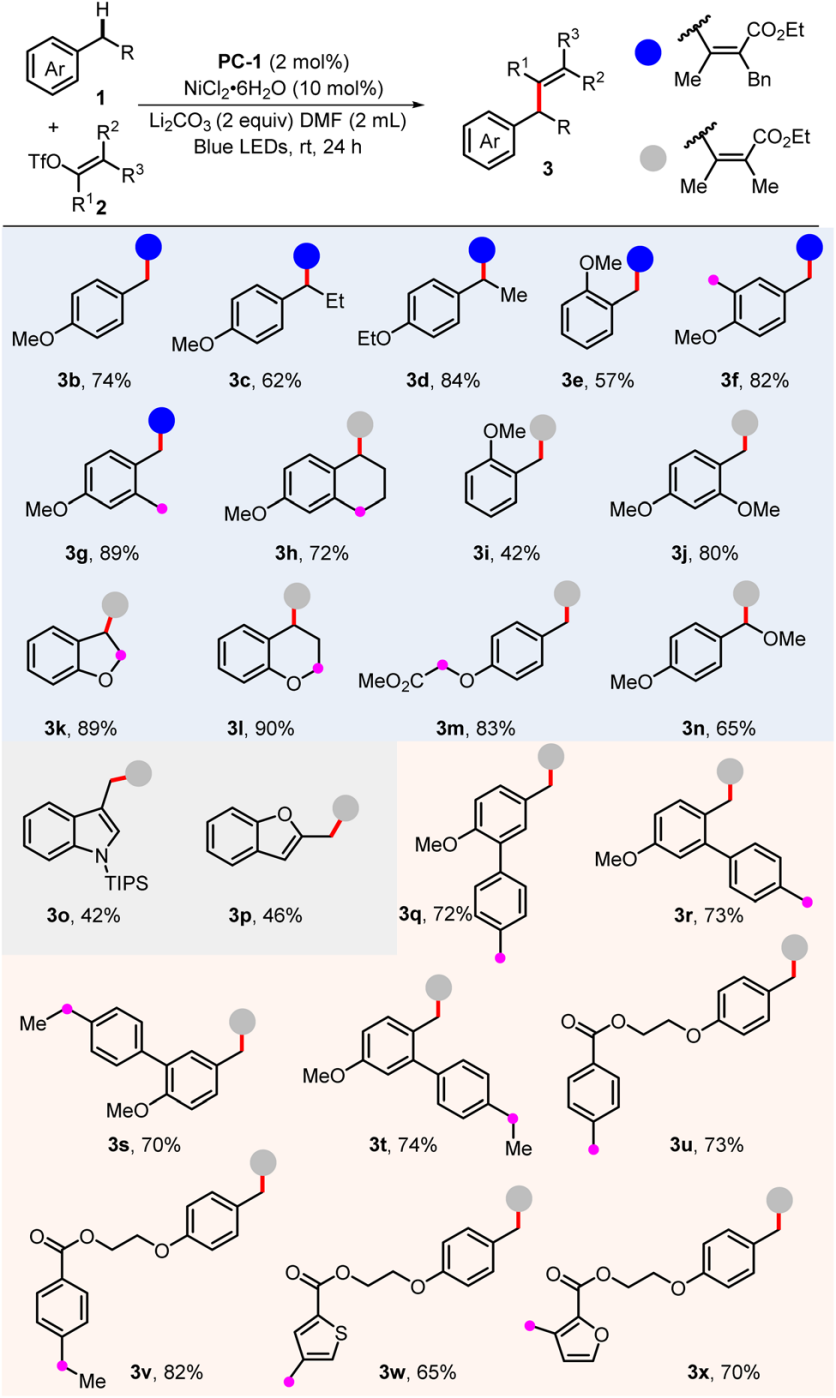
Substrate scope of toluene derivatives. Standard conditions: PC-1 (2 mol%), NiCl_2_·6H_2_O (10 mol%), 1 (0.4 mmol), 2 (0.2 mmol), Li_2_CO_3_ (0.4 mmol), DMF (2 mL), blue LEDs, 24 h. The *Z*/*E* ratios of 2a were determined by ^1^H NMR as >20/1.

With the optimized benzylic C(sp^3^)–H alkenylation conditions in hands, the scope of the alkylarenes was first determined, and the results are shown in Scheme 3. This method enabled selective C–H alkenylation of both primary and secondary benzylic C(sp^3^)–H bonds, providing the desired products in good yields (3b–3d). Interestingly, when several methyl and methylene groups were in the same aromatic ring, they could be differentiated well with excellent selectivity (3f, 3g, and 3h). In this case, it is found that the *para*-position is relatively more reactive, exhibiting superiority during the electron transfer–proton transfer strategy. When the methoxy group was adjacent, the yield of benzylic C–H alkenylation slightly decreased (3e and 3i), while adding another methoxy group in the 4-position promoted the transformation (3j). Importantly, the presence of weaker C–H bonds in the substrate did not significantly impact reaction efficiency (3m and 3n). In addition, the electric-rich heteroaromatics could also successfully deliver the desired product in acceptable yields (3o and 3p). A noteworthy observation was the regioselectivity displayed in substrates containing two comparable benzylic C–H bonds. As evidenced by products 3q–3x, the reaction preferentially targeted the C–H bond directly connected to the more electron-rich aromatic rings. We envisioned that an electron-rich aromatic ring would undergo single electron oxidation more rapidly and then proceed deprotonation to produce a benzylic radical intermediate.^10*a*,18^

**Scheme 4:**
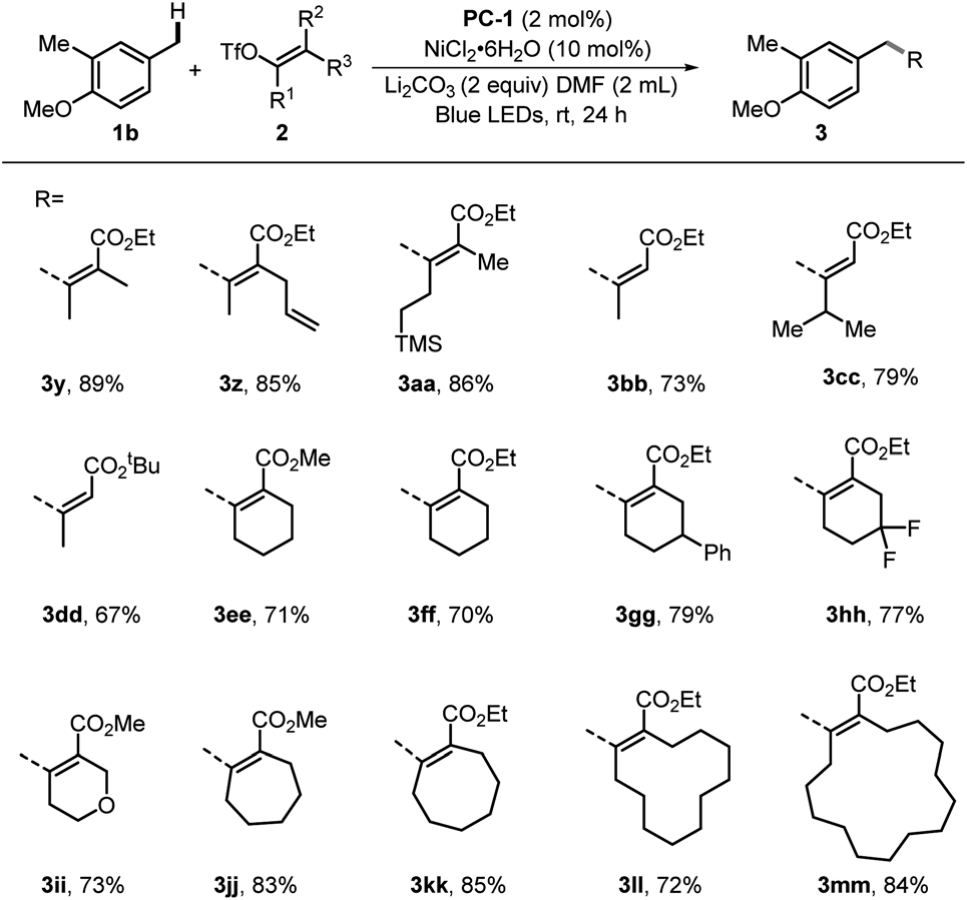
Scope of alkenyl triflates. Standard conditions: PC-1 (2 mol%), NiCl_2_·6H_2_O (10 mol%), 1b (0.4 mmol), 2 (0.2 mmol), Li_2_CO_3_ (0.4 mmol), DMF (2 mL), blue LEDs, 24 h. The *Z*/*E* ratios of 2 were determined by ^1^H NMR as >20/1.

We commenced the assessment of electron-poor alkenyl triflates bearing diverse substitutions (Scheme 4). The examples in this article included various active C–H bonds. They remained unchanged after experiencing the unique ET/PT process. It was envisioned that the ester group on the side chain could play a role in the stabilization of the transition state in coordination with the nickel center.^16*c*^ In all examples, excellent stereoselectivity was observed with the ratio exceeding 20 : 1 (regioselectivity and *Z*/*E* selectivity). This protocol performed well with the hindered tetrasubstituted acyclic triflates. Substrates containing methyl, alkenyl, and TMS groups produced the desired products (3y–3aa) in 89%, 85% and 86% yield, respectively. The reaction was also suitable for the synthesis of trisubstituted alkenes with good yields and definite configuration (3bb–3dd). The cyclic six-membered alkenyl triflates, including those with substituents at 4-positions, afforded products (3ee–3ii) in yields of 70–79%. Additionally, the cyclic vinyl triflates, derived from 7-, 8-, 12-, or 15-membered rings, could couple with 1b efficiently to furnish the target products (3jj–3mm) in moderate to good yields.

**Scheme 5:**
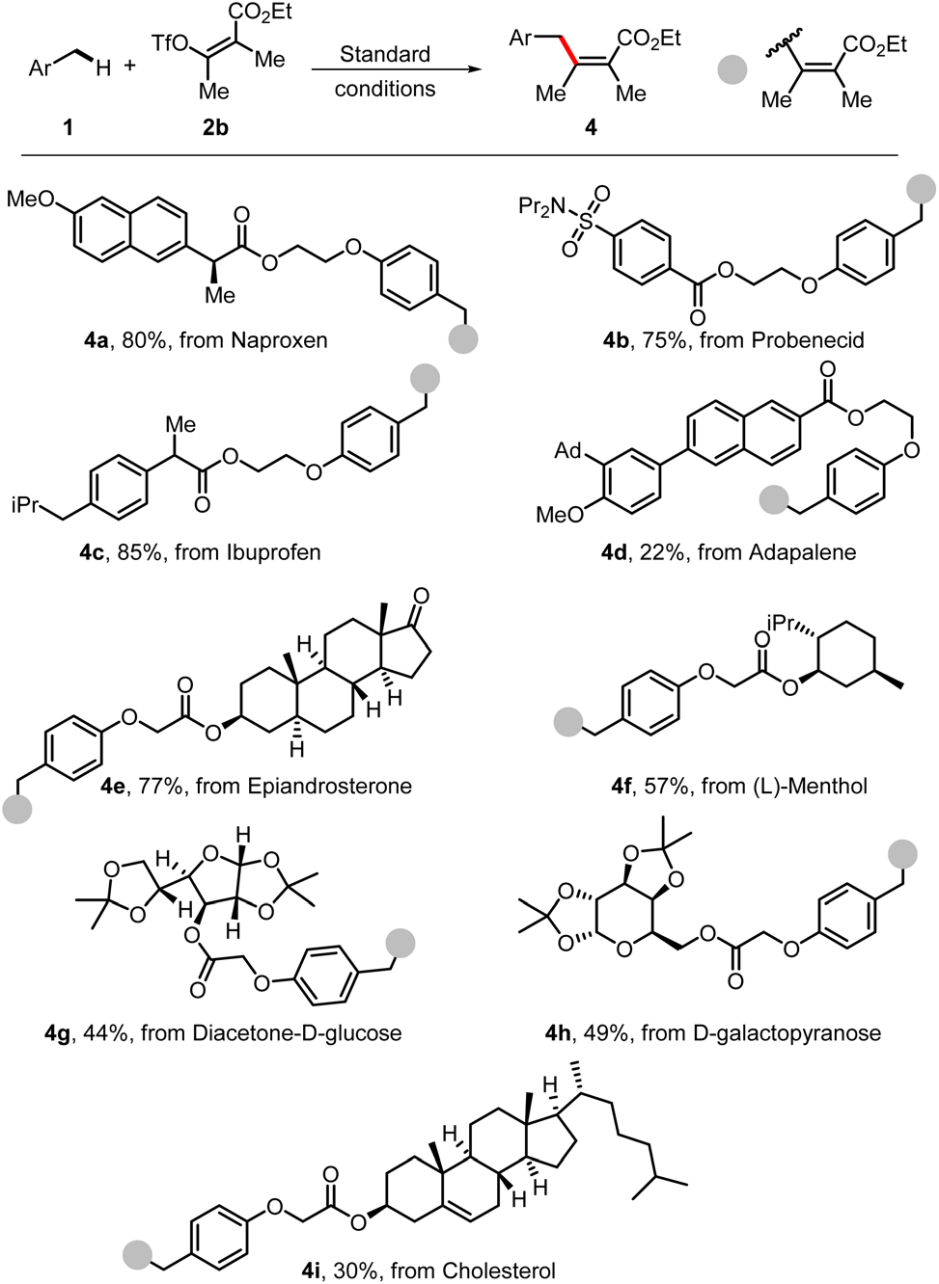
Late-stage functionalization of complex molecules. Standard conditions: PC-1 (2 mol%), NiCl_2_·6H_2_O (10 mol%), 1 (0.4 mmol), 2a (0.2 mmol), Li_2_CO_3_ (0.4 mmol), DMF (2 mL), blue LEDs, 24 h. The *Z*/*E* ratios of 2 were determined by ^1^H NMR as >20/1.

To further demonstrate the synthetic value of this methodology, the strategy was applied for late-stage modification of a number of complex molecules. Several molecules from biologically important natural products, pharmaceuticals, or complex carbohydrates were successfully applied in this benzylic C–H alkenylation and are shown in Scheme 5. The derivatives from naproxen (4a), probenecid (4b), ibuprofen (4c), and adapalene (4d) underwent this transformation smoothly. Moreover, complex natural products bearing several reactive C–H sites could also be modified, affording the desired products (4e, 4f, and 4i) in moderate yields. Additionally, the protected carbohydrates underwent this C–H alkenylation reaction well in satisfactory yields of 44–49% under standard conditions (4g and 4h).

**Scheme 6:**
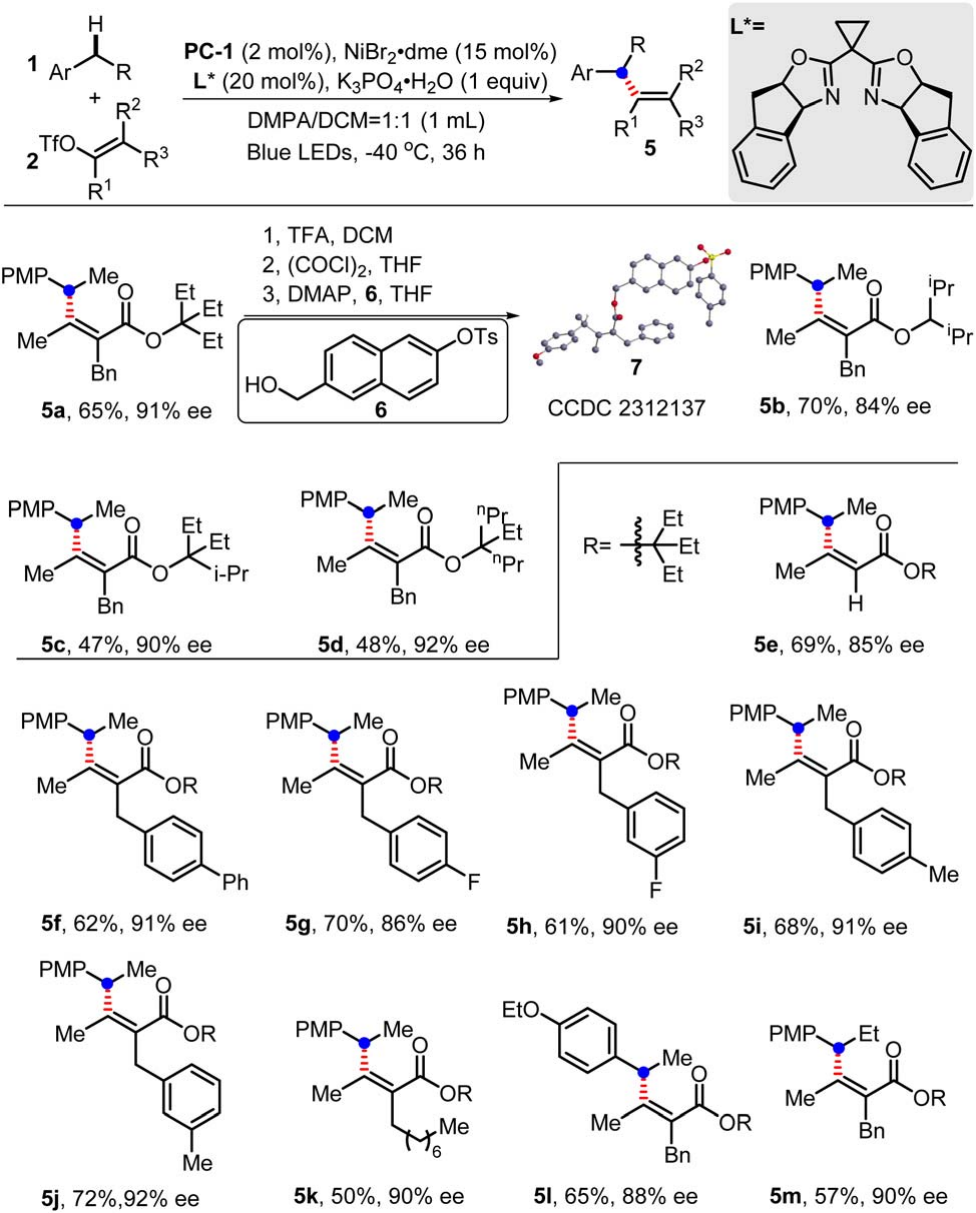
Enantioselective benzylic C(sp^3^)–H alkenylation by dual photoredox/nickel catalysis. Standard conditions: PC-1 (2 mol%), NiBr_2_·dme (15 mol%), L* (20 mol%), 1 (0.2 mmol), 2 (0.1 mmol), K_3_PO_4_·H_2_O (0.1 mmol), DMPA/DCM (v/v = 1 : 1, 1 mL), blue LEDs, −40 °C, 36 h. DMPA = *N*,*N*-dimethylpropionamide; PMP, *p*-methoxyphenyl; enantioselectivities were determined by chiral HPLC analysis; the definition of the absolute configuration was assigned by X-ray analysis of its derivative 7 (CCDC 2312137†).^19^ The *Z/E* ratios of 2 were determined by ^1^H NMR as >20/1.

The enantioselective benzylic C(sp^3^)–H alkenylation was then explored. With the chiral bisoxazoline ligand and a low temperature of −40 °C, the expected products could be obtained in moderate yields with high enantiomeric excess (Scheme 6). The function of the ester part installed in the alkenyl triflates was first explored, which showed that either multiple isopropyl, propyl or ethyl groups could result in a relatively high ee value (5a–5d). After careful investigation, 3-ethylpentan-3-yl ester was finally chosen to investigate alkenyl triflate substrates. Trisubstituted alkene can be obtained with a slightly lower ee value (85% ee, 5e). Changing substituents on the aromatic ring showed that substrates with either electron-deficient or electron-rich groups can uniformly undergo enantioselective C–H alkenylation with high ee values (5f–5j). Notably, the installation of the octyl group hardly influenced the ee value, although the yield decreased to 50% (5k). Substrates like 1-ethoxy-4-ethylbenzene or 1-methoxy-4-propylbenzene were also suitable to deliver products (5l–5m) with moderate yields and good ee value.

## Conclusions

In conclusion, we have developed synergistic photoredox/nickel catalysis for selective benzylic C(sp^3^)–H alkenylation under mild reaction conditions. This method can precisely target benzylic C–H bonds of substrates. The versatility of our approach is demonstrated through the synthesis of an extensive suite of both linear and cyclic (*Z*)-all-carbon tri- and tetrasubstituted olefins, showcasing an exceptional breadth of functional group compatibility. A crucial factor contributing to the excellent site-selectivity and compatibility is the implementation of a visible-light catalyzed relay electron transfer–proton transfer process, a novel mechanism in the context of photoredox/nickel catalysis applied to the functionalization of toluene derivatives. More importantly, an enantioselective benzylic C(sp^3^)–H alkenylation has also been achieved with the chiral bisoxazoline ligand, providing the products in good yield and enantioselectivity.

## Data availability

The data that support the findings of this study are available in the ESI† or from the corresponding author upon reasonable request.

## Author contributions

J. X. C. Z. and Y. L. conceived and designed the project. Y. L., H. B., Q. G., K. L., J. H. performed and analyzed the experimental data. J. X. wrote the manuscript with input from all authors.

## Conflicts of interest

There are no conflicts to declare.

## Supplementary Material

SC-015-D4SC02830A-s001

SC-015-D4SC02830A-s002

## References

[cit1] Top 200 Small Molecule Drugs by Retail Sales in 2022, Ryan E. Williams, Njarðarson Group (The University of Arizona)

[cit2] Sun C.-L., Li B.-J., Shi Z.-J. (2010). Chem. Rev..

[cit3] Prier C. K., Rankic D. A., MacMillan D. W. C. (2013). Chem. Rev..

[cit4] (a) Meijere A. and Diederich F., Metal-Catalyzed Cross-Couplig Reactions, WILEY-VCH, 2004

[cit5] Cao H., Tang X., Tang H., Yuan Y., Wu J. (2021). Chem Catal..

[cit6] Bao X., Wang Q., Zhu J. (2019). Angew. Chem., Int. Ed..

[cit7] Cheng X., Lu H., Lu Z. (2019). Nat. Commun..

[cit8] McManus J. B., Griffin J. D., White A. R., Nicewicz D. A. (2020). J. Am. Chem. Soc..

[cit9] Kato S., Saga Y., Kojima M., Fuse H., Matsunaga S., Fukatsu A., Kondo M., Masaoka S., Kanai M. (2017). J. Am. Chem. Soc..

[cit10] Lee B. J., DeGlopper K. S., Yoon T. P. (2019). Angew. Chem., Int. Ed..

[cit11] Cai C.-Y., Lai X.-L., Wang Y., Hu H.-H., Song J., Yang Y., Wang C., Xu H.-C. (2022). Nat. Catal..

[cit12] McCague R., Leclercq G., Legros N., Goodman J., Blackburn G. M., Jarman M., Foster A. B. (1989). J. Med. Chem..

[cit13] Kolb H. C., VanNieuwenhze M. S., Sharpless K. B. (1994). Chem. Rev..

[cit14] Maryanoff B. E., Reitz A. B. (1989). Chem. Rev..

[cit15] Li B.-J., Xu L., Wu Z.-H., Guan B.-T., Sun C.-L., Wang B.-Q., Shi Z.-J. (2009). J. Am. Chem. Soc..

[cit16] Ruzi R., Liu K., Zhu C., Xie J. (2020). Nat. Commun..

[cit17] Yue H., Zhu C., Huang L., Dewanji A., Rueping M. (2022). Chem. Commun..

[cit18] Xu W., Wang W., Liu T., Xie J., Zhu C. (2019). Nat. Commun..

[cit19] Deposition number CCDC 2312137 (for compound 7) contains the supplementary crystallographic data for this paper. These data are provided free of charge by the joint Cambridge Crystallographic Data Centre and Fachinformationszentrum Karlsruhe Access Structures service.†

