# Supplementary material for: Stereoselective benzylic C(sp3)–H alkenylation enabled by metallaphotoredox catalysis
Source: Chem Sci. 2024 Jul 8;15(31):12511–6. doi: 10.1039/d4sc02830a (PMC11304817; doi:10.1039/d4sc02830a)
Supplement: SC-015-D4SC02830A-s001 [file SC-015-D4SC02830A-s001.pdf]

Electronic Supplementary Material (ESI) for Chemical Science.
This journal is © The Royal Society of Chemistry 2024

SUPLUMMENTARY INFORMATION

Stereoselective Benzylic C(sp^3)-H Alkenylation Enabled by Metallaphotoredox catalysis

Yantao Li,a Haonan Bai,a Qi Gao,a Kai Liu,a Jie Han,a Weipeng Li,a Chengjian Zhu*ab, and Jin Xie*a

a. State Key Laboratory of Coordination Chemistry, Jiangsu Key Laboratory of Advanced Organic Materials, Chemistry and Biomedicine Innovation Center (ChemBIC), School of Chemistry and Chemical Engineering, Nanjing University, Nanjing 210023, China

b. Green Catalysis Center, and College of Chemistry, Zhengzhou University, Zhengzhou 450001, China

Table of Contents

1. General information 2
2. Preparation of substrates 2
3. General procedure 9
4. Characterization of products 11
5. Investigation of the reaction mechanism 37
   5.1 Radical inhibition experiments with TEMPO 37
   5.2 Byproducts detected experiments 37
   5.3 Kinetic experiments 38
   5.4 Luminescence quenching experiment 44
   5.5 The reactivity of Z/E vinyl triflates 44
6. NMR spectrum of substrates 49
7. NMR spectrum of products 78
8. HPLC analysis 144
9. Definition of the absolute configuration 160
10. DFT calculations 164
11. Reference 170

1. General information

All the reactions were conducted in oven-dried Schlenk tubes under air unless otherwise noted. All solvents were purified by Solvent Purification System. Reagents were purchased from Energy Chemical, TCI and etc. Flash column chromatographic purification of products was accomplished using forced-flow chromatography on Silica Gel (300-400 mesh). ^{1}H NMR, ^{13}C NMR and ^{19}F NMR spectra were recorded on a 400 MHz or 500 MHz spectrometer in $CDCl_3$ (δ H = 7.26 ppm, δ C = 77.0 ppm as standard). Data for ^{1}H NMR are reported as follows: chemical shift (ppm, scale), multiplicity, coupling constant (Hz), and integration. Data for ^{13}C NMR are reported in terms of chemical shift (ppm, scale), multiplicity, and coupling constant (Hz). Abbreviations for signal couplings are: s, singlet; d, doublet; t, triplet; m, multiple. GC-MS analyses were performed on a GC-MS with an EI mode. High resolution mass spectra were obtained using an Agilent 6210 Series TOF LC-MS equipped with electrospray ionization (ESI) probe operating in positive ion mode. Melting points (mp) were determined with a digital electrothermal apparatus without further correction. IR spectra were recorded on a Thermo Scientific Nicolet 380 FT-IR spectrometer. The 45 W blue LED lamps (λmax = 455 nm) were purchased from Kessil (A360NE/WE). High pressure liquid chromatography (HPLC) was performed on Agilent 1260 Series chromatographs using Daicel Chiralcel or Chiralpak columns (250 mm). Optical rotations were measured on a Rudolph Research Analytical Autopol VI automatic polarimeter using a 50 mm pathlength cell at 589 nm with $[\alpha]_D$ values reported in degrees; concentration (c) is in g/100 mL.

2. Preparation of substrates

Method A: General Procedure for the Synthesis of (*Z*)-Enol Triflates
The starting acetoacetate derivative (4 mmol) was added to a round-bottom flask and dissolved in either hexanes or toluene (20 mL, 0.2M). The solution was cooled with an ice bath to 5-10°C (internal temperature) followed by addition of a saturated aqueous solution of LiOH (6 mL, ~30 mmol) in one portion. The resulting biphasic mixture was *vigorously stirred* at 5-10°C for ~5 minutes followed by the addition of triflic anhydride (10 mmol) dropwise at a rate to maintain the internal temperature between 5-15 ºC. Upon completion of the reaction (as judged by TLC, typically <10 min), the biphasic solution was diluted with H_2O (5mL) and the layers were separated. The aqueous layer was extracted with EtOAc (1 x 10mL). The combined organic layers were washed with H_2O (1 x 5mL), brine (1 x 5mL), and dried over $MgSO_4$. The organic layer was filtered and concentrated under reduced pressure to yield the corresponding crude (*Z*)-enol triflate.[1]
Method B: General Procedure for the Synthesis of (*E*)-Enol Triflates
The starting acetoacetate derivative (4 mmol) was added to a round-bottom flask and dissolved in either hexanes or toluene (20 mL, 0.2M). The solution was cooled with an ice bath to 5-10°C (internal temperature), followed by addition of an aqueous solution of tetramethylammonium hydroxide (7.2 mL of a 25 wt% solution in water, 20 mmol) in one portion. The resulting

biphasic mixture was *vigorously stirred* at 5- 10°C for ~5 minutes followed by the addition of triflic anhydride (10 mmol) dropwise at a rate to maintain the internal temperature between 5-15 ºC. Upon completion of reaction (as judged by TLC, typically <10 min), the biphasic solution was diluted with H2O (5mL) and the layers were separated. The aqueous layer was extracted with EtOAc (1 x 10mL). The combined organic layers were washed with H_2O (1 x 5mL), brine (1 x 5mL), and dried over MgSO4. The organic layer was filtered and concentrated under reduced pressure to yield the corresponding crude (*E*)-enol triflate. [1]

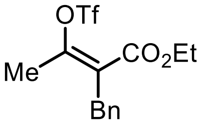

ethyl (Z)-2-benzyl-3-(((trifluoromethyl)sulfonyl)oxy)but-2-enoate (**2a**). [1] Method **A**. On 1.0 mmol scale; purified by flash column chromatography on SiO_2 (eluent: petroleum ether/ethyl acetate) to afford **2a**, 299.2 mg, 85%, light yellow oil; Rf = 0.5 (petroleum ether/ethyl acetate 10:1). ^{1}H NMR (400 MHz, Chloroform-*d*) δ 7.32 – 7.24 (m, 2H), 7.23 – 7.14 (m, 3H), 4.18 (q, *J* = 7.1 Hz, 2H), 3.72 (s, 2H), 2.18 (s, 3H), 1.19 (t, *J* = 7.1 Hz, 3H).^{13}C NMR (101 MHz, Chloroform-*d*) δ 164.88, 149.14 , 136.77 , 128.72 , 128.05 , 126.86 , 125.41 , 118.37 (q, *J* = 319.9 Hz), 61.78 , 35.02 , 17.80 , 13.75 . ^{19}F NMR (376 MHz, Chloroform-*d*) δ -74.73. ^{1}H NMR spectrum for compound **2a**

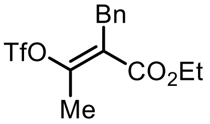

ethyl (E)-2-benzyl-3-(((trifluoromethyl)sulfonyl)oxy)but-2-enoate (**2a'**).[1] Method **B**. On 1.0 mmol scale; purified by flash column chromatography on SiO_2 (eluent: petroleum ether/ethyl acetate) to afford **2a'**, 232.3 mg, 66%, light yellow oil; Rf = 0.47 (petroleum ether/ethyl acetate 10:1). ^{1}H NMR (400 MHz, Chloroform-d) δ 7.30 – 7.23 (m, 2H), 7.22 – 7.17 m, 3H), 4.12 (q, J = 7.1 Hz, 2H), 3.81 (s, 2H), 2.46 (s, 3H), 1.14 (t, J = 7.1 Hz, 3H). ^{13}C NMR (101 MHz, Chloroform-*d*) δ 165.83, 153.70, 137.26, 128.50, 126.68, 126.40, 118.27 (q, *J* = 319.7 Hz), 61.54, 33.69, 18.93, 13.84. ^{19}F NMR (376 MHz, Chloroform-*d*) δ -74.45.

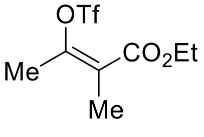

ethyl (Z)-2-methyl-3-(((trifluoromethyl)sulfonyl)oxy)but-2-enoate (**2b**).[1] Method **A**. On 1.0 mmol scale; purified by flash column chromatography on SiO_2 (eluent: petroleum ether/ethyl acetate) to afford **2b**, 234.6 mg, 85%, light yellow oil; Rf = 0.6 (petroleum ether/ethyl acetate 10:1).^{1}H NMR (400 MHz, Chloroform-*d*) δ 4.30 – 4.24 (m, 2H), 2.08 (d, *J* = 1.5 Hz, 3H), 1.92 (t, *J* = 1.4 Hz, 3H), 1.26 (t, *J* = 7.1 Hz, 3H). ^{13}C NMR (101 MHz, Chloroform-*d*) δ 165.21, 147.54 , 121.75 , 118.23 (q, *J* = 319.7 Hz), 61.54 , 17.41 , 14.98 , 13.67 . ^{19}F NMR (376 MHz, Chloroform-*d*) δ -75.08.

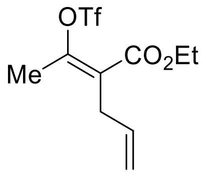

ethyl (Z)-2-(1-(((trifluoromethyl)sulfonyl)oxy)ethylidene)pent-4-enoate (**2c**).[1] Method **A**. On 1.0 mmol scale; purified by flash column chromatography on SiO_2 (eluent: petroleum ether/ethyl acetate) to afford **2c**, 235.6 mg, 78%, light yellow oil; Rf = 0.4 (petroleum ether/ethyl acetate 10:1).^{1}H NMR (400 MHz, Chloroform-*d*) δ 5.83 – 5.73(m, 1H), 5.15 – 5.09 (m, 2H), 4.27 (q, *J* = 7.2 Hz, 2H), 3.11 – 3.08(m, 2H), 2.13 (s, 3H), 1.31 (t, *J* = 7.1 Hz, 3H). ^{13}C NMR (101 MHz, Chloroform-*d*) δ 164.85, 149.00, 132.46 , 123.99 , 118.30 (q, *J* = 319.7 Hz), 116.95 , 61.81 , 33.25 , 17.56 , 13.90 . ^{19}F NMR (376 MHz, Chloroform-*d*) δ -74.67.

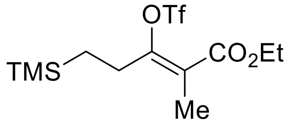

ethyl (Z)-2-methyl-3-(((trifluoromethyl)sulfonyl)oxy)-5-(trimethylsilyl)pent-2-enoate (**2d**).[1] Method **A**. On 1.0 mmol scale; purified by flash column chromatography on SiO_2 (eluent: petroleum ether/ethyl acetate) to afford **2d**, 314.9 mg, 87%, light yellow oil; Rf = 0.7 (petroleum ether/ethyl acetate 10:1). ^{1}H NMR (400 MHz, Chloroform-*d*) δ 4.26 (q, *J* = 7.1 Hz, 2H), 2.39 – 2.35 (m, 2H), 1.96 (s, 3H), 1.32 (t, *J* = 7.1 Hz, 3H), 0.81 – 0.77 (m, 2H), 0.03 (s, 9H). ^{13}C NMR (101 MHz, Chloroform-*d*) δ 165.59, 153.47 , 120.21 , 118.31 (q, *J* = 320.0 Hz), 61.64 , 25.81 , 14.87 , 13.84 , 13.39 , -2.22 . ^{19}F NMR (376 MHz, Chloroform-*d*) δ -74.95.

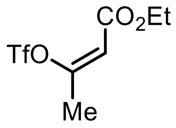

ethyl (Z)-3-(((trifluoromethyl)sulfonyl)oxy)but-2-enoate (**2e**).[1] Method **A**. On 1.0 mmol scale; purified by flash column chromatography on SiO_2 (eluent: petroleum ether/ethyl acetate) to afford **2d**, 152.0 mg, 58%, light yellow oil; Rf = 0.6 (petroleum ether/ethyl acetate 10:1). ^{1}H NMR (400 MHz, Chloroform-*d*) δ 5.77 (s, 1H), 4.25 (q, *J* = 7.1 Hz, 2H), 2.17 (s, 3H), 1.30 (t, *J* = 7.1 Hz, 3H). ^{13}C NMR (101 MHz, Chloroform-*d*) δ 162.28, 155.06, 118.30 (q, *J* = 319.8 Hz), 112.78, 61.18, 20.82, 13.96. ^{19}F NMR (376 MHz, Chloroform-*d*) δ -74.77.

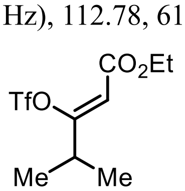

ethyl (Z)-4-methyl-3-(((trifluoromethyl)sulfonyl)oxy)pent-2-enoate (**2f**).[1] Method **A**. On 1.0 mmol scale; purified by flash column chromatography on SiO_2 (eluent: petroleum ether/ethyl acetate) to afford **2f**, 191.4 mg, 66%, light yellow oil; Rf = 0.7 (petroleum ether/ethyl acetate 10:1). ^{1}H NMR (400 MHz, Chloroform-*d*) δ 5.77 (s, 1H), 4.26 (q, *J* = 7.1 Hz, 2H), 2.62 (h, *J* = 6.8 Hz, 1H), 1.31 (t, *J* = 7.1 Hz, 3H), 1.21 (d, *J* = 6.7 Hz, 6H). ^{13}C NMR (101 MHz, Chloroform-*d*) δ 163.99, 162.76, 118.36 (q, *J* = 320.1 Hz), 109.81, 61.24, 33.34, 19.69, 13.99. ^{19}F NMR

(376 MHz, Chloroform-*d*) δ -74.67.

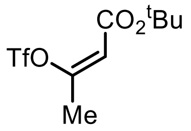

tert-butyl (Z)-3-(((trifluoromethyl)sulfonyl)oxy)but-2-enoate (**2g**).[1] Method **A**. On 1.0 mmol scale; purified by flash column chromatography on SiO_2 (eluent: petroleum ether/ethyl acetate) to afford **2g**, 246.5 mg, 85%, light yellow oil; Rf = 0.6 (petroleum ether/ethyl acetate 10:1). ^{1}H NMR (400 MHz, Chloroform-*d*) δ 5.69 (s, 1H), 2.12 (s, 3H), 1.50 (s, 9H). ^{13}C NMR (101 MHz, Chloroform-*d*) δ 161.53, 153.59, 118.32 (q, J = 319.9 Hz), 114.41, 82.33, 27.89, 20.55. ^{19}F NMR (376 MHz, Chloroform-*d*) δ -74.77.

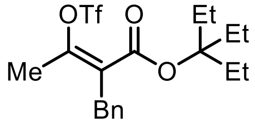

3-ethylpentan-3-yl (Z)-2-benzyl-3-(((trifluoromethyl)sulfonyl)oxy)but-2-enoate (**2h**). Method **A**. On 1.0 mmol scale; purified by flash column chromatography on SiO_2 (eluent: petroleum ether/ethyl acetate) to afford **2h**, 177.2 mg, 42%, light yellow oil; Rf = 0.7 (petroleum ether/ethyl acetate 10:1). ^{1}H NMR (400 MHz, Chloroform-*d*) δ 7.31 – 7.27 (m, 2H), 7.23 – 7.18 (m, 3H), 3.69 (s, 2H), 2.17 (s, 3H), 1.76 (q, J = 7.5 Hz, 6H), 0.66 (t, J = 7.5 Hz, 9H). ^{13}C NMR (101 MHz, Chloroform-*d*) δ 163.34, 148.40, 136.88, 128.65, 128.02, 126.76, 126.29, 118.39 (q, J = 320.0 Hz), 91.48, 35.27, 26.71, 17.56, 7.44. ^{19}F NMR (376 MHz, Chloroform-*d*) δ -74.50. HRMS (ESI) Calculated for $C_{19}H_{25}F_3O_5S$ [M+K]$^+$: 461.1006, found: 461.1002. IR ν (neat, cm^{-1}): 2998.5, 1759.4, 1497.1, 1200.8, 1124.3, 997.2, 873.0.

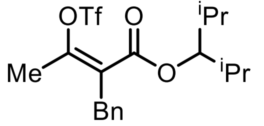

2,4-dimethylpentan-3-yl (Z)-2-benzyl-3-(((trifluoromethyl)sulfonyl)oxy)but-2-enoate (**2i**). Method **A**. On 1.0 mmol scale; purified by flash column chromatography on SiO_2 (eluent: petroleum ether/ethyl acetate) to afford **2i**, 265.9 mg, 63%, light yellow oil; Rf = 0.6 (petroleum ether/ethyl acetate 10:1). ^{1}H NMR (400 MHz, Chloroform-*d*) δ 7.29 (dd, J = 8.4, 6.4 Hz, 2H), 7.22 – 7.18 (m, 3H), 4.68 (t, J = 6.1 Hz, 1H), 3.77 (s, 2H), 2.21 (s, 3H), 1.88 – 1.77 (m, J = 6.7 Hz, 2H), 0.72 (dd, J = 9.7, 6.8 Hz, 12H). ^{13}C NMR (101 MHz, Chloroform-*d*) δ 164.29, 150.16, 136.85, 128.71, 127.89, 126.80, 124.87, 118.39 (q, J = 320.0 Hz), 84.58, 34.92, 29.20, 19.25, 17.98, 17.06. ^{19}F NMR (376 MHz, Chloroform-*d*) δ -74.51. HRMS (ESI) Calculated for $C_{19}H_{25}F_3O_5S$ [M+K]$^+$: 461.1006, found: 461.1003. IR ν (neat, cm^{-1}): 3001.4, 1700.5, 1492.5, 1240.8, 1200.5, 997.2, 805.2.

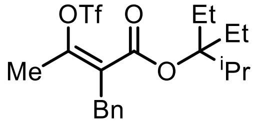

3-ethyl-2-methylpentan-3-yl (Z)-2-benzyl-3-(((trifluoromethyl)sulfonyl)oxy)but-2-enoate (**2j**). Method **A**. On 1.0 mmol scale; purified by flash column chromatography on SiO_2 (eluent: petroleum ether/ethyl acetate) to afford **2j**, 231.1 mg, 53%, light yellow oil; Rf = 0.6 (petroleum ether/ethyl acetate 10:1). ^{1}H NMR (400 MHz, Chloroform-*d*) δ 7.31 – 7.27 (m, 2H), 7.22 – 7.17 (m, 3H), 3.71 (s, 2H), 2.28 (hept, *J* = 7.0 Hz, 1H), 2.16 (s, 3H), 1.89 – 1.86 (m, *J* = 7.4 Hz, 4H), 0.80 (d, *J* = 7.0 Hz, 6H), 0.73 (t, *J* = 7.6 Hz, 6H). ^{13}C NMR (101 MHz, Chloroform-*d*) δ 163.46, 148.44, 136.77, 128.67, 127.94, 126.87 – 126.67 (m), 126.34, 118.40 (q, *J* = 319.9 Hz), 93.50, 35.16, 33.59, 26.68, 17.54, 8.56. ^{19}F NMR (376 MHz, Chloroform-*d*) δ -74.55. HRMS (ESI) Calculated for $C_{20}H_{27}F_3O_5S$ $[M+K]^+$: 471.1163, found: 471.1156. IR ν (neat, cm^{-1}): 3012.4, 1754.1, 1495.6, 1200.5, 1180.2, 997.5, 780.3.

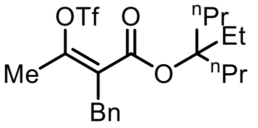

4-ethylheptan-4-yl (Z)-2-benzyl-3-(((trifluoromethyl)sulfonyl)oxy)but-2-enoate (**2k**). Method **A**. On 1.0 mmol scale; purified by flash column chromatography on SiO_2 (eluent: petroleum ether/ethyl acetate) to afford **2k**, 248.1 mg, 55%, light yellow oil; Rf = 0.6 (petroleum ether/ethyl acetate 10:1). ^{1}H NMR (400 MHz, Chloroform-*d*) δ 7.31 – 7.26 (m, 2H), 7.23 – 7.17 (m, 3H), 3.68 (s, 2H), 2.17 (s, 3H), 1.75 (q, *J* = 7.5 Hz, 2H), 1.69 – 1.64 (m, 4H), 1.11 – 1.01 (m, 4H), 0.79 (t, *J* = 7.3 Hz, 6H), 0.67 (t, *J* = 7.5 Hz, 3H). ^{13}C NMR (101 MHz, Chloroform-*d*) δ 163.40, 148.15, 136.82, 128.63, 128.05, 126.75, 126.41, 118.38 (q, *J* = 320.1 Hz), 91.00, 37.02, 35.29, 27.77, 17.51, 16.40, 14.38, 7.60. ^{19}F NMR (376 MHz, Chloroform-*d*) δ -74.49. HRMS (ESI) Calculated for $C_{21}H_{29}F_3O_5S$ $[M+K]^+$: 489.1319, found: 489.1312. IR ν (neat, cm^{-1}): 3080.5, 1780.4, 1490.2, 1250.3, 1197.8, 998.1, 785.6, 605.4.

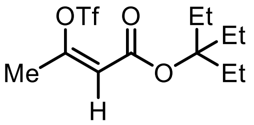

3-ethylpentan-3-yl (Z)-3-(((trifluoromethyl)sulfonyl)oxy)but-2-enoate (**2l**). Method **A**. On 1.0 mmol scale; purified by flash column chromatography on SiO_2 (eluent: petroleum ether/ethyl acetate) to afford **2l**, 285.5 mg, 86%, light yellow oil; Rf = 0.4 (petroleum ether/ethyl acetate 10:1). ^{1}H NMR (400 MHz, Chloroform-*d*) δ 5.73 (d, *J* = 1.1 Hz, 1H), 2.13 (d, *J* = 0.9 Hz, 3H), 1.87 (q, *J* = 7.5 Hz, 6H), 0.82 (t, *J* = 7.5 Hz, 9H). ^{13}C NMR (101 MHz, Chloroform-*d*) δ 161.12, 154.16, 118.33 (q, *J* = 319.8 Hz), 114.03, 90.41, 26.64, 20.66, 7.51. ^{19}F NMR (376 MHz, Chloroform-*d*) δ -74.78. HRMS (ESI) Calculated for $C_{12}H_{19}F_3O_5S$ $[M+K]^+$: 371.0537, found: 371.0534. IR ν (neat, cm^{-1}): 2987.5, 1756.3, 1500.2, 1250.7, 1244.9, 750.2, 698.7.

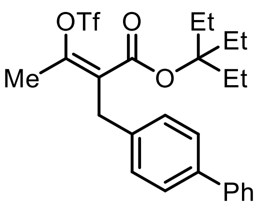

3-ethylpentan-3-yl (Z)-2-([1,1'-biphenyl]-4-ylmethyl)-3-(((trifluoromethyl)sulfonyl)oxy)but-2-enoate (**2m**). Method **A**. On 1.0 mmol scale; purified by flash column chromatography on SiO_2

(eluent: petroleum ether/ethyl acetate) to afford **2m**, 194.2 mg, 39%, light yellow oil; Rf = 0.6 (petroleum ether/ethyl acetate 10:1). ^{1}H NMR (400 MHz, Chloroform-*d*) δ 7.57 – 7.52 (m, 4H), 7.43 (m, 7.44 – 7.41, 2H), 7.33 (m, 7.35 – 7.31, 1H), 7.26 (m, 7.27 – 7.24, 2H), 3.73 (s, 2H), 2.19 (s, 3H), 1.77 (q, *J* = 7.5 Hz, 6H), 0.67 (t, *J* = 7.5 Hz, 9H). ^{13}C NMR (101 MHz, Chloroform-*d*) δ 163.38, 148.40, 140.79, 139.79, 135.99, 128.79, 128.49, 127.39, 127.27, 127.00, 118.42 (q, *J* = 319.9 Hz), 91.62, 34.99, 26.75, 17.62, 7.49. ^{19}F NMR (376 MHz, Chloroform-*d*) δ -74.42. HRMS (ESI) Calculated for $C_{25}H_{29}F_3O_5S$ $[M+Na]^+$: 521.1580, found: 521.1571. IR ν (neat, cm^{-1}): 2893.1, 1834.2, 1572.1, 1207.3, 996.2, 758.7.

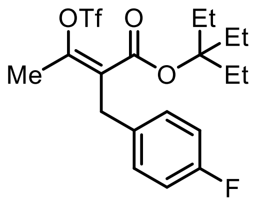

3-ethylpentan-3-yl (Z)-2-(4-fluorobenzyl)-3-(((trifluoromethyl)sulfonyl)oxy)but-2-enoate (**2n**). Method **A**. On 1.0 mmol scale; purified by flash column chromatography on SiO_2 (eluent: petroleum ether/ethyl acetate) to afford **2n**, 250.8 mg, 57%, light yellow oil; Rf = 0.6 (petroleum ether/ethyl acetate 10:1). ^{1}H NMR (400 MHz, Chloroform-*d*) δ 7.17 – 7.14 (m, 2H), 7.00 – 6.96 (m, 2H), 3.66 (s, 2H), 2.17 (s, 3H), 1.76 (q, *J* = 7.5 Hz, 6H), 0.67 (t, *J* = 7.5 Hz, 9H). ^{13}C NMR (101 MHz, Chloroform-*d*) δ 163.10 (d, *J* = 21.5 Hz), 160.56, 148.42, 132.56 (d, *J* = 3.3 Hz), 129.51 (d, *J* = 8.0 Hz), 126.23, 118.37 (q, *J* = 319.9 Hz), 115.45 (d, *J* = 21.4 Hz), 91.64, 34.51, 26.69, 17.50, 7.42. ^{19}F NMR (376 MHz, Chloroform-*d*) δ -74.51, -116.24. HRMS (ESI) Calculated for $C_{19}H_{24}F_4O_5S$ $[M+K]^+$: 479.0912, found: 479.0906. IR ν (neat, cm^{-1}): 3001.2, 1748.3, 1500.4, 1480.6, 1238.1, 804.2, 731.2.

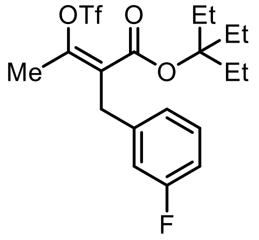

3-ethylpentan-3-yl (Z)-2-(4-fluorobenzyl)-3-(((trifluoromethyl)sulfonyl)oxy)but-2-enoate (**2o**). Method **A**. On 1.0 mmol scale; purified by flash column chromatography on SiO_2 (eluent: petroleum ether/ethyl acetate) to afford **2o**, 233.5 mg, 53%, light yellow oil; Rf = 0.8 (petroleum ether/ethyl acetate 10:1). ^{1}H NMR (400 MHz, Chloroform-*d*) δ 7.29 – 7.23 (m, 1H), 6.99 – 6.97 (m, 1H), 6.93 – 6.89 (m, 1H), 3.69 (s, 2H), 2.17 (s, 3H), 1.77 (q, *J* = 7.5 Hz, 6H), 0.68 (t, *J* = 7.5 Hz, 9H). ^{13}C NMR (101 MHz, Chloroform-*d*) δ 163.65 (d, *J* = 117.6 Hz), 161.79, 148.88, 139.51 (d, *J* = 7.3 Hz), 130.15 (d, *J* = 8.3 Hz), 125.69, 123.62 (d, *J* = 2.9 Hz), 118.37 (q, *J* = 320.0 Hz), 115.03 (d, *J* = 22.0 Hz), 113.70 (d, *J* = 20.9 Hz), 91.71, 34.96, 26.68, 17.60, 7.41. ^{19}F NMR (376 MHz, Chloroform-*d*) δ -74.50, -101.37 – -116.29 (m). HRMS (ESI) Calculated for $C_{19}H_{24}F_4O_5S$ $[M+K]^+$: 479.0912, found: 479.0911. IR ν (neat, cm^{-1}): 2997.3, 1748.2, 1518.2, 1476.8, 1203.7, 1145.8, 824.2.

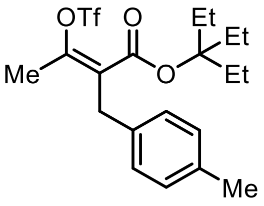

3-ethylpentan-3-yl (Z)-2-(4-methylbenzyl)-3-(((trifluoromethyl)sulfonyl)oxy)but-2-enoate (**2p**). Method **A**. On 1.0 mmol scale; purified by flash column chromatography on SiO_2 (eluent: petroleum ether/ethyl acetate) to afford **2p**, 266.0 mg, 61%, light yellow oil; Rf = 0.5 (petroleum ether/ethyl acetate 10:1). ^{1}H NMR (500 MHz, Chloroform-*d*) δ 7.08 (q, *J* = 8.2 Hz, 3H), 3.65 (s, 2H), 2.31 (s, 3H), 2.15 (s, 3H), 1.77 (q, *J* = 7.5 Hz, 6H), 0.66 (s, 9H). ^{13}C NMR (126 MHz, Chloroform-d) δ 163.44, 148.16, 136.29, 133.71, 129.28, 127.88, 126.48, 118.36 (q, J = 320.5 Hz), 91.43, 34.8, 26.71, 20.97, 17.51, 7.46. ^{19}F NMR (471 MHz, Chloroform-*d*) δ -74.46. HRMS (ESI) Calculated for $C_{20}H_{27}F_3O_5S$ $[M+Na]^+$: 459.1424, found: 459.1427. IR ν (neat, cm^{-1}): 2890.2, 1740.2, 1404.2, 1240.6, 1128.1, 689.4.

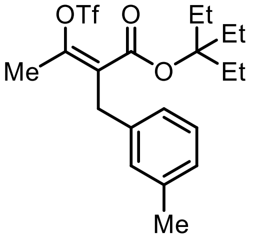

3-ethylpentan-3-yl (Z)-2-(3-methylbenzyl)-3-(((trifluoromethyl)sulfonyl)oxy)but-2-enoate (**2q**). Method **A**. On 1.0 mmol scale; purified by flash column chromatography on SiO_2 (eluent: petroleum ether/ethyl acetate) to afford **2q**, 204.9 mg, 47%, light yellow oil; Rf = 0.5 (petroleum ether/ethyl acetate 10:1). ^{1}H NMR (400 MHz, Chloroform-*d*) δ 7.17 (t, *J* = 7.5 Hz, 1H), 7.03 – 6.97 (m, 3H), 3.65 (s, 2H), 2.31 (s, 3H), 2.16 (s, 3H), 1.76 (q, *J* = 7.5 Hz, 6H), 0.68 (t, *J* = 7.5 Hz, 9H). ^{13}C NMR (101 MHz, Chloroform-*d*) δ 163.41, 148.26, 138.23, 136.79, 128.84, 128.53, 127.44, 126.43, 125.10, 118.41 (q, *J* = 320.0 Hz), 91.42, 35.17, 26.74, 21.31, 17.53, 7.42. ^{19}F NMR (376 MHz, Chloroform-*d*) δ -74.58. HRMS (ESI) Calculated for $C_{20}H_{27}F_3O_5S$ $[M+Na]^+$: 459.1424, found: 459.1418. IR ν (neat, cm^{-1}): 3010.2, 1756.1, 1487.5, 1201.7, 1170.5, 998.3, 697.2.

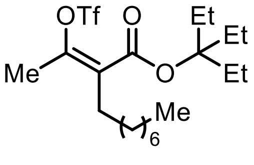

3-ethylpentan-3-yl (Z)-2-(1-(((trifluoromethyl)sulfonyl)oxy)ethylidene)decanoate (**2r**). Method **A**. On 1.0 mmol scale; purified by flash column chromatography on SiO_2 (eluent: petroleum ether/ethyl acetate) to afford **2r**, 160.2 mg, 36%, light yellow oil; Rf = 0.8 (petroleum ether/ethyl acetate 10:1). ^{1}H NMR (400 MHz, Chloroform-*d*) δ 2.29 – 2.25 (m, 2H), 2.10 (s, 3H), 1.90 (q, *J* = 7.5 Hz, 6H), 1.52 – 1.45 (m, 2H), 1.35 – 1.26 (m, 10H), 0.90 – 0.83(m, 12H). ^{13}C NMR (101 MHz, Chloroform-*d*) δ 164.12, 145.99, 128.17, 118.35 (q, *J* = 320.0 Hz), 91.16,

31.78, 29.90, 29.26, 29.18, 29.13, 28.31, 26.86, 22.63, 16.89, 14.04, 7.67. ^{19}F NMR (376 MHz, Chloroform-*d*) δ -74.62. HRMS (ESI) Calculated for $C_{20}H_{35}F_3O_5S$ $[M+K]^+$: 483.1789, found: 483.1779. IR ν (neat, cm^{-1}): 2997.2, 1763.8, 1578.5, 1302.5, 985.2.

3. General procedure

General procedure A (Standard conditions)

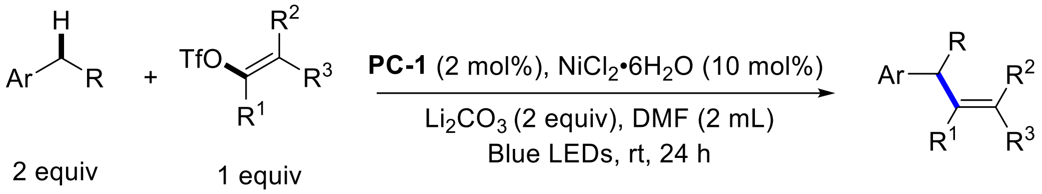

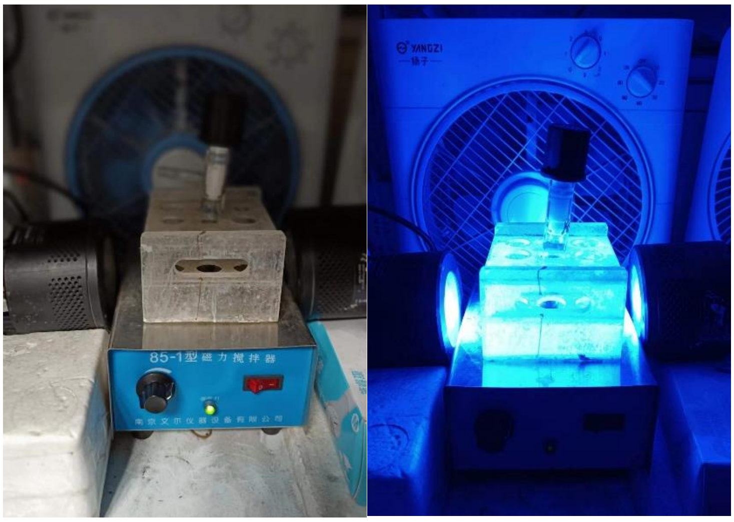

Supplementary Figure 1. Reaction device

Photocatalyst $[Ir(dF(CF_3)ppy)_2(4,4'\text{-}dCF_3bpy)]PF_6$ (4.6 mg, 2 mol%), benzylic compound (0.4 mmol, 2 equiv), alkenyl triflates (0.2 mmol, 1 equiv), $NiCl_2$•$6H_2O$ (4.8 mg, 10 mol%), and anhydrous powder Li_2CO_3 (29.6 mg, 0.4 mmol, 2 equiv) were added to an oven-dried 10 mL Schlenk tube equipped with a magnetic stirring bar. The tube was evacuated and backfilled with argon three times. Subsequently, DMF (2 mL) was added under argon. The tube was then sealed and placed ~5 cm from 2 × 45 W blue LEDs. The reaction mixture was stirred for 24 h at room temperature (air-condition was used to keep the temperature is 25 °C or so). After completion, the reaction mixture was removed from the light, diluted with water and the aqueous layer was extracted with EtOAc (3 × 2 mL). The combined organic layers were washed with brine, dried over anhydrous Na_2SO_4, filtered, and concentrated. The residue was purified by flash chromatography on silica gel to afford the corresponding products.

General procedure B

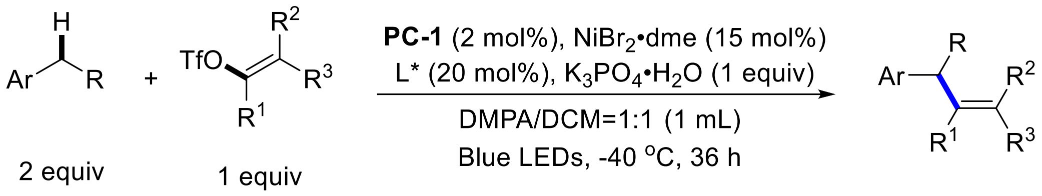

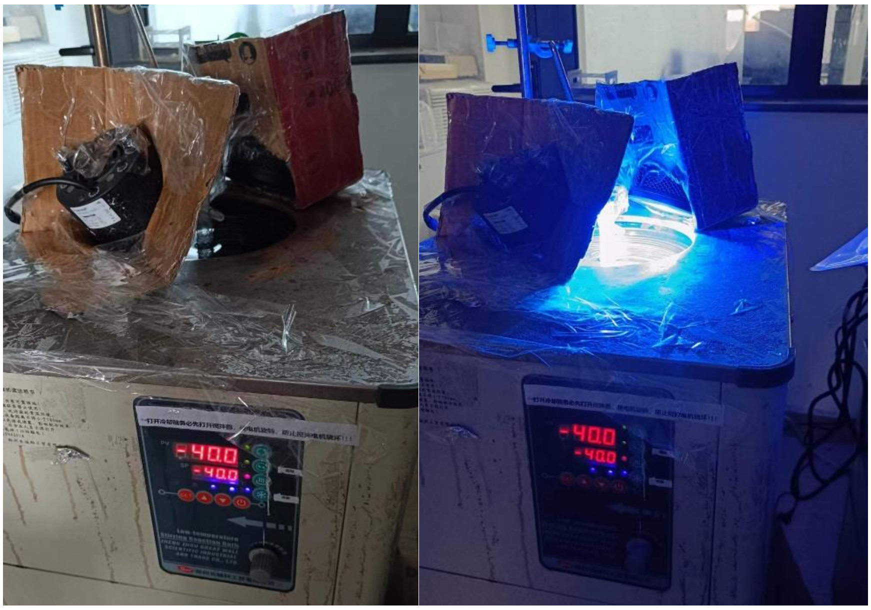

Supplementary Figure 2. Reaction device

Preparation of Ni-based catalyst solution: In the nitrogen-filled glove box, a stirring bar, $NiBr_2$·dme (4.6 mg, 15 mol%), (3aS,3'aS,8aR,8'a'R)-2,2'-cyclopropylidenebis[3a,8a-dihydro-8H-Indeno[1,2-d]oxazole (7.1 mg, 20 mol%) and N,N-Dimethylpropionamide/DCM (1 mL, V/V = 1:1) were successively added to an oven-dried vial (8 mL screw-cap threaded). The vial was then sealed with a Teflon-lined plastic screw-cap and stirred until the resulting mixture become homogeneous (about 20 min). Photocatalyst [Ir(dF(CF_3)ppy)$_2$(4,4'-dCF_3bpy)]PF_6 (2.3 mg, 2 mol%), and K_3PO_4·H_2O (23.0 mg, 0.1 mmol, 1 equiv) were added to an oven-dried 10 mL Schlenk tube equipped with a magnetic stirring bar. The tube was evacuated and backfilled with argon three times. Subsequently, the nickel-catalyst solution was transferred into this Schlenk tube under argon. Then benzylic compound (0.2 mmol, 2 equiv), alkenyl triflates (0.1 mmol, 1 equiv) were added. The tube was then sealed and placed ~5 cm from 2 × 45 W blue LEDs. The reaction mixture was stirred for 36 h at -40 ºC. After completion, the reaction mixture was removed from the light, diluted with water and the aqueous layer was extracted with DCM (3 × 2 mL). The combined organic layers were washed with brine, dried over anhydrous Na_2SO_4, filtered, and concentrated. The residue was purified by flash chromatography on silica gel to afford the corresponding ketone products.

Gram-scale reaction

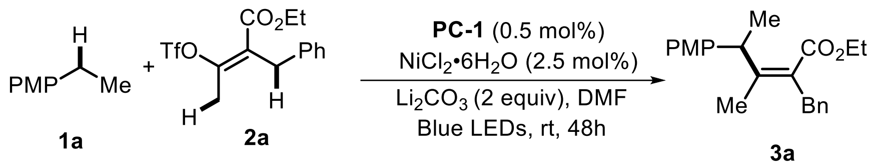

Photocatalyst $[Ir(dF(CF_3)ppy)_2(4,4'\text{-}dCF_3bpy)]PF_6$ (17.2 mg, 0.5 mol%), benzylic compound (6 mmol, 2 equiv), alkenyl triflates (3 mmol, 1 equiv), $NiCl_2•6H_2O$ (17.9 mg, 2.5 mol%), and anhydrous powder Li_2CO_3 (444 mg, 6 mmol, 2 equiv) were added to an oven-dried 100 mL Schlenk tube equipped with a magnetic stirring bar. The tube was evacuated and backfilled with argon three times. Subsequently, DMF (30 mL) was added under argon. The tube was then sealed and placed ~5 cm from 2 × 45 W blue LEDs. The reaction mixture was stirred for 48 h at room temperature (air-condition was used to keep the temperature is 25 °C or so). After completion, the reaction mixture was removed from the light, diluted with water and the aqueous layer was extracted with EtOAc (3 × 20 mL). The combined organic layers were washed with brine, dried over anhydrous Na_2SO_4, filtered, and concentrated. The residue was purified by flash chromatography on silica gel to afford the corresponding products in 79% yield (0.8011g).

4. Characterization of products

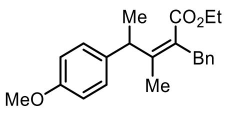

ethyl (Z)-2-benzyl-4-(4-methoxyphenyl)-3-methylpent-2-enoate (**3a**). The reaction was carried out according to the general procedure A on 0.2 mmol scale (24 h); purified by flash column chromatography on SiO_2 (eluent: petroleum ether/ethyl acetate) to afford **3a**, 57.5 mg, 85%, light yellow oil; Rf = 0.8 (petroleum ether/ethyl acetate 10:1). ^{1}H NMR (400 MHz, Chloroform-*d*) δ 7.28 – 7.14 (m, 7H), 6.87 – 6.83 (m, 2H), 4.51 (q, J = 7.0 Hz, 1H), 4.13 (q, J = 7.1 Hz, 2H), 3.78 (s, 3H), δ 3.69 (d, J = 6.6 Hz, 1H), 1.51 (s, 3H), 1.43 (d, J = 7.0 Hz, 3H), 1.17 (t, J = 7.1 Hz, 3H). ^{13}C NMR (101 MHz, Chloroform-*d*) δ 169.8, 157.9, 148.3, 139.4, 135.3, 128.4(2C), 128.3(2C), 128.1(2C), 127.1, 125.9, 113.5(2C), 60.3, 55.2, 40.9, 35.9, 16.9, 14.3, 14.1. HRMS (ESI) Calculated for $C_{22}H_{26}O_3$ $[M+H]^+$: 339.1955, found: 339.1947. IR ν (neat, cm^{-1}): 2970, 1708, 1610, 1510, 1454, 1247, 1178, 1077, 1033, 833, 699.

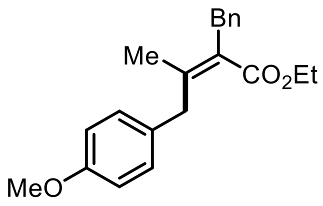

ethyl (Z)-2-benzyl-4-(4-methoxyphenyl)-3-methylbut-2-enoate (**3b**). The reaction was carried out according to the general procedure A on 0.2 mmol scale (24 h); purified by flash column chromatography on SiO_2 (eluent: petroleum ether/ethyl acetate) to afford **3b**, 48.0 mg, 74%,

light yellow oil; Rf = 0.8 (petroleum ether/ethyl acetate 10:1). ^{1}H NMR (400 MHz, Chloroform-*d*) δ 7.28 – 7.14 (m, 7H), 6.83 (d, *J* = 8.6 Hz, 2H), 4.12 (q, *J* = 7.1 Hz, 2H), 3.78 (s, 3H), 3.74 (s, 2H), 3.68 (s, 2H), 1.74 (s, 3H), 1.16 (t, *J* = 7.1 Hz, 3H). ^{13}C NMR (101 MHz, Chloroform-*d*) δ 169.3, 158.0, 144.8, 139.5, 131.4, 129.9(2C), 128.3(2C), 128.2(2C), 128.1, 125.9, 113.7(2C), 60.3, 55.2, 41.0, 35.8, 19.3, 14.1. HRMS (ESI) Calculated for $C_{21}H_{24}O_3$ $[M+H]^+$: 325.1798, found: 325.1790. IR ν (neat, cm^{-1}): 2916, 1705, 1509, 1246, 1174, 1096, 1034, 698, 670.

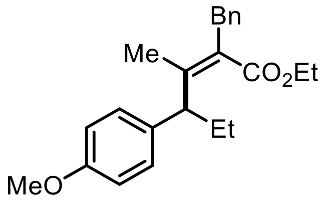

ethyl (Z)-2-benzyl-4-(4-methoxyphenyl)-3-methylhex-2-enoate (**3c**). The reaction was carried out according to the general procedure A on 0.2 mmol scale (24 h); purified by flash column chromatography on SiO_2 (eluent: petroleum ether/ethyl acetate) to afford **3c**, 42.2 mg, 60%, light yellow oil; Rf = 0.8 (petroleum ether/ethyl acetate 10:1). ^{1}H NMR (400 MHz, Chloroform-*d*) δ 7.28 – 7.14 (m, 7H), 6.87 – 6.83 (m, 2H), 4.26 (dd, *J* = 9.2, 6.2 Hz, 1H), 4.15 (q, *J* = 6.8 Hz, 2H), 3.79 (s, 3H), 3.69 (d, *J* = 5.3 Hz, 2H), 1.94 – 1.74 (m, 2H), 1.50 (s, 3H), 1.19 (t, *J* = 7.1 Hz, 3H), 0.94 (t, *J* = 7.3 Hz, 3H). ^{13}C NMR (101 MHz, Chloroform-*d*) δ 170.0, 157.9, 146.3, 139.4, 134.7, 128.9(2C), 128.3, 128.3(2C), 128.2(2C), 125.9, 113.5(2C), 60.2, 55.2, 48.3, 36.1, 23.7, 14.2, 13.8, 12.2. HRMS (ESI) Calculated for $C_{23}H_{28}O_3$ $[M+H]^+$: 353.2111, found: 353.2105. IR ν (neat, cm^{-1}): 2961, 2932, 1709, 1510, 1494, 1246, 1178, 1086, 1033, 815, 699.

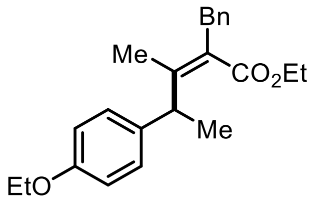

ethyl (Z)-2-benzyl-4-(4-ethoxyphenyl)-3-methylpent-2-enoate (**3d**). The reaction was carried out according to the general procedure A on 0.2 mmol scale (24 h); purified by flash column chromatography on SiO_2 (eluent: petroleum ether/ethyl acetate) to afford **3d**, 59.1 mg, 84%, light yellow oil; Rf = 0.8 (petroleum ether/ethyl acetate 10:1). ^{1}H NMR (400 MHz, Chloroform-*d*) δ 7.27 – 7.14 (m, 7H), 6.84 (d, *J* = 8.7 Hz, 2H), 4.50 (q, *J* = 7.0 Hz, 1H), 4.13 (q, *J* = 7.1 Hz, 2H), 4.01 (q, *J* = 7.0 Hz, 2H), 3.69 (d, *J* = 6.6 Hz, 2H), 1.50 (s, 3H), 1.43 – 1.38 (m, 6H), 1.17 (t, *J* = 7.1 Hz, 3H). ^{13}C NMR (101 MHz, Chloroform-*d*) δ 169.8, 157.3, 148.3, 139.4, 135.2, 128.4(2C), 128.3(2C), 128.1(2C), 127.0, 125.9, 114.1(2C), 63.3, 60.2, 40.9, 35.9, 16.9, 14.8, 14.3, 14.1. HRMS (ESI) Calculated for $C_{23}H_{28}O_3$ $[M+H]^+$: 353.2111, found: 353.2104. IR ν (neat, cm^{-1}): 2977, 2930, 1708, 1509, 1243, 1177, 1077, 1046, 807, 697.

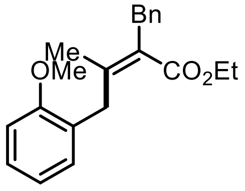

ethyl (Z)-2-benzyl-4-(2-methoxyphenyl)-3-methylbut-2-enoate (**3e**). The reaction was carried

out according to the general procedure A on 0.2 mmol scale (24 h); purified by flash column chromatography on SiO_2 (eluent: petroleum ether/ethyl acetate) to afford **3e**, 36.9 mg, 57%, light yellow oil; Rf = 0.8 (petroleum ether/ethyl acetate 10:1). ^{1}H NMR (400 MHz, Chloroform-*d*) δ 7.28 – 7.15 (m, 7H), 6.91 – 6.83 (m, 2H), 4.11 (q, *J* = 7.1 Hz, 2H), 3.81 (s, 2H), 3.80 (s, 3H), 3.75 (s, 2H), 1.74 (s, 3H), 1.14 (t, *J* = 7.1 Hz, 3H). ^{13}C NMR (101 MHz, Chloroform-*d*) δ 169.3, 157.6, 145.0, 139.8, 129.9, 128.3, 128.3(2C), 128.2(2C), 127.8, 127.2, 125.9, 120.4, 110.2, 60.1, 55.2, 35.9, 35.2, 19.5, 14.1. HRMS (ESI) Calculated for $C_{21}H_{24}O_3$ $[M+H]^+$: 325.1798, found: 325.1790. IR ν (neat, cm^{-1}): 2931, 1709, 1600, 1455, 1242, 1183, 1114, 1076, 1030, 752, 698.

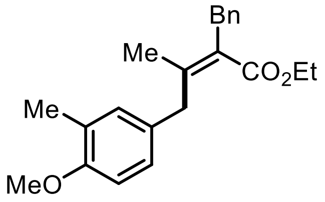

ethyl (Z)-2-benzyl-4-(4-methoxy-3-methylphenyl)-3-methylbut-2-enoate (**3f**). The reaction was carried out according to the general procedure A on 0.2 mmol scale (24 h); purified by flash column chromatography on SiO_2 (eluent: petroleum ether/ethyl acetate) to afford **3f**, 55.4mg, 82%, light yellow oil; Rf = 0.8 (petroleum ether/ethyl acetate 10:1). ^{1}H NMR (400 MHz, Chloroform-*d*) δ 7.28 – 7.15 (m, 5H), 7.04 (d, *J* = 7.5 Hz, 2H), 6.74 (d, *J* = 8.1 Hz, 1H), 4.12 (q, *J* = 7.1 Hz, 2H), 3.79 (s, 3H), 3.74 (s, 2H), 3.65 (s, 2H), 2.20 (s, 3H), 1.16 (t, *J* = 7.1 Hz, 3H). ^{13}C NMR (101 MHz, Chloroform-*d*) δ 169.3, 156.2, 145.0, 139.6, 131.3, 131.0, 128.3(2C), 128.2(2C), 128.0, 127.0, 126.3, 125.9, 109.8, 60.2, 55.3, 41.0, 35.8, 19.4, 16.2, 14.1. HRMS (ESI) Calculated for $C_{22}H_{26}O_3$ $[M+H]^+$: 339.1955, found: 339.1947. IR ν (neat, cm^{-1}): 2934, 1708, 1608, 1498, 1453, 1289, 1255, 1197, 1181, 1050, 863, 699.

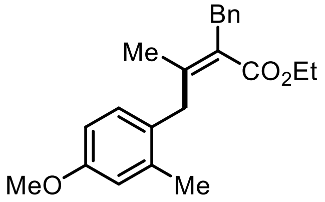

ethyl (Z)-2-benzyl-4-(4-methoxy-2-methylphenyl)-3-methylbut-2-enoate (**3g**). The reaction was carried out according to the general procedure A on 0.2 mmol scale (24 h); purified by flash column chromatography on SiO_2 (eluent: petroleum ether/ethyl acetate) to afford **3g**, 60.2 mg, 89%, light yellow oil; Rf = 0.8 (petroleum ether/ethyl acetate 10:1). ^{1}H NMR (400 MHz, Chloroform-*d*) δ 7.36 – 7.15 (m, 5H), 7.05 (d, *J* = 8.1 Hz, 1H), 6.70 (m, 6.70 – 6.67, 2H), 4.10 (q, *J* = 7.1 Hz, 2H), 3.76 (s, 5H), 3.72 (s, 2H), 2.25 (s, 3H), 1.71 (s, 3H), 1.13 (t, *J* = 7.1 Hz, 3H). ^{13}C NMR (101 MHz, Chloroform-*d*) δ 169.2, 157.8, 145.0, 139.7, 137.8, 129.9, 129.7, 128.6, 128.3, 128.2, 125.9, 115.7, 111.0, 60.2, 55.1, 38.1, 35.8, 19.9, 19.5, 14.1. HRMS (ESI) Calculated for $C_{22}H_{26}O_3$ $[M+H]^+$: 339.1944, found: 339.1947. IR ν (neat, cm^{-1}): 2978, 2936, 1710, 1608, 1499, 1453, 1289, 1198, 1051, 699.

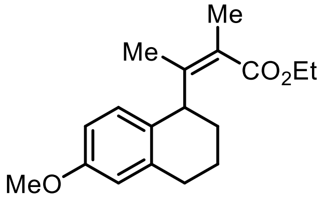

ethyl (Z)-3-(6-methoxy-1,2,3,4-tetrahydronaphthalen-1-yl)-2-methylbut-2-enoate (**3h**). The reaction was carried out according to the general procedure A on 0.2 mmol scale (24 h); purified by flash column chromatography on SiO_2 (eluent: petroleum ether/ethyl acetate) to afford **3h**, 41.5 mg, 72%, light yellow oil; Rf = 0.9 (petroleum ether/ethyl acetate 10:1). ^{1}H NMR (400 MHz, Chloroform-*d*) δ 6.91 (d, *J* = 8.6 Hz, 1H), 6.66 (dd, *J* = 8.4, 2.9 Hz, 1H), 6.60 (d, *J* = 2.8 Hz, 1H), 4.30 (dd, *J* = 10.4, 5.6 Hz, 1H), 4.21 – 4.13 (m, 2H), 3.76 (s, 3H), 2.78 – 2.72 (m, 2H), 1.92 (d, *J* = 1.0 Hz, 5H), 1.73 – 1.58 (m, 2H), 1.48 (q, *J* = 1.1 Hz, 3H), 1.28 (t, *J* = 7.1 Hz, 3H). ^{13}C NMR (101 MHz, Chloroform-*d*) δ 170.4, 157.4, 146.0, 138.8, 130.0, 129.6, 125.3, 113.4, 112.1, 60.2, 55.1, 43.7, 30.4, 28.7, 22.7, 16.0, 16.0, 14.2. HRMS (ESI) Calculated for $C_{18}H_{24}O_3$ $[M+H]^+$: 289.1798, found: 289.1788. IR ν (neat, cm^{-1}): 2932, 1710, 1609, 1500, 1278, 1258, 1217, 1092, 1040.

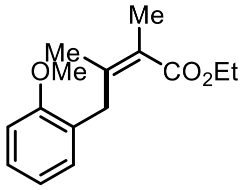

ethyl (Z)-4-(2-methoxyphenyl)-2,3-dimethylpent-2-enoate (**3i**). The reaction was carried out according to the general procedure A on 0.2 mmol scale (24 h); purified by flash column chromatography on SiO_2 (eluent: petroleum ether/ethyl acetate) to afford **3i**, 22.0 mg, 42%, light yellow oil; Rf = 0.8 (petroleum ether/ethyl acetate 10:1). ^{1}H NMR (400 MHz, Chloroform-*d*) δ 7.25 (dd, *J* = 7.0, 1.2 Hz, 1H), 7.18 (td, *J* = 7.8, 1.7 Hz, 1H), 6.93 (td, *J* = 7.5, 1.2 Hz, 1H), 6.78 (dd, *J* = 8.2, 1.2 Hz, 1H), 4.79 (q, *J* = 7.2 Hz, 1H), 4.33 – 4.18 (m, 2H), 3.70 (s, 3H), 1.83 (s, 3H), 1.45 (s, 3H), 1.39 (d, *J* = 7.2 Hz, 3H), 1.33 (t, *J* = 7.2 Hz, 3H). ^{13}C NMR (101 MHz, Chloroform-*d*) δ 170.4, 157.7, 145.5, 132.3, 127.5, 127.1, 123.1, 120.0, 110.2, 59.9, 55.2, 36.1, 17.0, 16.3, 14.7, 14.3. HRMS (ESI) Calculated for $C_{16}H_{22}O_3$ $[M+H]^+$: 263.1642, found: 263.1633. IR ν (neat, cm^{-1}): 2924, 1712, 1632, 1440, 1275, 1244, 1207, 1106, 1029, 753.

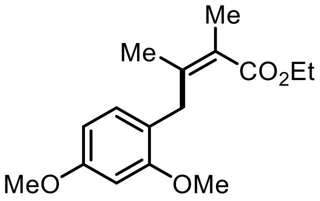

ethyl (Z)-4-(2,4-dimethoxyphenyl)-2,3-dimethylbut-2-enoate (**3j**). The reaction was carried out according to the general procedure A on 0.2 mmol scale (24 h); purified by flash column chromatography on SiO_2 (eluent: petroleum ether/ethyl acetate) to afford **3j**, 44.5 mg, 80%, light yellow oil; Rf = 0.8 (petroleum ether/ethyl acetate 10:1). ^{1}H NMR (400 MHz, Chloroform-*d*) δ 7.03 (dd, *J* = 7.8, 0.9 Hz, 1H), 6.44 – 6.40 (m, 2H), 4.19 (q, *J* = 7.2 Hz, 2H), 3.78 (m, 6H), 3.65 (s, 2H), 1.91 (s, 3H), 1.66 (s, 3H), 1.27 (t, *J* = 7.1 Hz, 3H). ^{13}C NMR (101 MHz, Chloroform-*d*) δ 170.1, 159.2, 158.4, 143.6, 130.0, 124.2, 120.3, 60.1, 55.3, 55.3, 34.2, 19.3, 16.0, 14.2. HRMS (ESI) Calculated for $C_{16}H_{22}O_4$ $[M+H]^+$: 279.1591, found: 279.1582. IR ν (neat, cm^{-1}): 2935, 1709, 1612, 1505, 1463, 1289, 1208, 1156, 1038, 833.

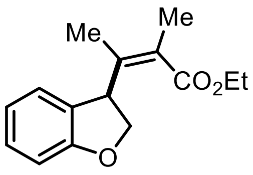

ethyl (Z)-3-(2,3-dihydrobenzofuran-3-yl)-2-methylbut-2-enoate (**3k**). The reaction was carried out according to the general procedure A on 0.2 mmol scale (24 h); purified by flash column chromatography on SiO_2 (eluent: petroleum ether/ethyl acetate) to afford **3k**, 43.8 mg, 89%, light yellow oil; Rf = 0.9 (petroleum ether/ethyl acetate 10:1). ^{1}H NMR (400 MHz, Chloroform-*d*) δ 7.17 – 7.08 (m, 2H), 6.94 – 6.74 (m, 2H), 5.04 (dd, *J* = 9.9, 6.5 Hz, 1H), 4.73 – 4.64 (m, 1H), 4.32 (dd, *J* = 9.2, 6.4 Hz, 1H), 4.23 (q, *J* = 7.1 Hz, 2H), 1.92 (s, 3H), 1.60 (s, 3H), 1.33 (t, *J* = 7.2 Hz, 3H). ^{13}C NMR (101 MHz, Chloroform-*d*) δ 169.6, 160.4, 128.8, 128.4, 125.8, 125.1, 120.5, 109.5, 75.1, 60.5, 46.5, 16.1, 15.2, 14.2. HRMS (ESI) Calculated for $C_{15}H_{18}O_3$ $[M+H]^+$: 247.1329, found: 247.1321. IR ν (neat, cm^{-1}): 2980, 1706, 1596, 1482, 1277, 1231, 1094, 973, 750.

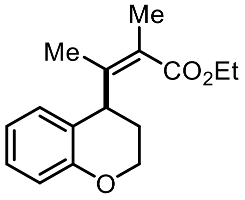

ethyl (Z)-3-(chroman-4-yl)-2-methylbut-2-enoate (**3l**).The reaction was carried out according to the general procedure A on 0.2 mmol scale (24 h); purified by flash column chromatography on SiO_2 (eluent: petroleum ether/ethyl acetate) to afford **3l**, 46.8 mg, 90%, light yellow oil; Rf = 0.8 (petroleum ether/ethyl acetate 10:1). ^{1}H NMR (400 MHz, Chloroform-*d*) δ 7.10 – 7.06 (m, 1H), 6.94 – 6.92 (m, 1H), 6.84 – 6.79 (m, 2H), 4.57 – 4.53 (m, 1H), 4.34 (dt, *J* = 10.9, 3.1 Hz, 1H), 4.25 – 4.14 (m, 2H), 4.06 (td, *J* = 11.8, 2.1 Hz, 1H), 2.10 – 1.99 (m, 2H), 1.94 (s, 3H), 1.51 (s, 3H), 1.29 (t, *J* = 7.1 Hz, 3H). ^{13}C NMR (101 MHz, Chloroform-*d*) δ 170.0, 155.5, 144.4, 129.1, 127.5, 126.7, 123.8, 120.5, 116.8, 65.7, 60.4, 39.9, 27.4, 16.1, 15.8, 14.2. HRMS (ESI) Calculated for $C_{16}H_{20}O_3$ $[M+H]^+$: 261.1485, found: 261.1475. IR ν (neat, cm^{-1}): 2977, 2929, 1708, 1581, 1487, 1452, 1273, 1255, 1118, 1062, 1014, 756.

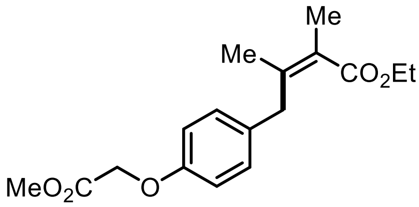

ethyl (Z)-4-(4-(2-methoxy-2-oxoethoxy)phenyl)-2,3-dimethylbut-2-enoate (**3m**). The reaction was carried out according to the general procedure A on 0.2 mmol scale (24 h); purified by flash column chromatography on SiO_2 (eluent: petroleum ether/ethyl acetate) to afford **3m**, 50.8 mg, 83%, light yellow oil; Rf = 0.5 (petroleum ether/ethyl acetate 10:1). ^{1}H NMR (400 MHz, Chloroform-*d*) δ 7.16 (d, *J* = 8.8 Hz, 2H), 6.82 (d, *J* = 8.8 Hz, 2H), 4.61 (s, 2H), 4.21 (q, *J* = 7.2 Hz, 2H), 3.80 (s, 3H), 3.62 (s, 2H), 1.91 (s, 3H), 1.66 (s, 3H), 1.29 (t, *J* = 7.2 Hz, 3H). ^{13}C NMR (101 MHz, Chloroform-*d*) δ 169.9, 169.5, 156.2, 143.1, 132.8, 129.9, 124.3, 114.5, 65.4, 60.2, 52.2, 40.7, 19.3, 15.9, 14.2. HRMS (ESI) Calculated for $C_{17}H_{22}O_5$ $[M+H]^+$: 307.1540,

found: 307.1531. IR ν (neat, cm^{-1}): 2920, 1762, 1706, 1509, 1438, 1273, 1203, 1173, 1102,

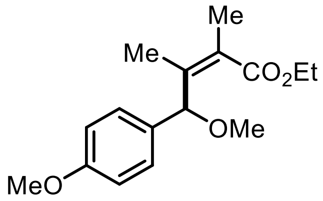

ethyl (Z)-4-methoxy-4-(4-methoxyphenyl)-2,3-dimethylbut-2-enoate (**3n**). The reaction was carried out according to the general procedure A on 0.2 mmol scale (24 h); purified by flash column chromatography on SiO_2 (eluent: petroleum ether/ethyl acetate) to afford **3n**, 36.1 mg, 65%, light yellow oil; Rf = 0.8 (petroleum ether/ethyl acetate 10:1). ^{1}H NMR (400 MHz, Chloroform-*d*) δ 7.34 (d, *J* = 8.9 Hz, 2H), 6.85 (d, *J* = 8.8 Hz, 2H), 5.51 (s, 1H), 4.26 (q, *J* = 7.2 Hz, 2H), 3.79 (s, 3H), 3.32 (s, 3H), 1.92 (d, *J* = 1.1 Hz, 3H), 1.57 (d, *J* = 1.1 Hz, 3H), 1.33 (t, *J* = 7.2 Hz, 3H). ^{13}C NMR (101 MHz, Chloroform-*d*) δ 169.8, 158.6, 142.5, 132.6, 127.2, 127.0, 113.4, 80.9, 60.5, 56.3, 55.2, 16.1, 14.3, 12.6. HRMS (ESI) Calculated for $C_{16}H_{22}O_4$ [M+H]$^+$: 279.1591, found: 279.1582. IR ν (neat, cm^{-1}): 2931, 1710, 1612, 1510, 1463, 1301, 1246, 1213, 1093, 1035, 836.

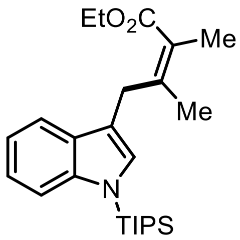

ethyl (Z)-2,3-dimethyl-4-(1-(triisopropylsilyl)-1H-indol-3-yl)but-2-enoate (**3o**). The reaction was carried out according to the general procedure A on 0.2 mmol scale (24 h); purified by flash column chromatography on SiO_2 (eluent: petroleum ether/ethyl acetate) to afford **3o**, 34.8 mg, 42%, light yellow oil; Rf = 0.9 (petroleum ether/ethyl acetate 10:1). ^{1}H NMR (400 MHz, Chloroform-*d*) δ 7.62 – 7.60 (m, 1H), 7.47 – 7.44 (m, 1H), 7.14 – 7.06 (m, 2H), 7.02 (s, 1H), 4.25 (q, *J* = 7.2 Hz, 2H), 3.82 (s, 2H), 1.92 (d, *J* = 1.0 Hz, 3H), 1.72 – 1.64 (m, 6H), 1.30 (t, *J* = 7.1 Hz, 3H), 1.13 (d, *J* = 7.6 Hz, 18H). ^{13}C NMR (101 MHz, Chloroform-*d*) δ 170.4, 143.4, 141.3, 131.2, 129.5, 123.5, 121.2, 119.3, 119.0, 115.6, 113.8, 60.2, 31.5, 19.2, 18.1, 16.1, 14.3, 12.8. HRMS (ESI) Calculated for $C_{25}H_{39}NO_2Si$ [M+H]$^+$: 414.2823, found: 414.2812. IR ν (neat, cm^{-1}): 2947, 2868, 1711, 1450, 1173, 1140, 1088, 740.

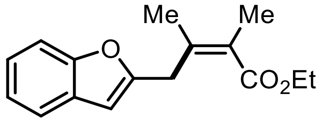

ethyl (Z)-4-(benzofuran-2-yl)-2,3-dimethylbut-2-enoate (**3p**). The reaction was carried out according to the general procedure A on 0.2 mmol scale (24 h); purified by flash column chromatography on SiO_2 (eluent: petroleum ether/ethyl acetate) to afford **3p**, 23.7 mg, 46%, light yellow oil; Rf = 0.9 (petroleum ether/ethyl acetate 10:1). ^{1}H NMR (400 MHz, Chloroform-*d*) δ 7.48 – 7.45 (m, 1H), 7.42 – 7.39 (m, 1H), 7.22 – 7.14 (m, 2H), 6.43 (d, *J* = 1.1 Hz, 1H), 4.22 (q, *J* = 7.2 Hz, 2H), 3.93 (s, 2H), 1.94 (s, 3H), 1.87 (s, 3H), 1.29 (t, *J* = 7.2 Hz, 3H). ^{13}C NMR (101 MHz, Chloroform-*d*) δ 169.2, 156.8, 154.7, 140.7, 128.9, 125.5, 123.2, 122.4, 120.3,

110.8, 103.1, 60.4, 34.7, 20.2, 16.0, 14.2. HRMS (ESI) Calculated for $C_{16}H_{18}O_3$ $[M+H]^+$: 259.1329, found: 259.1320. IR ν (neat, cm^{-1}): 2929, 1709, 1454, 1297, 1271, 1159, 1084, 751.

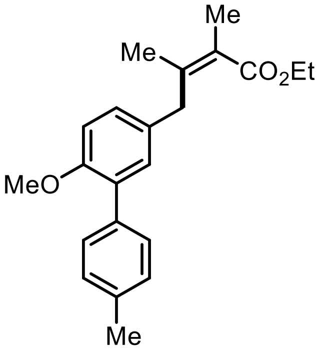

ethyl (Z)-4-(6-methoxy-4'-methyl-[1,1'-biphenyl]-3-yl)-2,3-dimethylbut-2-enoate (**3q**). The reaction was carried out according to the general procedure A on 0.2 mmol scale (24 h); purified by flash column chromatography on SiO_2 (eluent: petroleum ether/ethyl acetate) to afford **3q**, 48.7 mg, 72%, light yellow oil; Rf = 0.8 (petroleum ether/ethyl acetate 10:1). ^{1}H NMR (400 MHz, Chloroform-*d*) δ 7.42 (d, *J* = 7.9 Hz, 2H), 7.22 – 7.15 (m, 4H), 6.88 (d, *J* = 8.1 Hz, 1H), 4.21 (q, *J* = 7.1 Hz, 1H), 3.76 (s, 3H), 3.67 (s, 2H), 2.38 (s, 3H), 1.90 (s, 3H), 1.70 (s, 3H), 1.29 (t, *J* = 7.1 Hz, 3H). ^{13}C NMR (101 MHz, Chloroform-*d*) δ 169.9, 154.9, 143.2, 136.5, 135.7, 131.7, 131.3, 130.3, 129.4(2C), 128.7(2C), 128.6, 124.2, 111.1, 60.2, 55.6, 40.7, 21.1, 19.3, 16.0, 14.3. HRMS (ESI) Calculated for $C_{22}H_{26}O_3$ $[M+H]^+$: 339.1955, found: 339.1947. IR ν (neat, cm^{-1}): 2923, 1709, 1518, 1494, 1296, 1239, 1172, 1105, 1044, 821.

11,

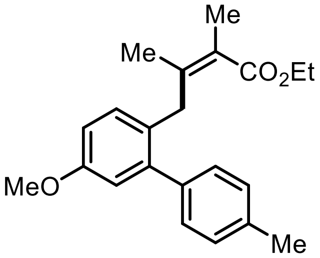

ethyl (Z)-4-(5-methoxy-4'-methyl-[1,1'-biphenyl]-2-yl)-2,3-dimethylbut-2-enoate (**3r**). The reaction was carried out according to the general procedure A on 0.2 mmol scale (24 h); purified by flash column chromatography on SiO_2 (eluent: petroleum ether/ethyl acetate) to afford **3r**, 49.3 mg, 73%, light yellow oil; Rf = 0.8 (petroleum ether/ethyl acetate 10:1). ^{1}H NMR (400 MHz, Chloroform-*d*) δ 7.12 – 7.07 (m, 5H), 6.75 (dd, *J* = 8.6, 2.8 Hz, 1H), 6.68 (d, *J* = 2.8 Hz, 1H), 4.08 (q, *J* = 7.2 Hz, 2H), 3.70 (s, 3H), 3.56 (s, 2H), 2.30 (s, 3H), 1.77 (d, *J* = 1.0 Hz, 3H), 1.41 (d, *J* = 1.1 Hz, 3H), 1.18 (t, *J* = 7.1 Hz, 3H). ^{13}C NMR (101 MHz, Chloroform-*d*) δ 169.7, 157.5, 144.2, 143.3, 138.8, 136.5, 129.9, 129.1, 1289.0, 128.7, 124.4, 115.0, 113.1, 60.1, 55.2, 37.8, 21.1, 19.3, 15.8, 14.2. HRMS (ESI) Calculated for $C_{22}H_{26}O_3$ $[M+H]^+$: 339.1955, found: 339.1947. IR ν (neat, cm^{-1}): 2932, 1708, 1606, 1493, 1296, 1271, 1221, 1175, 1103, 1082, 1017, 823.

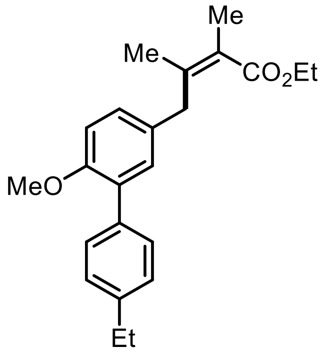

ethyl (Z)-4-(4'-ethyl-6-methoxy-[1,1'-biphenyl]-3-yl)-2,3-dimethylbut-2-enoate (**3s**). The reaction was carried out according to the general procedure A on 0.2 mmol scale (24 h); purified by flash column chromatography on SiO_2 (eluent: petroleum ether/ethyl acetate) to afford **3s**, 49.3 mg, 70%, light yellow oil; Rf = 0.8 (petroleum ether/ethyl acetate 10:1). ^{1}H NMR (400 MHz, Chloroform-*d*) δ 7.45 (d, *J* = 8.1 Hz, 2H), 7.26 – 7.20 (m, 2), 7.21 – 7.12 (m, 2H), 6.88 (d, *J* = 8.2 Hz, 1H), 4.20 (t, *J* = 7.2 Hz, 2H), 3.77 (s, 3H), 3.67 (s, 2H), 2.68 (q, *J* = 7.6 Hz, 2H), 1.90 (s, 3H), 1.69 (s, 3H), 1.28 (q, *J* = 7.1 Hz, 6H). ^{13}C NMR (101 MHz, Chloroform-*d*) δ 169.9, 154.9, 143.2, 142.7, 135.9, 131.7, 131.4, 130.3, 129.4, 128.6, 127.4, 124.2, 111.1, 60.2, 55.6, 40.8, 28.5, 19.3, 16.0, 15.4, 14.3. HRMS (ESI) Calculated for $C_{23}H_{28}O_3$ $[M+H]^+$: 353.2111, found: 353.2105. IR ν (neat, cm^{-1}): 2963, 2931, 1710, 1517, 1494, 1267, 1241, 1146, 1083, 1028, 834.

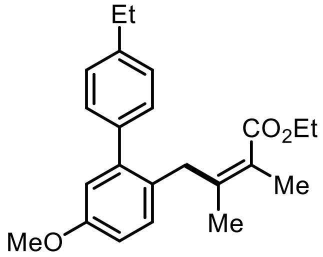

ethyl (Z)-4-(4'-ethyl-5-methoxy-[1,1'-biphenyl]-2-yl)-2,3-dimethylbut-2-enoate (**3t**). The reaction was carried out according to the general procedure A on 0.2 mmol scale (24 h); purified by flash column chromatography on SiO_2 (eluent: petroleum ether/ethyl acetate) to afford **3t**, 52.1 mg, 74%, light yellow oil; Rf = 0.8 (petroleum ether/ethyl acetate 10:1). ^{1}H NMR (400 MHz, Chloroform-*d*) δ 7.21 – 7.16 (m, 5H), δ 6.83 (dd, *J* = 8.6, 2.8 Hz, 1H), 6.77 (d, *J* = 2.8 Hz, 1H), 4.17 (q, *J* = 7.2 Hz, 2H), 3.79 (s, 3H), 3.64 (s, 2H), 2.69 (q, *J* = 7.6 Hz, 2H), 1.85 (s, 3H), 1.49 (s, 3H), 1.30 – 1.24 (m, 6H). ^{13}C NMR (101 MHz, Chloroform-*d*) δ 169.8, 157.5, 144.2, 143.4, 142.8, 139.0, 129.9, 129.2, 129.0, 127.5, 124.4, 114.9, 113.1, 60.1, 55.2, 37.8, 28.5, 19.4, 15.8, 15.5, 14.2. HRMS (ESI) Calculated for $C_{23}H_{28}O_3$ $[M+H]^+$: 353.2111, found: 353.2102. IR ν (neat, cm^{-1}): 2963, 3933, 1707, 1605, 1492, 1295, 1271, 1174, 1102, 1016, 835.

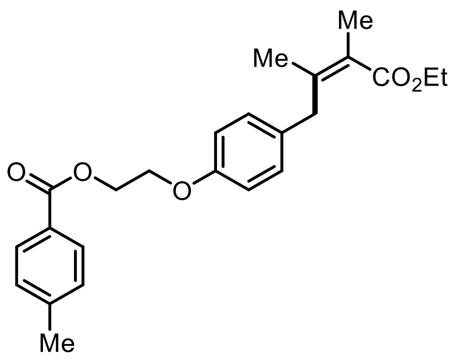

(Z)-2-(4-(4-ethoxy-2,3-dimethyl-4-oxobut-2-en-1-yl)phenoxy)ethyl 4-methylbenzoate (**3u**). The reaction was carried out according to the general procedure A on 0.2 mmol scale (24 h); purified by flash column chromatography on SiO_2 (eluent: petroleum ether/ethyl acetate) to afford **3u**, 57.8 mg, 73%, light yellow oil; Rf = 0.6 (petroleum ether/ethyl acetate 10:1). ^{1}H NMR (400 MHz, Chloroform-*d*) δ 7.97 (d, *J* = 8.3 Hz, 2H), 7.25 (d, *J* = 7.8 Hz, 2H), 7.19 (d, *J* = 8.7 Hz, 2H), 6.89 (d, *J* = 8.7 Hz, 2H), 4.69 – 4.64 (m, 2H), 4.35 – 4.29 (m, 2H), 4.25 (q, *J* = 7.1 Hz, 2H), 3.66 (s, 2H), 2.42 (s, 3H), 1.94 (s, 3H), 1.69 (s, 3H), 1.32 (t, *J* = 7.1 Hz, 3H). ^{13}C NMR (101 MHz, Chloroform-*d*) δ 169.9, 166.5, 157.0, 143.7, 143.2, 132.1, 129.9, 129.7, 1289.0, 127.1, 124.1, 114.5, 66.0, 63.2, 60.2, 40.7, 21.6, 19.2, 15.9, 14.2. HRMS (ESI) Calculated for $C_{24}H_{28}O_5$ $[M+H]^+$: 397.2010, found: 397.2002. IR ν (neat, cm^{-1}): 2926, 1713, 1611, 1509, 1272, 1240, 1176, 1103, 777.

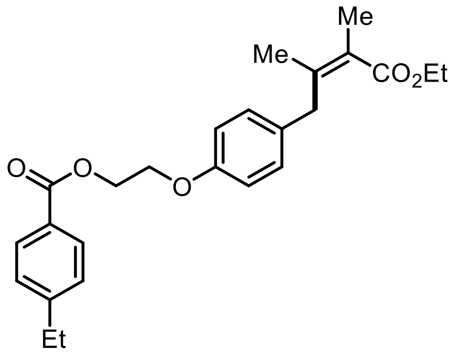

(Z)-2-(4-(4-ethoxy-2,3-dimethyl-4-oxobut-2-en-1-yl)phenoxy)ethyl 4-ethylbenzoate (**3v**). The reaction was carried out according to the general procedure A on 0.2 mmol scale (24 h); purified by flash column chromatography on SiO_2 (eluent: petroleum ether/ethyl acetate) to afford **3v**, 67.4 mg, 82%, light yellow oil; Rf = 0.6 (petroleum ether/ethyl acetate 10:1). ^{1}H NMR (400 MHz, Chloroform-*d*) δ 7.97 (d, *J* = 8.3 Hz, 2H), 7.25 (d, *J* = 8.4 Hz, 2H), 7.16 (d, *J* = 8.7 Hz, 2H), 6.86 (d, *J* = 8.7 Hz, 2H), 4.63 (t, *J* = 4.9 Hz, 2H), 4.27 (t, *J* = 4.8 Hz, 2H), 4.22 (q, *J* = 7.1 Hz, 2H), 3.63 (s, 2H), 2.69 (q, *J* = 7.6 Hz, 2H), 1.91 (s, 13H), 1.66 (s, 3H), 1.31 – 1.23 (m, 6H). ^{13}C NMR (101 MHz, Chloroform-*d*) δ 169.9, 166.6, 157.0, 149.9, 143.2, 132.2, 129.9, 129.8, 127.8, 127.4, 124.2, 114.6, 66.1, 63.2, 60.2, 40.7, 28.9, 19.2, 15.9, 15.2, 14.2. HRMS (ESI) Calculated for $C_{25}H_{30}O_5$ $[M+H]^+$: 411.2166, found: 411.2155. IR ν (neat, cm^{-1}): 2967, 1715, 1611, 1510, 1274, 1242, 1177, 1104, 853.

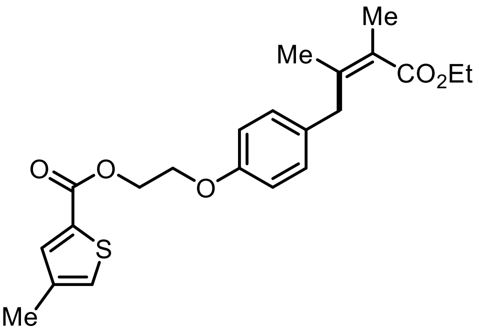

(Z)-2-(4-(4-ethoxy-2,3-dimethyl-4-oxobut-2-en-1-yl)phenoxy)ethyl 4-methylthiophene-2-carboxylate (**3w**). The reaction was carried out according to the general procedure A on 0.2 mmol scale (24 h); purified by flash column chromatography on SiO_2 (eluent: petroleum ether/ethyl acetate) to afford **3w**, 52.4 mg, 65%, light yellow oil; Rf = 0.5 (petroleum ether/ethyl acetate 10:1). ^{1}H NMR (400 MHz, Chloroform-*d*) δ 7.61 (d, *J* = 1.7 Hz, 1H), 7.17 – 7.13 (m, 3H), 6.88 – 6.84 (m, 2H), 4.60 (dd, *J* = 5.5, 4.3 Hz, 2H), 4.26 – 4.19 (m, 4H), 3.62 (s, 2H), 2.26 (s, 3H), 1.91 (s, 3H), 1.66 (s, 3H), 1.29 (t, *J* = 7.2 Hz, 3H). ^{13}C NMR (101 MHz, Chloroform-*d*) δ 169.9, 162.1, 156.9, 143.2, 138.4, 135.5, 132.8, 132.2, 129.9, 128.4, 124.1, 114.5, 65.9, 63.3, 60.2, 40.7, 19.2, 15.9, 15.4, 14.2. HRMS (ESI) Calculated for $C_{22}H_{26}O_5S$ $[M+H]^+$: 403.1574, found: 403.1567. IR ν (neat, cm^{-1}): 2926, 1706, 1509, 1429, 1276, 1234, 1175, 1081, 951, 772.

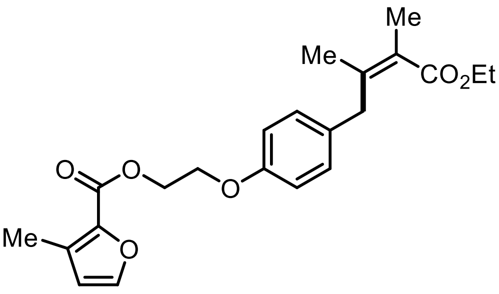

(Z)-2-(4-(4-ethoxy-2,3-dimethyl-4-oxobut-2-en-1-yl)phenoxy)ethyl 3-methylfuran-2-carboxylate (**3x**). The reaction was carried out according to the general procedure A on 0.2 mmol scale (24 h); purified by flash column chromatography on SiO_2 (eluent: petroleum ether/ethyl acetate) to afford **3x**, 54.0 mg, 70%, light yellow oil; Rf = 0.4 (petroleum ether/ethyl acetate 10:1). ^{1}H NMR (400 MHz, Chloroform-*d*) δ 7.44 (d, *J* = 1.8 Hz, 1H), 7.15 (d, *J* = 8.7 Hz, 2H), 6.85 (d, *J* = 8.8 Hz, 2H), 6.35 (d, *J* = 1.8 Hz, 1H), 4.64 – 4.62 (m, 2H), 4.27 – 4.18 (m, 4H), 3.62 (s, 2H), 2.35 (s, 3H), 1.91 (q, *J* = 1.1 Hz, 3H), 1.66 (q, *J* = 1.1 Hz, 3H), 1.29 (t, *J* = 7.1 Hz, 3H). ^{13}C NMR (101 MHz, Chloroform-*d*) δ 169.9, 159.3, 156.9, 145.1, 143.2, 132.2, 131.5, 129.9, 129.4, 124.1, 115.1, 114.5, 65.9, 62.7, 60.2, 40.7, 19.2, 15.9, 14.2, 11.5. HRMS (ESI) Calculated for $C_{22}H_{26}O_6$ $[M+H]^+$: 387.1802, found: 387.1795. IR ν (neat, cm^{-1}): 2928, 1705, 1509, 1290, 1242, 1177, 1098, 1073, 929, 776.

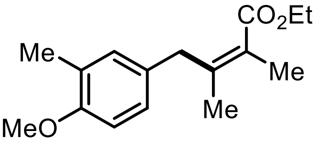

ethyl (Z)-4-(4-methoxy-3-methylphenyl)-2,3-dimethylbut-2-enoate (**3y**).The reaction was

carried out according to the general procedure A on 0.2 mmol scale (24 h); purified by flash column chromatography on SiO_2 (eluent: petroleum ether/ethyl acetate) to afford **3y**, 46.6 mg, 89%, light yellow oil; Rf = 0.9 (petroleum ether/ethyl acetate 10:1). ^{1}H NMR (400 MHz, Chloroform-*d*) δ 7.05 – 6.97 (m, 2H), 6.72 (d, *J* = 8.8 Hz, 1H), 4.22 (q, *J* = 7.2 Hz, 2H), 3.79 (s, 3H), 3.59 (s, 2H), 2.19 (s, 3H), 1.90 (q, *J* = 1.0 Hz, 3H), 1.66 (q, *J* = 1.0 Hz, 3H), 1.30 (t, *J* = 7.2 Hz, 3H).
^{13}C NMR (101 MHz, Chloroform-*d*) δ 170.0, 156.1, 143.4, 131.2, 131.1, 126.9, 126.3, 123.9, 109.7, 60.2, 55.2, 40.7, 19.2, 16.2, 15.9, 14.2. HRMS (ESI) Calculated for $C_{16}H_{22}O_3$ $[M+H]^+$: 263.1642, found: 263.1632. IR ν (neat, cm^{-1}): 2928, 1710, 1504, 1464, 1443, 1273, 1252, 1174, 1105, 1035.

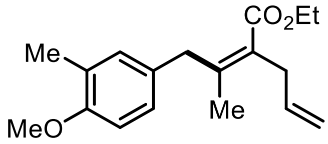

ethyl (Z)-2-(1-(4-methoxy-3-methylphenyl)propan-2-ylidene)pent-4-enoate (**3z**). The reaction was carried out according to the general procedure A on 0.2 mmol scale (24 h); purified by flash column chromatography on SiO_2 (eluent: petroleum ether/ethyl acetate) to afford **3z**, 49.0 mg, 85%, light yellow oil; Rf = 0.9 (petroleum ether/ethyl acetate 10:1). ^{1}H NMR (400 MHz, Chloroform-*d*) δ 7.03 – 7.01 (m, 2H), 6.73 (d, *J* = 8.8 Hz, 1H), 5.87 – 5.77 (m, 1H), 5.09 – 4.99 (m, 2H), 4.22 (q, *J* = 7.1 Hz, 2H), 3.79 (s, 3H), 3.60 (s, 2H), 3.10 (d, *J* = 6.1 Hz, 2H), 2.19 (s, 3H), 1.68 (s, 3H), 1.28 (t, *J* = 7.2 Hz, 3H). ^{13}C NMR (101 MHz, Chloroform-*d*) δ 169.4, 156.2, 144.7, 135.1, 131.3, 131.0, 127.0, 126.6, 126.3, 115.3, 109.8, 60.2, 55.3, 40.9, 34.3, 18.8, 16.2, 14.2. HRMS (ESI) Calculated for $C_{18}H_{24}O_3$ $[M+H]^+$: 289.1798, found: 289.1789. IR ν (neat, cm^{-1}): 2979, 1710, 1504, 1442, 1274, 1202, 1134, 1036, 913, 807.

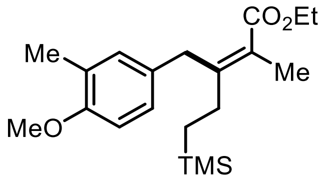

ethyl (E)-3-(4-methoxy-3-methylbenzyl)-2-methyl-5-(trimethylsilyl)pent-2-enoate (**3aa**). The reaction was carried out according to the general procedure A on 0.2 mmol scale (24 h); purified by flash column chromatography on SiO_2 (eluent: petroleum ether/ethyl acetate) to afford **3aa**, 60.0 mg, 86%, light yellow oil; Rf = 0.9 (petroleum ether/ethyl acetate 10:1). ^{1}H NMR (400 MHz, Chloroform-*d*) δ 7.02 – 6.99 (m, 2H), 6.73 (d, *J* = 8.9 Hz, 1H), 4.22 (q, *J* = 7.2 Hz, 2H), 3.80 (s, 3H), 3.62 (s, 2H), 2.20 (s, 3H), 1.99 – 1.95 (m, 2H), 1.91 (s, 3H), 1.30 (t, *J* = 7.2 Hz, 3H), 0.59 – 0.55 (m, 2H), -0.02 (s, 9H). ^{13}C NMR (101 MHz, Chloroform-*d*) δ 170.3, 156.1, 150.2, 131.4, 127.1, 126.2, 122.6, 109.8, 60.2, 55.3, 37.8, 26.2, 16.2, 15.3, 14.8, 14.3, -2.0. HRMS (ESI) Calculated for $C_{20}H_{32}O_3Si$ $[M+H]^+$: 349.2193, found: 349.2178. IR ν (neat, cm^{-1}): 2952, 1711, 1504, 1250, 1175, 1092, 1036, 861, 836.

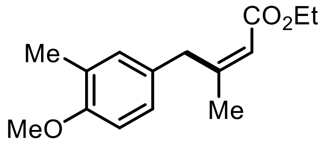

ethyl (Z)-4-(4-methoxy-3-methylphenyl)-3-methylbut-2-enoate (**3bb**). The reaction was carried out according to the general procedure A on 0.2 mmol scale (24 h); purified by flash column chromatography on SiO_2 (eluent: petroleum ether/ethyl acetate) to afford **3bb**, 36.2 mg, 73%, light yellow oil; Rf = 0.9 (petroleum ether/ethyl acetate 10:1). ^{1}H NMR (400 MHz, Chloroform-*d*) δ 7.03 – 7.00 (m, 2H), 6.72 (d, *J* = 8.1 Hz, 1H), 5.74 (s, 1H), 4.19 (q, *J* = 7.1 Hz, 2H), 3.92 (s, 2H), 3.79 (s, 3H), 2.18 (s, 3H), 1.78 (s, 3H), 1.29 (t, *J* = 7.2 Hz, 3H). ^{13}C NMR (101 MHz, Chloroform-*d*) δ 166.5, 158.2, 156.3, 131.2, 130.5, 127.0, 126.4, 116.6, 109.8, 59.6, 55.2, 37.9, 24.5, 16.1, 14.3. HRMS (ESI) Calculated for $C_{15}H_{20}O_3$ $[M+H]^+$: 249.1485, found: 249.1475. IR ν (neat, cm^{-1}): 2979, 1712, 1649, 1504, 1464, 1253, 1170, 1137,1053, 1036, 807.

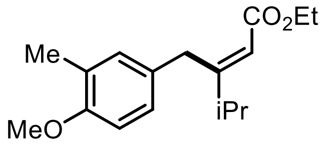

ethyl (E)-3-(4-methoxy-3-methylbenzyl)-4-methylpent-2-enoate (**3cc**). The reaction was carried out according to the general procedure A on 0.2 mmol scale (24 h); purified by flash column chromatography on SiO_2 (eluent: petroleum ether/ethyl acetate) to afford **3cc**, 43.6 mg, 79%, light yellow oil; Rf = 0.9 (petroleum ether/ethyl acetate 10:1). ^{1}H NMR (400 MHz, Chloroform-*d*) δ 7.00 - 6.97 (m, 2H), 6.71 (d, *J* = 8.2 Hz, 1H), 5.79 (s, 1H), 4.18 (q, *J* = 7.2 Hz, 2H), 3.98 (s, 2H), 3.79 (s, 3H), 2.36 – 2.30 (m, 1H), 2.18 (s, 3H), 1.29 (t, *J* = 7.1 Hz, 3H), 1.01 (d, *J* = 6.8 Hz, 6H). ^{13}C NMR (101 MHz, Chloroform-*d*) δ 167.6, 167.2, 156.1, 131.2, 130.6, 126.8, 126.3, 114.1, 109.8, 59.7, 55.3, 35.9, 34.1, 21.8, 16.2, 14.3. HRMS (ESI) Calculated for $C_{17}H_{24}O_3$ $[M+H]^+$: 277.1798, found: 277.1790. IR ν (neat, cm^{-1}): 2963, 1713, 1641, 1504, 1253, 1164, 1134, 1035.

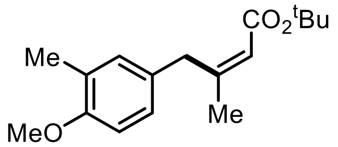

tert-butyl (Z)-4-(4-methoxy-3-methylphenyl)-3-methylbut-2-enoate (**3dd**). The reaction was carried out according to the general procedure A on 0.2 mmol scale (24 h); purified by flash column chromatography on SiO_2 (eluent: petroleum ether/ethyl acetate) to afford **3dd**, 37.0 mg, 67%, light yellow oil; Rf = 0.9 (petroleum ether/ethyl acetate 10:1). ^{1}H NMR (400 MHz, Chloroform-*d*) δ 7.02 – 6.98 (m, 2H), 6.72 (d, *J* = 8.2 Hz, 1H), 5.67 (s, 1H), 3.87 (s, 2H), 3.79 (s, 3H), 2.18 (s, 3H), 1.74 (d, *J* = 1.5 Hz, 3H), 1.50 (s, 9H). ^{13}C NMR (101 MHz, Chloroform-*d*) δ 166.1, 156.2, 156.1, 131.2, 130.6, 127.0, 126.4, 118.5, 109.8, 79.7, 55.3, 37.7, 28.2, 24.3, 16.2. HRMS (ESI) Calculated for $C_{17}H_{24}O_3$ $[M+H]^+$: 277.1798, found: 277.1789. IR ν (neat, cm^{-1}): 2976, 1706, 1647, 1504, 1366, 1251, 1152, 1134, 1035, 863.

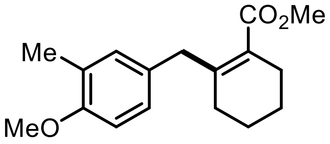

methyl 2-(4-methoxy-3-methylbenzyl)cyclohex-1-ene-1-carboxylate (**3ee**). The reaction was carried out according to the general procedure A on 0.2 mmol scale (24 h); purified by flash column chromatography on SiO_2 (eluent: petroleum ether/ethyl acetate) to afford **3ee**, 38.9 mg, 71%, light yellow oil; Rf = 0.9 (petroleum ether/ethyl acetate 10:1). ^{1}H NMR (400 MHz, Chloroform-*d*) δ 7.00 – 6.92 (m, 2H), 6.72 (d, *J* = 7.9 Hz, 1H), 3.79 (s, 3H), 3.74 (s, 3H), 3.62 (s, 2H), 2.36 – 2.32 (m, 2H), 2.19 (s, 3H), 2.03 – 2.00 (m, 2H), 1.63 – 1.50 (m, 4H). ^{13}C NMR (101 MHz, Chloroform-*d*) δ 169.8, 156.1, 146.8, 131.4, 131.2, 126.8, 126.3, 125.4, 109.7, 55.3, 51.3, 39.8, 30.2, 26.7, 22.2, 22.1, 16.2. HRMS (ESI) Calculated for $C_{17}H_{22}O_3$ $[M+H]^+$: 275.1642, found: 275.1632. IR ν (neat, cm^{-1}): 2930, 1711, 1503, 1433, 1277, 1251, 1134, 1036. 1079, 1025, 833.

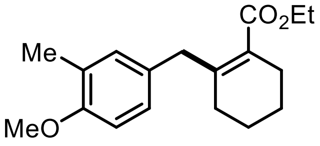

ethyl 2-(4-methoxy-3-methylbenzyl)cyclohex-1-ene-1-carboxylate (**3ff**). The reaction was carried out according to the general procedure A on 0.2 mmol scale (24 h); purified by flash column chromatography on SiO_2 (eluent: petroleum ether/ethyl acetate) to afford **3ff**, 40.3 mg, 70%, light yellow oil; Rf = 0.9 (petroleum ether/ethyl acetate 10:1). ^{1}H NMR (400 MHz, Chloroform-*d*) δ 7.01 – 6.98 (m, 2H), 6.73 – 6.71 (m, 1H), 4.2 (q, *J* = 7.1 Hz, 2H), 3.79 (s, 3H), 3.60 (s, 2H), 2.36 – 2.32 (m, 2H), 2.19 (s, 3H), 2.02 – 1.99 (m, 2H), 1.63 – 1.50 (m, 4H), 1.29 (t, *J* = 7.1 Hz, 3H). ^{13}C NMR (101 MHz, Chloroform-*d*) δ 169.6, 156.1, 145.8, 131.4, 131.2, 126.8, 126.2, 125.8, 109.8, 60.1, 55.29, 39.8, 30.1, 26.8, 22.3, 22.2, 16.2, 14.3. HRMS (ESI) Calculated for $C_{17}H_{22}O_3$ $[M+H]^+$: 275.1642, found: 275.1632. IR ν (neat, cm^{-1}): 2930, 1709, 1504, 1252, 1224, 1134, 1046.

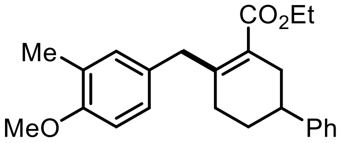

ethyl 4-(4-methoxy-3-methylbenzyl)-1,2,5,6-tetrahydro-[1,1'-biphenyl]-3-carboxylate (**3gg**). The reaction was carried out according to the general procedure A on 0.2 mmol scale (24 h); purified by flash column chromatography on SiO_2 (eluent: petroleum ether/ethyl acetate) to afford **3gg**, 57.5 mg, 79%, light yellow oil; Rf = 0.9 (petroleum ether/ethyl acetate 10:1). ^{1}H NMR (400 MHz, Chloroform-*d*) δ 7.31 – 7.24 (m, 2H), 7.23 – 7.17 (m, 3H), 7.05 – 7.02 (m, 2H), 6.74 (d, *J* = 8.8 Hz, 1H), 4.21 (q, *J* = 7.1 Hz, 2H), 3.80 (s, 3H), 3.74 – 3.62 (m, 2H), 2.79 – 2.68 (m, 2H), 2.45 – 2.36 (m, 1H), 2.21 – 2.18 (s, 4H), 1.89 – 1.86 (m, 1H), 1.74 – 1.65 (m, 1H), 1.28 (t, *J* = 7.2 Hz, 3H). ^{13}C NMR (101 MHz, Chloroform-*d*) δ 169.0, 156.2, 146.1, 146.1, 131.3, 131.2, 128.4, 126.9, 126.8, 126.3, 126.2, 125.4, 109.8, 60.2, 55.3, 39.8, 39.4, 34.8, 30.9, 29.1, 16.2, 14.3. HRMS (ESI) Calculated for $C_{24}H_{28}O_3$ $[M+H]^+$: 365.2111, found: 365.2098. IR ν (neat, cm^{-1}): 2926, 1708, 1503, 1464, 1251, 1235, 1208, 1132, 1036, 755, 701.

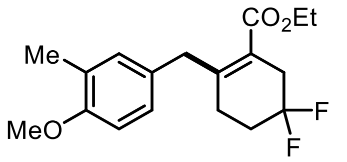

ethyl 5,5-difluoro-2-(4-methoxy-3-methylbenzyl)cyclohex-1-ene-1-carboxylate (**3hh**). ^{1}H NM The reaction was carried out according to the general procedure A on 0.2 mmol scale (24 h); purified by flash column chromatography on SiO_2 (eluent: petroleum ether/ethyl acetate) to afford **3hh**, 49.9 mg, 77%, light yellow oil; Rf = 0.8 (petroleum ether/ethyl acetate 10:1). R (400 MHz, Chloroform-*d*) δ 6.99 – 6.97 (m, 2H), 6.73 (d, *J* = 8.9 Hz, 1H), 4.23 (q, *J* = 7.1 Hz, 2H), 3.80 (s, 3H), 3.76 (s, 2H), 2.87 (t, *J* = 14.9 Hz, 2H), 2.33 (t, *J* = 6.8 Hz, 2H), 2.19 (s, 3H), 1.99 – 1.88 (m, 2H), 1.30 (t, *J* = 7.1 Hz, 3H). ^{13}C NMR (101 MHz, Chloroform-*d*) δ 167.0, 156.4, 147.4, 131.1, 130.4, 126.8, 126.6, 122.4, 121.0 (t, *J* = 5.7 Hz), 109.9, 60.6, 55.3, 38.7, 35.7 (t, *J* = 27.9 Hz), 29.8 (t, *J* = 24.3 Hz), 28.8 (t, *J* = 5.4 Hz), 16.2, 14.2. ^{19}F NMR (376 MHz, Chloroform-*d*) δ -96.67. HRMS (ESI) Calculated for $C_{18}H_{22}F_2O_3$ $[M+H]^+$: 325.1610, found: 325.1601. IR ν (neat, cm^{-1}): 2928, 1715, 1504, 1374, 1253, 1235, 1115, 1077, 960.

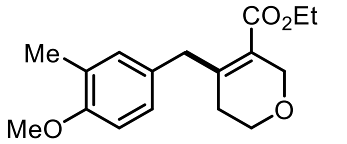

methyl 4-(4-methoxy-3-methylbenzyl)-5,6-dihydro-2H-pyran-3-carboxylate (**3ii**). The reaction was carried out according to the general procedure A on 0.2 mmol scale (24 h); purified by flash column chromatography on SiO_2 (eluent: petroleum ether/ethyl acetate) to afford **3ii**, 40.3 mg, 73%, light yellow oil; Rf = 0.5 (petroleum ether/ethyl acetate 10:1). ^{1}H NMR (400 MHz, Chloroform-*d*) δ 7.01 – 6.96 (m, 2H), 6.75 – 6.72 (m, 1H), 4.35 (s, 2H), 3.85 (s, 2H), 3.80 (s, 3H), 3.76 (s, 3H), 3.68 (t, *J* = 5.6 Hz, 2H), 2.19 – 2.13 (m, 5H). ^{13}C NMR (101 MHz, Chloroform-*d*) δ 166.3, 156.3, 148.6, 131.2, 130.3, 127.0, 126.5, 123.7, 109.9, 65.6, 63.9, 55.3, 51.3, 38.9, 29.9, 16.2. HRMS (ESI) Calculated for $C_{16}H_{20}O_4$ $[M+H]^+$: 277.1434, found: 277.1428. IR ν (neat, cm^{-1}): 2951, 1716, 1503, 1256, 1231, 1130, 1078, 1040.

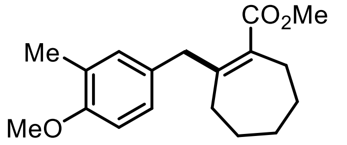

methyl 2-(4-methoxy-3-methylbenzyl)cyclohept-1-ene-1-carboxylate (**3jj**). The reaction was carried out according to the general procedure A on 0.2 mmol scale (24 h); purified by flash column chromatography on SiO_2 (eluent: petroleum ether/ethyl acetate) to afford **3jj**, 50.5 mg, 83%, light yellow oil; Rf = 0.8 (petroleum ether/ethyl acetate 10:1). *^{1}H NMR (400 MHz, Chloroform-d) δ 7.03 – 6.99 (m, 2H), 6.73 (d, J = 8.1 Hz, 1H), 3.79 (s,* 3H), 3.74 (s, 3H), 3.55 (s, 2H), 2.49 – 2.46 (m, 2H), 2.19 – 2.15 (m, 5H), 1.74 – 1.68 (m, 2H), 1.57 – 1.51 (m, 2H), 1.36 – 1.30 (m, 2H). ^{13}C NMR (101 MHz, Chloroform-*d*) δ 170.8, 156.2, 151.8, 131.5, 131.4, 130.4, 127.2, 126.2, 109.7, 55.3, 51.4, 41.1, 34.4, 32.3, 30.3, 26.5, 25.6, 16.2. HRMS (ESI) Calculated for $C_{18}H_{24}O_3$ $[M+H]^+$: 289.1798, found: 289.1789. IR ν (neat, cm^{-1}): 2923, 2851, 1711, 1504, 1441, 1288, 1200, 1134, 1033.

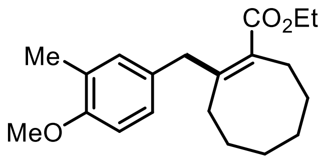

ethyl (Z)-2-(4-methoxy-3-methylbenzyl)cyclooct-1-ene-1-carboxylate (**3kk**). The reaction was carried out according to the general procedure A on 0.2 mmol scale (24 h); purified by flash column chromatography on SiO_2 (eluent: petroleum ether/ethyl acetate) to afford **3kk**, 53.7 mg, 85%, light yellow oil; Rf = 0.9 (petroleum ether/ethyl acetate 10:1). ^{1}H NMR (400 MHz, Chloroform-*d*) δ 7.05 – 7.03 (m, 2H), 6.72 (d, *J* = 8.9 Hz, 1H), 4.22 (q, *J* = 7.2 Hz, 2H), 3.79 (s, 3H), 3.58 (s, 2H), 2.45 – 2.42 (m, 2H), 2.19 – 2.14 (m, 5H), 1.71 – 1.65 (m, 2H), 1.49 – 1.43 (m, 6H), 1.29 (t, *J* = 7.1 Hz, 3H). ^{13}C NMR (101 MHz, Chloroform-*d*) δ 170.0, 156.1, 148.1, 131.5, 131.1, 128.7, 127.2, 126.2, 109.7, 60.0, 55.3, 38.9, 31.2, 29.9, 28.9, 28.6, 26.7, 26.2, 16.2, 14.3.
HRMS (ESI) Calculated for $C_{20}H_{28}O_3$ $[M+H]^+$: 317.2111, found: 317.2101. IR ν (neat, cm^{-1}): 2923, 1707, 1504, 1290, 1253, 1200, 1180, 1133, 1038.

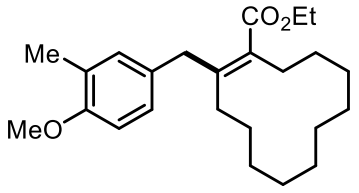

ethyl (Z)-2-(4-methoxy-3-methylbenzyl)cyclododec-1-ene-1-carboxylate (**3ll**). The reaction was carried out according to the general procedure A on 0.2 mmol scale (24 h); purified by flash column chromatography on SiO_2 (eluent: petroleum ether/ethyl acetate) to afford **3ll**, 53.7 mg, 72%, light yellow oil; Rf = 0.9 (petroleum ether/ethyl acetate 10:1). ^{1}H NMR (400 MHz, Chloroform-*d*) δ 6.97 – 6.93 (m, 2H), 6.71 (d, *J* = 7.9 Hz, 1H), 4.22 (q, *J* = 7.1 Hz, 1H), 3.79 (s, 2H), 3.48 (s, 2H), 2.40 (t, *J* = 7.3 Hz, 2H), 2.18 (s, 3H), 2.03 (t, *J* = 7.4 Hz, 2H), 1.55 – 1.33 (m, 16H), 1.29 (t, *J* = 7.2 Hz, 3H). ^{13}C NMR (101 MHz, Chloroform-*d*) δ 171.1, 156.1, 142.8, 131.4, 131.1, 131.1, 126.8, 126.3, 109.7, 60.2, 55.3, 37.8, 27.3, 27.1, 26.1, 25.6, 25.4, 25.0, 24.8, 24.8, 22.5, 16.2, 14.3. HRMS (ESI) Calculated for $C_{24}H_{36}O_3$ $[M+H]^+$: 373.2737, found: 373.2728. IR ν (neat, cm^{-1}): 2927, 2859, 1716, 1504, 1467, 1252, 1212, 1134, 1036.

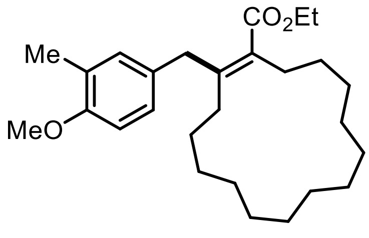

ethyl (Z)-2-(4-methoxy-3-methylbenzyl)cyclopentadec-1-ene-1-carboxylate (**3mm**). The reaction was carried out according to the general procedure A on 0.2 mmol scale (24 h); purified by flash column chromatography on SiO_2 (eluent: petroleum ether/ethyl acetate) to afford **3mm**, 69.7 mg, 84%, light yellow oil; Rf = 0.8 (petroleum ether/ethyl acetate 10:1). ^{1}H NMR (400 MHz, Chloroform-*d*) δ 6.98 (d, *J* = 6.8 Hz, 2H), 6.71 (d, *J* = 9.0 Hz, 1H), 4.20 (q, *J* = 7.2 Hz, 2H), 3.78 (s, 3H), 3.50 (s, 2H), 2.36 – 2.27 (m, 2H), 2.18 (s, 3H), 2.01 – 1.91 (m, 2H), 1.53 – 1.25 (m, 25H). ^{13}C NMR (101 MHz, Chloroform-*d*) δ 170.7, 156.1, 144.6, 131.3, 131.2, 130.3, 126.9, 126.2, 109.7, 60.1, 55.2, 38.7, 31.3, 29.9, 27.6, 27.4, 26.8, 26.8, 26.7, 26.6, 26.1, 26.1,

25.4, 25.4, 16.2, 14.2. HRMS (ESI) Calculated for $C_{27}H_{42}O_3$ $[M+H]^+$: 415.3208, found: 415.3198.
IR ν (neat, cm^{-1}): 2927, 2857, 1713, 1504, 1462, 1253, 1222, 1134, 1036.

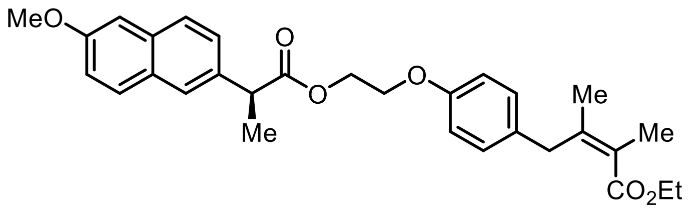

*ethyl (S,Z)-4-(4-(2-((2-(6-methoxynaphthalen-2-yl)propanoyl)oxy)ethoxy)phenyl)-2,3-*dimethylbut-2-enoate (**4a**). The reaction was carried out according to the general procedure A on 0.2 mmol scale (24 h); purified by flash column chromatography on SiO_2 (eluent: petroleum ether/ethyl acetate) to afford **4a**, 78.6 mg, 80%, light yellow oil; Rf = 0.3 (petroleum ether/ethyl acetate 10:1). ^{1}H NMR (400 MHz, Chloroform-*d*) δ 7.67 – 7.64 (m, 3H), 7.39 (dd, *J* = 8.6, 1.8 Hz, 1H), 7.13 – 7.08 (m, 4H), 6.74 (d, *J* = 8.7 Hz, 2H), 4.46 – 4.32 (m, 2H), 4.21 (q, *J* = 7.2 Hz, 2H), 4.07 (t, *J* = 4.8 Hz, 2H), 3.88 (s, 3H), 3.61 (s, 2H), 1.90 (s, 3H), 1.65 (s, 3H), 1.57 (d, *J* = 7.2 Hz, 3H), 1.28 (t, *J* = 7.2 Hz, 3H). ^{13}C NMR (101 MHz, Chloroform-*d*) δ 174.5, 169.9, 157.6, 156.8, 143.2, 135.4, 133.6, 132.1, 129.8, 129.2, 128.8, 127.1, 126.1, 125.9, 124.1, 118.9, 114.5, 105.5, 65.8, 63.1, 60.2, 55.2, 45.2, 40.7, 19.2, 18.5, 15.9, 14.2. HRMS (ESI) Calculated for $C_{30}H_{34}O_6$ $[M+H]^+$: 491.2428, found: 491.2412. IR ν (neat, cm^{-1}): 2978, 2936, 1734, 1509, 1270, 1174, 1077, 925.

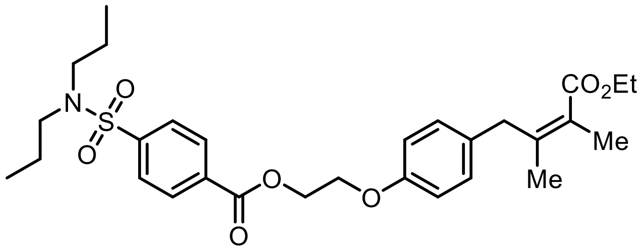

(Z)-2-(4-(4-ethoxy-2,3-dimethyl-4-oxobut-2-en-1-yl)phenoxy)ethyl 4-(N,N-dipropylsulfamoyl)benzoate (**4b**). The reaction was carried out according to the general procedure A on 0.2 mmol scale (24 h); purified by flash column chromatography on SiO_2 (eluent: petroleum ether/ethyl acetate) to afford **4b**, 81.9 mg, 75%, light yellow oil; Rf = 0.2 (petroleum ether/ethyl acetate 10:1). ^{1}H NMR (400 MHz, Chloroform-*d*) δ 8.18 – 8.16 (m, 2H), 7.88 – 7.86 (m, 2H), 7.18 – 7.16 (m, 2H), 6.87 – 6.85 (m, 2H), 4.70 – 4.67 (m, 2H), 4.31 – 4.29 (m, 2H), 4.22 (q, *J* = 7.2 Hz, 2H), 3.63 (s, 2H), 3.12 – 3.08 (m, 4H), 1.91 (s, 3H), 1.67 (s, 3H), 1.59 – 1.50 (m, 4H), 1.29 (t, *J* = 7.1 Hz, 3H), 0.87 (t, *J* = 7.4 Hz, 6H). ^{13}C NMR (101 MHz, Chloroform-*d*) δ 169.9, 165.1, 156.8, 144.3, 143.1, 133.1, 132.4, 130.3, 129.9, 126.9, 124.2, 114.5, 65.8, 64.0, 60.2, 49.8, 40.7, 21.8, 19.2, 15.9, 14.2, 11.1. HRMS (ESI) Calculated for $C_{29}H_{39}NO_7S$ $[M+H]^+$: 546.2520, found: 546.2510. IR ν (neat, cm^{-1}): 2967, 1722, 1509, 1343, 1240, 1174, 1087, 993, 778, 694.

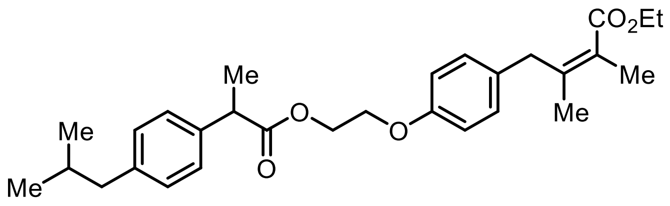

ethyl (Z)-4-(4-(2-((2-(4-isobutylphenyl)propanoyl)oxy)ethoxy)phenyl)-2,3-dimethylbut-2-enoate (**4c**). The reaction was carried out according to the general procedure A on 0.2 mmol scale (24 h); purified by flash column chromatography on SiO_2 (eluent: petroleum ether/ethyl acetate) to afford **4c**, 79.4 mg, 85%, light yellow oil; Rf = 0.5 (petroleum ether/ethyl acetate 10:1). ^{1}H NMR (400 MHz, Chloroform-*d*) δ 7.20 (d, *J* = 8.2 Hz, 2H), 7.13 (d, *J* = 8.7 Hz, 2H), 7.06 (d, *J* = 8.2 Hz, 2H), 6.77 (d, *J* = 8.7 Hz, 2H), 4.46 – 4.33 (m, 2H), 4.22 (q, *J* = 7.2 Hz, 2H), 4.09 (t, *J* = 4.8 Hz, 2H), 3.73 (q, *J* = 7.1 Hz, 1H), 3.62 (s, 2H), 2.43 (d, *J* = 7.1 Hz, 2H), 1.91 (s, 3H), 1.82 (dq, *J* = 13.6, 6.8 Hz, 1H), 1.66 (s, 3H), 1.49 (d, *J* = 7.1 Hz, 3H), 1.29 (t, *J* = 7.1 Hz, 3H), 0.88 (d, *J* = 6.6 Hz, 6H). ^{13}C NMR (101 MHz, Chloroform-*d*) δ 174.7, 169.9, 156.9, 143.2, 140.5, 137.5, 132.1, 129.8, 129.3, 127.1, 124.2, 114.5, 65.9, 63.0, 60.2, 45.0, 45.0, 40.7, 30.1, 22.3, 19.2, 18.5, 15.9, 14.2. HRMS (ESI) Calculated for $C_{29}H_{38}O_5$ $[M+H]^+$: 467.2792, found: 467.2792. IR ν (neat, cm^{-1}): 2955, 2930, 1736, 1710, 1510, 1299, 1244, 1166, 1102, 1077.

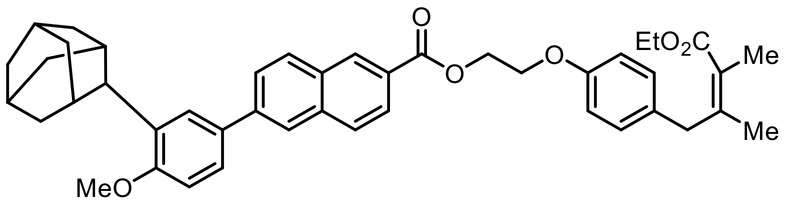

(Z)-2-(4-(4-ethoxy-2,3-dimethyl-4-oxobut-2-en-1-yl)phenoxy)ethyl 6-(3-(adamantan-2-yl)-4-methoxyphenyl)-2-naphthoate (**4d**). The reaction was carried out according to the general procedure A on 0.2 mmol scale (24 h); purified by flash column chromatography on SiO_2 (eluent: petroleum ether/ethyl acetate) to afford **4d**, 29.6 mg, 22%, light yellow oil; Rf = 0.4 (petroleum ether/ethyl acetate 10:1). ^{1}H NMR (400 MHz, Chloroform-*d*) δ 8.61 (s, 1H), 8.07 (dd, *J* = 8.6, 1.8 Hz, 1H), 8.00 – 7.97 (m, 2H), 7.90 (d, *J* = 8.7 Hz, 1H), 7.79 (dd, *J* = 8.6, 1.8 Hz, 1H), 7.60 (d, *J* = 2.3 Hz, 1H), 7.54 (dd, *J* = 8.4, 2.4 Hz, 1H), 7.18 (d, *J* = 8.7 Hz, 2H), 6.99 (d, *J* = 8.4 Hz, 1H), 6.92 – 6.88 (m, 2H), 4.72 (dd, *J* = 5.5, 4.2 Hz, 2H), 4.34 (dd, *J* = 5.4, 4.2 Hz, 2H), 4.22 (q, *J* = 7.2 Hz, 2H), 3.90 (s, 3H), 3.63 (s, 2H), 2.18 (d, *J* = 3.3 Hz, 6H), 2.10 (s, 3H), 1.91 (d, *J* = 1.0 Hz, 3H), 1.80 (s, 6H), 1.66 (d, *J* = 1.1 Hz, 3H), 1.31 – 1.25 (m, 6H). ^{13}C NMR (101 MHz, Chloroform-*d*) δ 170.0, 166.8, 158.9, 157.0, 143.2, 141.4, 139.0, 136.0, 132.5, 132.3, 131.2, 131.0, 130.0, 129.7, 128.2, 126.6, 126.5, 126.0, 125.7, 125.6, 124.7, 124.2, 114.6, 112.1, 66.1, 63.5, 60.3, 55.1, 40.8, 40.6, 37.1, 29.1, 19.3, 16.0, 14.3. HRMS (ESI) Calculated for $C_{44}H_{48}O_6$ $[M+H]^+$: 673.3524, found: 673.3510. IR ν (neat, cm^{-1}): 2903, 1715, 1629, 1509, 1278, 1237, 1216, 1139, 1076, 809.

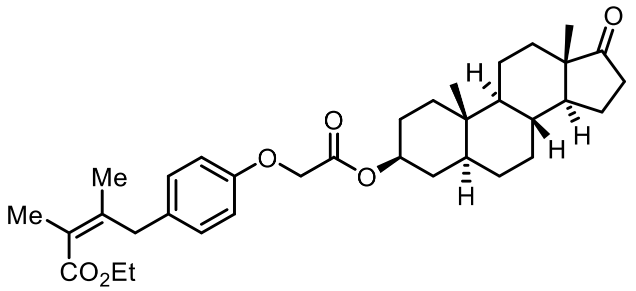

*ethyl (Z)-4-(4-(2-(((3S,5S,8R,9S,10S,13S,14S)-10,13-dimethyl-17-oxohexadecahydro-1H-*cyclopenta[a]phenanthren-3-yl)oxy)-2-oxoethoxy)phenyl)-2,3-dimethylbut-2-enoate (**4e**). The reaction was carried out according to the general procedure A on 0.2 mmol scale (24 h); purified by flash column chromatography on SiO_2 (eluent: petroleum ether/ethyl acetate) to afford **4e**, 87.0 mg, 77%, light yellow oil; Rf = 0.2 (petroleum ether/ethyl acetate 10:1). ^{1}H NMR (400 MHz, Chloroform-*d*) δ 7.15 (d, *J* = 8.7 Hz, 2H), 6.81 (d, *J* = 8.7 Hz, 2H), 4.83 (ddd, *J* = 11.5, 6.5, 4.9 Hz, 1H), 4.55 (s, 2H), 4.21 (q, *J* = 7.1 Hz, 2H), 3.62 (s, 2H), 2.43 (dd, *J* = 19.3, 8.1 Hz, 1H), 2.16 – 2.02 (m, 1H), 1.97 – 1.60 (m, 1H), 1.60 – 1.15 (m, 13H), 1.12 – 0.93 (m, 2H), 0.85 (d, *J* = 3.4 Hz, 6H), 0.77 – 0.66 (m, 1H). ^{13}C NMR (101 MHz, Chloroform-*d*) δ 221.1, 169.9, 168.5, 156.3, 143.1, 132.7, 129.8, 124.2, 114.5, 74.6, 65.7, 60.2, 54.2, 51.3, 47.7, 44.5, 40.7, 36.5, 35.7, 35.5, 34.9, 33.7, 31.4, 30.7, 28.2, 27.2, 21.7, 20.4, 19.2, 15.9, 14.2, 13.7, 12.1. HRMS (ESI) Calculated for $C_{35}H_{48}O_6$ $[M+H]^+$: 565.3524, found: 565.3516. IR ν (neat, cm^{-1}): 2931, 2855, 1737, 1708, 1509, 1444, 1273, 1233, 1195, 1176, 1103, 1080, 1013.

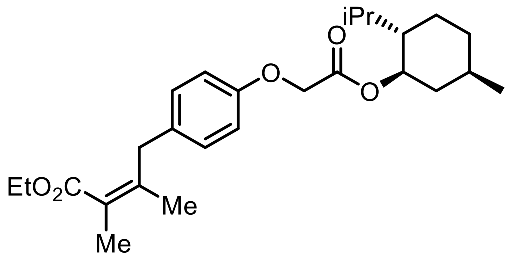

ethyl (Z)-4-(4-(2-(((1R,2S,5R)-2-isopropyl-5-methylcyclohexyl)oxy)-2-oxoethoxy)phenyl)-2,3-dimethylbut-2-enoate (**4f**). The reaction was carried out according to the general procedure A on 0.2 mmol scale (24 h); purified by flash column chromatography on SiO_2 (eluent: petroleum ether/ethyl acetate) to afford **4f**, 49.1 mg, 57%, light yellow oil; Rf = 0.5 (petroleum ether/ethyl acetate 10:1). ^{1}H NMR (400 MHz, Chloroform-*d*) δ 7.14 (d, *J* = 8.7 Hz, 2H), 6.81 (d, *J* = 8.8 Hz, 2H), 4.80 (td, *J* = 10.9, 4.4 Hz, 1H), 4.57 (d, *J* = 1.6 Hz, 2H), 4.21 (q, *J* = 7.1 Hz, 1H), 3.62 (s, 2H), 2.06 – 1.99 (m, 1H), 1.90 (d, *J* = 1.0 Hz, 3H), 1.77 – 1.65 (m, 6H), 1.54 – 1.27 (m, 6H), 1.10 – 1.01 (m, 1H), 0.91 – 0.84 (m, 7H), 0.72 (d, *J* = 6.8 Hz, 3H). ^{13}C NMR (101 MHz, Chloroform-*d*) δ 169.9, 168.1, 156.3, 143.1, 132.7, 129.9, 124.2, 114.4, 75.4, 65.6, 60.2, 46.9, 40.7, 40.7, 34.1, 31.3, 26.1, 23.3, 21.9, 20.7, 19.2, 16.1, 15.9, 14.2. HRMS (ESI) Calculated for $C_{26}H_{38}O_5$ $[M+H]^+$: 431.2792, found: 431.2791. IR ν (neat, cm^{-1}): 2955, 2927, 1757, 1711, 1509, 1274, 1196, 1176, 1103, 1081.

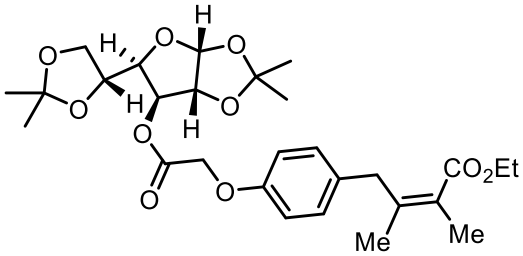

ethyl (Z)-4-(4-(2-(((3aR,5R,6S,6aR)-5-((R)-2,2-dimethyl-1,3-dioxolan-4-yl)-2,2-dimethyltetrahydrofuro[2,3-d][1,3]dioxol-6-yl)oxy)-2-oxoethoxy)phenyl)-2,3-dimethylbut-2-enoate (**4g**). The reaction was carried out according to the general procedure A on 0.2 mmol scale (24 h); purified by flash column chromatography on SiO_2 (eluent: petroleum ether/ethyl acetate) to afford **4g**, 47.1 mg, 44%, light yellow oil; Rf = 0.2 (petroleum ether/ethyl acetate 10:1). ^{1}H NMR (400 MHz, Chloroform-*d*) δ 7.16 (d, *J* = 8.7 Hz, 2H), 6.82 (d, *J* = 8.8 Hz, 2H), 5.79 (d, *J* = 3.7 Hz, 1H), 5.39 (d, *J* = 3.1 Hz, 1H), 4.64 (s, 2H), 4.46 (s, 1H), 4.24 – 4.18 (m, 3H), 4.15 – 4.11 (m, 1H), 4.07 – 3.98 (m, 2H), 3.62 (s, 2H), 1.90 (s, 3H), 1.65 (s, 3H), 1.51 (s, 3H), 1.40 (s, 3H), 1.31 – 1.27 (m, 9H). ^{13}C NMR (101 MHz, Chloroform-*d*) δ 169.9, 167.9, 156.1, 143.0, 133.1, 130.0, 124.4, 114.5, 112.4, 109.4, 105.1, 83.2, 79.8, 72.4, 67.2, 65.4, 60.3, 40.7, 26.9, 26.7, 26.2, 25.2, 19.3, 15.9, 14.3. HRMS (ESI) Calculated for $C_{28}H_{38}O_{10}$ $[M+H]^+$: 525.2538, found: 525.2533. IR ν (neat, cm^{-1}): 2987, 1772, 1708, 1509, 1374, 1215, 1164, 1076, 844.

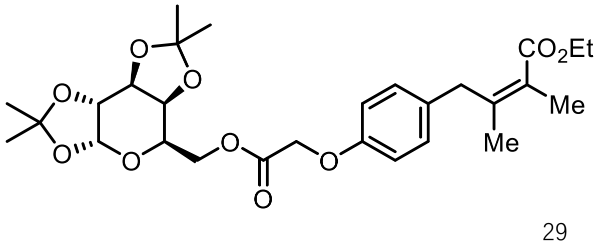

ethyl (Z)-2,3-dimethyl-4-(4-(2-oxo-2-(((3aR,5R,5aS,8aS,8bR)-2,2,7,7-tetramethyltetrahydro-5H-bis([1,3]dioxolo)[4,5-b:4',5'-d]pyran-5-yl)methoxy)ethoxy)phenyl)but-2-enoate (**4h**). The reaction was carried out according to the general procedure A on 0.2 mmol scale (24 h); purified by flash column chromatography on SiO_2 (eluent: petroleum ether/ethyl acetate) to afford **4h**, 52.4 mg, 49%, light yellow oil; Rf = 0.2 (petroleum ether/ethyl acetate 10:1). ^{1}H NMR (400 MHz, Chloroform-*d*) δ 7.14 (d, *J* = 8.7 Hz, 2H), 6.82 (d, *J* = 8.8 Hz, 2H), 5.54 (d, *J* = 5.0 Hz, 1H), 4.64 (s, 2H), 4.61 (dd, *J* = 7.8, 2.6 Hz, 1H), 4.42 (dd, *J* = 11.6, 4.6 Hz, 1H), 4.37 – 4.30 (m, 2H), 4.20 (dd, *J* = 7.9, 6.3 Hz, 3H), 4.09 – 4.02 (m, 1H), 3.62 (s, 2H), 1.90 (s, 3H), 1.66 (s, 3H), 1.49 (s, 3H), 1.45 (s, 3H), 1.33 (s, 6H), 1.29 (t, *J* = 7.2 Hz, 3H). ^{13}C NMR (101 MHz, Chloroform-*d*) δ 169.9, 168.9, 156.3, 143.1, 132.7, 129.9, 124.2, 114.6, 109.7, 108.8, 96.2, 70.9, 70.6, 70.3, 65.8, 65.4, 64.0, 60.2, 40.7, 26.0, 25.9, 24.9, 24.4, 19.3, 15.9, 14.2. HRMS (ESI) Calculated for $C_{28}H_{38}O_{10}$ $[M+H]^+$: 535.2538, found: 535.2530. IR ν (neat, cm^{-1}): 2985, 1762, 1708, 1509, 1381, 1167, 1004, 859, 511.

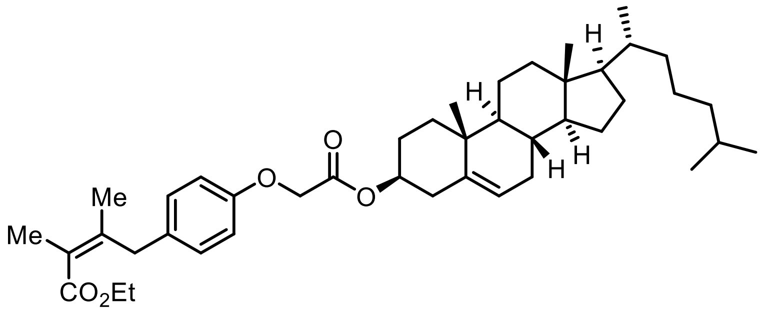

ethyl (Z)-4-(4-(2-(((3S,8S,9S,10R,13R,14S,17R)-10,13-dimethyl-17-((R)-6-methylheptan-2-yl)-2,3,4,7,8,9,10,11,12,13,14,15,16,17-tetradecahydro-1H-cyclopenta[a]phenanthren-3-yl)oxy)-2-oxoethoxy)phenyl)-2,3-dimethylbut-2-enoate (**4i**). The reaction was carried out according to the general procedure A on 0.2 mmol scale (24 h); purified by flash column chromatography on SiO_2 (eluent: petroleum ether/ethyl acetate) to afford **4i**, 39.7 mg, 30%, light yellow oil; Rf = 0.8 (petroleum ether/ethyl acetate 10:1). ^{1}H NMR (400 MHz, Chloroform-*d*) δ 7.15 (d, *J* = 8.7 Hz, 2H), 6.82 (d, *J* = 8.7 Hz, 2H), 5.38 (dd, *J* = 5.0, 1.7 Hz, 1H), 4.78 – 4.70 (m, 1H), 4.56 (s, 2H), 4.21 (q, *J* = 7.2 Hz, 2H), 3.62 (s, 2H), 2.34 (d, *J* = 8.2 Hz, 2H), 2.04 – 1.79 (m, 8H), 1.65 – 1.48 (m, 10H), 1.39 – 1.24 (m, 8H), 1.19 – 1.07 (m, 6H), 1.06 – 0.97 (m, 6H), 0.45 – 0.85 (m, 9H), 0.68 (s, 3H). ^{13}C NMR (101 MHz, Chloroform-*d*) δ 169.9, 168.5, 156.3, 143.1, 139.2, 132.7, 129.9, 124.2, 122.9, 114.5, 75.1, 65.7, 60.2, 56.6, 56.1, 50.0, 42.3, 40.7, 39.7, 39.5, 37.9, 36.9, 36.5, 36.1, 35.7, 31.8, 31.8, 28.2, 28.0, 27.6, 24.2, 23.8, 22.8, 22.5, 21.0, 19.2, 18.7, 15.92 14.2, 11.8. HRMS (ESI) Calculated for $C_{43}H_{64}O_5$ $[M+H]^+$: 661.4827, found: 661.4822. IR ν (neat, cm^{-1}): 2936, 2868, 1760, 1711, 1509, 1274, 1195, 1176, 1103, 1081.

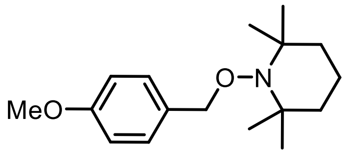

1-((4-methoxybenzyl)oxy)-2,2,6,6-tetramethylpiperidine (**11**). The reaction was carried out according to the general procedure A (extra 3 equiv. TEMPO was added) on 0.2 mmol scale (24 h); purified by flash column chromatography on SiO_2 (eluent: petroleum ether/ethyl acetate) to afford **11**, 2.2 mg, 4%, light yellow oil; Rf = 0.8 (petroleum ether/ethyl acetate 10:1).

^{1}H NMR (400 MHz, Chloroform-*d*) δ 7.31 – 7.27 (m, 2H), 6.89 – 6.86 (m, 2H), 4.74 (s, 2H), 3.80 (s, 3H), 1.61 – 1.48 (m, 6H), 1.26 (s, 6H), 1.13 (s, 6H).

^{13}C NMR (101 MHz, Chloroform-*d*) δ 159.0, 130.4, 129.2, 113.7, 78.4, 60.0, 55.3, 39.7, 33.2, 20.3, 17.2.

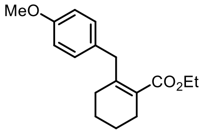

ethyl 2-(4-methoxybenzyl)cyclohex-1-ene-1-carboxylate (**13**). The reaction was carried out according to the mordified procedure on 0.2 mmol scale. Modified conditions: **PC** [Ir(dF(CF$_3$)ppy)$_2$(5,5'-dFbpy)]PF$_6$ (2 mol%), NiCl$_2$•6H$_2$O (10 mol%), 4,4'-Di-tert-butyl-2,2'-bipyridine (15%), **1** (0.4 mmol), **2a** (0.2 mmol), K_2HPO_4 (0.4 mmol), DMF (2 mL), blue LEDs, 24 h; purified by flash column chromatography on SiO_2 (eluent: petroleum ether/ethyl acetate) to afford **13**, 32.9 mg, 60%, light yellow oil; Rf = 0.8 (petroleum ether/ethyl acetate 10:1). ^{1}H NMR (400 MHz, Chloroform-*d*) δ 7.14 (d, J = 8.6 Hz, 2H), 6.81 (d, J = 8.6 Hz, 2H), 4.21 (q, J = 7.1 Hz, 2H), 3.78 (s, 3H), 3.63 (s, 2H), 2.36 – 2.32 (m, 2H), 2.02 – 1.98 (m, 2H), 1.64 – 1.50 (m, 4H), 1.28 (t, J = 7.1 Hz, 3H). ^{13}C NMR (101 MHz, Chloroform-d) δ 169.5, 157.9, 145.7, 131.9, 129.7, 126.0, 113.6, 60.1, 55.2, 39.8, 30.1, 26.7, 22.2, 22.2, 14.3. HRMS (ESI) Calculated for $C_{17}H_{22}O_3$ [M+H]$^+$: 275.1642, found: 275.1634. IR ν (neat, cm^{-1}): 2931, 2856, 1415, 1246, 1207, 1143, 1052, 883, 832, 612.

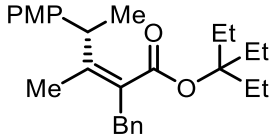

3-ethylpentan-3-yl (S,Z)-2-benzyl-4-(4-methoxyphenyl)-3-methylpent-2-enoate (**5a**). The reaction was carried out according to the general procedure B on 0.1 mmol scale (36 h); purified by flash column chromatography on SiO_2 (eluent: petroleum ether/ethyl acetate) to afford **5a**, 26.6mg, 65%, light yellow oil; Rf = 0.8 (petroleum ether/ethyl acetate 10:1). ^{1}H NMR (400 MHz, Chloroform-*d*) δ 7.26 – 7.13 (m, 7H), 6.85 (d, J = 8.8 Hz, 2H), 4.63 (q, J = 7.1 Hz, 1H), 3.79 (s, 3H), 3.68 (d, J = 8.9 Hz, 2H), 1.78 (q, J = 7.6 Hz, 6H), 1.45 – 1.43 (m, 6H), 0.72 (t, J = 7.5 Hz, 9H). ^{13}C NMR (101 MHz, Chloroform-*d*) δ 168.9, 157.9, 147.2, 139.6, 135.6, 128.5, 128.2, 128.1, 125.8, 113.5, 89.0, 55.2, 40.6, 36.1, 26.9, 16.9, 14.2, 7.6. HRMS (ESI) Calculated for $C_{27}H_{36}O_3$ [M+K]$^+$: 447.2296, found: 447.2286. IR ν (neat, cm^{-1}): 2969, 2940, 1703, 1511, 1455, 1283, 1248, 1216, 1179, 1136, 1103, 1039, 833. HPLC analysis CHIRALCEL® two AD-H columns, n-hexane/iso-propanol = 98.5/1.5, flow rate 0.5 mL/min, 254 nm UV detector, t_R (minor) = 23.820 min, t_R (major) = 25.226 min. $[\alpha]_D^{25}$ = -7 (c = 0.6, CH_2Cl_2); 91% ee

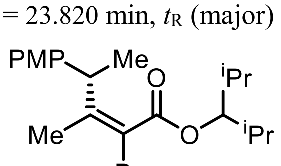

2,4-dimethylpentan-3-yl (S,Z)-2-benzyl-4-(4-methoxyphenyl)-3-methylpent-2-enoate (**5b**). The reaction was carried out according to the general procedure B on 0.1 mmol scale (36 h); purified by flash column chromatography on SiO_2 (eluent: petroleum ether/ethyl acetate) to afford **5b**, 28.6 mg, 70%, light yellow oil; Rf = 0.8 (petroleum ether/ethyl acetate 10:1). ^{1}H NMR (400

MHz, Chloroform-*d*) δ 7.27 – 7.23 (m, 4H), 7.19 – 7.12 (m, 3H), 6.85 (d, *J* = 8.8 Hz, 2H), 4.76 – 4.66 (m, 2H), 3.79 (s, 3H), 3.74 (d, *J* = 8.3 Hz, 2H), 1.88 – 1.79 (m, 2H), 1.50 (s, 3H), 1.45 (d, *J* = 7.1 Hz, 3H), 0.80 – 0.73 (m, 12H). ^{13}C NMR (101 MHz, Chloroform-*d*) δ 169.5, 157.9, 149.7, 139.4, 135.5, 128.5, 128.3, 128.0, 126.9, 125.9, 113.5, 82.9, 55.2, 40.6, 35.8, 29.4, 29.4, 19.5, 17.3, 17.1, 14.6. HRMS (ESI) Calculated for $C_{27}H_{36}O_3$ [M+K]$^+$: 447.2296, found: 447.2291. IR ν (neat, cm^{-1}): 2964, 2934, 1707, 1511, 1463, 1248, 1196, 1076, 833. HPLC analysis CHIRALCEL® two AD-H columns, n-hexane/iso-propanol = 98.5/1.5, flow rate 0.5 mL/min, 254 nm UV detector, t_R (minor) = 26.577 min, t_R (major) = 27.077 min. $[\alpha]_D^{25}$ = -13 (c = 1.3, CH_2Cl_2); 84% ee

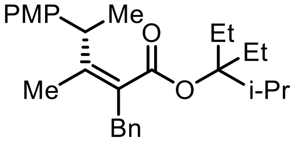

3-ethyl-2-methylpentan-3-yl (S,Z)-2-benzyl-4-(4-methoxyphenyl)-3-methylpent-2-enoate (**5c**). The reaction was carried out according to the general procedure B on 0.1 mmol scale (36 h); purified by flash column chromatography on SiO_2 (eluent: petroleum ether/ethyl acetate) to afford **5c**, 19.8 mg, 47%, light yellow oil; Rf = 0.8 (petroleum ether/ethyl acetate 10:1). ^{1}H NMR (400 MHz, Chloroform-*d*) δ 7.26 – 7.13 (m, 7H), 6.85 (d, *J* = 8.8 Hz, 2H), 4.64 (q, *J* = 7.1 Hz, 1H), 3.79 (s, 3H), 3.69 (d, *J* = 6.7 Hz, 2H), 2.32 (h, *J* = 7.0 Hz, 1H), 1.94 – 1.78 (m, 4H), 1.45 – 1.43 (m, 6H), 0.85 (d, *J* = 7.0 Hz, 6H), 0.78 (td, *J* = 7.6, 3.1 Hz, 6H). ^{13}C NMR (101 MHz, Chloroform-*d*) δ 169.1, 157.9, 147.3, 139.5, 135.6, 128.5, 128.2, 128.1, 125.8, 113.5, 91.0, 55.2, 40.5, 36.0, 33.9, 27.0, 27.0, 17.7, 17.0, 14.1, 8.8. HRMS (ESI) Calculated for $C_{28}H_{38}O_3$ [M+K]$^+$: 461.2453, found: 461.2448. IR ν (neat, cm^{-1}): 2968, 2936, 1703, 1511, 1455, 1248, 1220, 1200, 1077, 833. HPLC analysis CHIRALCEL® two AD-H columns, n-hexane/iso-propanol = 98.5/1.5, flow rate 0.5 mL/min, 254 nm UV detector, t_R (minor) = 21.223 min, t_R (major) = 23.154 min. $[\alpha]_D^{25}$ = -16 (c = 0.4, CH_2Cl_2); 90% ee

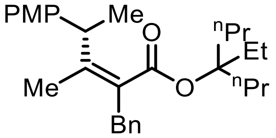

4-ethylheptan-4-yl (S,Z)-2-benzyl-4-(4-methoxyphenyl)-3-methylpent-2-enoate (**5d**). The reaction was carried out according to the general procedure B on 0.1 mmol scale (36 h); purified by flash column chromatography on SiO_2 (eluent: petroleum ether/ethyl acetate) to afford **5d**, 21.0 mg, 48%, light yellow oil; Rf = 0.8 (petroleum ether/ethyl acetate 10:1). ^{1}H NMR (400 MHz, Chloroform-*d*) δ 7.37 – 7.10 (m, 7H), 6.85 (d, *J* = 8.7 Hz, 2H), 4.60 (q, *J* = 7.1 Hz, 1H), 3.79 (s, 3H), 3.66 (d, *J* = 9.8 Hz, 2H), 1.78 (q, *J* = 7.5 Hz, 2H), 1.73 – 1.63 (m, 4H), 1.50 – 1.40 (m, 6H), 1.18 – 1.05 (m, 4H), 0.81 (t, *J* = 7.3 Hz, 6H), 0.72 (t, *J* = 7.5 Hz, 3H). ^{13}C NMR (101 MHz, Chloroform-*d*) δ 169.0, 157.9, 146.9, 139.5, 135.6, 128.5, 128.3, 128.2, 128.2, 125.8, 113.5, 88.6, 55.2, 40.6, 37.3, 36.1, 28.0, 16.9, 16.6, 14.5, 14.1, 7.8. HRMS (ESI) Calculated for $C_{29}H_{40}O_3$ [M+K]$^+$: 475.2609, found: 475.2599. IR ν (neat, cm^{-1}): 2960, 2933, 2874, 1702, 1510, 1455, 1281, 1247, 1217, 1179, 1123, 1076, 808. HPLC analysis CHIRALCEL® two AD-H columns, n-hexane/iso-propanol = 99.0/1.0, flow rate 0.5 mL/min, 254 nm UV detector, t_R (minor)

= 109.672 min, t_R (major) = 96.385 min. $[\alpha]_D^{25}$ = -27 (c = 0.3, CH_2Cl_2); 92% ee

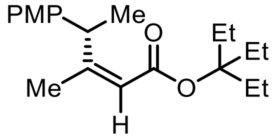

3-ethylpentan-3-yl (R,Z)-4-(4-methoxyphenyl)-3-methylpent-2-enoate (**5e**). The reaction was carried out according to the general procedure B on 0.1 mmol scale (36 h); purified by flash column chromatography on SiO_2 (eluent: petroleum ether/ethyl acetate) to afford **5e**, 21.9 mg, 69%, light yellow oil; Rf = 0.8 (petroleum ether/ethyl acetate 10:1). ^{1}H NMR (400 MHz, Chloroform-*d*) δ 7.19 (dd, *J* = 8.9, 0.7 Hz, 2H), 6.83 (d, *J* = 8.8 Hz, 2H), 5.63 (d, *J* = 1.3 Hz, 1H), 5.24 (q, *J* = 7.2 Hz, 1H), 3.78 (s, 3H), 1.88 (q, *J* = 7.5 Hz, 6H), 1.57 (d, *J* = 1.3 Hz, 3H), 1.39 (d, *J* = 7.1 Hz, 3H), 0.85 (t, *J* = 7.5 Hz, 9H). ^{13}C NMR (101 MHz, Chloroform-*d*) δ 165.78, 160.6, 157.9, 135.3, 128.4, 117.7, 113.5, 87.9, 55.2, 37.5, 26.9, 19.5, 17.0, 7.7. HRMS (ESI) Calculated for $C_{20}H_{30}O_3$ $[M+Na]^+$: 341.2087, found: 341.2072. IR ν (neat, cm^{-1}): 2969, 2942, 1703, 1639, 1510, 1457, 1247, 1222, 1130, 1040, 832. HPLC analysis CHIRALCEL® two AD-H columns, n-hexane/iso-propanol = 99.0/1.0, flow rate 0.5 mL/min, 254 nm UV detector, t_R (minor) = 23.898 min, t_R (major) = 25.037 min. $[\alpha]_D^{25}$ = -23 (c = 0.2, CH_2Cl_2); 85% ee

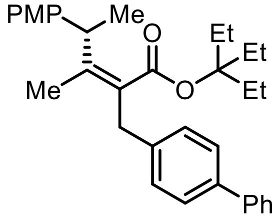

3-ethylpentan-3-yl (S,Z)-2-([1,1'-biphenyl]-4-ylmethyl)-4-(4-methoxyphenyl)-3-methylpent-2-enoate (**5f**). The reaction was carried out according to the general procedure B on 0.1 mmol scale (36 h); purified by flash column chromatography on SiO_2 (eluent: petroleum ether/ethyl acetate) to afford **5f**, 30.1 mg, 62%, light yellow oil; Rf = 0.8 (petroleum ether/ethyl acetate 10:1). ^{1}H NMR (400 MHz, Chloroform-*d*) δ 7.60 – 7.54 (m, 2H), 7.51 – 7.46 (m, 2H), 7.42 (dd, *J* = 8.4, 6.9 Hz, 2H), 7.34 – 7.23 (m, 5H), 6.89 – 6.82 (m, 2H), 4.64 (q, *J* = 7.0 Hz, 1H), 3.79 (s, 3H), 3.71 (d, *J* = 9.2 Hz, 2H), 1.80 (q, *J* = 7.5 Hz, 6H), 1.48 (s, 3H), 1.45 (d, *J* = 7.0 Hz, 3H), 0.73 (t, *J* = 7.5 Hz, 9H). ^{13}C NMR (101 MHz, Chloroform-*d*) δ 169.0, 157.9, 147.2, 141.2, 138.8, 135.6, 128.7, 128.6, 128.5, 128.2, 127.0, 127.0, 113.5, 89.1, 55.2, 40.6, 35.8, 26.9, 17.0, 14.2, 7.6. HRMS (ESI) Calculated for $C_{33}H_{40}O_3$ $[M+K]^+$: 523.2609, found: 523.2605. IR ν (neat, cm^{-1}): 2969, 2938, 1702, 1510, 1457, 1285, 1247, 1179, 1072, 832, 758, 698. HPLC analysis CHIRALCEL® two AD-H columns, n-hexane/iso-propanol = 98.5/1.5, flow rate 0.5 mL/min, 254 nm UV detector, t_R (minor) = 31.609 min, t_R (major) = 27.815 min. $[\alpha]_D^{25}$ = -11 (c = 1.0, CH_2Cl_2); 91% ee

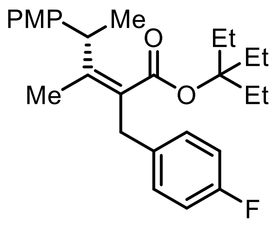

3-ethylpentan-3-yl (S,Z)-2-(4-fluorobenzyl)-4-(4-methoxyphenyl)-3-methylpent-2-enoate (**5g**). The reaction was carried out according to the general procedure B on 0.1 mmol scale (36 h); purified by flash column chromatography on SiO_2 (eluent: petroleum ether/ethyl acetate) to afford **5g**, 29.9 mg, 70%, light yellow oil; Rf = 0.8 (petroleum ether/ethyl acetate 10:1). ^{1}H NMR (400 MHz, Chloroform-*d*) δ 7.23 (d, *J* = 8.1 Hz, 2H), 7.15 – 7.11 (m, 2H), 6.94 (t, *J* = 8.8 Hz, 2H), 6.85 (d, *J* = 8.9 Hz, 2H), 4.61 (q, *J* = 7.0 Hz, 1H), 3.79 (s, 3H), 3.68 – 3.58 (m, 2H), 1.79 (q, *J* = 7.5 Hz, 6H), 1.45 – 1.42 (m, 6H), 0.72 (t, *J* = 7.5 Hz, 9H). ^{19}F NMR (376 MHz, Chloroform-*d*) δ -117.80. ^{13}C NMR (101 MHz, Chloroform-*d*) δ 168.8, 161.3 (d, *J* = 243.4 Hz), 157.9, 147.3, 135.4, 135.2 (d, *J* = 3.2 Hz), 129.4 (d, *J* = 7.7 Hz), 128.4, 128.1, 115.0 (d, *J* = 21.3 Hz), 113.5, 89.2, 55.2, 40.6, 35.3, 26.9, 16.9, 14.1, 7.6. HRMS (ESI) Calculated for $C_{27}H_{35}FO_3$ $[M+Na]^+$: 449.2462, found: 449.2453. IR ν (neat, cm^{-1}): 2970, 2939, 1702, 1509, 1458, 1285, 1247, 1221, 1179, 1040, 832. HPLC analysis CHIRALCEL® two AD-H columns, n-hexane/iso-propanol = 98.5/1.5, flow rate 0.5 mL/min, 254 nm UV detector, t_R (minor) = 22.312 min, t_R (major) = 23.787 min. $[\alpha]_D^{25}$ = -13 (c = 0.6, CH_2Cl_2); 86% ee

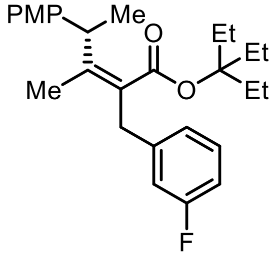

3-ethylpentan-3-yl (Z)-2-(3-fluorobenzyl)-4-(4-methoxyphenyl)-3-methylpent-2-enoate (**5h**). The reaction was carried out according to the general procedure B on 0.1 mmol scale (36 h); purified by flash column chromatography on SiO_2 (eluent: petroleum ether/ethyl acetate) to afford **5h**, 26.0 mg, 61%, light yellow oil; Rf = 0.8 (petroleum ether/ethyl acetate 10:1). ^{1}H NMR (400 MHz, Chloroform-*d*) δ 7.28 – 7.16 (m, 3H), 7.02 – 6.94 (m, 1H), 6.94 – 6.82 (m, 4H), 4.65 (q, *J* = 7.1 Hz, 1H), 3.79 (s, 3H), 3.66 (d, *J* = 7.7 Hz, 2H), 1.79 (q, *J* = 7.5 Hz, 6H), 1.44 (d, *J* = 7.2 Hz, 6H), 0.73 (t, *J* = 7.5 Hz, 9H). ^{19}F NMR (376 MHz, Chloroform-*d*) δ -113.95. ^{13}C NMR (101 MHz, Chloroform-*d*) δ 168.7, 163.0 (d, *J* = 244.8 Hz), 157.9, 148.1, 142.3 (d, *J* = 6.8 Hz), 135.4, 129.6 (d, *J* = 8.2 Hz), 128.4, 127.5, 123.8 (d, *J* = 2.7 Hz), 114.9 (d, *J* = 21.3 Hz), 113.6, 112.7 (d, *J* = 21.3 Hz), 89.3, 55.2, 40.6, 35.8, 35.8, 26.9, 16.9, 14.2, 7.6. HRMS (ESI) Calculated for $C_{27}H_{35}FO_3$ $[M+K]^+$: 465.2202, found: 465.2193. IR ν (neat, cm^{-1}): 2969, 2940, 1702, 1613, 1510, 1454, 1284, 1246, 1178, 1133, 1073, 1038, 776. HPLC analysis CHIRALCEL® two AD-H columns, n-hexane/iso-propanol = 98.5/1.5, flow rate 0.5 mL/min, 254 nm UV detector, t_R (minor) = 23.029 min, t_R (major) = 24.539 min. $[\alpha]_D^{25}$ = -15 (c = 0.6, CH_2Cl_2); 90% ee

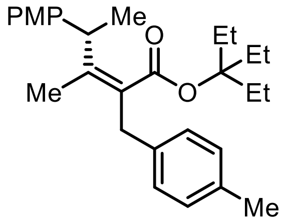

3-ethylpentan-3-yl (S,Z)-4-(4-methoxyphenyl)-3-methyl-2-(4-methylbenzyl)pent-2-enoate (**5i**). The reaction was carried out according to the general procedure B on 0.1 mmol scale (36 h); purified by flash column chromatography on SiO_2 (eluent: petroleum ether/ethyl acetate) to afford **5i**, 28.8 mg, 68%, light yellow oil; Rf = 0.8 (petroleum ether/ethyl acetate 10:1). ^{1}H NMR (400 MHz, Chloroform-*d*) δ 7.25 – 7.23 (m, 2H), 7.08 – 7.04 (m, 4H), 6.85 (d, *J* = 8.8 Hz, 2H), 4.59 (q, *J* = 7.0 Hz, 1H), 3.79 (s, 3H), 3.63 (d, *J* = 10.0 Hz, 2H), 2.29 (s, 3H), 1.79 (t, *J* = 7.5 Hz, 6H), 1.42 (d, *J* = 7.3 Hz, 6H), 0.73 (t, *J* = 7.5 Hz, 9H). ^{13}C NMR (101 MHz, Chloroform-*d*) δ 169.1, 157.8, 146.8, 136.4, 135.6, 135.2, 128.9, 128.5, 128.0, 113.5, 89.0, 55.2, 40.6, 35.6, 26.9, 21.0, 16.9, 14.1, 7.6. HRMS (ESI) Calculated for $C_{28}H_{38}O_3$ $[M+K]^+$: 461.2453, found: 461.2444. IR ν (neat, cm^{-1}): 2969, 2941, 1702, 1511, 1284, 1248, 1179, 1040, 832. HPLC analysis CHIRALCEL® two AD-H columns, n-hexane/iso-propanol = 98.5/1.5, flow rate 0.5 mL/min, 254 nm UV detector, t_R (minor) = 22.294 min, t_R (major) = 23.385 min. $[\alpha]_D^{25}$ = -16 (c = 0.5, CH_2Cl_2); 91% ee

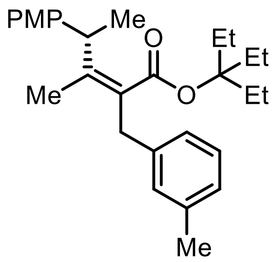

3-ethylpentan-3-yl (S,Z)-4-(4-methoxyphenyl)-3-methyl-2-(3-methylbenzyl)pent-2-enoate (**5j**). The reaction was carried out according to the general procedure B on 0.1 mmol scale (36 h); purified by flash column chromatography on SiO_2 (eluent: petroleum ether/ethyl acetate) to afford **5j**, 30.5 mg, 72%, light yellow oil; Rf = 0.8 (petroleum ether/ethyl acetate 10:1). ^{1}H NMR (400 MHz, Chloroform-*d*) δ 7.26 – 7.24 (m, 2H), 7.13 (t, *J* = 7.5 Hz, 1H), 7.00 – 6.94 (m, 3H), 6.85 (d, *J* = 8.8 Hz, 2H), 4.62 (q, *J* = 7.1 Hz, 1H), 3.79 (s, 3H), 3.69 – 3.58 (m, 2H), 2.30 (s, 3H), 1.79 (q, *J* = 7.6 Hz, 6H), 1.44 – 1.42 (m, 6H), 0.73 (t, *J* = 7.5 Hz, 9H). ^{13}C NMR (101 MHz, Chloroform-*d*) δ 169.0, 157.8, 146.9, 139.5, 137.6, 135.6, 129.0, 128.5, 128.3, 128.1, 126.5, 125.2, 113.5, 89.0, 55.2, 40.6, 35.9, 26.9, 21.4, 16.9, 14.1, 7.6. HRMS (ESI) Calculated for $C_{28}H_{38}O_3$ $[M+K]^+$: 461.2453, found: 461.2445. IR ν (neat, cm^{-1}): 2969, 2939, 1702, 1510, 1457, 1284, 1217, 1135, 1072, 832. HPLC analysis CHIRALCEL® two AD-H columns, n-hexane/iso-propanol = 98.5/1.5, flow rate 0.5 mL/min, 254 nm UV detector, t_R (minor) = 21.487 min, t_R (major) = 22.989 min. $[\alpha]_D^{25}$ = -13 (c = 0.7, CH_2Cl_2); 92% ee

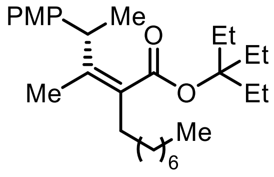

3-ethylpentan-3-yl (S,Z)-2-(3-(4-methoxyphenyl)butan-2-ylidene)decanoate (**5k**). The reaction was carried out according to the general procedure B on 0.1 mmol scale (36 h); purified by flash column chromatography on SiO_2 (eluent: petroleum ether/ethyl acetate) to afford **5k**, 21.6 mg, 50%, light yellow oil; Rf = 0.8 (petroleum ether/ethyl acetate 10:1). ^{1}H NMR (400 MHz, Chloroform-*d*) δ 7.21 – 7.19 (m, 2H), 6.83 (d, *J* = 8.8 Hz, 2H), 4.35 (q, *J* = 7.1 Hz, 1H), 3.78 (s, 3H), 2.30 – 2.18 (m, 2H), 1.89 (t, *J* = 7.5 Hz, 6H), 1.45 – 1.26 (m, 18H), 0.89 – 0.84 (m, 12H). ^{13}C NMR (101 MHz, Chloroform-*d*) δ 170.0, 157.7, 143.0, 135.8, 130.5, 128.4, 113.4, 88.7, 55.2, 40.7, 31.8, 30.6, 29.6, 29.4, 29.3, 28.8, 27.0, 22.7, 16.9, 14.1, 13.1, 7.8. HRMS (ESI) Calculated for $C_{28}H_{46}O_3$ $[M+K]^+$: 469.3079, found: 469.3069. IR ν (neat, cm^{-1}): 2965, 2925, 1703, 1510, 1458, 1282, 1178, 1116, 1038, 832. HPLC analysis CHIRALCEL® two AD-H columns, n-hexane/iso-propanol = 99.0/1.0, flow rate 0.5 mL/min, 254 nm UV detector, t_R (minor) = 65.871 min, t_R (major) = 44.660 min. $[\alpha]_D^{25}$ = -13 (c = 0.5, CH_2Cl_2); 90% ee

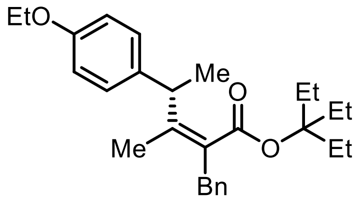

3-ethylpentan-3-yl (S,Z)-2-benzyl-4-(4-ethoxyphenyl)-3-methylpent-2-enoate (**5l**). The reaction was carried out according to the general procedure B on 0.1 mmol scale (36 h); purified by flash column chromatography on SiO_2 (eluent: petroleum ether/ethyl acetate) to afford **5l**, 27.5 mg, 65%, light yellow oil; Rf = 0.8 (petroleum ether/ethyl acetate 10:1). ^{1}H NMR (400 MHz, Chloroform-*d*) δ 7.27 – 7.13 (m, 7H), 6.86 – 6.82 (m, 2H), 4.62 (q, *J* = 7.1 Hz, 1H), 4.02 (q, *J* = 7.0 Hz, 2H), 3.72 – 3.62 (m, 2H), 1.78 (q, *J* = 7.5 Hz, 6H), 1.45 – 1.38 (m, 9H), 0.71 (t, *J* = 7.5 Hz, 9H). ^{13}C NMR (101 MHz, Chloroform-*d*) δ 169.0, 157.2, 147.2, 139.6, 135.4, 128.4, 128.2, 128.2, 128.1, 125.8, 114.1, 89.0, 63.3, 40.6, 36.1, 26.9, 16.9, 14.9, 14.2, 7.6. HRMS (ESI) Calculated for $C_{28}H_{38}O_3$ $[M+K]^+$: 461.2453, found: 461.2442. IR ν (neat, cm^{-1}): 2971, 2937, 1703, 1510, 1454, 1282, 1245, 1220, 1135, 1074, 835, 737. HPLC analysis CHIRALCEL® two AD-H columns, n-hexane/iso-propanol = 99.0/1.0, flow rate 0.5 mL/min, 254 nm UV detector, t_R (minor) = 26.749 min, t_R (major) = 24.300 min. $[\alpha]_D^{25}$ = -17 (c = 0.4, CH_2Cl_2); 88% ee

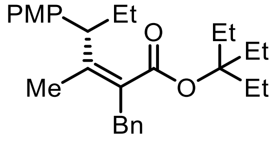

3-ethylpentan-3-yl (S,Z)-2-benzyl-4-(4-methoxyphenyl)-3-methylhex-2-enoate (**5m**). The reaction was carried out according to the general procedure B on 0.1 mmol scale (36 h); purified by flash column chromatography on SiO_2 (eluent: petroleum ether/ethyl acetate) to afford **5m**,

24.1 mg, 57%, light yellow oil; Rf = 0.8 (petroleum ether/ethyl acetate 10:1). ^{1}H NMR (400 MHz, Chloroform-*d*) δ 7.32 – 7.29 (m, 2H), 7.25 – 7.12 (m, 5H), 6.87 – 6.83 (m, 2H), 4.39 (dd, *J* = 9.1, 6.4 Hz, 1H), 3.79 (s, 3H), 3.68 (d, *J* = 6.5 Hz, 2H), 1.97 – 1.87 (m, 1H), 1.82 – 1.77 (m, 7H), 1.45 (s, 3H), 0.96 (t, *J* = 7.4 Hz, 3H), 0.73 (t, *J* = 7.5 Hz, 9H). ^{13}C NMR (101 MHz, Chloroform-*d*) δ 169.1, 157.8, 145.5, 139.7, 134.8, 129.4, 128.9, 128.2, 125.8, 113.5, 89.1, 55.2, 47.8, 36.3, 27.0, 23.7, 13.8, 12.2, 7.7. HRMS (ESI) Calculated for $C_{28}H_{38}O_3$ $[M+K]^+$: 461.2453, found: 461.2448. IR ν (neat, cm^{-1}): 2966, 2934, 1701, 1510, 1454, 1245, 1210, 1178, 1134, 1088, 866, 732. HPLC analysis CHIRALCEL® two AD-H columns, n-hexane/iso-propanol = 99.0/1.0, flow rate 0.5 mL/min, 254 nm UV detector, t_R (minor) = 24.495 min, t_R (major) = 26.007 min. $[\alpha]_D^{25}$ = -18 (c = 0.5, CH_2Cl_2); 90% ee

5. Investigation of the reaction mechanism

5.1 Radical inhibition experiments with TEMPO

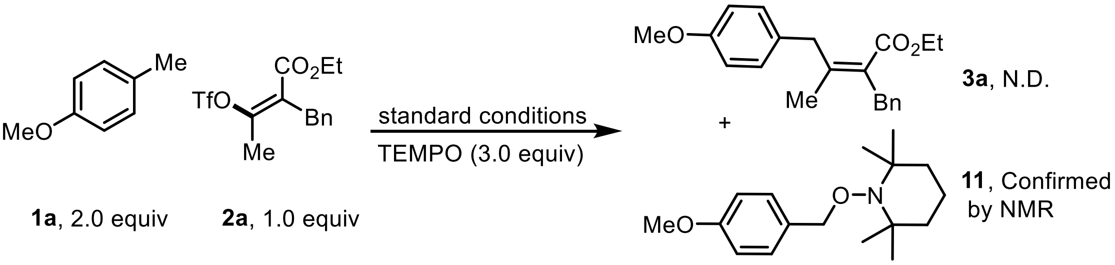

The reaction was completely inhibited by TEMPO. The isolated compound **11** indicated that the reaction probably proceeded via a radical process.

5.2 Byproducts detected experiments

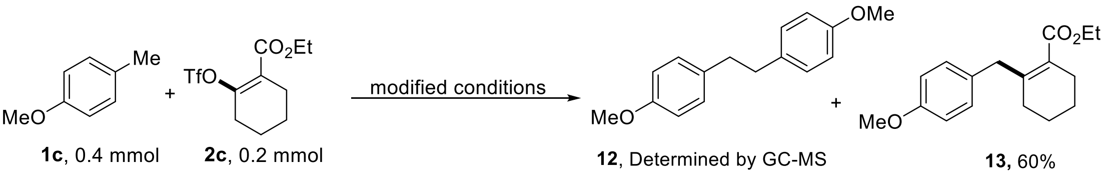

Modified conditions: Photocatalyst $[Ir(dF(CF_3)ppy)_2(5,5'\text{-}dFbpy)]PF_6$ (4.2 mg, 2 mol%), $NiCl_2\bullet 6H_2O$ (4.8 mg, 10 mol%), 4,4'-Di-tert-butyl-2,2'-bipyridine (8.0 mg, 15%) and K_2HPO_4 (34.8 mg, 0.2 mmol, 1 equiv) were added to an oven-dried 10 mL Schlenk tube equipped with a magnetic stirring bar. The tube was evacuated and backfilled with argon three times. Subsequently, DMF was added under argon. Benzylic compound (0.4 mmol, 2 equiv), alkenyl triflates (0.2 mmol, 1 equiv) were then added. The tube was then sealed and placed ~5 cm from 2 × 45 W blue LEDs. The reaction mixture was stirred for 24 h at room temperature (air-condition was used to keep the temperature is 25 °C or so). After completion, the reaction

mixture was removed from the light, diluted with water and the aqueous layer was extracted with EtOAc (3 × 2 mL). The combined organic layers were washed with brine, dried over anhydrous Na_2SO_4, filtered, and concentrated. The residue was purified by flash chromatography on silica gel to afford the corresponding products.
We found the byproduct **12**, which further confirms the existence of the benzyl radical.

5.3 Kinetic experiments

5.3.1 The total reaction profile

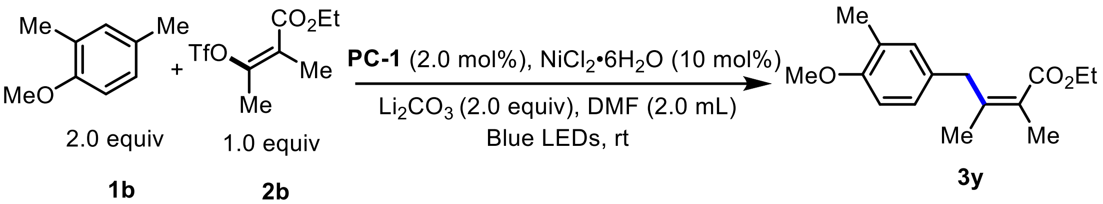

1b **2b** **3y**

Photocatalyst [Ir(dF(CF$_3$)ppy)$_2$(4,4'-dCF$_3$bpy)]PF$_6$ (4.8 mg, 2 mol%), benzylic compound (0.4 mmol, 2.0 equiv), alkenyl triflates (0.2 mmol, 1.0 equiv), $NiCl_2$•$6H_2O$ (4.8 mg, 10 mol%), and anhydrous powder Li_2CO_3 (29.6 mg, 0.4 mmol, 2.0 equiv) were added to an oven-dried 10 mL Schlenk tube equipped with a magnetic stirring bar. The tube was evacuated and backfilled with argon three times. Subsequently, DMF was added under argon. The tube was then sealed and placed ~5 cm from 2 × 45 W blue LEDs. The reaction mixture was stirred at room temperature (air-condition was used to keep the temperature is 25 °C or so). At 5, 10, 15, 20, 25, 30, 35, 40, 50, 60, 80, 100, 120, 150, 180, 240, 300, 360, 420, 480, 600 min, 20 μL the reaction mixture was carefully taken out by micro-syringe into 2.0 mL vial. Then 1.0 ml EA was added into the vial. The reaction mixture was analyzed by GC-MS after filtration.

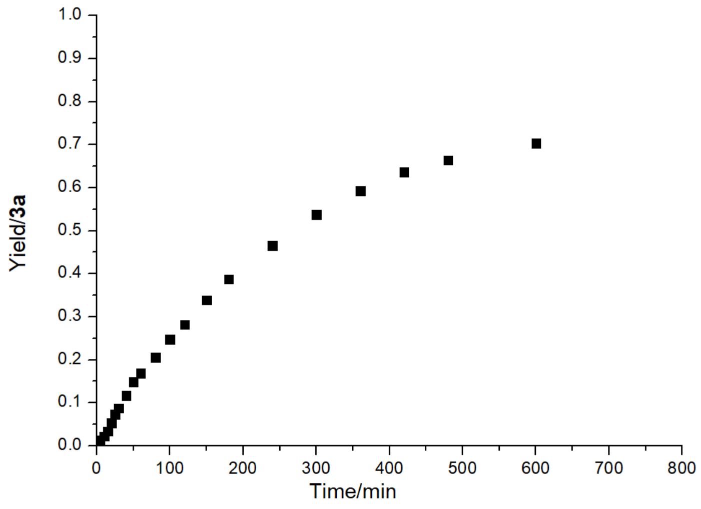

Supplementary Figure 3. The total reaction profile

5.3.2 Dependence of the reaction rate on concentration of $NiCl_2$•$6H_2O$

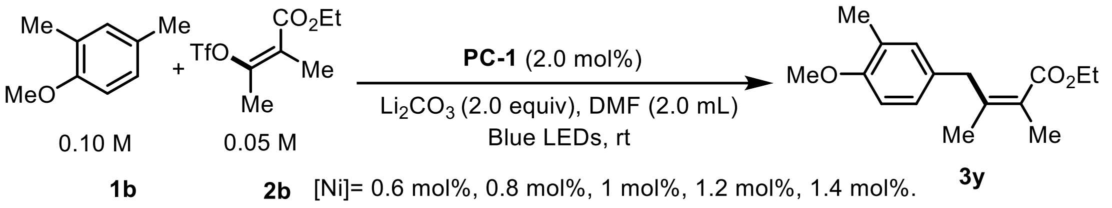

Photocatalyst [Ir(dF(CF$_3$)ppy)$_2$(4,4'-dCF$_3$bpy)]PF$_6$ (4.8 mg, 2 mol%), benzylic compound (0.4 mmol, 2.0 equiv), alkenyl triflates (0.2 mmol, 1.0 equiv), NiCl$_2$•6H$_2$O (0.6 mol%, 0.8 mol%, 1.0 mol%, 1.2 mol%, 1.4 mol%), and anhydrous powder Li$_2$CO$_3$ (29.6 mg, 0.4 mmol, 2.0 equiv) were added to an oven-dried 10 mL Schlenk tube equipped with a magnetic stirring bar. The tube was evacuated and backfilled with argon three times. Subsequently, DMF was added under argon. The tube was then sealed and placed ~5 cm from 2 × 45 W blue LEDs. The reaction mixture was stirred for 24 h at room temperature (air-condition was used to keep the temperature is 25 °C or so). At 6, 12, 18, 24, 30, 36, 42, 48, 54, 60 min, 20 μL the reaction mixture was carefully taken out by micro-syringe into 2.0 mL vial. Then 1.0 ml EA was added into the vial. The reaction mixture was analyzed by GC-MS after filtration.

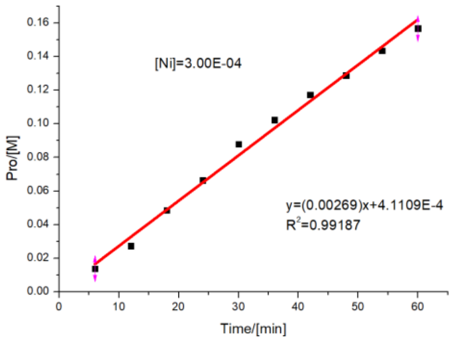

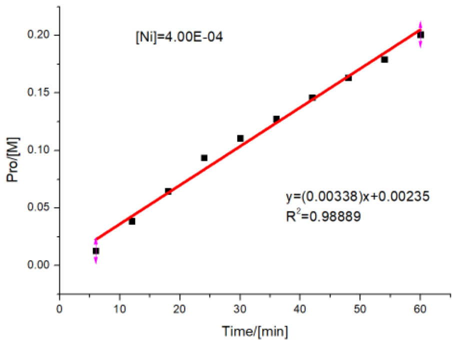

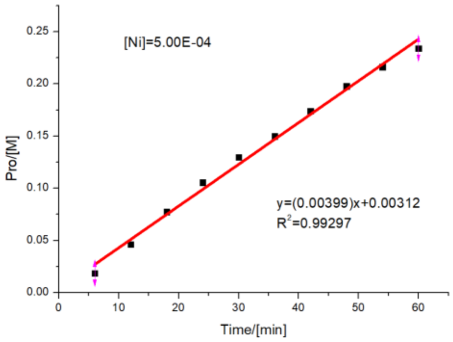

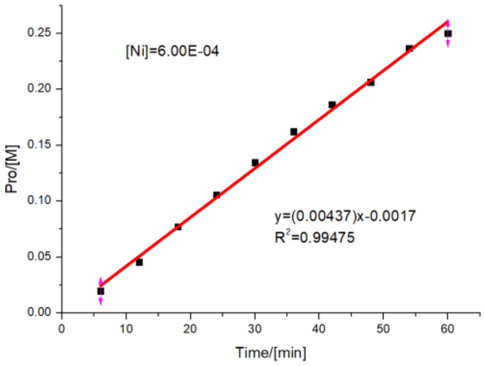

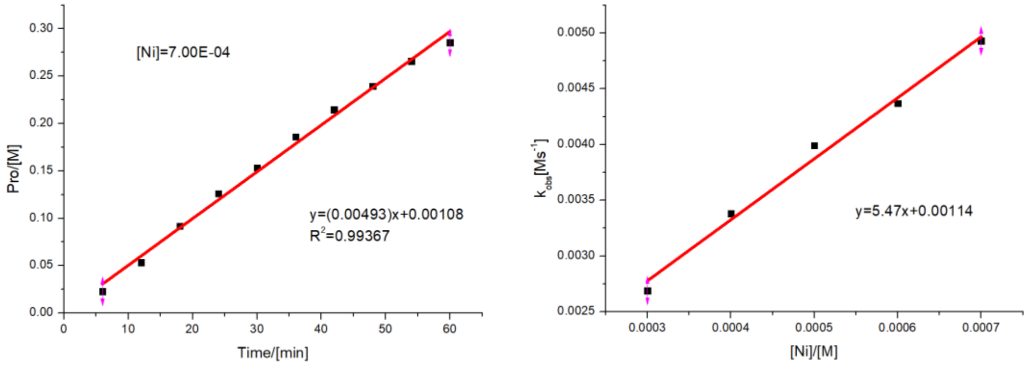

Supplementary Figure 4. Dependence of the reaction rate on loading of [Ni]

The plot of *Kobs* vs [**Ni**] displayed a linear relationship in [**Ni**], which should suggest a first-order kinetic dependence in [**Ni**].

5.3.3 Dependence of the reaction rate on concentration of **PC-1**

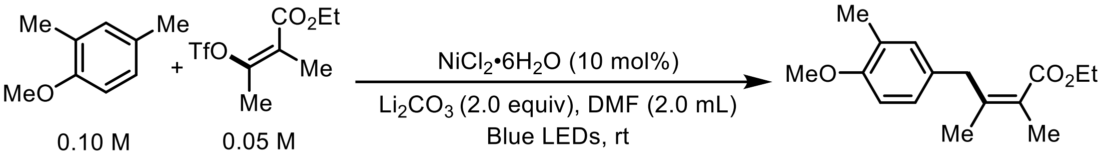

1b **2b** [**PC-1**]= 0.6 mol%, 0.8 mol%, 1 mol%, 1.2 mol%, 1.4 mol%. **3y**

Photocatalyst [Ir(dF(CF$_3$)ppy)$_2$(4,4'-dCF$_3$bpy)]PF$_6$ (0.6 mol%, 0.8 mol%, 1.0 mol%, 1.2 mol%, 1.4 mol%), benzylic compound (0.4 mmol, 2.0 equiv), alkenyl triflates (0.2 mmol, 1.0 equiv), $NiCl_2$•$6H_2O$ (10 mol%), and anhydrous powder Li_2CO_3 (29.6 mg, 0.4 mmol, 2.0 equiv) were added to an oven-dried 10 mL Schlenk tube equipped with a magnetic stirring bar. The tube was evacuated and backfilled with argon three times. Subsequently, DMF was added under argon. The tube was then sealed and placed ~5 cm from 2 × 45 W blue LEDs. The reaction mixture was stirred for 24 h at room temperature (air-condition was used to keep the temperature is 25 °C or so). At 6, 12, 18, 24, 30, 36, 42, 48, 54, 60 min, 20 μL the reaction mixture was carefully taken out by micro-syringe into 2.0 mL vial. Then 1.0 ml EA was added into the vial. The reaction mixture was analyzed by GC-MS after filtration.

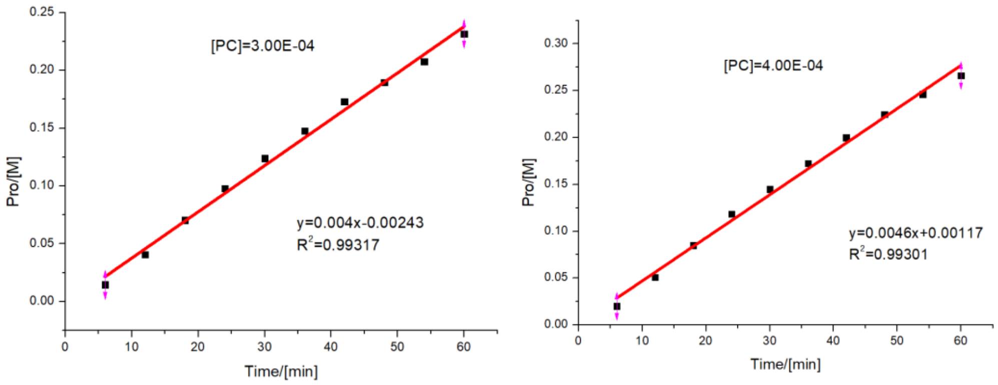

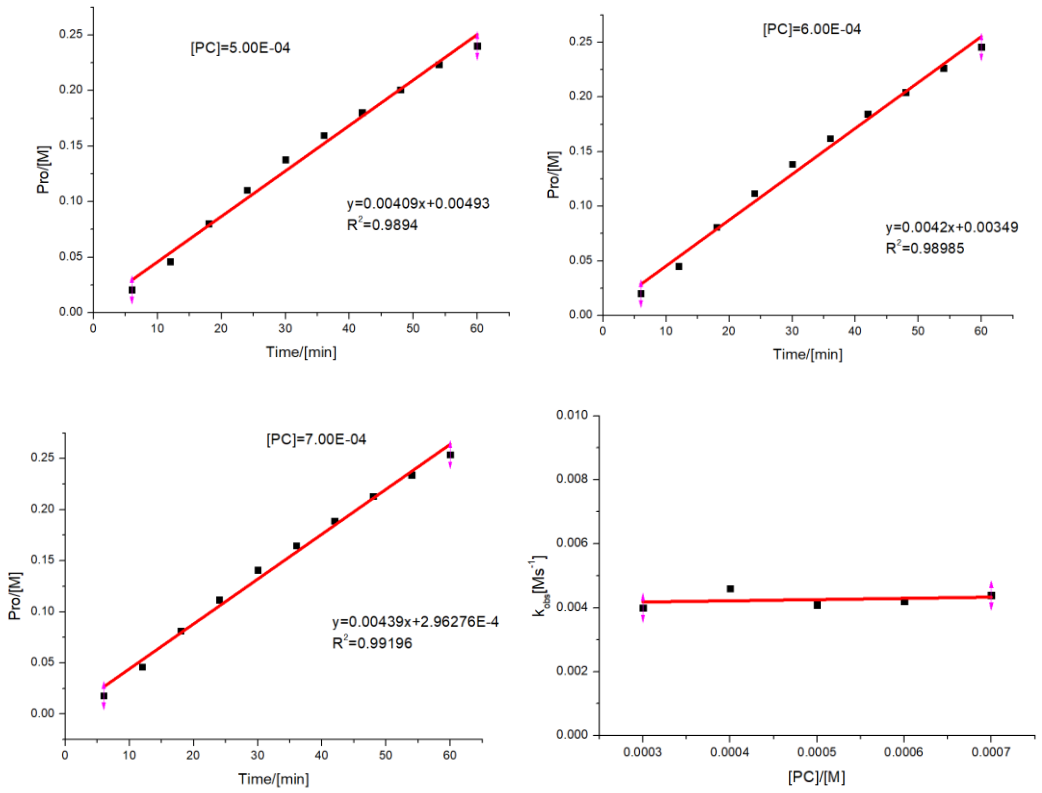

Supplementary Figure 5. Dependence of the reaction rate on [**PC-1**]

The plot of *Kobs* vs [**PC-1**] suggests a zero-order kinetic dependence in [**PC-1**].

5.3.4 Dependence of the reaction rate on concentration of **1b**

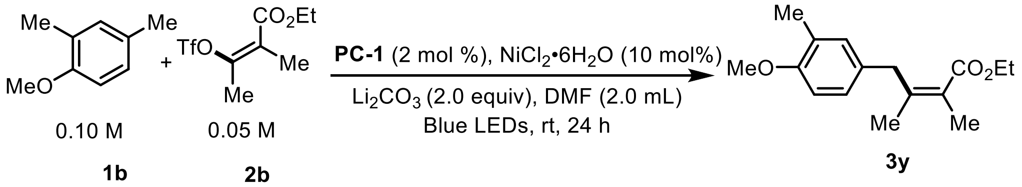

0.075 M
0.0875 M
0.1 M
0.113 M
0.125 M

Photocatalyst [Ir(dF(CF$_3$)ppy)$_2$(4,4'-dCF$_3$bpy)]PF$_6$ (2 mol%), benzylic compound (0.3 mmol, 0.35 mmol, 0.40 mmol, 0.45 mmol, 0.50 mmol), alkenyl triflates (0.2 mmol, 1.0 equiv), $NiCl_2$ •$6H_2O$ (10 mol%), and anhydrous powder Li_2CO_3 (29.6 mg, 0.4 mmol, 2.0 equiv) were added to an oven-dried 10 mL Schlenk tube equipped with a magnetic stirring bar. The tube was evacuated and backfilled with argon three times. Subsequently, DMF was added under argon. The tube was then sealed and placed ~5 cm from 2 × 45 W blue LEDs. The reaction mixture was stirred for 24 h at room temperature (air-condition was used to keep the temperature is 25 °C or so). At 6, 12, 18, 24, 30, 36, 42, 48, 54, 60 min, 20 μL the reaction mixture was carefully taken out by micro-syringe into 2.0 mL vial. Then 1.0 ml EA was added into the vial. The reaction mixture was analyzed by GC-MS after filtration.

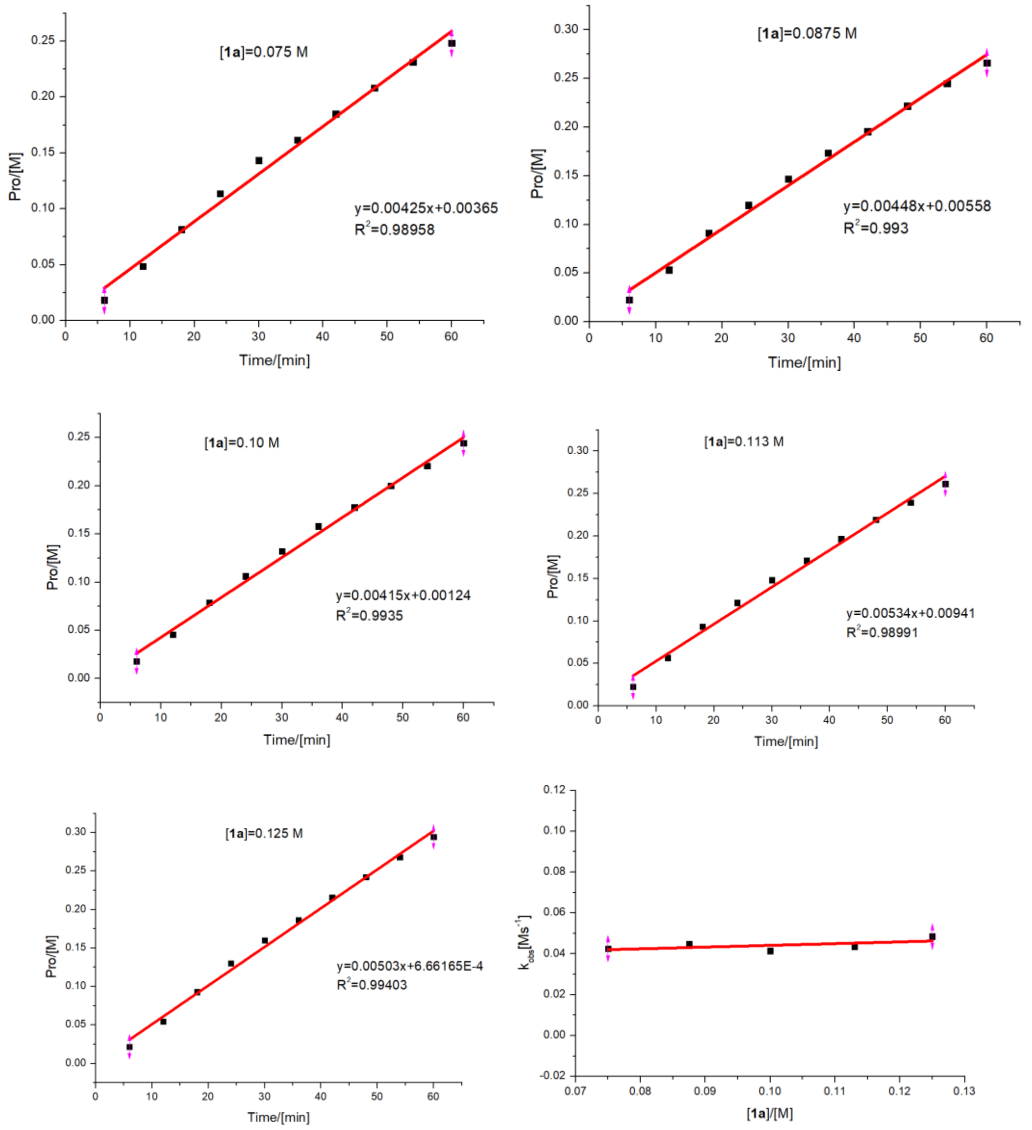

Supplementary Figure 6. Dependence of the reaction rate on [**1b**]

The plot of *Kobs* vs [**1a**] shows the same reaction rate regardless of the initial concentrations of **1a**, which would suggest a zero-order kinetic dependence in [**1b**].

5.3.5 Dependence of the reaction rate on concentration of **2b**

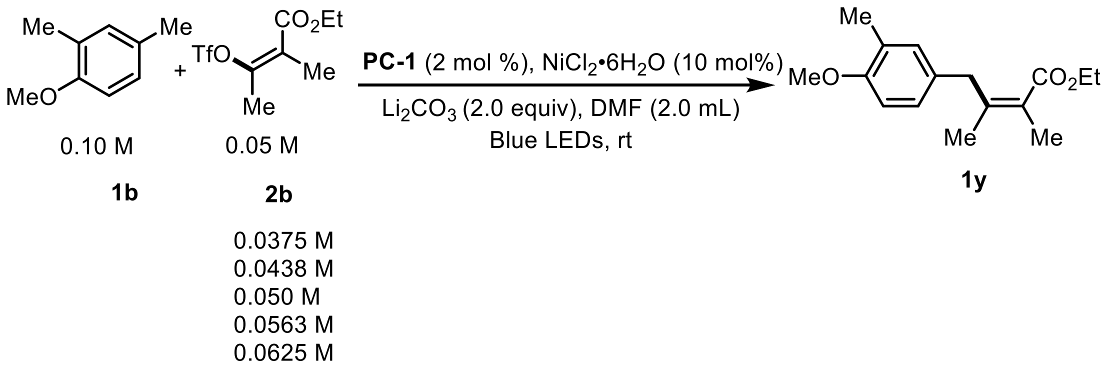

Photocatalyst [Ir(dF(CF_3)ppy)$_2$(4,4'-dCF_3bpy)]PF_6 (2 mol%), benzylic compound (0.4 mmol),

alkenyl triflates (0.15 mmol, 0.175 mmol, 0.20 mmol, 0.225 mmol, 0.25 mmol), $NiCl_2 \cdot 6H_2O$ (10 mol%), and anhydrous powder Li_2CO_3 (29.6 mg, 0.4 mmol, 2.0 equiv) were added to an oven-dried 10 mL Schlenk tube equipped with a magnetic stirring bar. The tube was evacuated and backfilled with argon three times. Subsequently, DMF was added under argon. The tube was then sealed and placed ~5 cm from 2 × 45 W blue LEDs. The reaction mixture was stirred for 24 h at room temperature (air-condition was used to keep the temperature is 25 °C or so). At 6, 12, 18, 24, 30, 36, 42, 48, 54, 60 min, 20 μL the reaction mixture was carefully taken out by micro-syringe into 2.0 mL vial. Then 1.0 ml EA was added into the vial. The reaction mixture was analyzed by GC-MS after filtration.

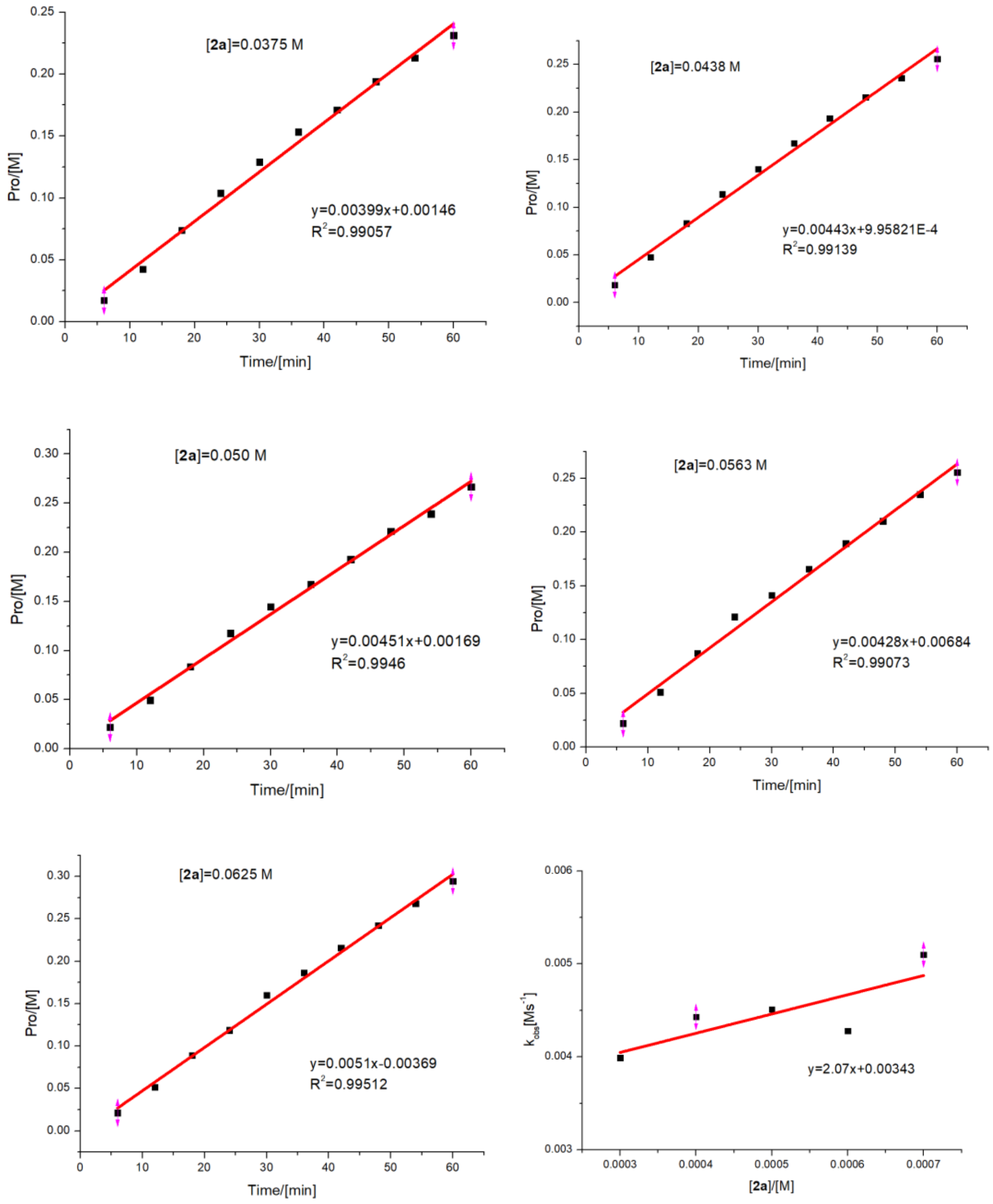

Supplementary Figure 7. Dependence of the reaction rate on [**2b**]

The plot of *Kobs* vs [**2a**] suggests a first-order kinetic dependence in [**2b**].

5.4 Luminescence quenching experiment

The luminescence quenching experiment was taken using a F-7000 FL Spectrophotometer (Hitachi, Japan). The experiments were carried out in 1 x 10^{-6} mol/L of [Ir(dF(CF_3)ppy)$_2$(4,4'-dCF$_3$bpy)]PF_6 in DMF at 25 °C. The excitation wavelength was 315 nm and the emission intensity was collected at 581 nm. The concentrations of quenchers (1a and 2a) in DMF were 0, 6, 12, 20, 30, 42 mM.

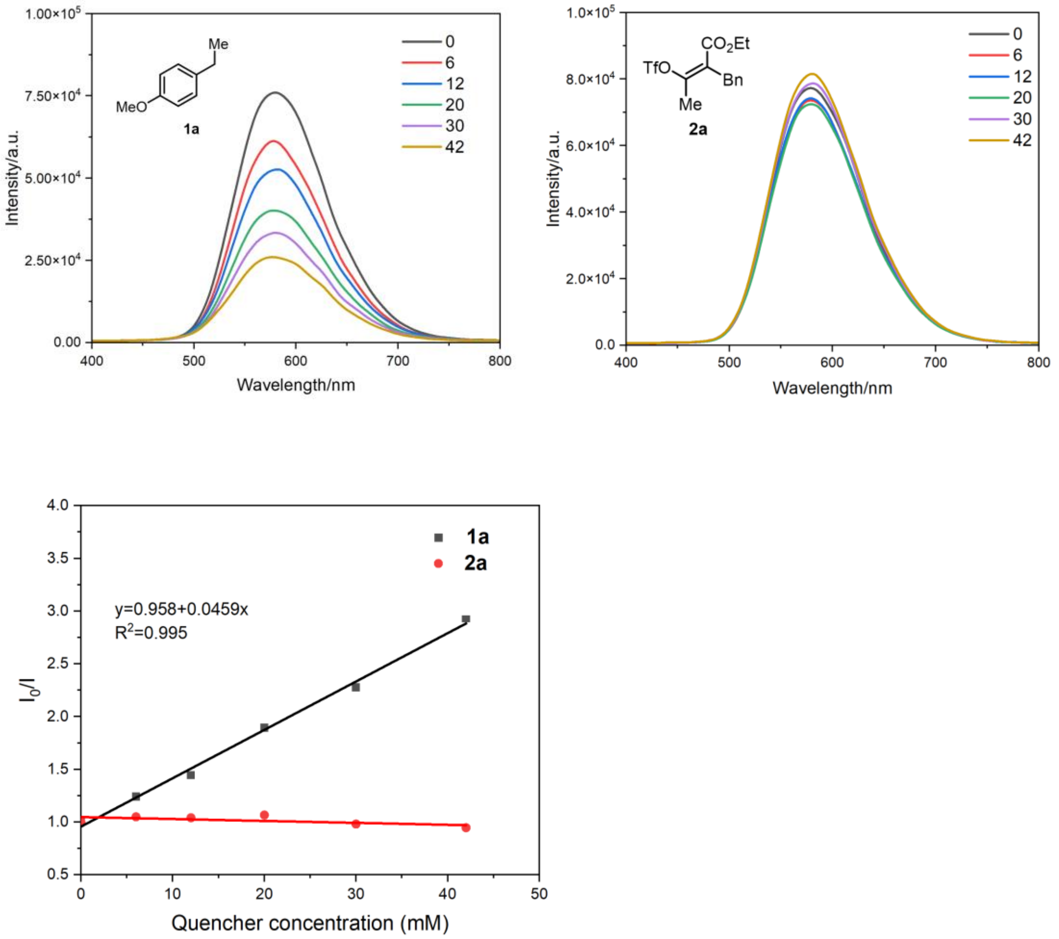

Supplementary Figure 8. The data of fluorescence quenching of [Ir(dF(CF_3)ppy)$_2$(4,4'-dCF$_3$bpy)]PF_6 by **1a** and **2a**.

To determine whether a reductive or oxidative quenching cycle is operative in the reaction, fluorescence quenching studies were conducted. Based on the above data, photoexcited [Ir(dF(CF_3)ppy)$_2$(4,4'- dCF$_3$bpy)]PF_6* can be quenched by **1a**, involving a reductive quenching cycle.

5.5 The reactivity of Z/E vinyl triflates

We have prepared the Z and E vinyl triflate. And we have done the following experiments. (reactions 1-3)

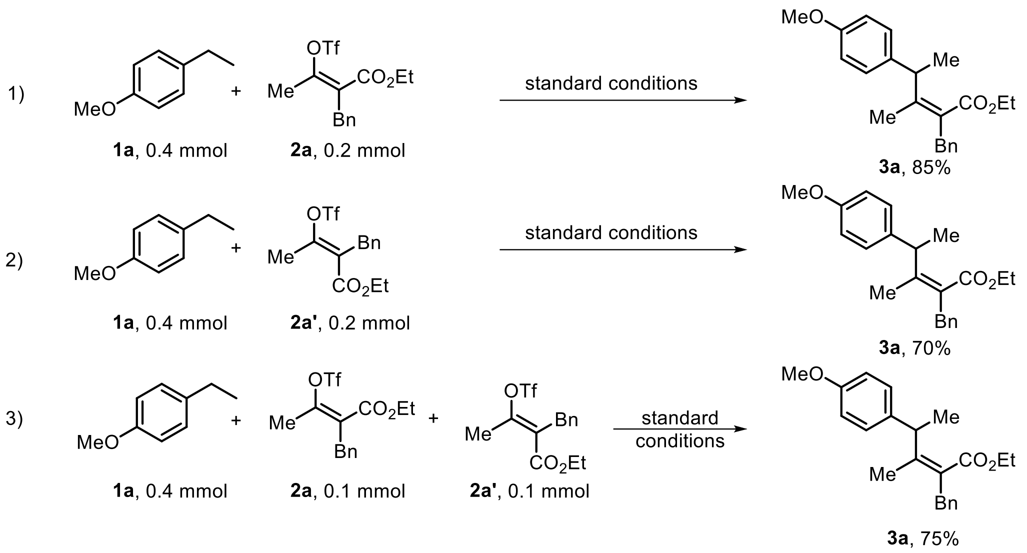

When defined Z, E, or E/Z mixture of the vinyl triflates are treated as substrates, only the Z product is formed in high yields.

For three experiments, the final reaction mixture was first analyzed by GC-MS, proving these reactions produce the same product, without any isomer. Their NMR spectrum are also the same. The NOESY spectrum verified the Z product.

Thus, we speculate that the ester group on the side chain surely plays a role in the stabilization of the transition state with coordination to the nickel center.

5.5.1 GC-MS spectrum

For reaction 1, the GC-MS spectrum of the reaction mixture is as follows:

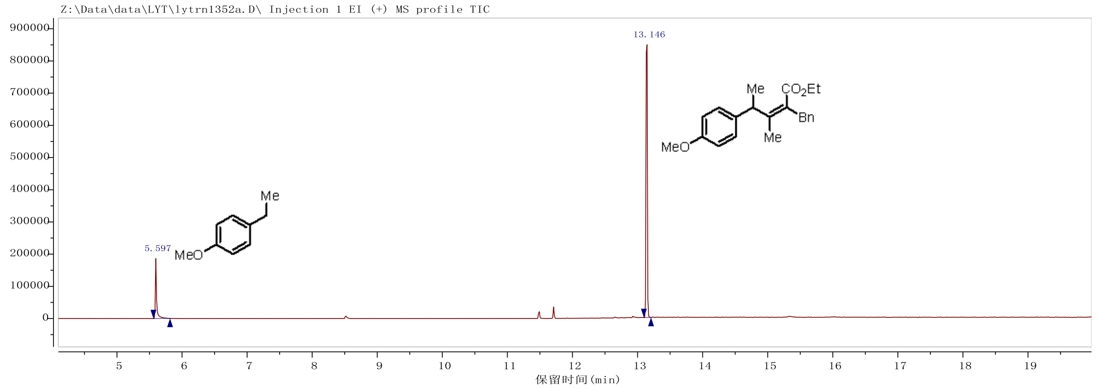

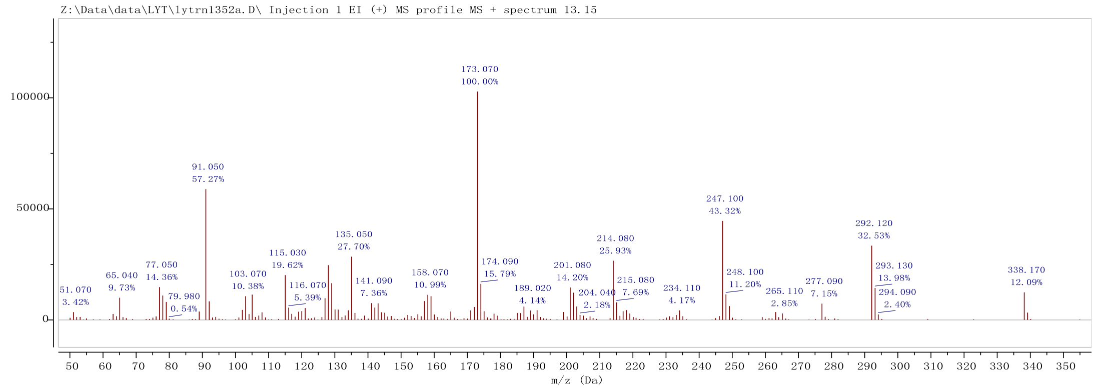

For the reaction 2,

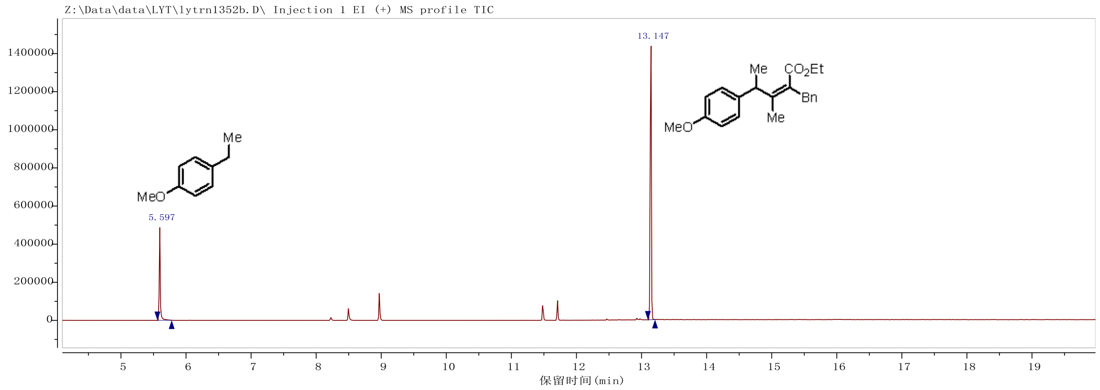

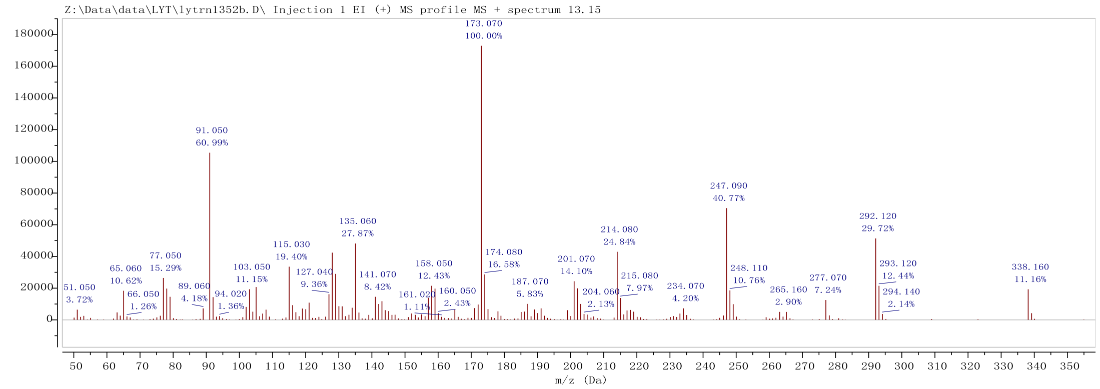

For the reaction 3

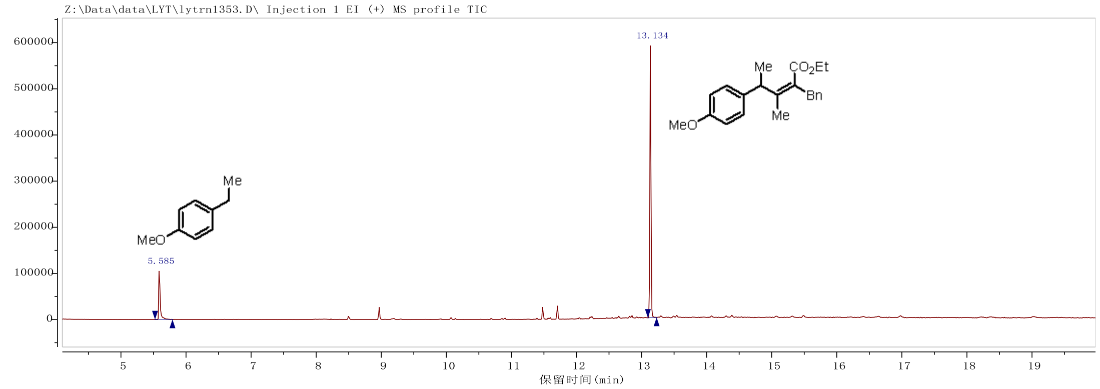

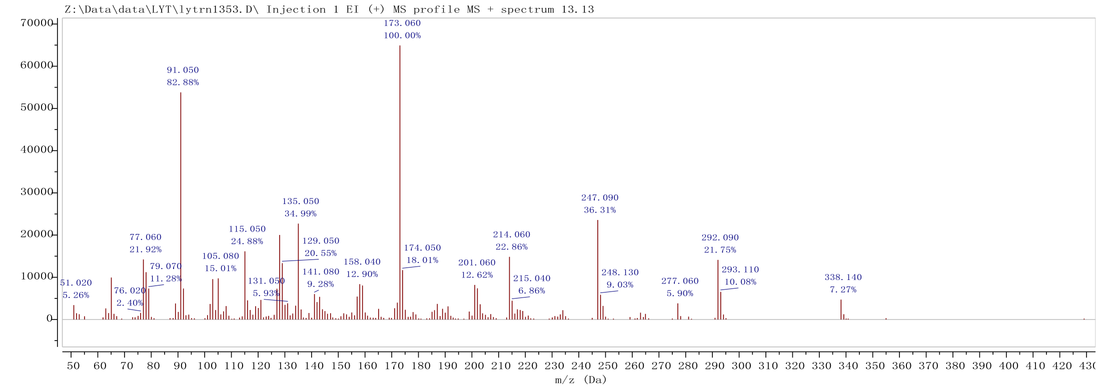

The remain time of the products is nearly the same, t=13.146, 13.147, 13.134. There are no isomers.

5.5.2 NMR analysis.

The comparation of the products' NMR spectrum from reaction 1-3 is as follows.

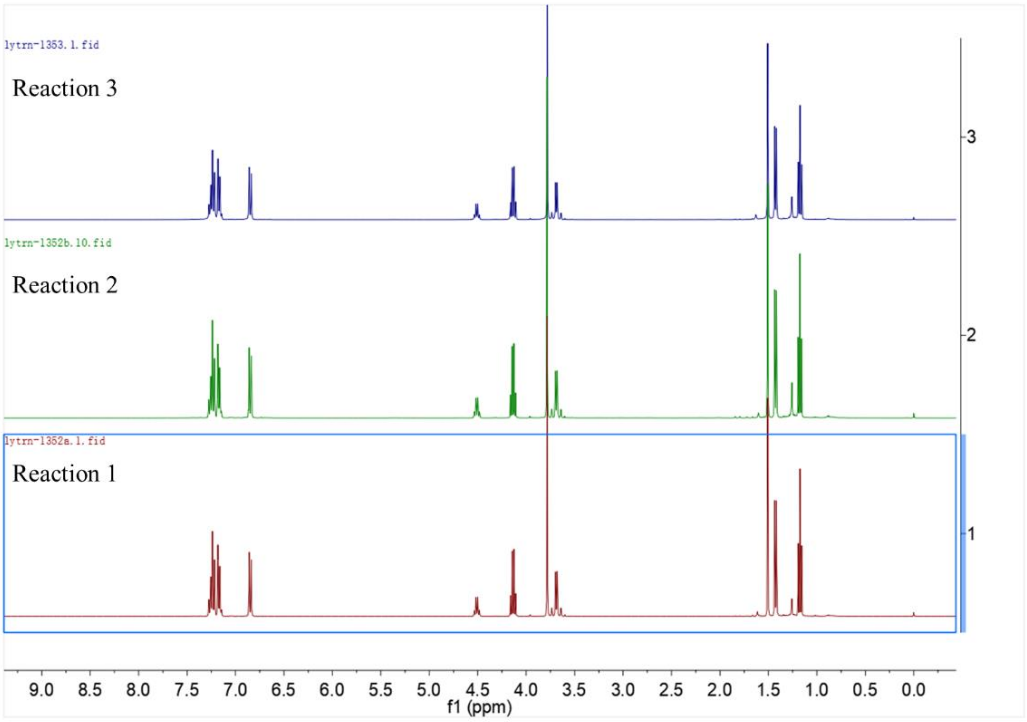

They have the same ^{1}H NMR spectrum.

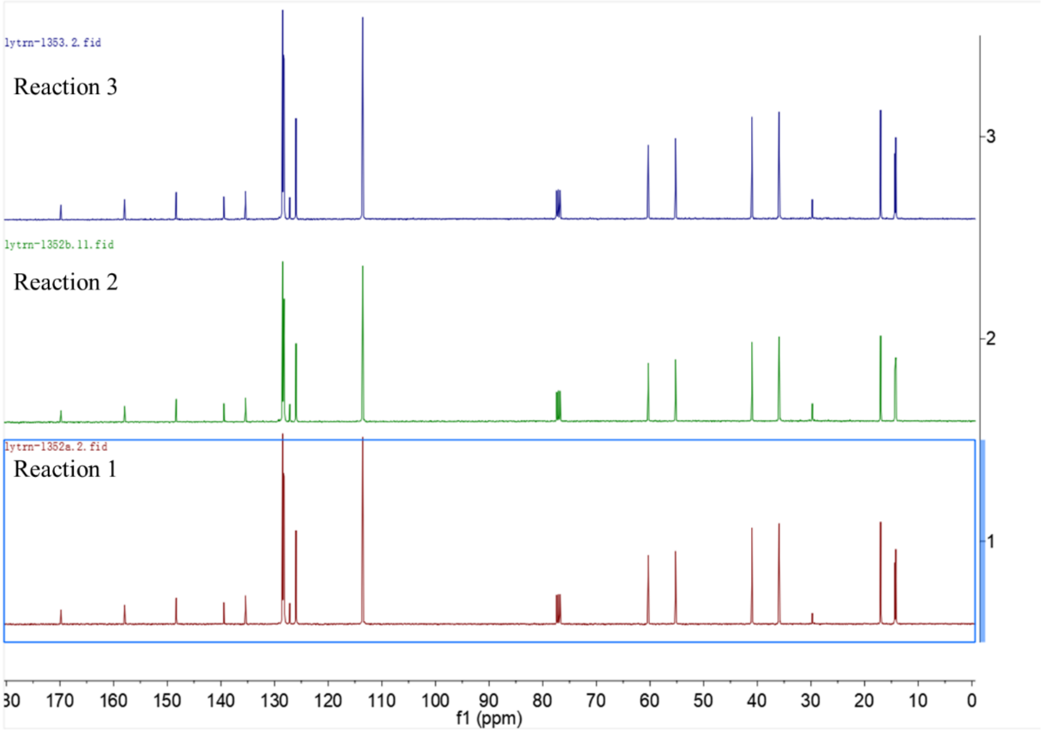

They have the same ^{13}C NMR spectrum.

H-H NOESY (product of reaction 1):

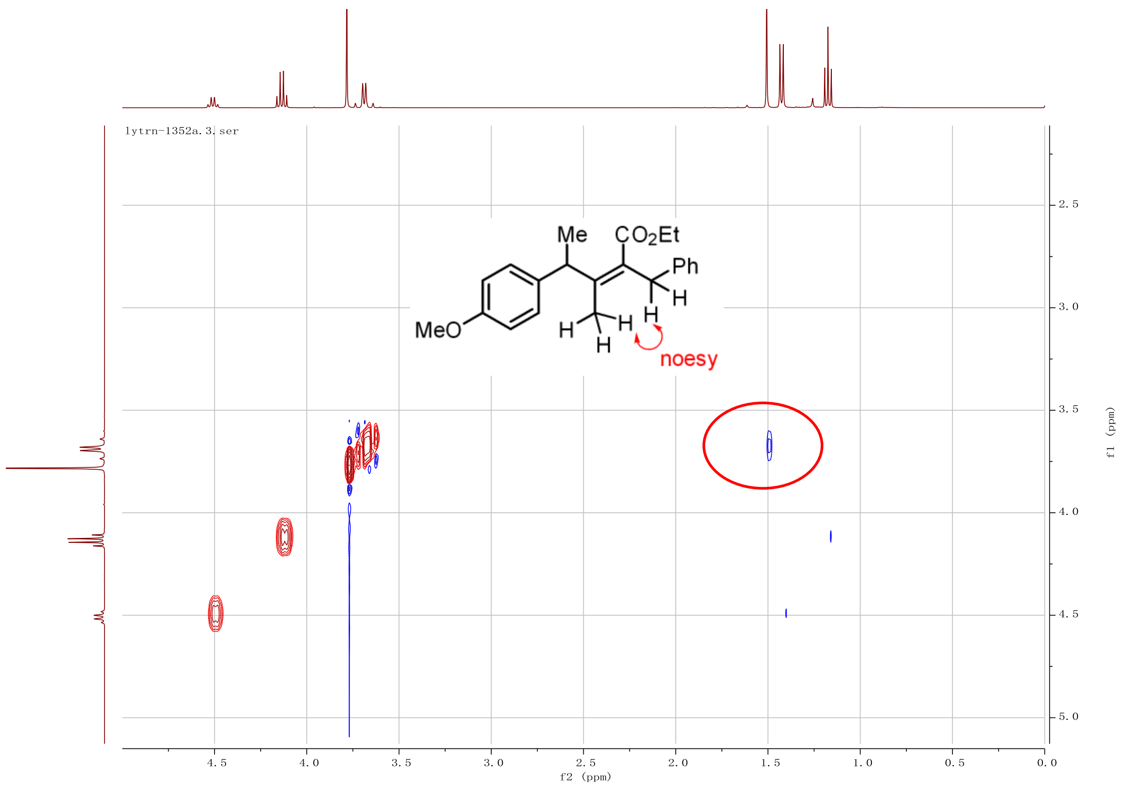

H-H NOESY (product of reaction 2):

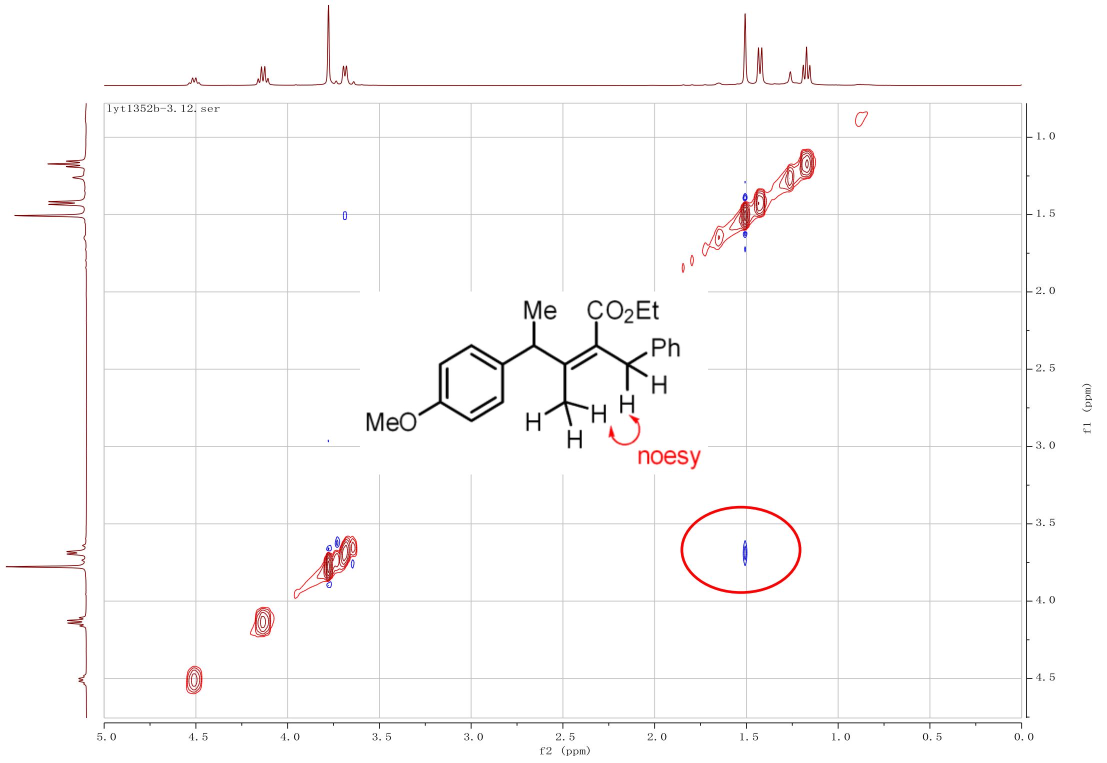

H-H NOESY (product of reaction 3):

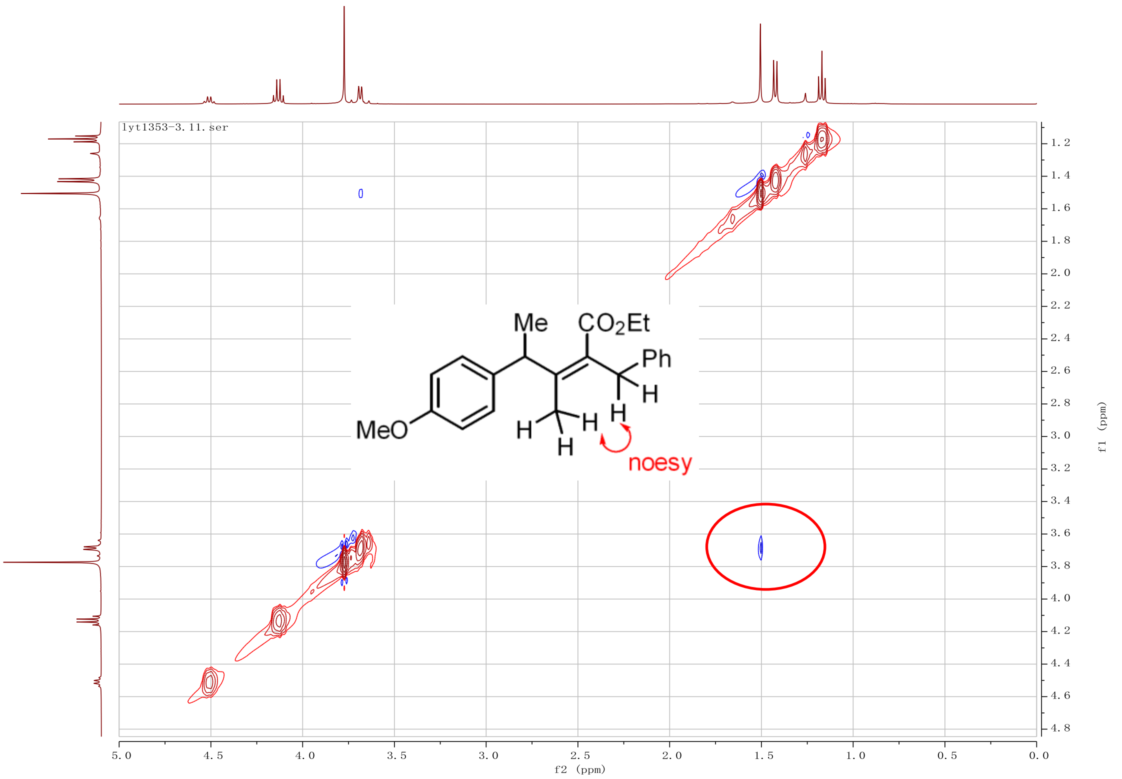

They have the same H-H NOESY spectrum.

6. NMR spectrum of substrates

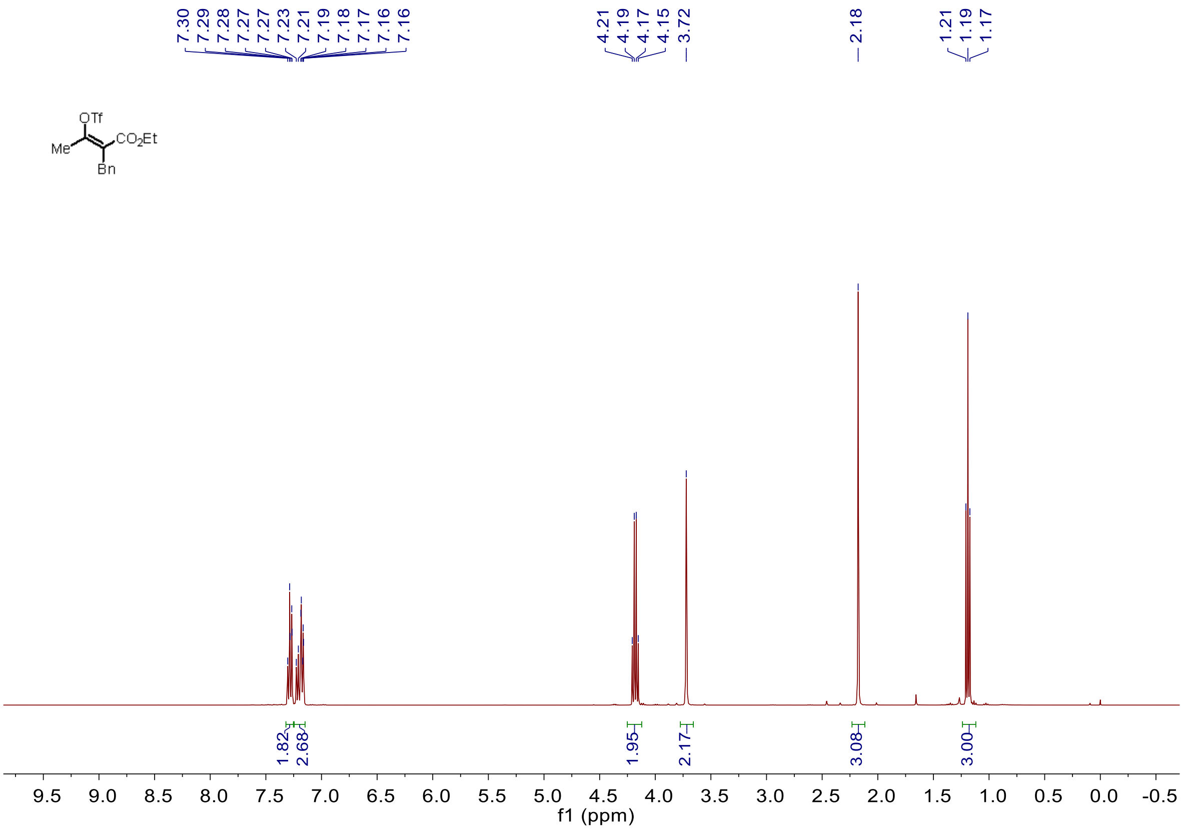

^{1}H NMR spectrum for compound **2a**

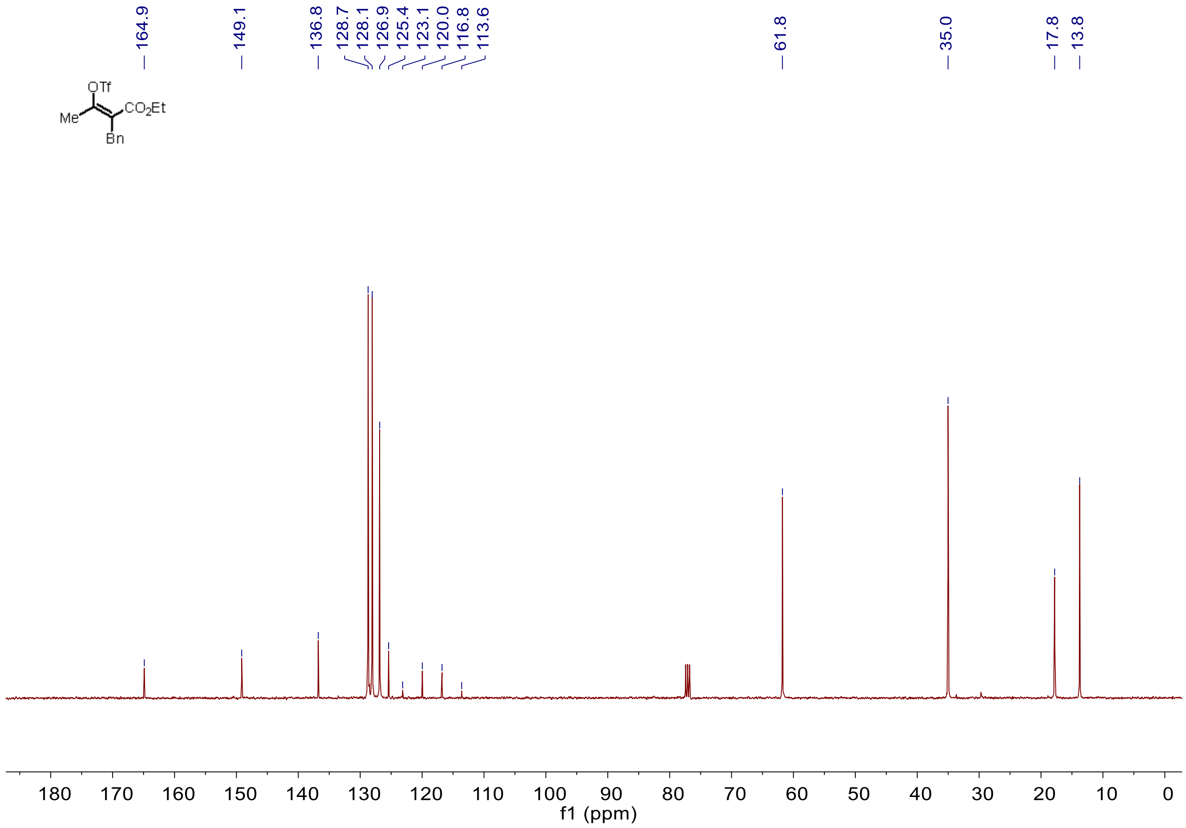

^{13}C NMR spectrum for compound **2a**

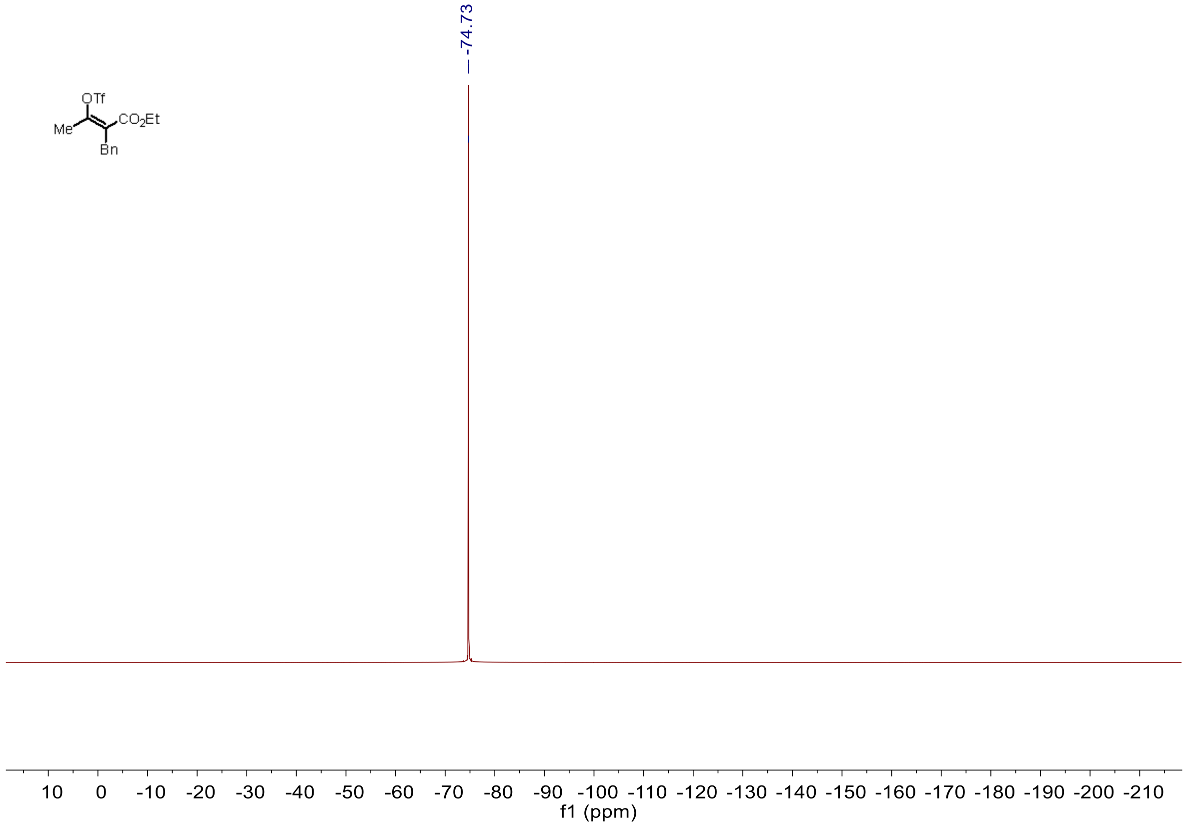

^{19}F NMR spectrum for compound **2a**

.

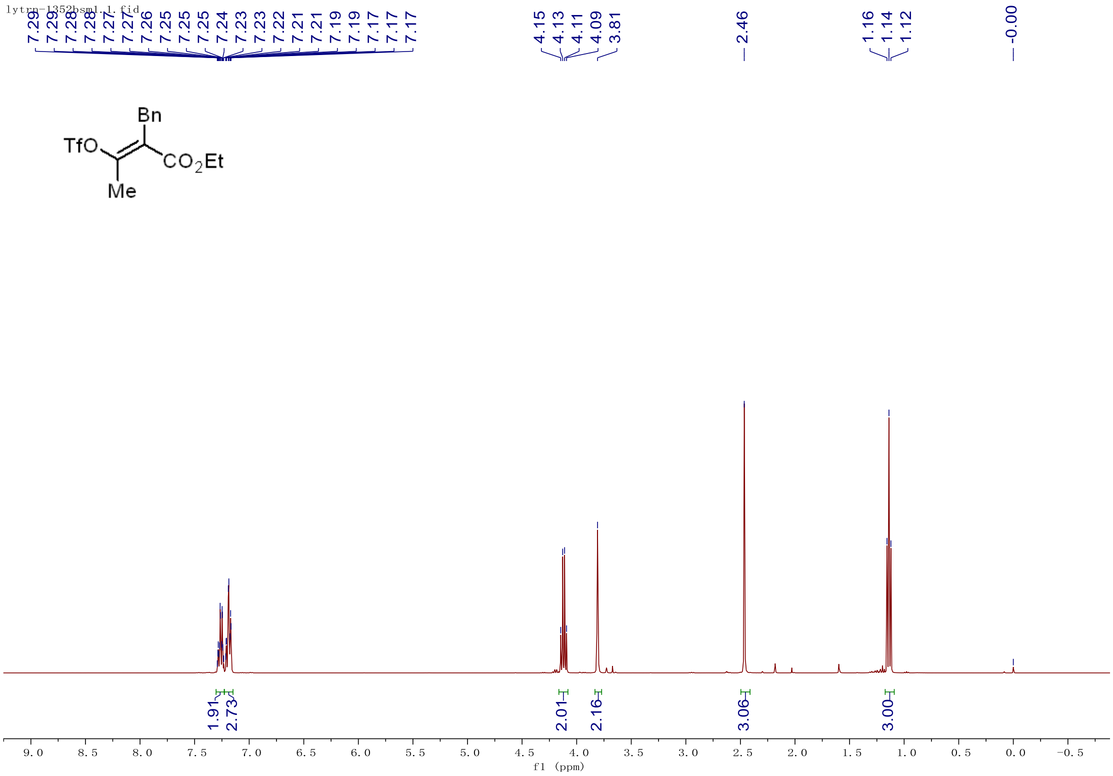

^{1}H NMR spectrum for compound **2a'**

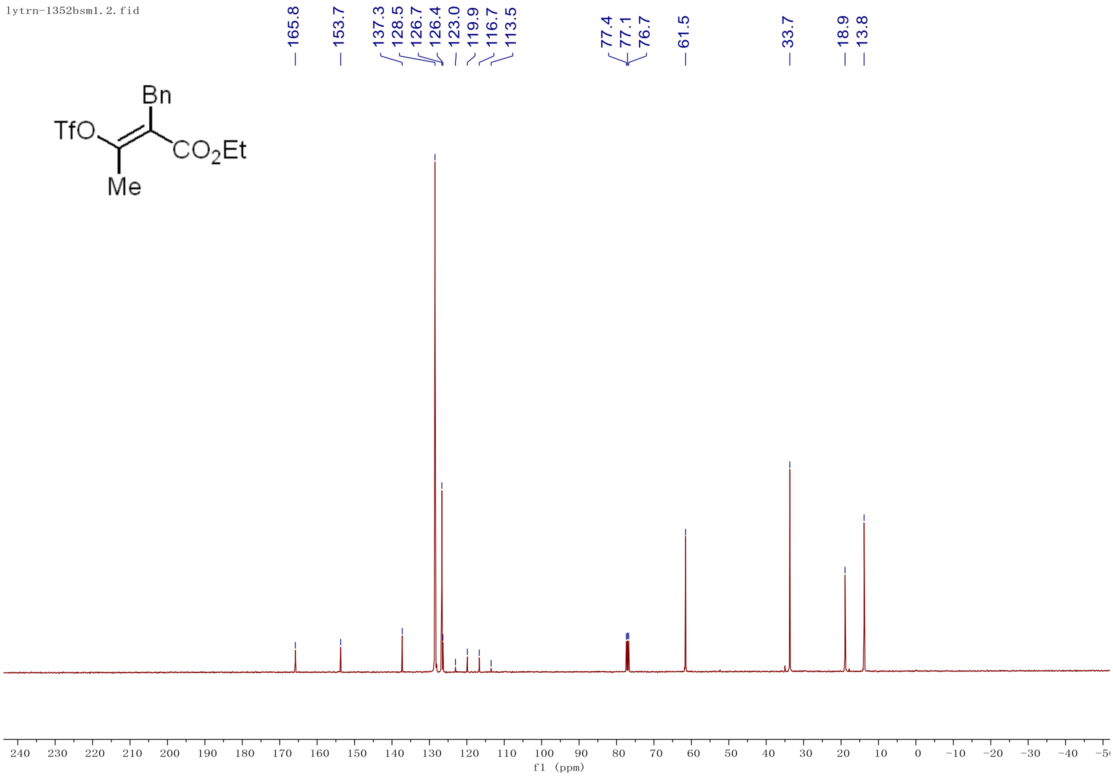

^{13}C NMR spectrum for compound **2a'**

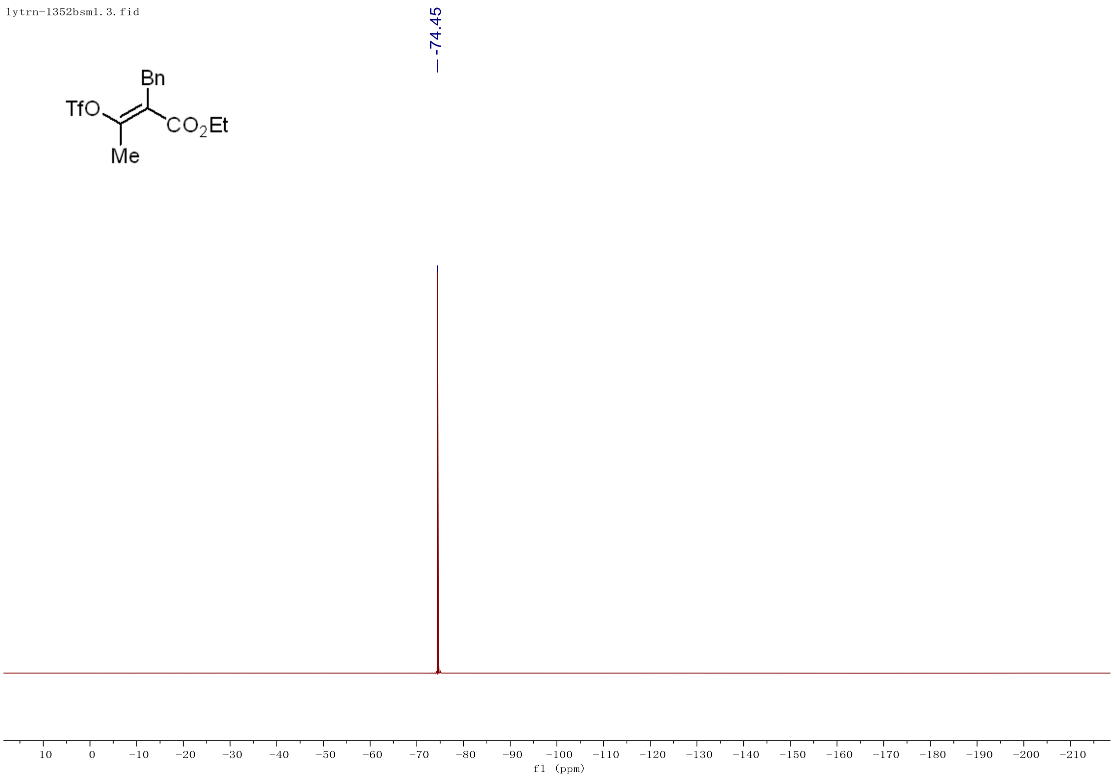

^{19}F NMR spectrum for compound **2a'**

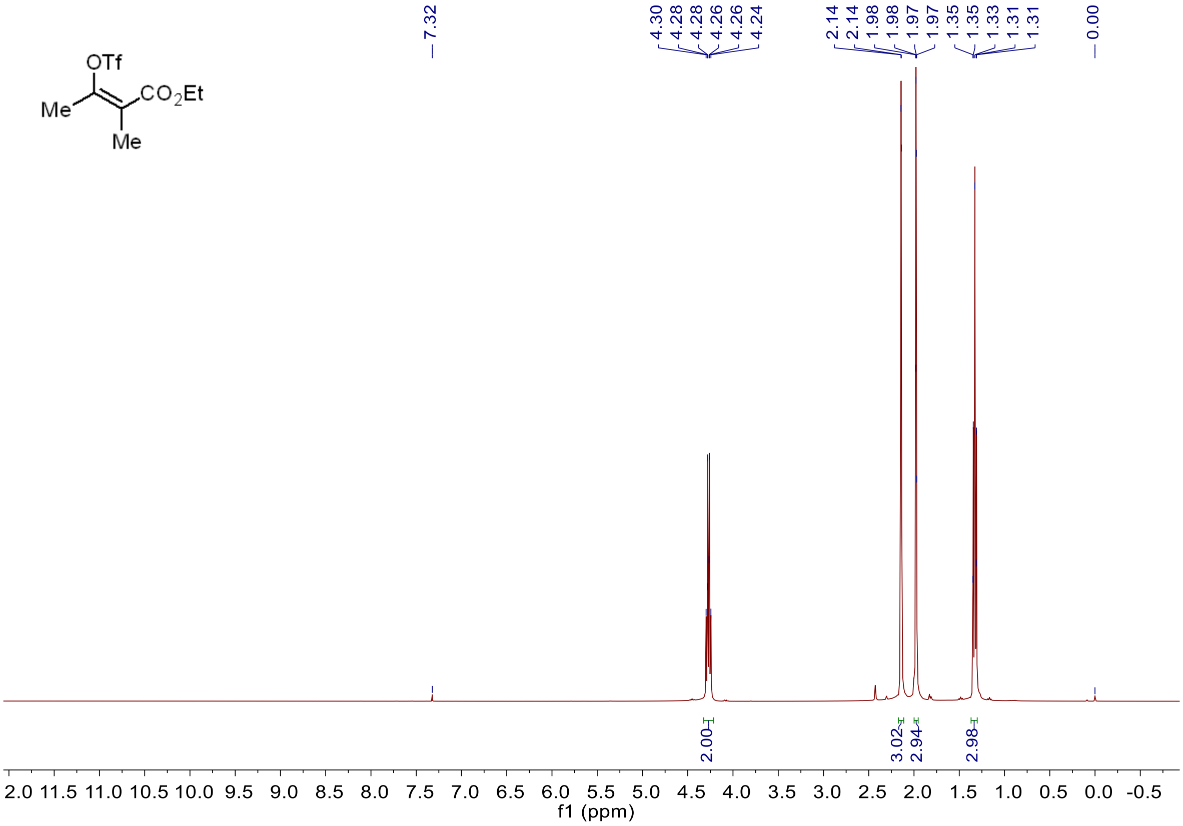

^{1}H NMR spectrum for compound **2b**

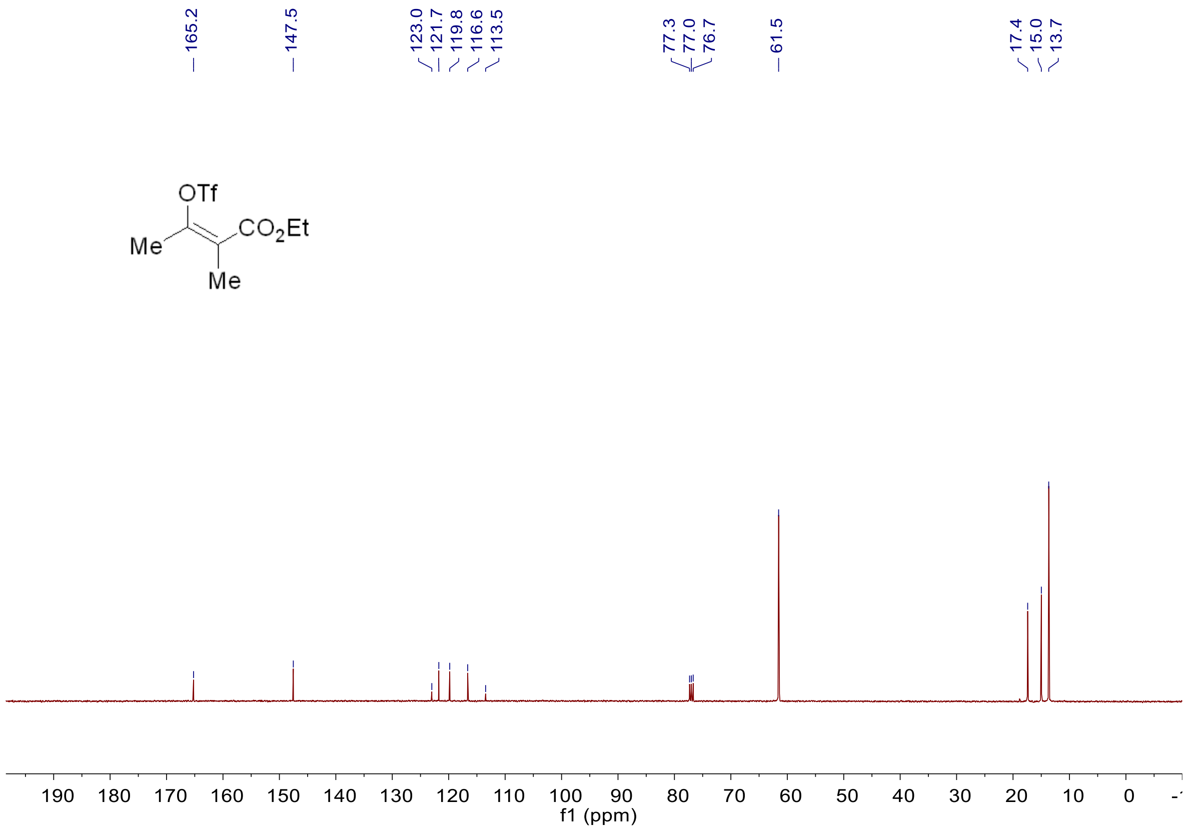

^{13}C NMR spectrum for compound **2b**

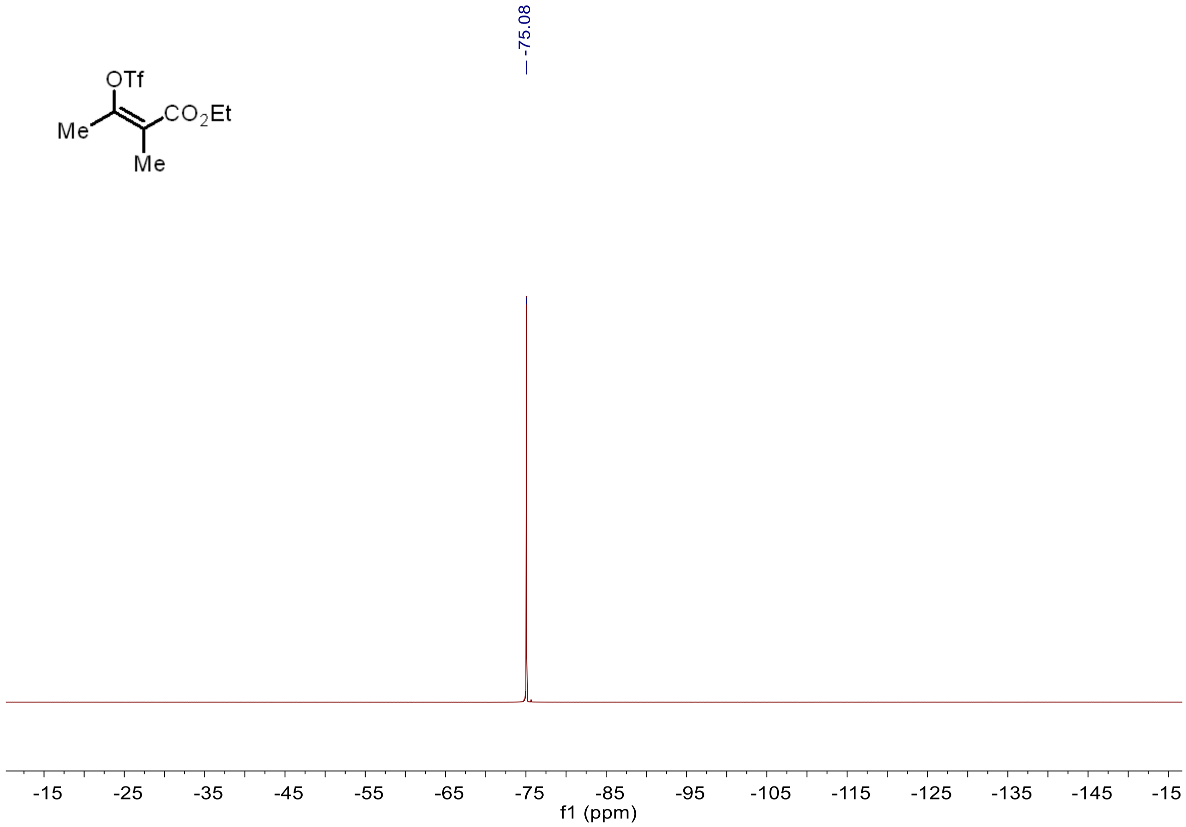

^{19}F NMR spectrum for compound **2b**

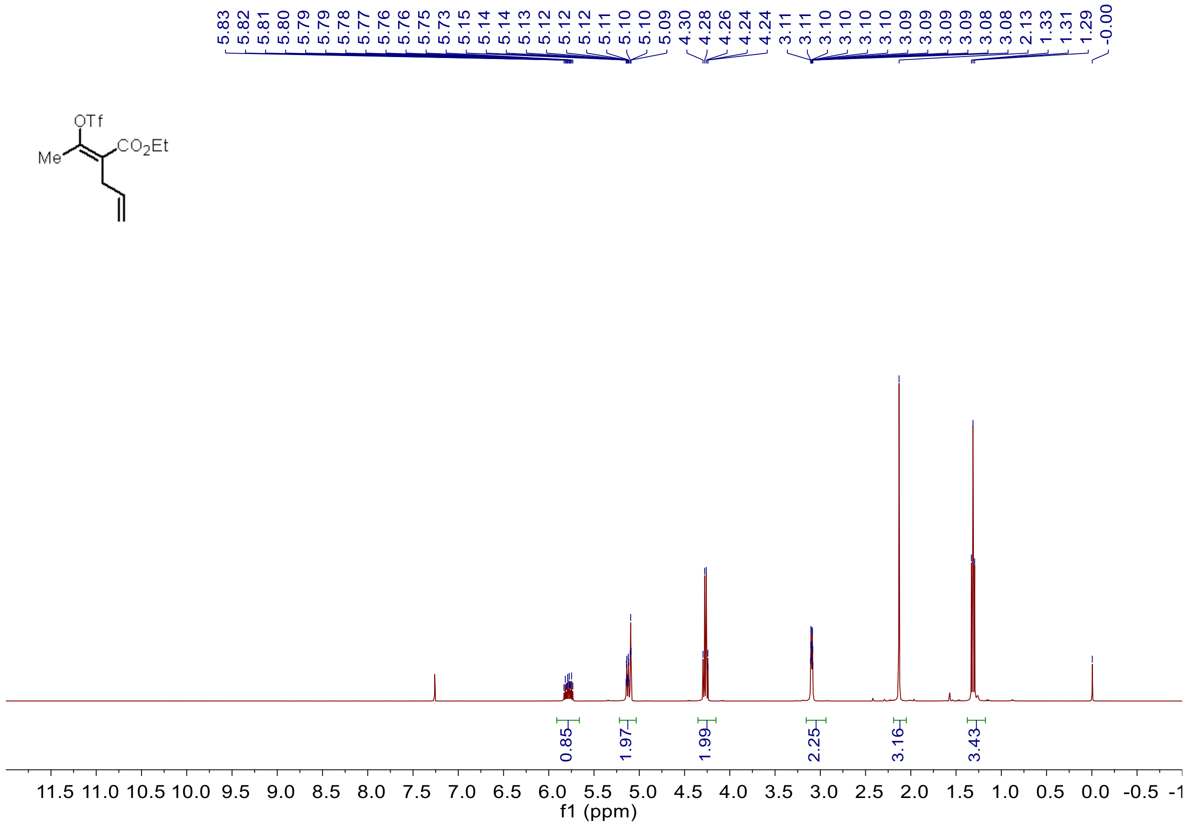

^{1}H NMR spectrum for compound **2c**

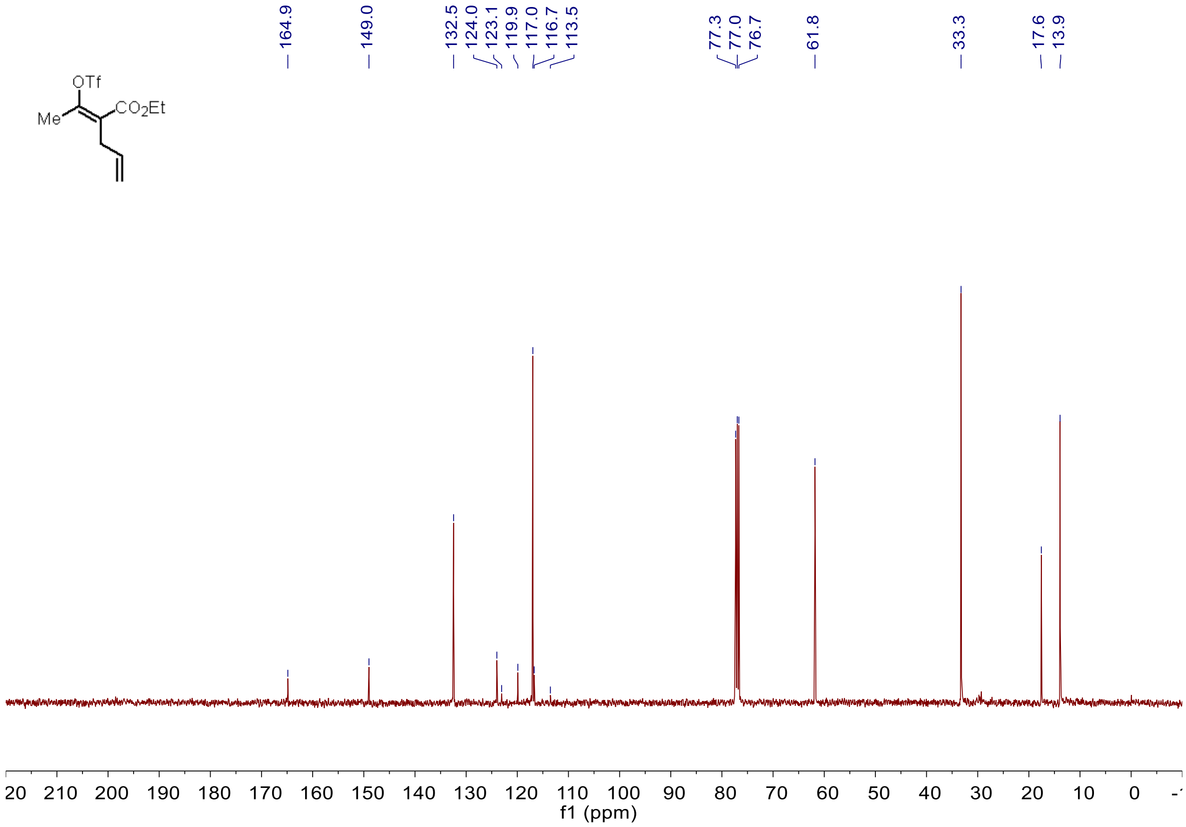

^{13}C NMR spectrum for compound **2c**

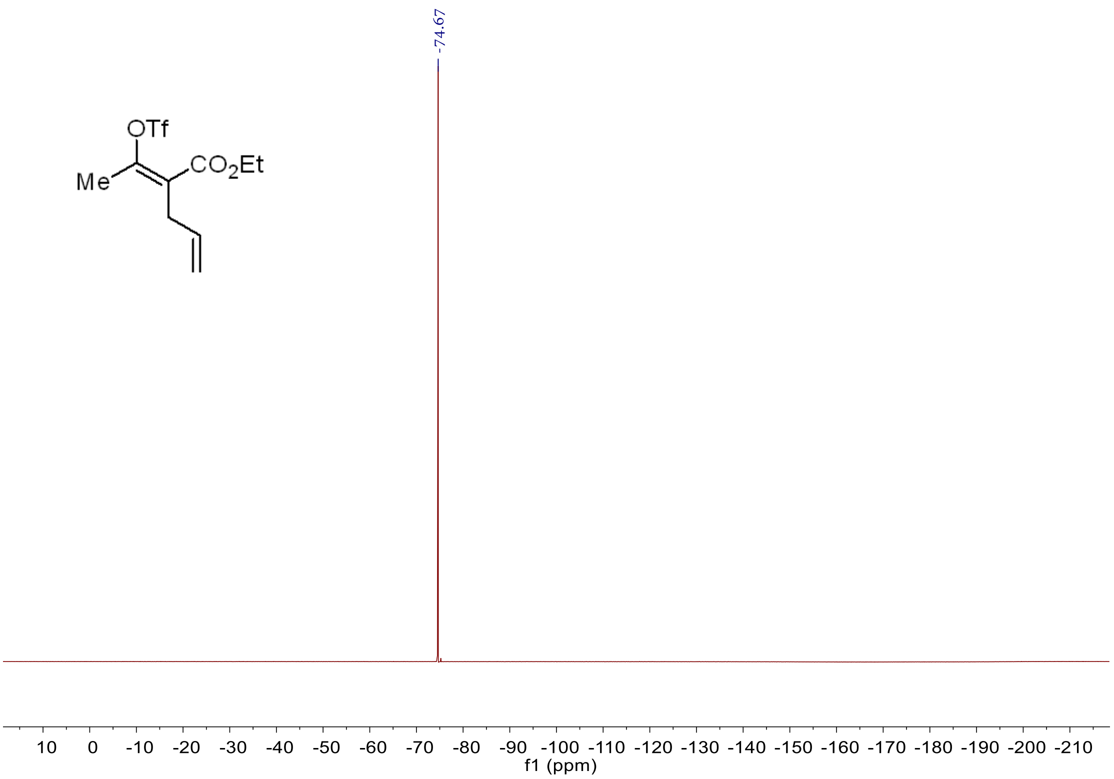

^{19}F NMR spectrum for compound **2c**

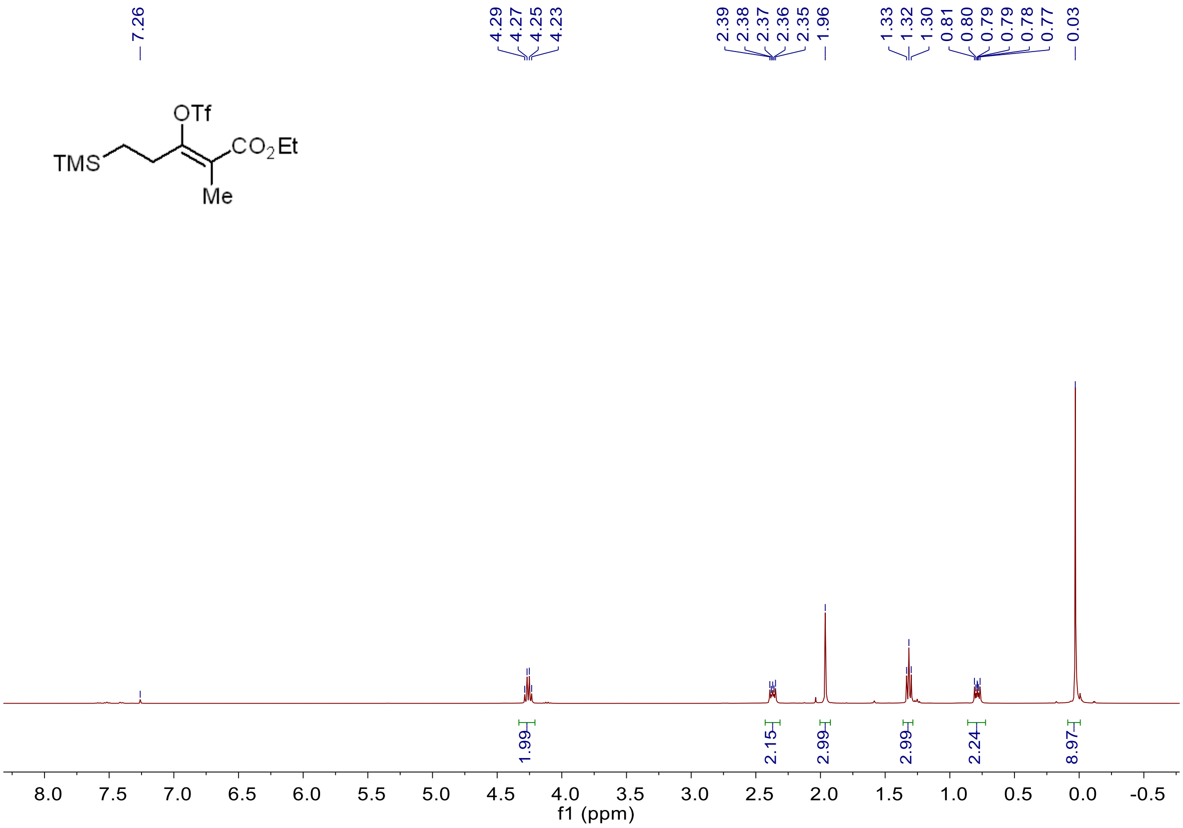

^{1}H NMR spectrum for compound **2d**

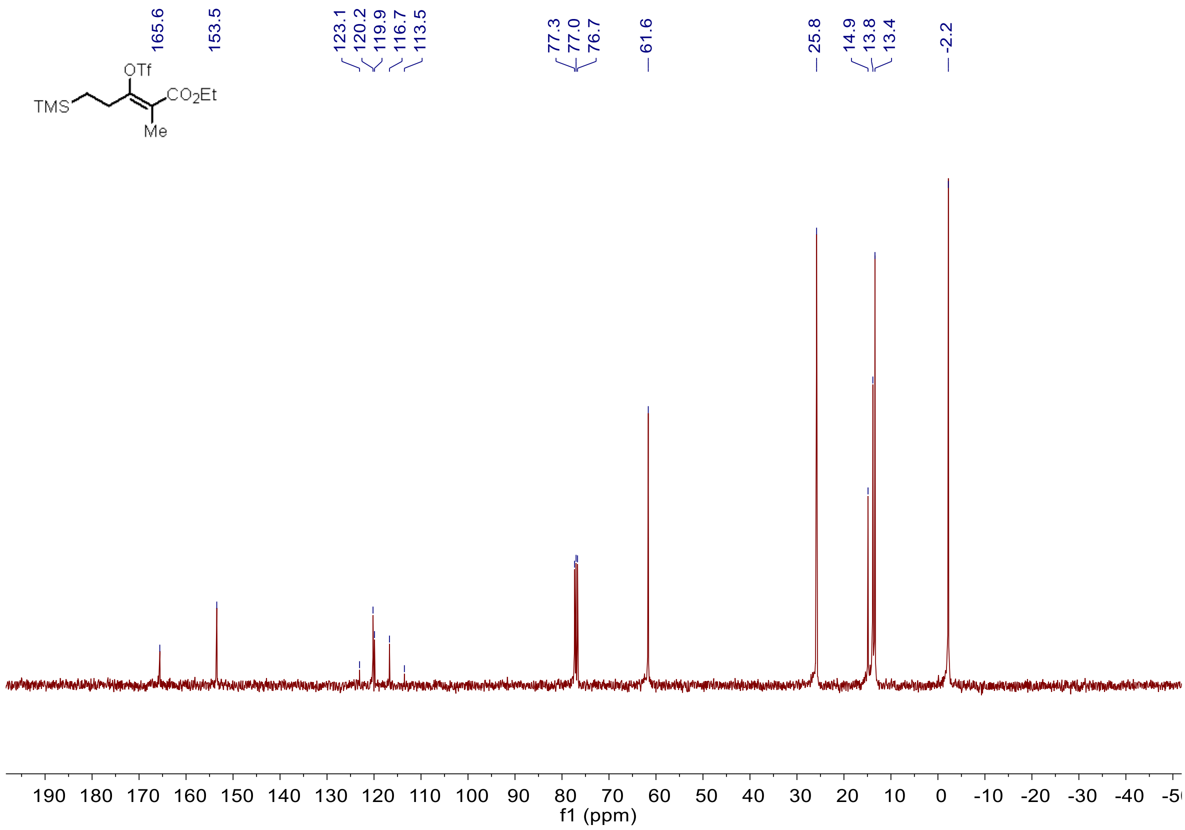

^{13}C NMR spectrum for compound **2d**

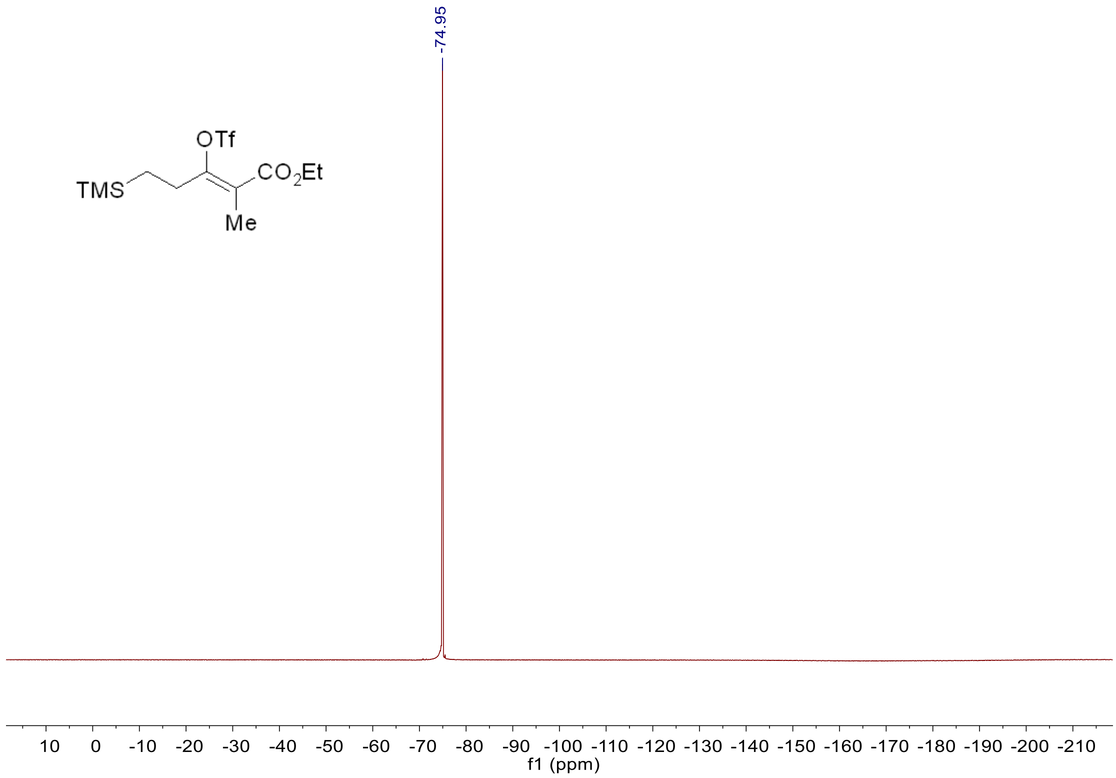

^{19}F NMR spectrum for compound **2d**

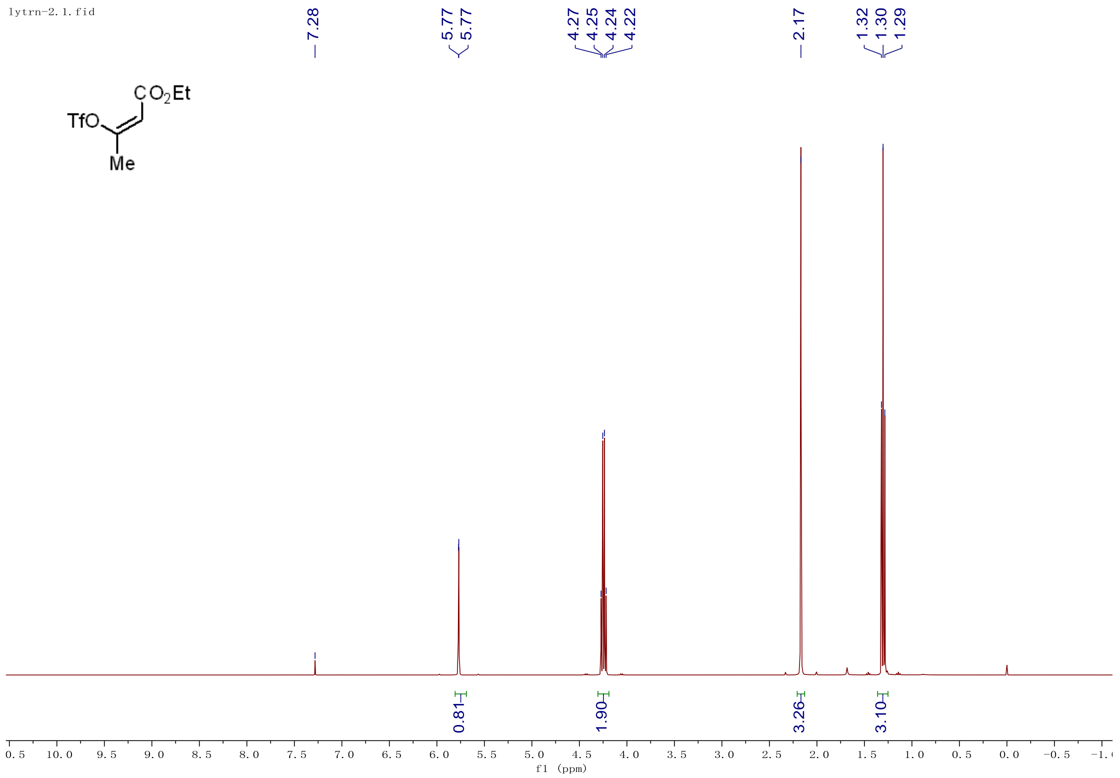

^{1}H NMR spectrum for compound **2e**

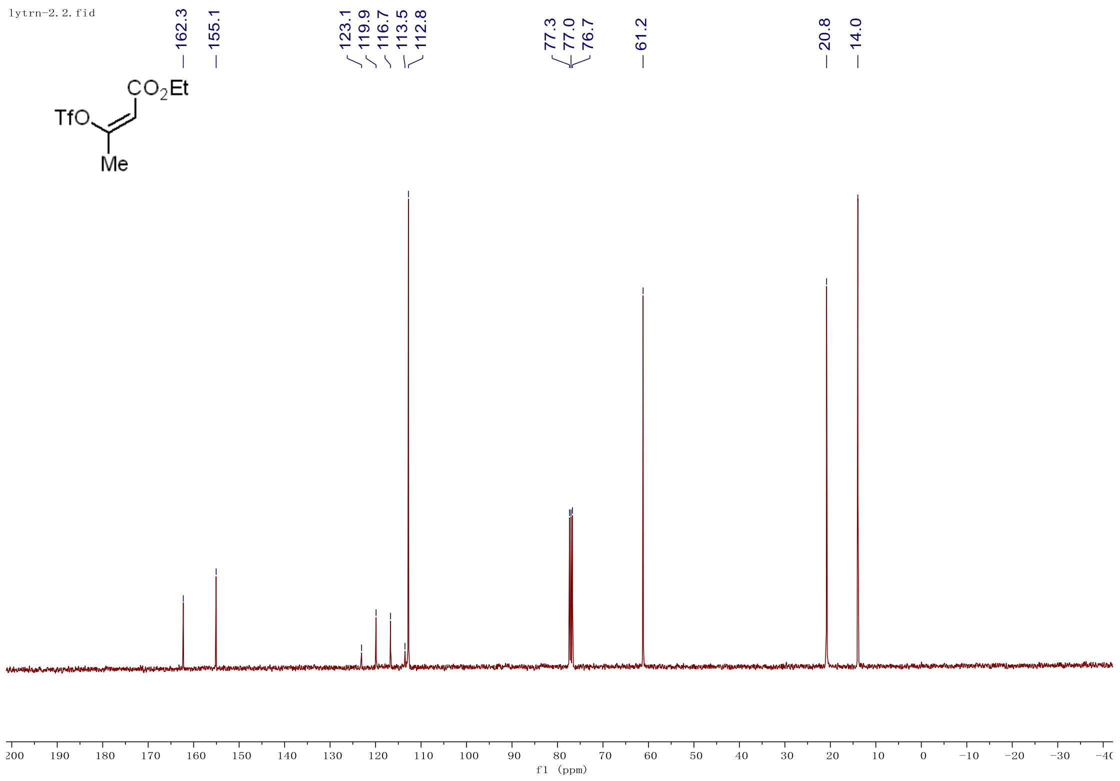

^{13}C NMR spectrum for compound **2e**

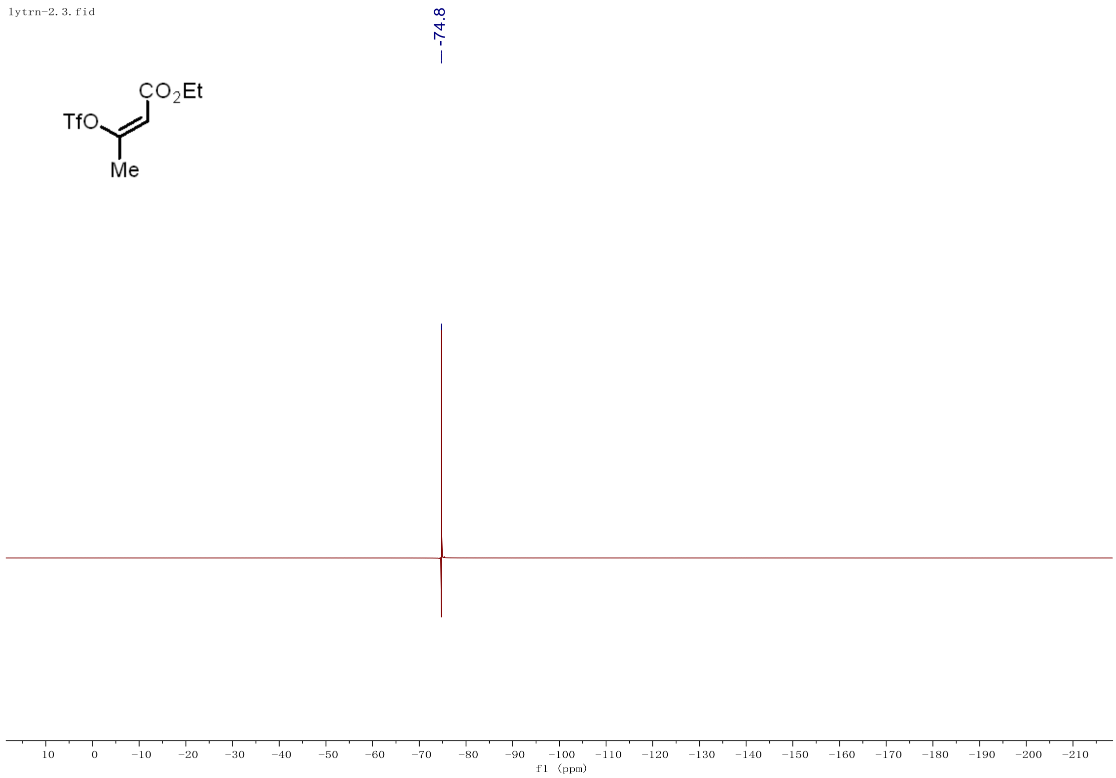

^{19}F NMR spectrum for compound **2e**

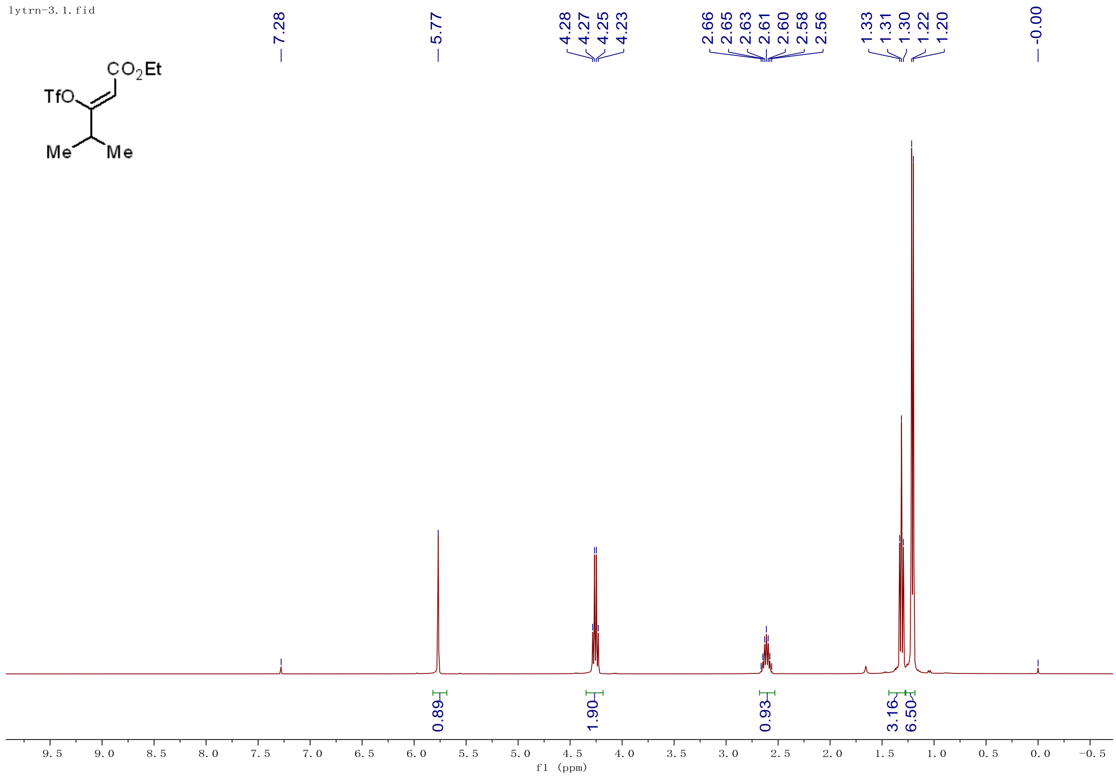

^{1}H NMR spectrum for compound **2f**

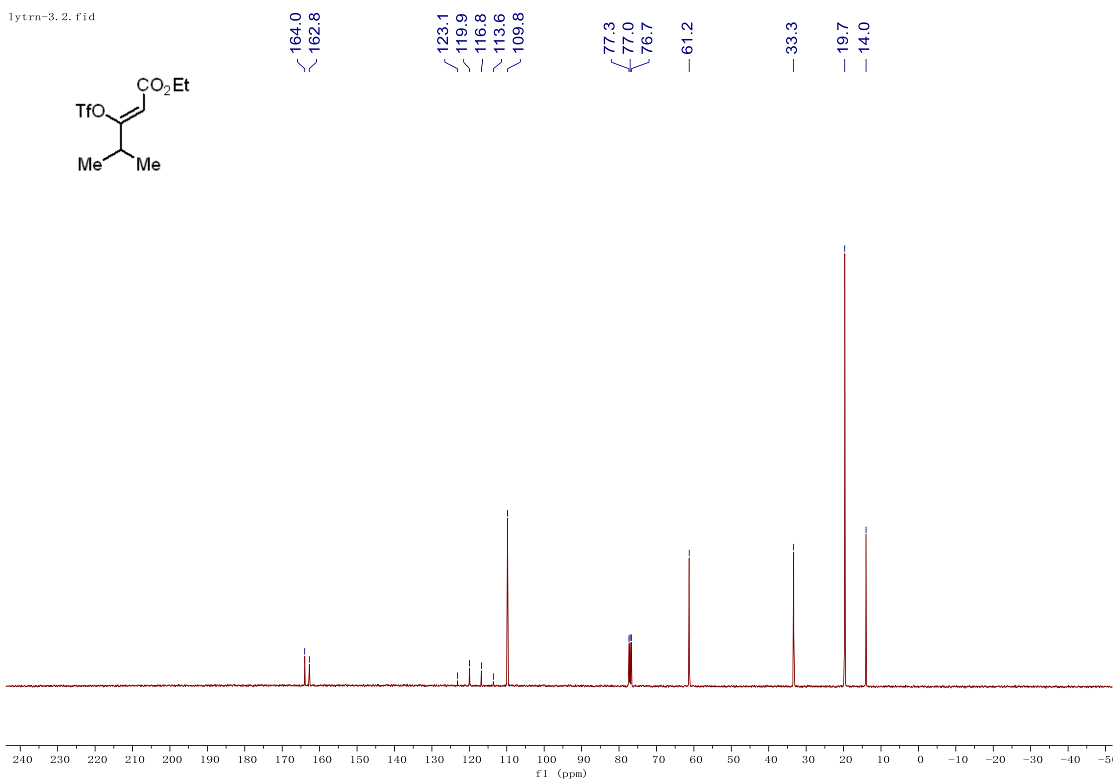

^{13}C NMR spectrum for compound **2f**

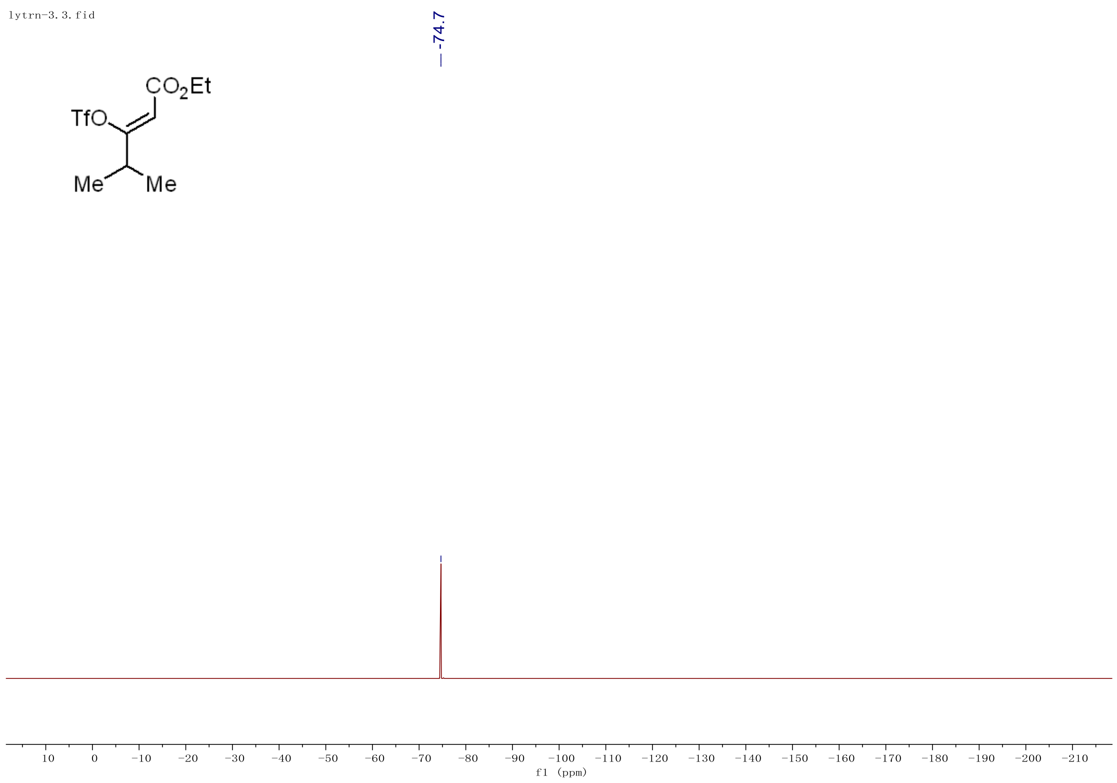

^{19}F NMR spectrum for compound **2f**

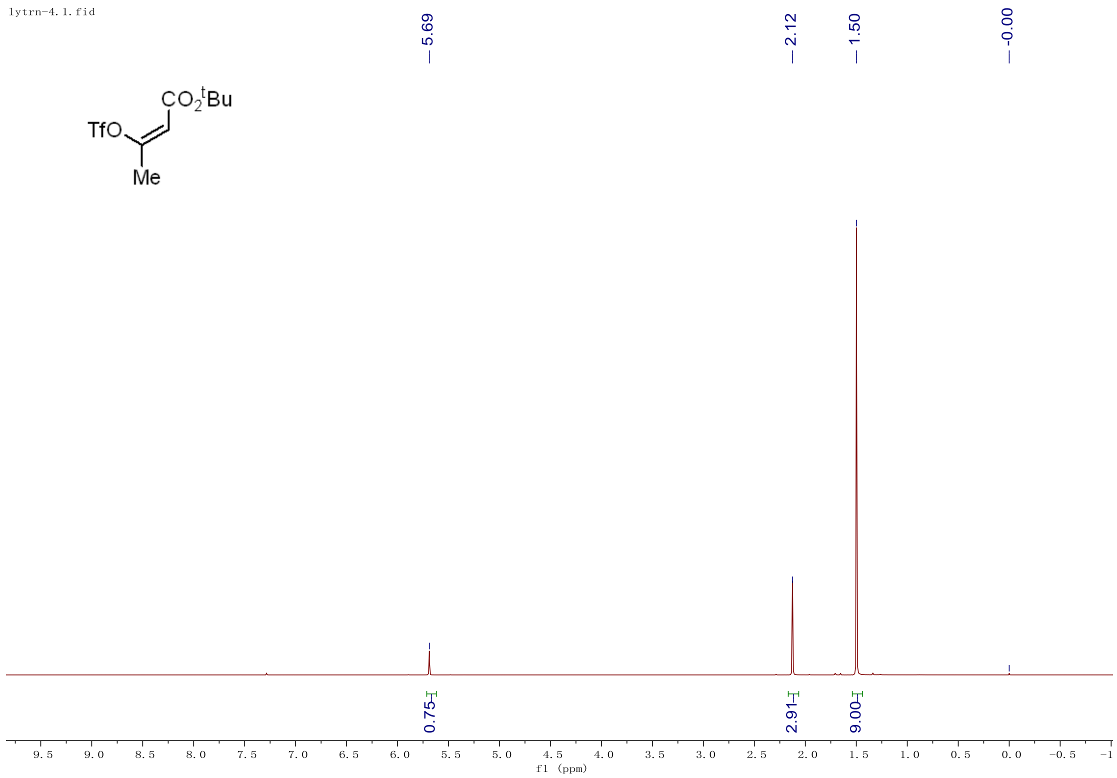

^{1}H NMR spectrum for compound **2g**

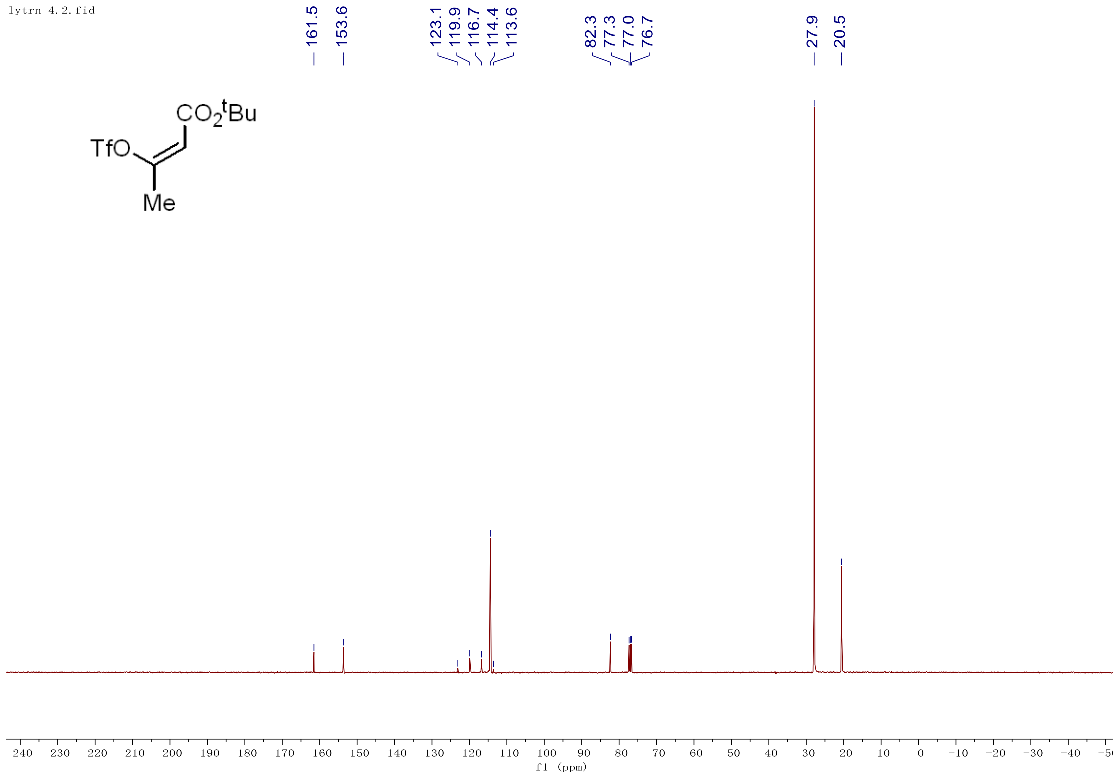

^{13}C NMR spectrum for compound **2g**

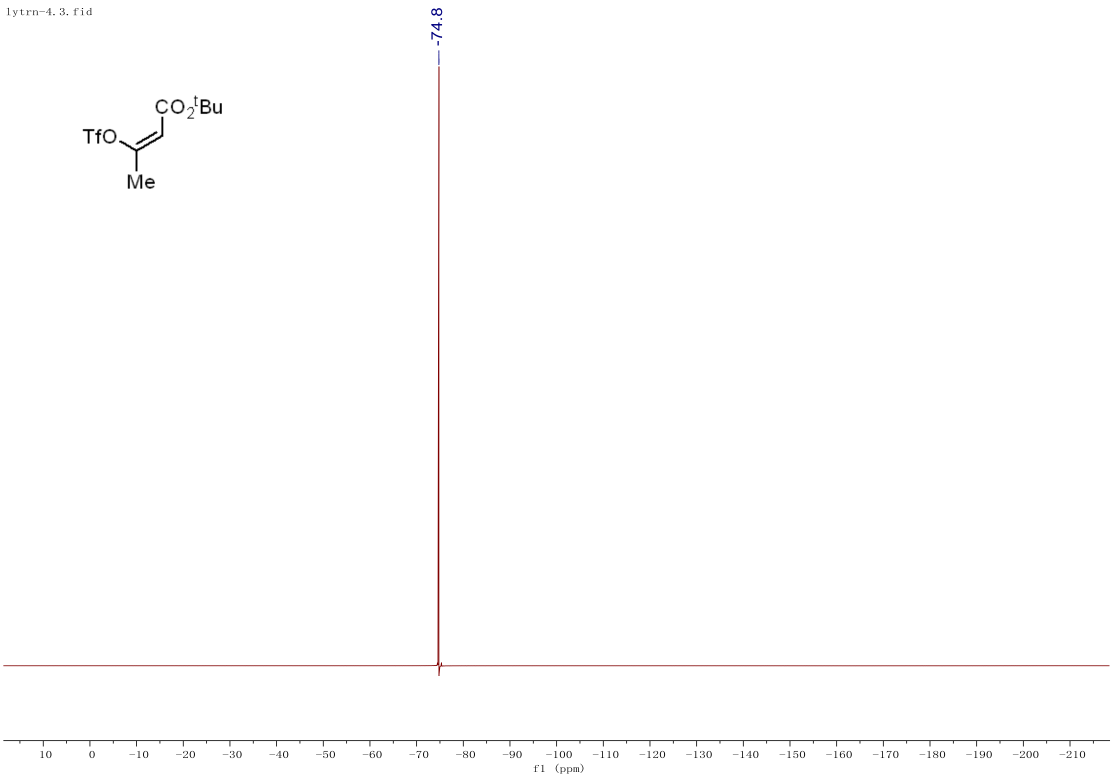

^{19}F NMR spectrum for compound **2g**

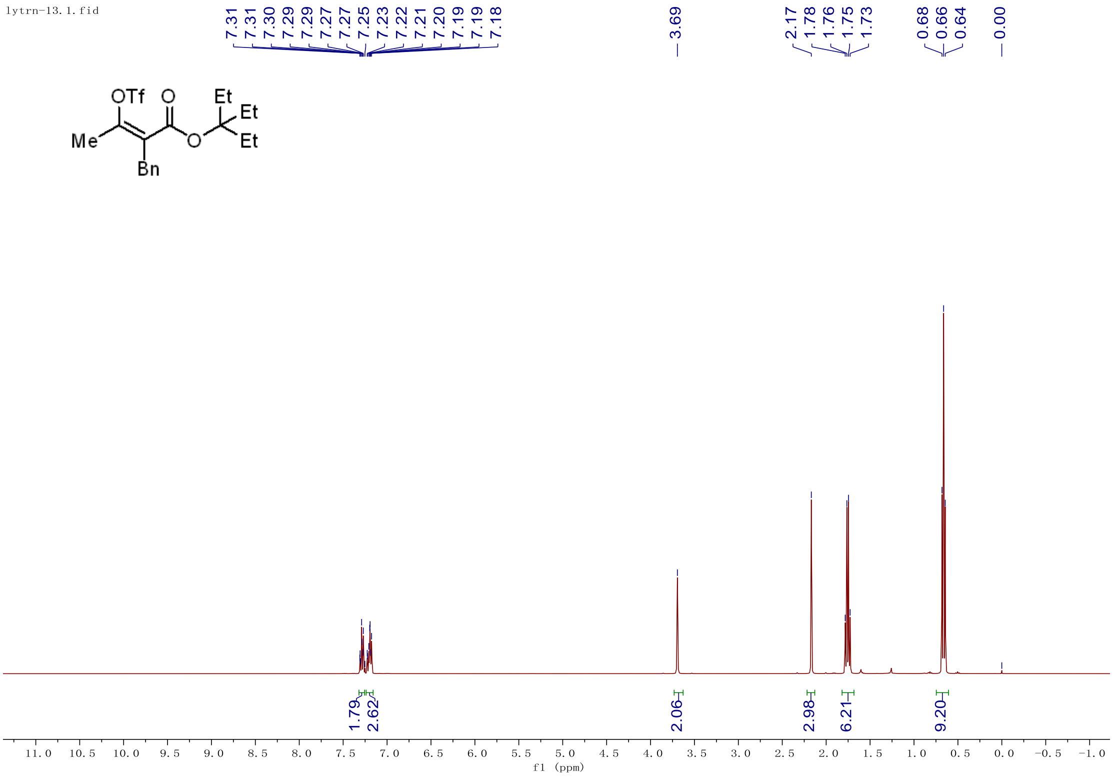

^{1}H NMR spectrum for compound **2h**

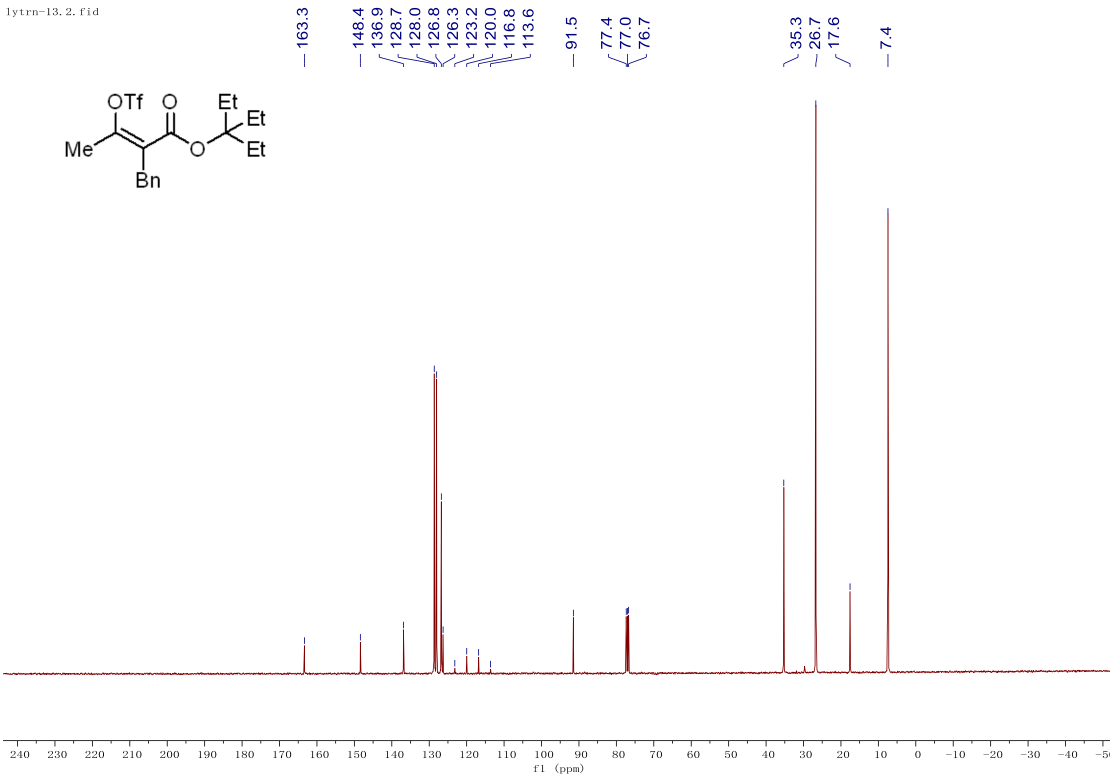

^{13}C NMR spectrum for compound **2h**

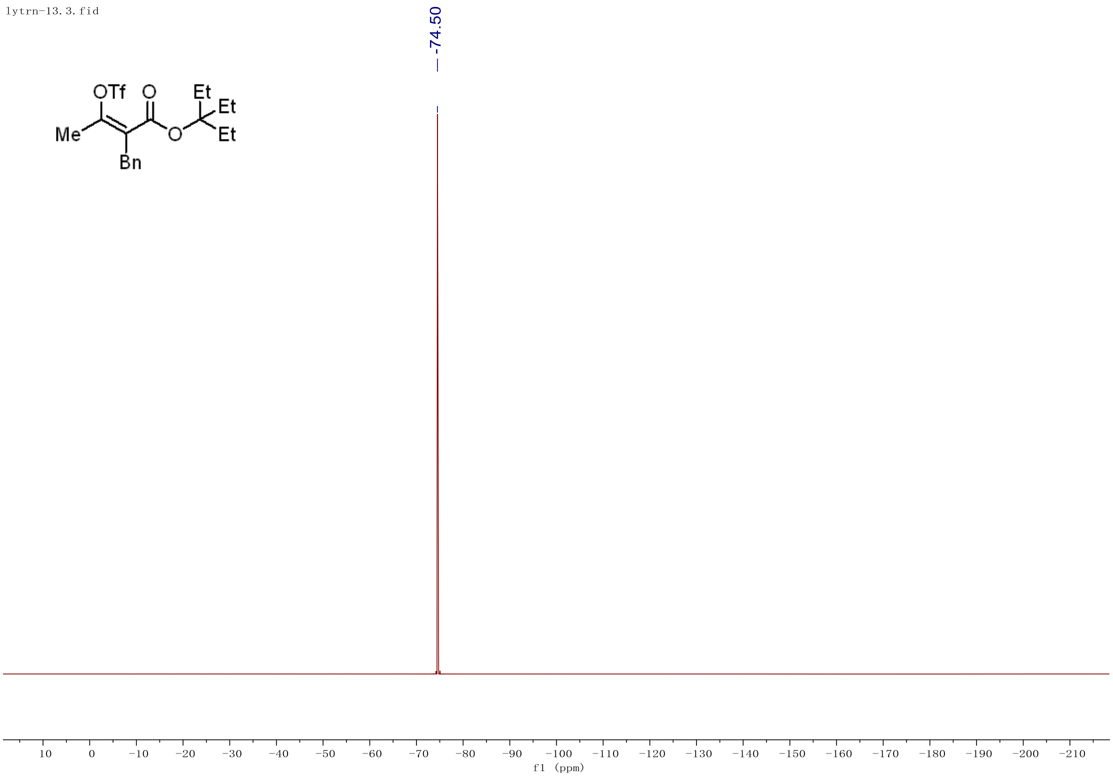

^{19}F NMR spectrum for compound **2h**

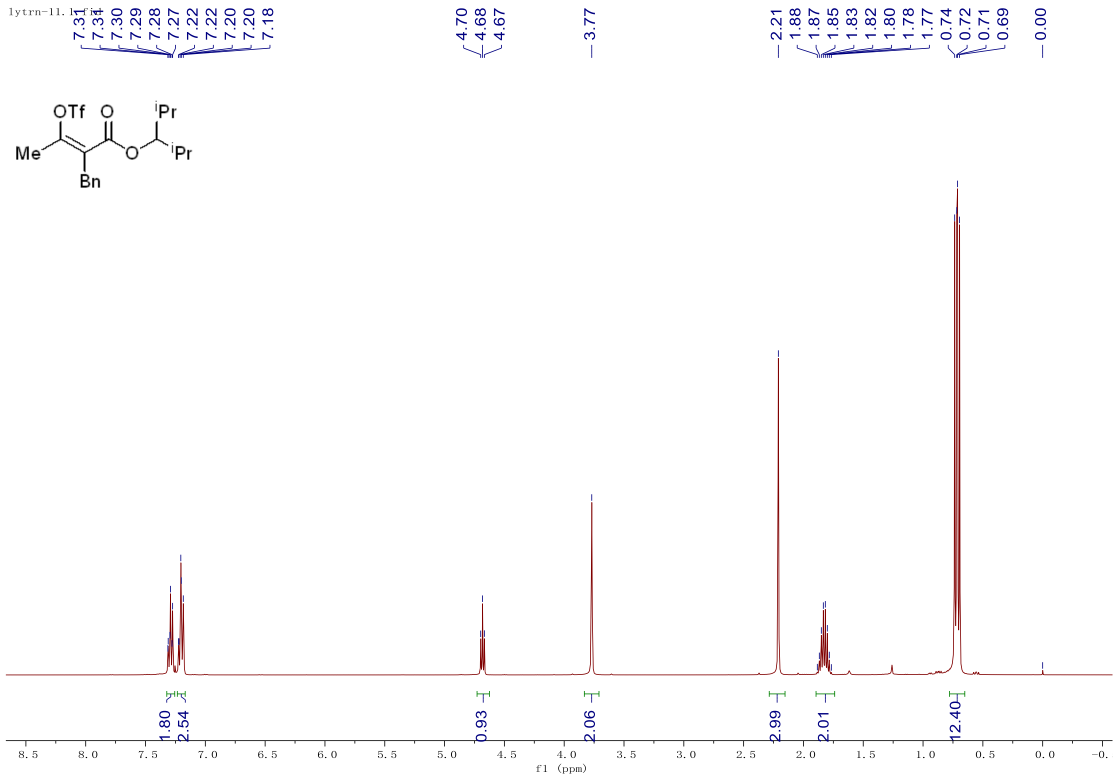

^{1}H NMR spectrum for compound **2i**

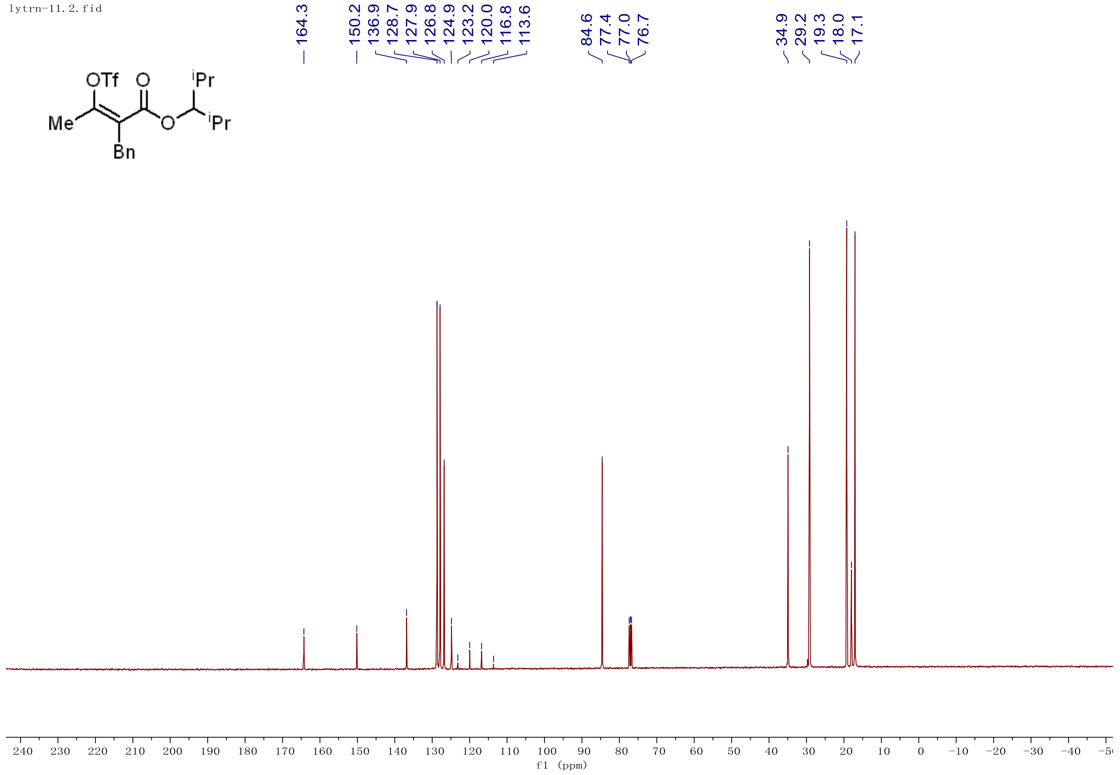

^{13}C NMR spectrum for compound **2i**

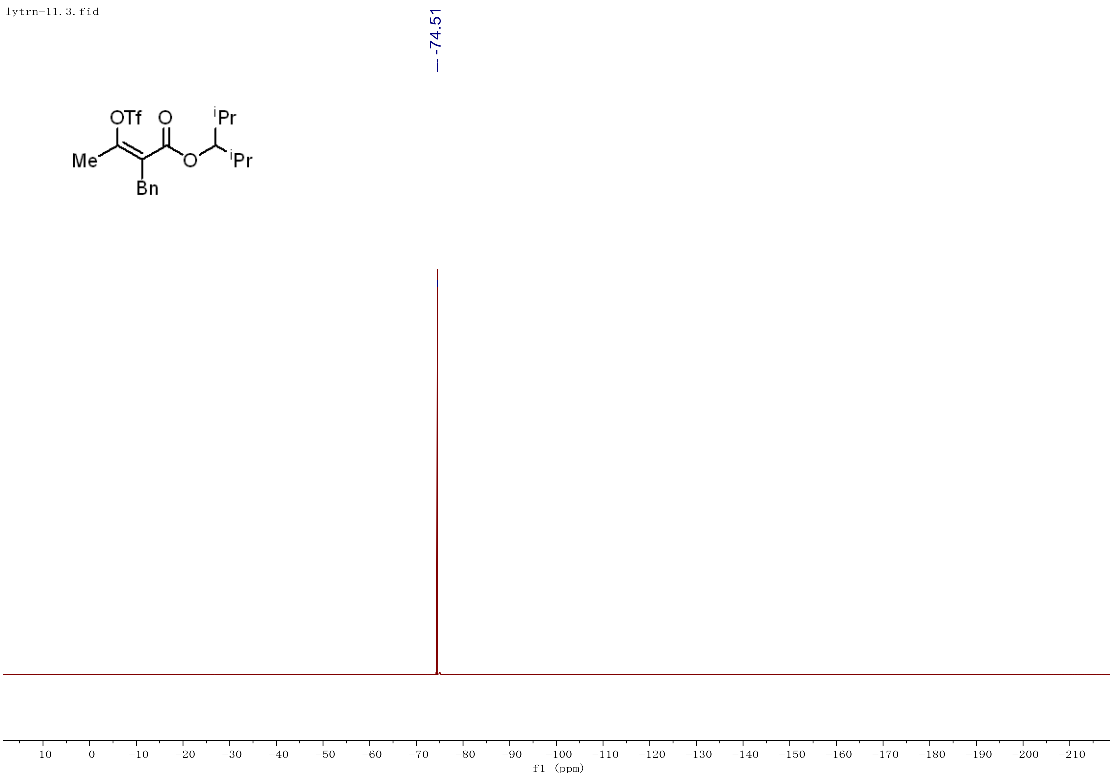

^{19}F NMR spectrum for compound **2i**

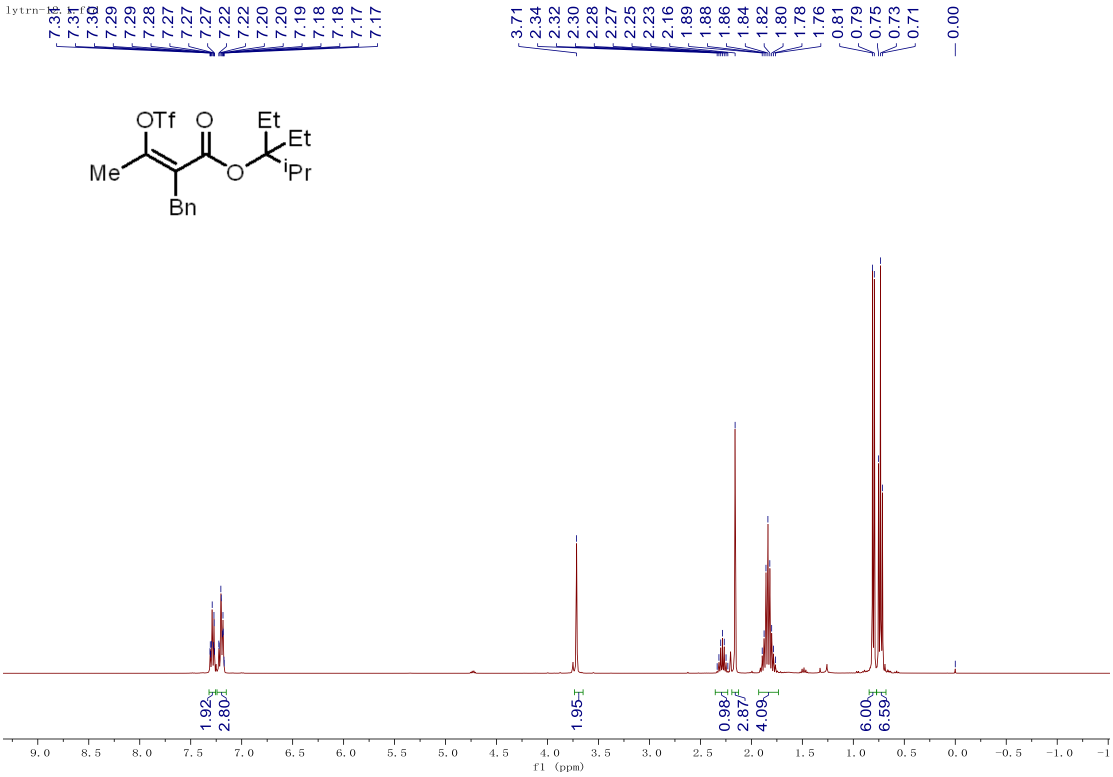

^{1}H NMR spectrum for compound **2j**

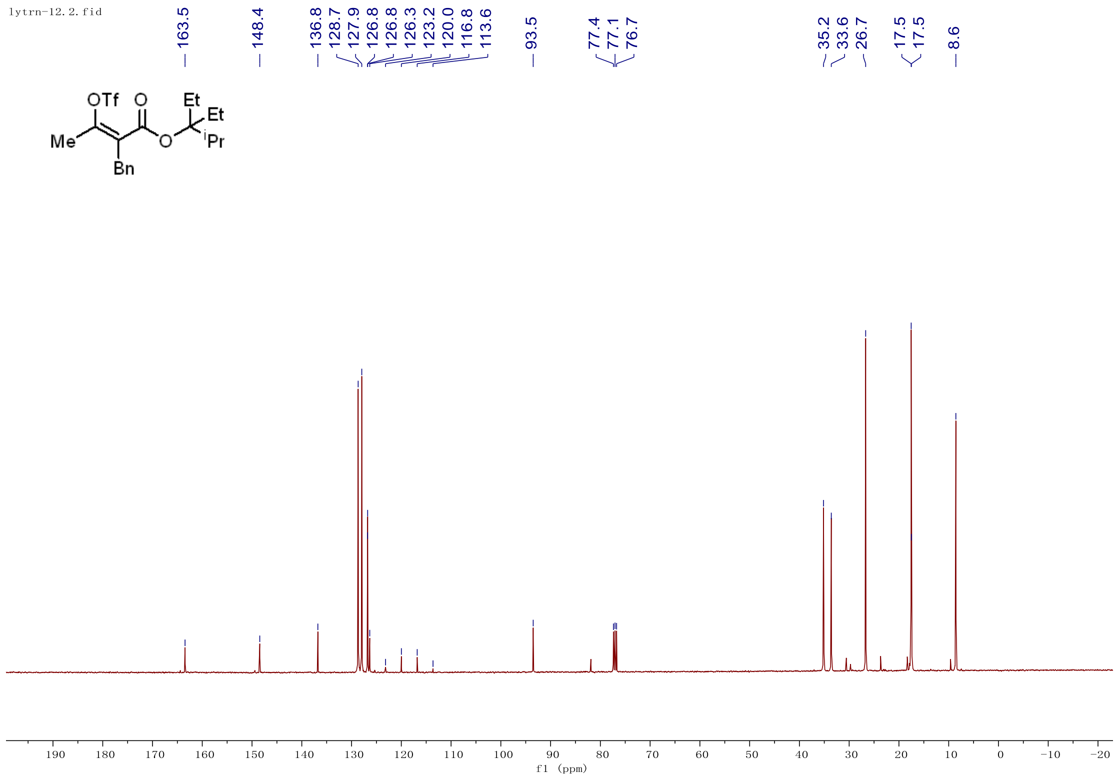

^{13}C NMR spectrum for compound **2j**

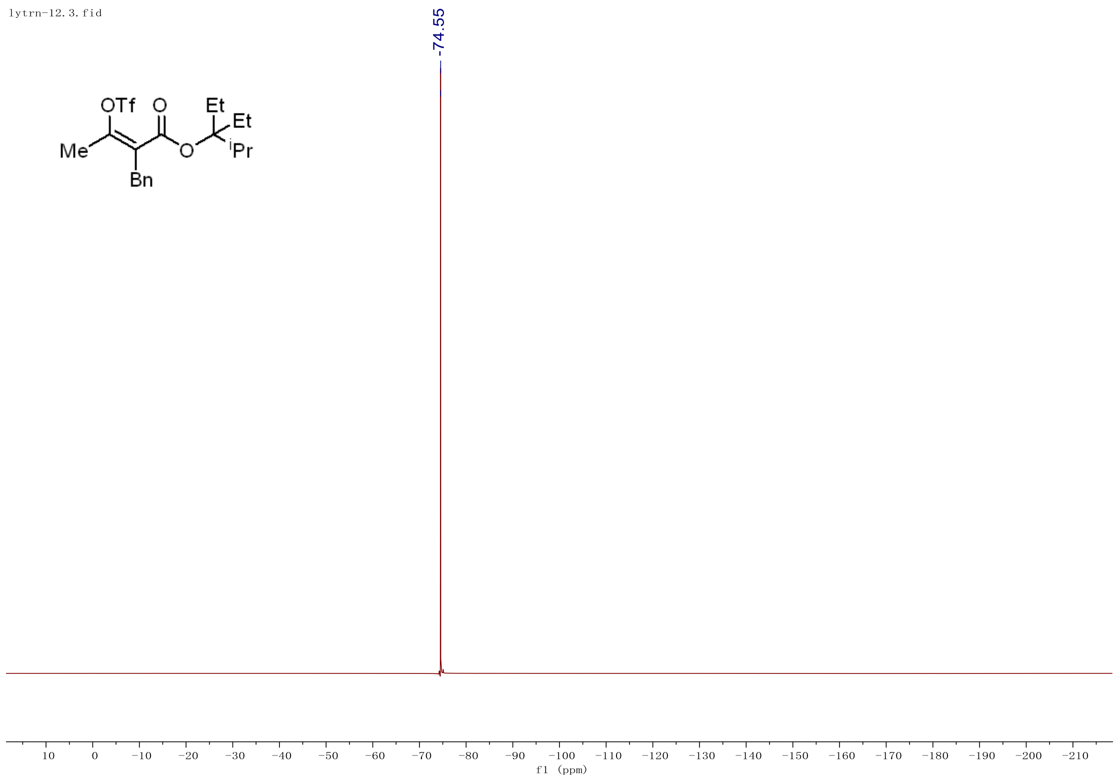

^{19}F NMR spectrum for compound **2j**

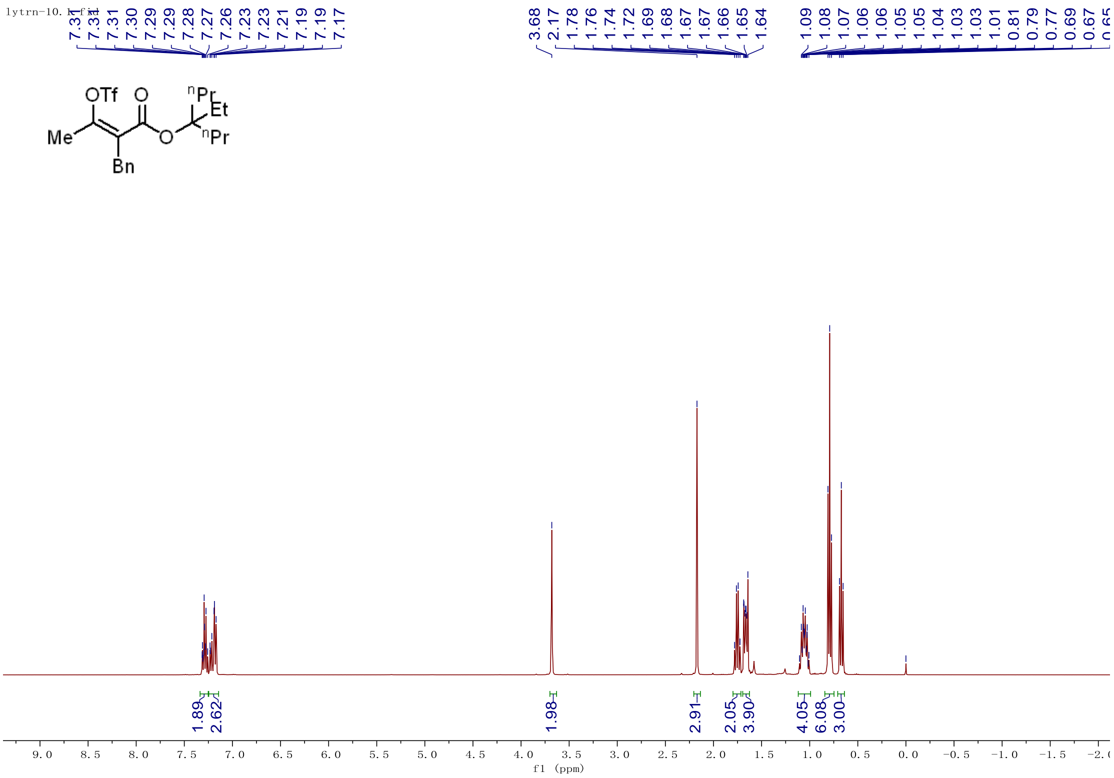

^{1}H NMR spectrum for compound **2k**

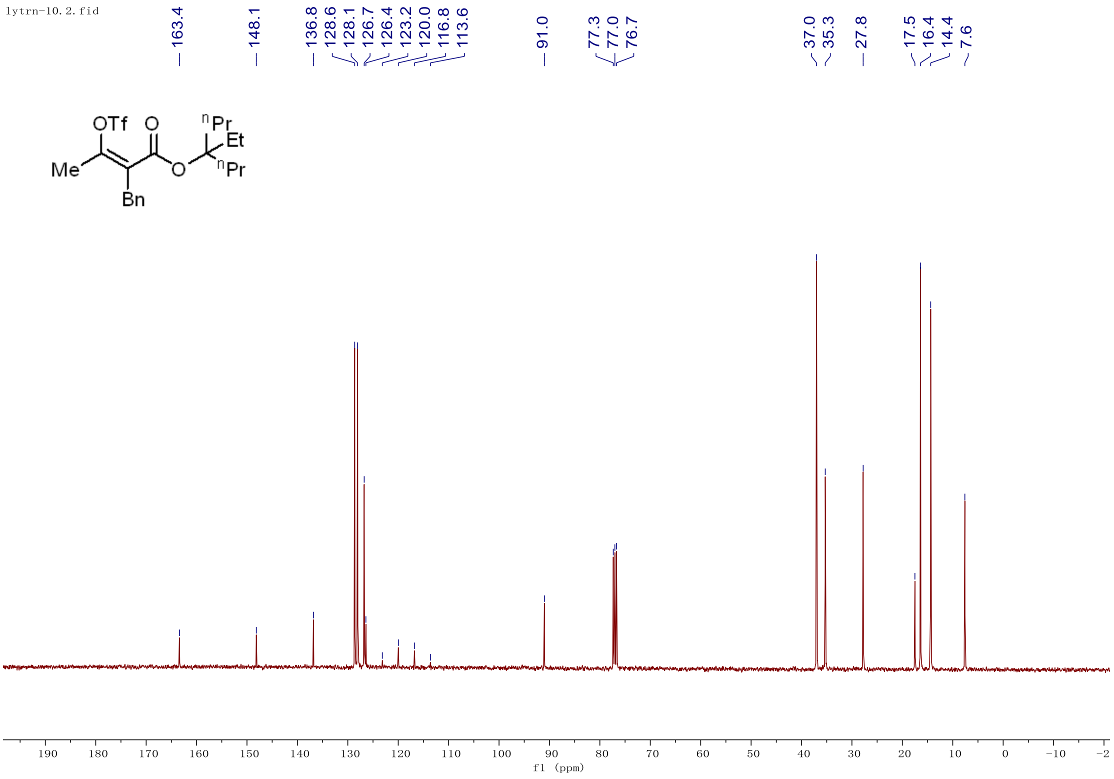

^{13}C NMR spectrum for compound **2k**

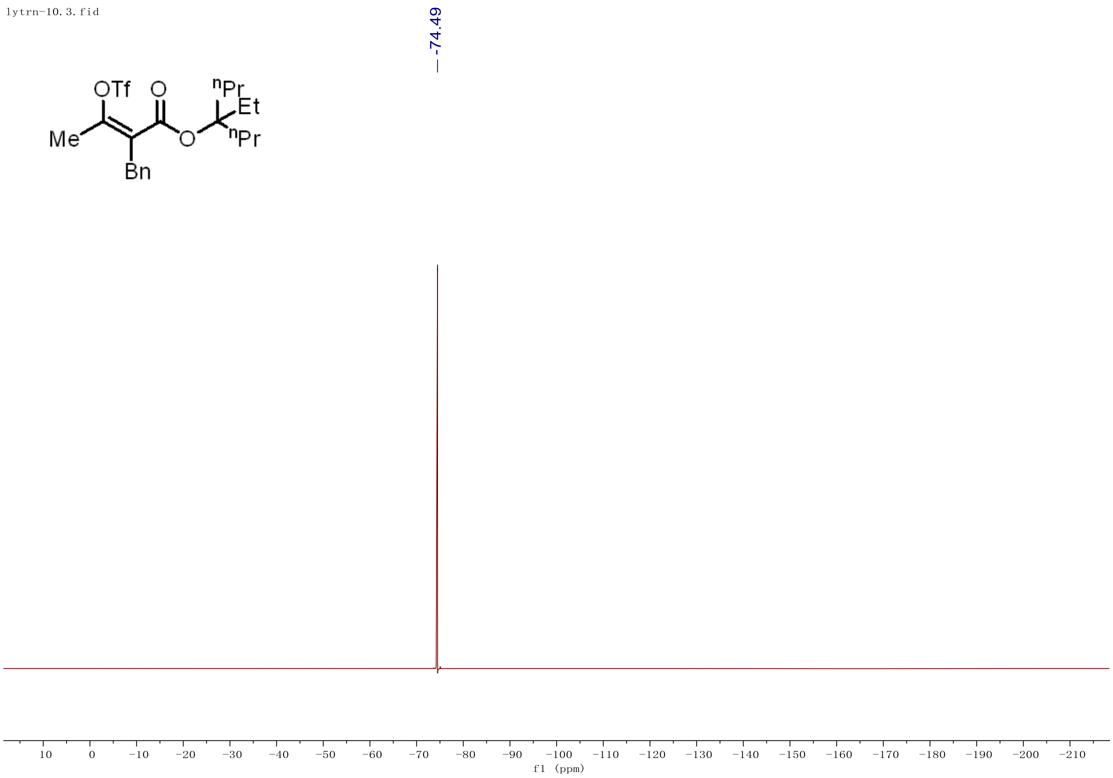

^{19}F NMR spectrum for compound **2k**

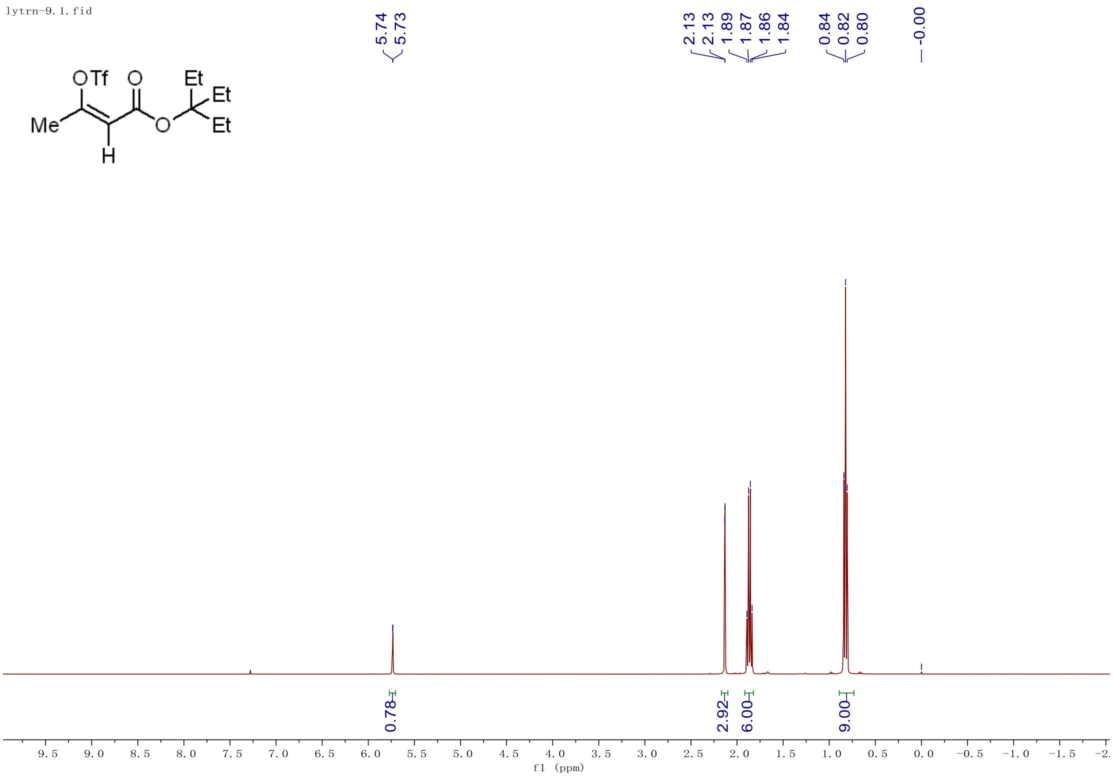

^{1}H NMR spectrum for compound **2l**

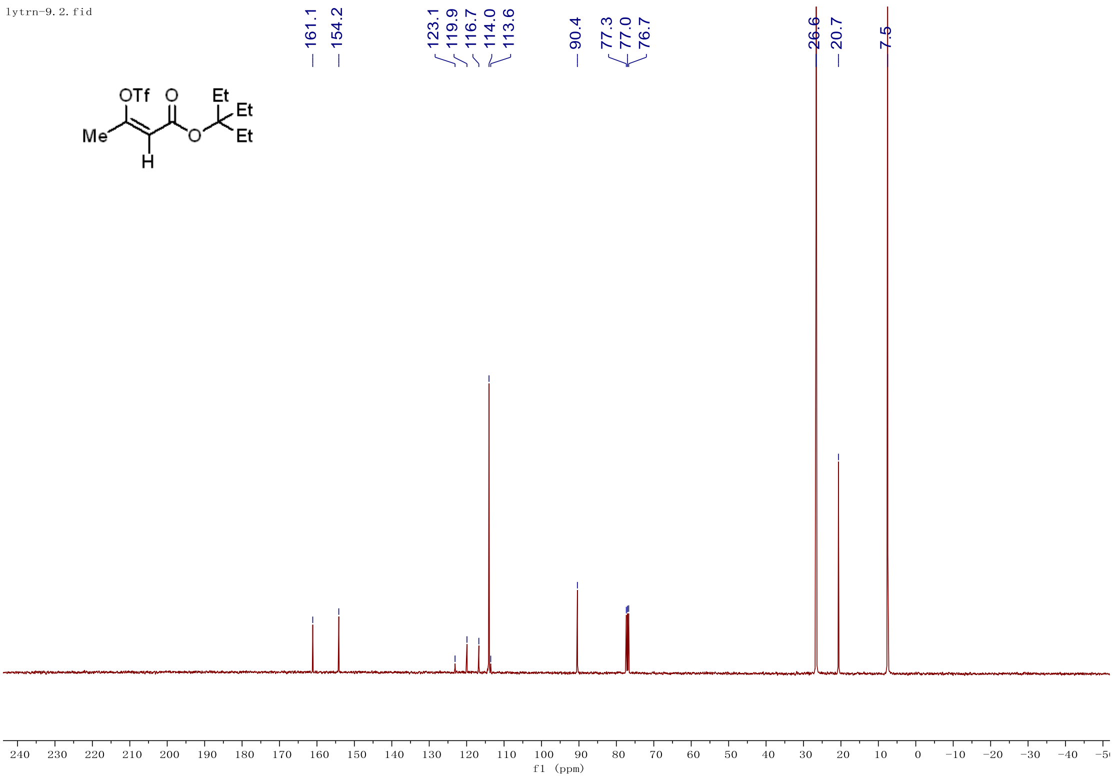

^{13}C NMR spectrum for compound **2l**

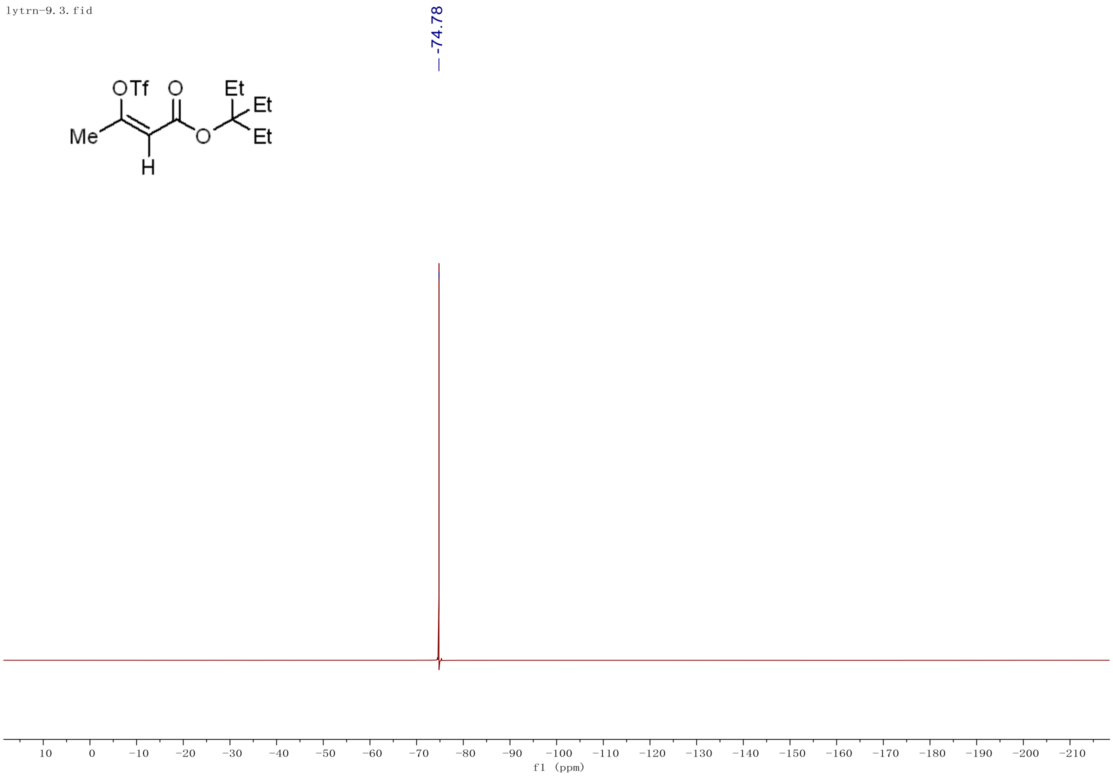

^{19}F NMR spectrum for compound **2l**

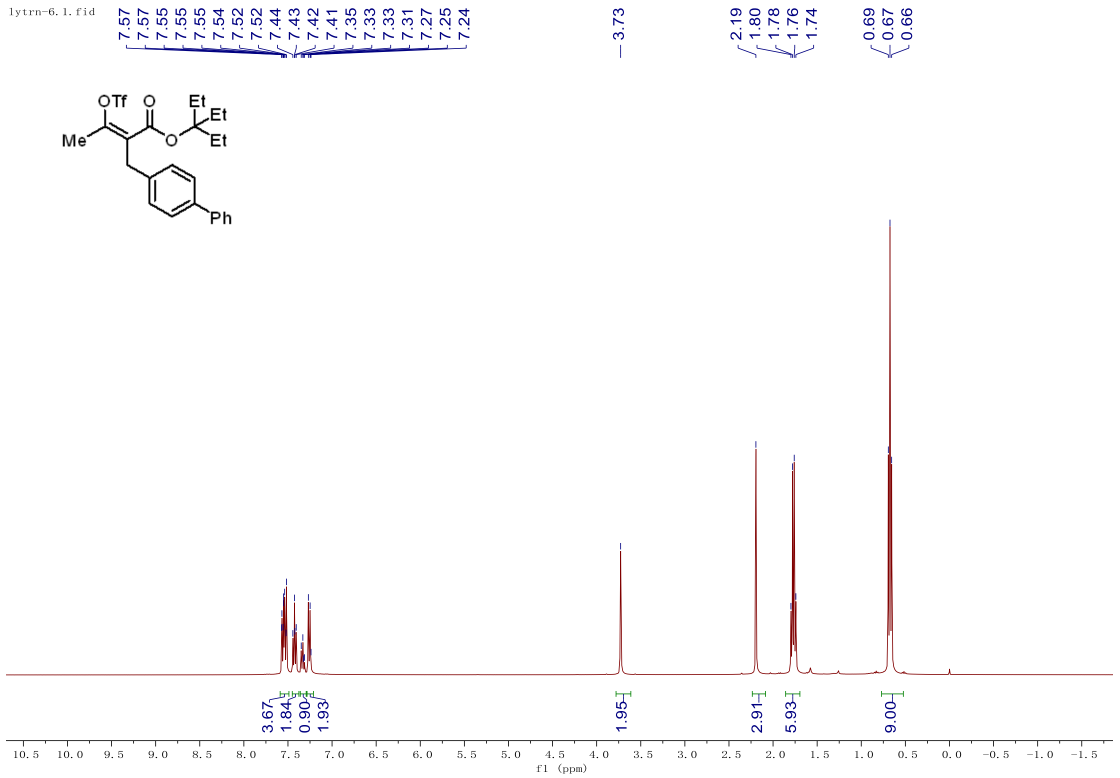

^{1}H NMR spectrum for compound **2m**

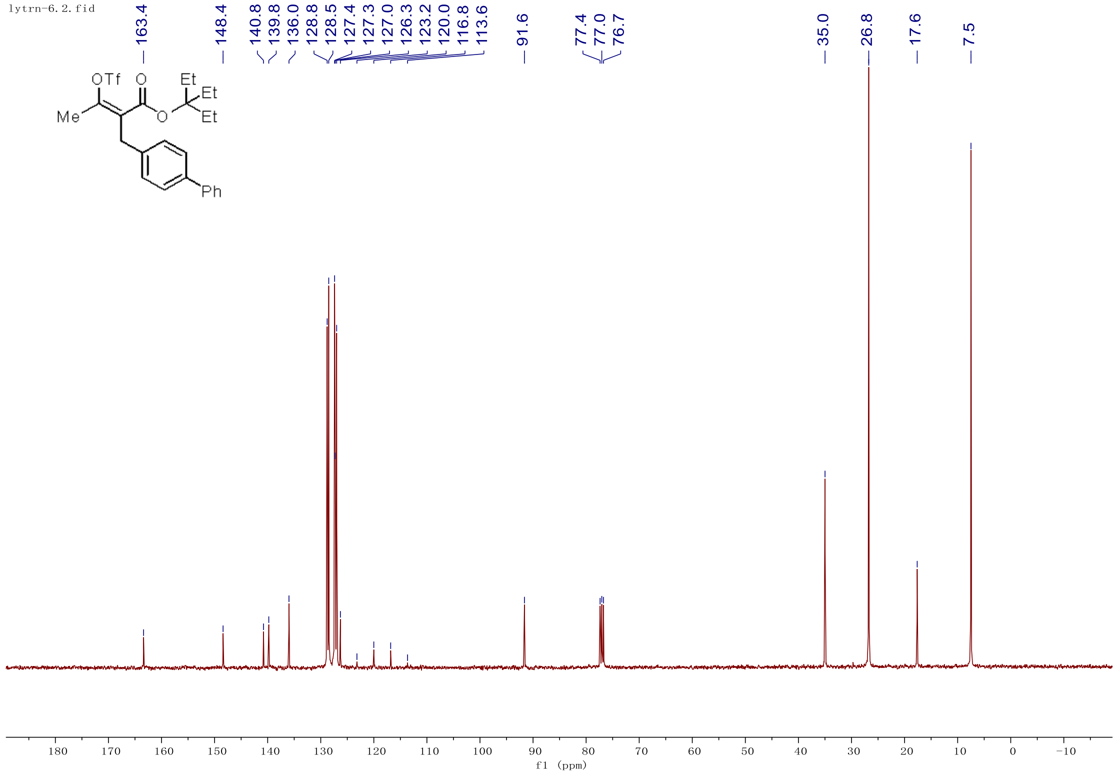

^{13}C NMR spectrum for compound **2m**

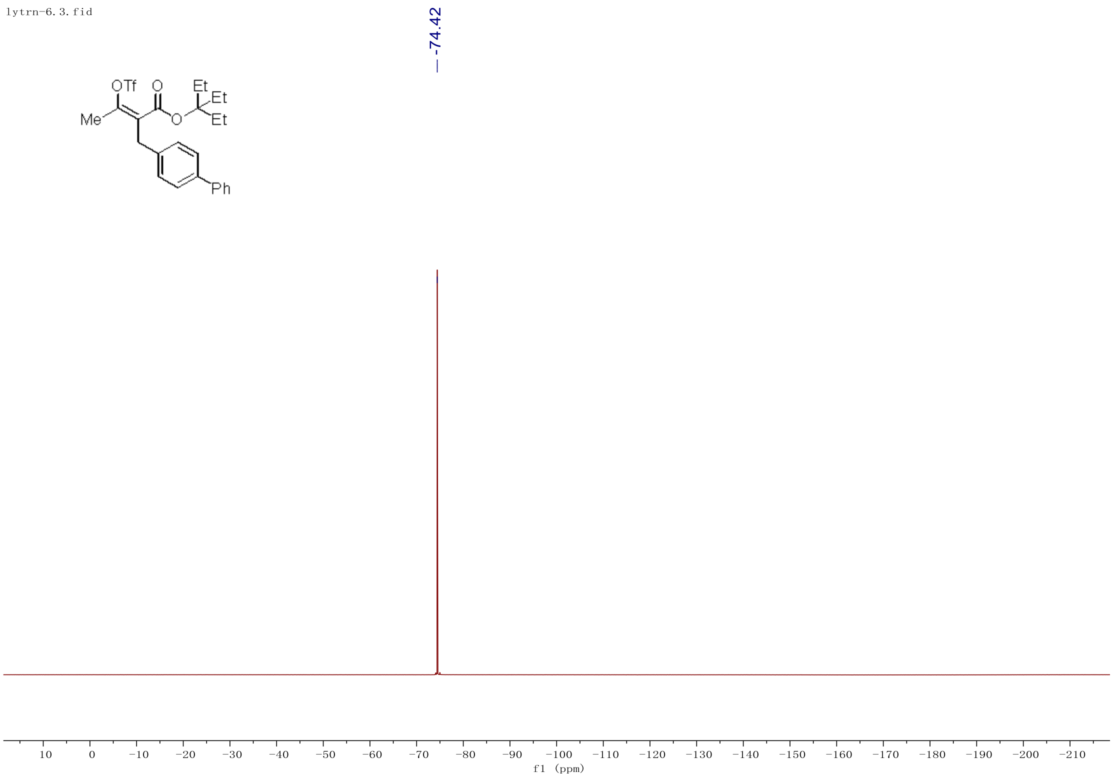

^{19}F NMR spectrum for compound **2m**

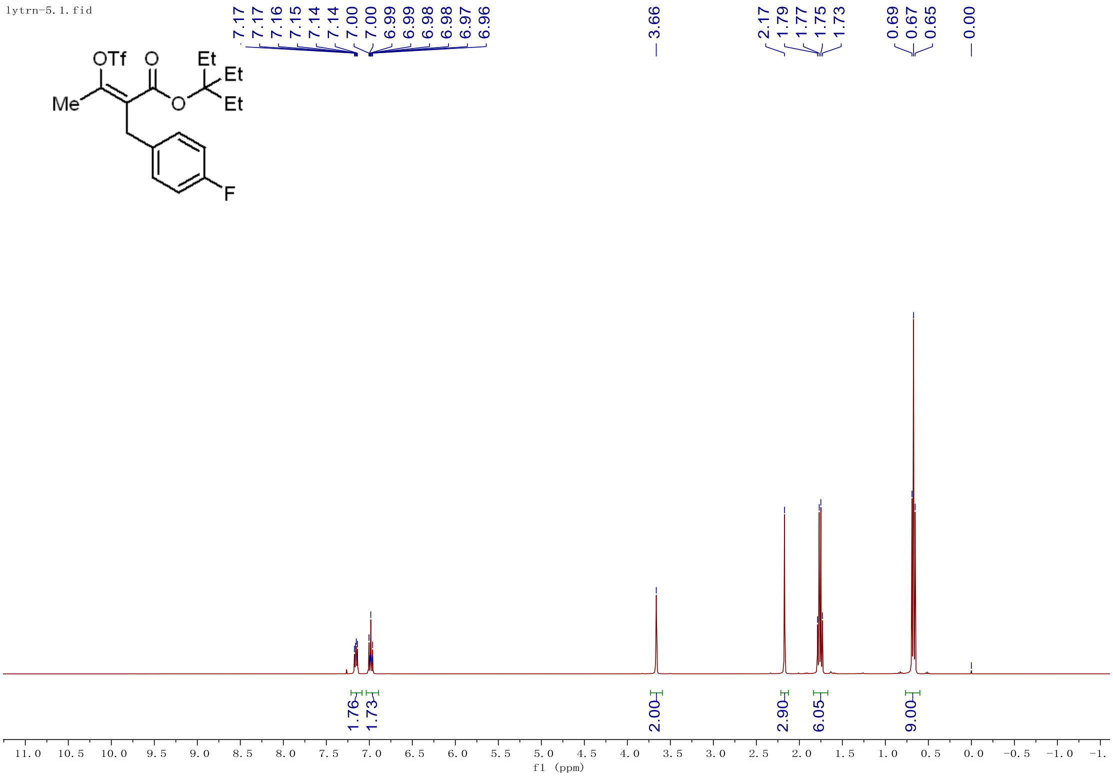

^{1}H NMR spectrum for compound **2n**

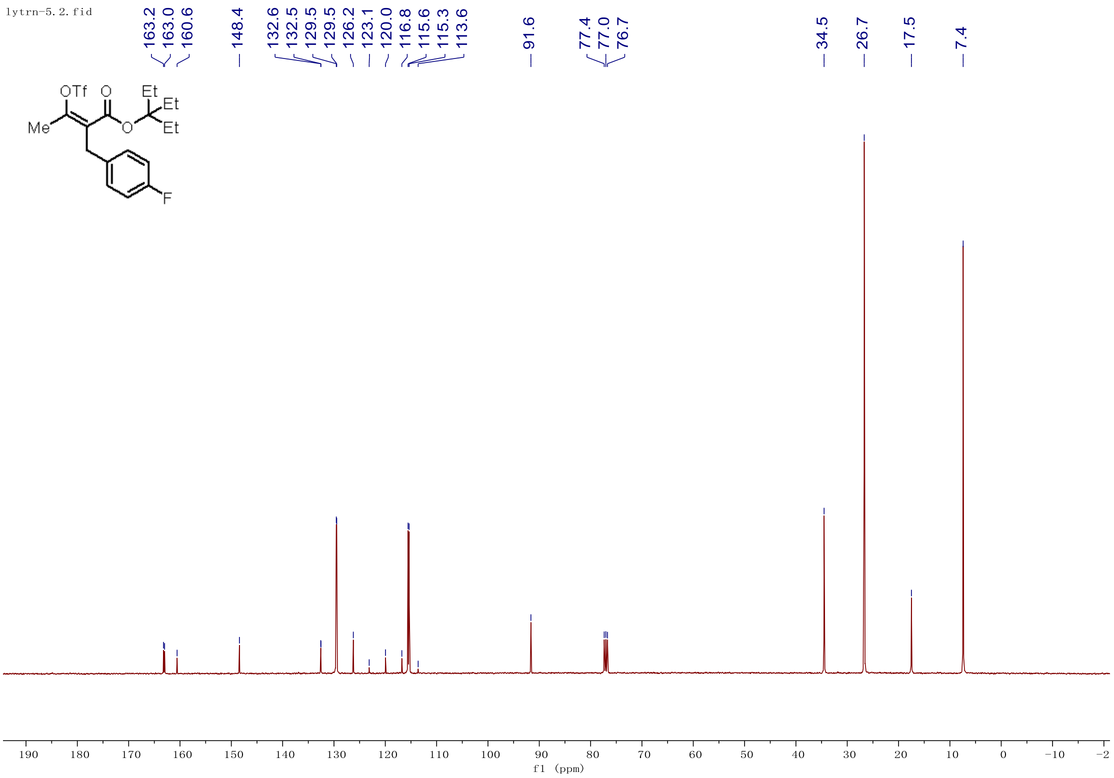

^{13}C NMR spectrum for compound **2n**

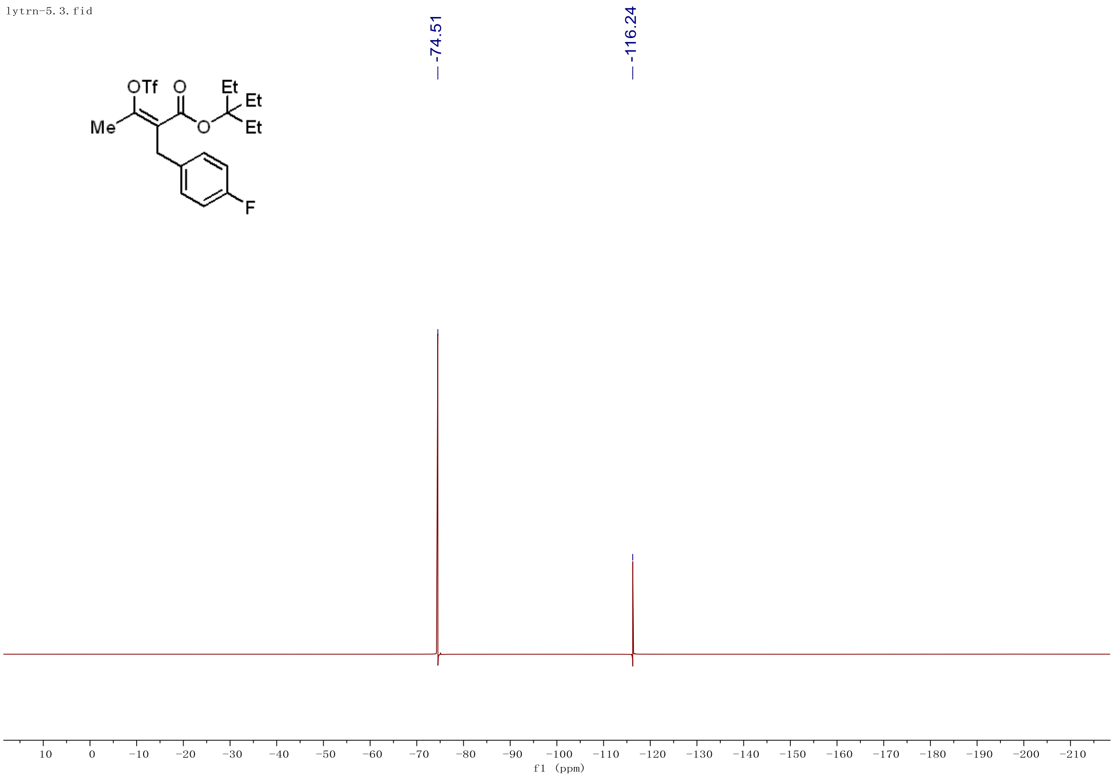

^{19}F NMR spectrum for compound **2n**

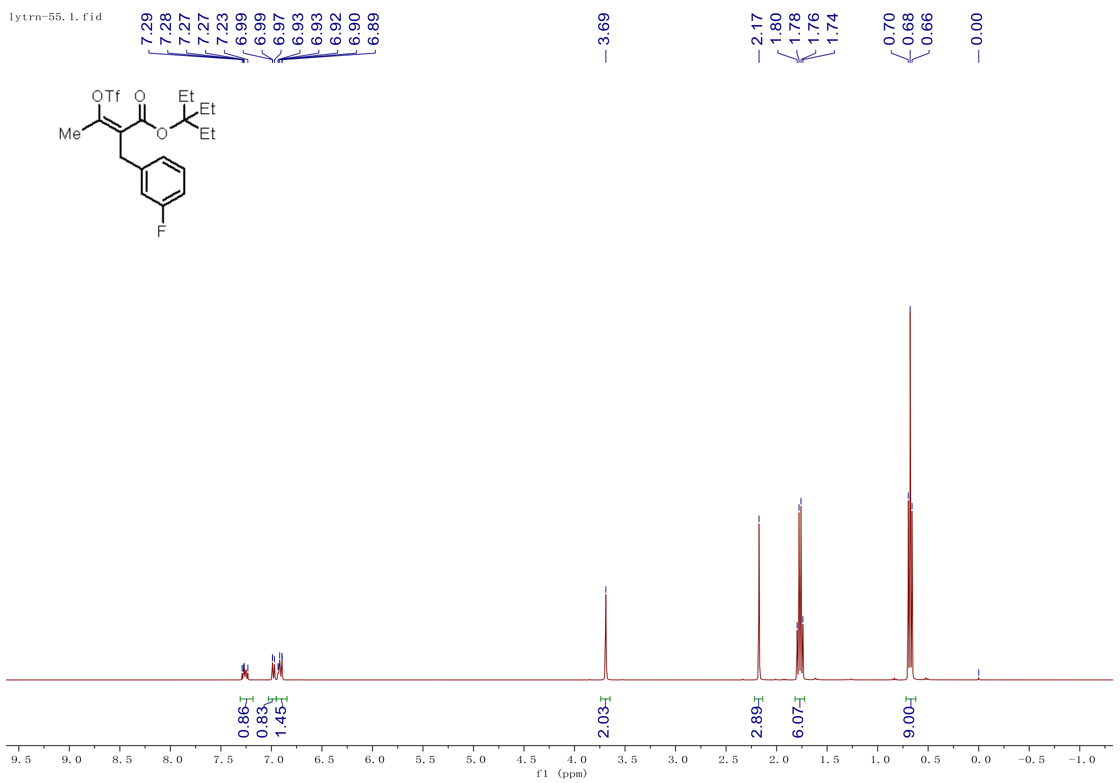

^{1}H NMR spectrum for compound **2o**

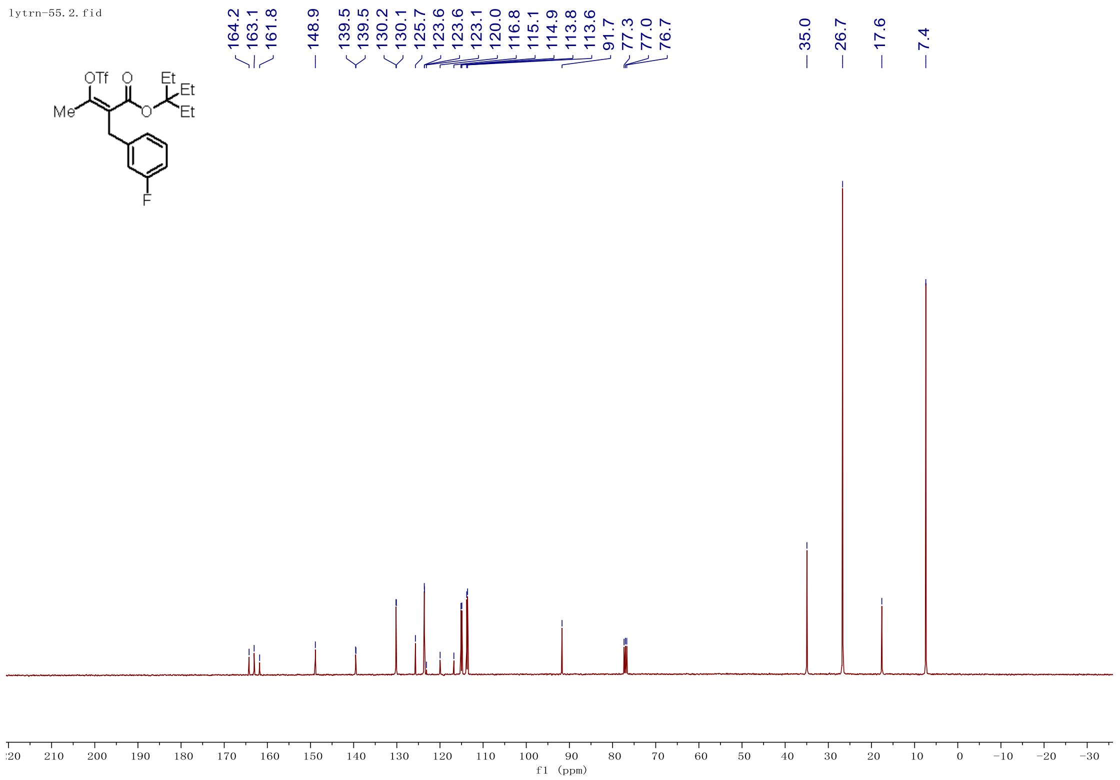

^{13}C NMR spectrum for compound **2o**

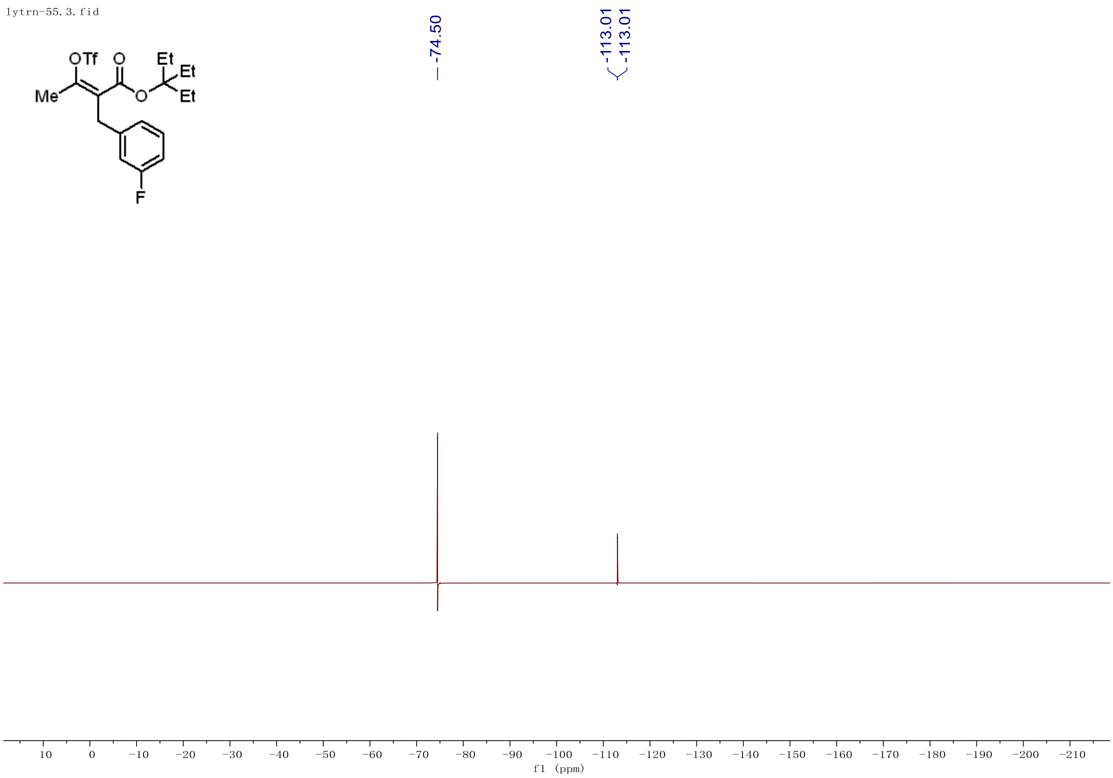

^{19}F NMR spectrum for compound **2o**

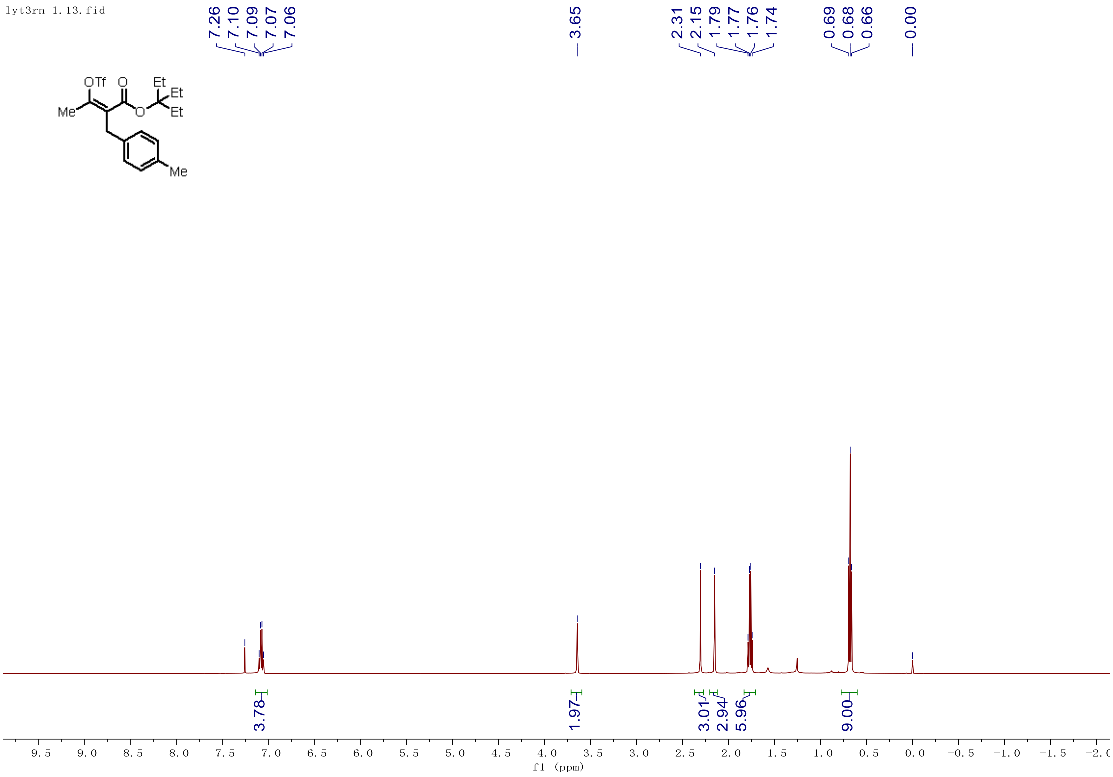

^{1}H NMR spectrum for compound **2p**

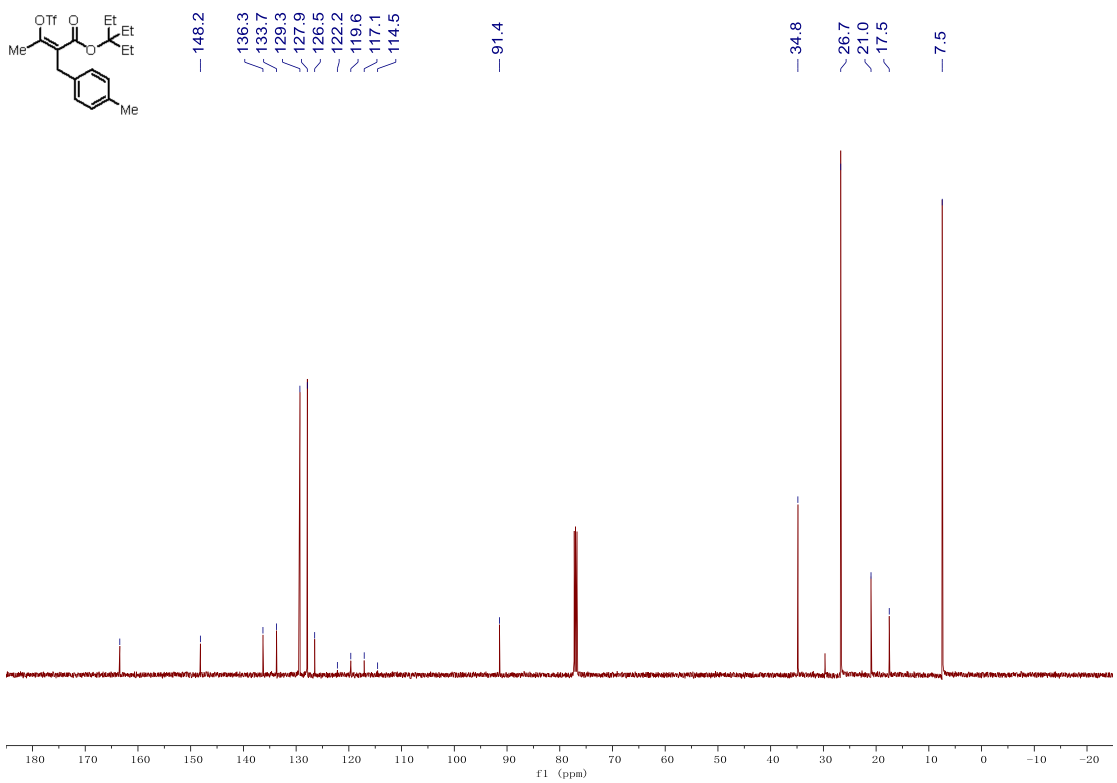

^{13}C NMR spectrum for compound **2p**

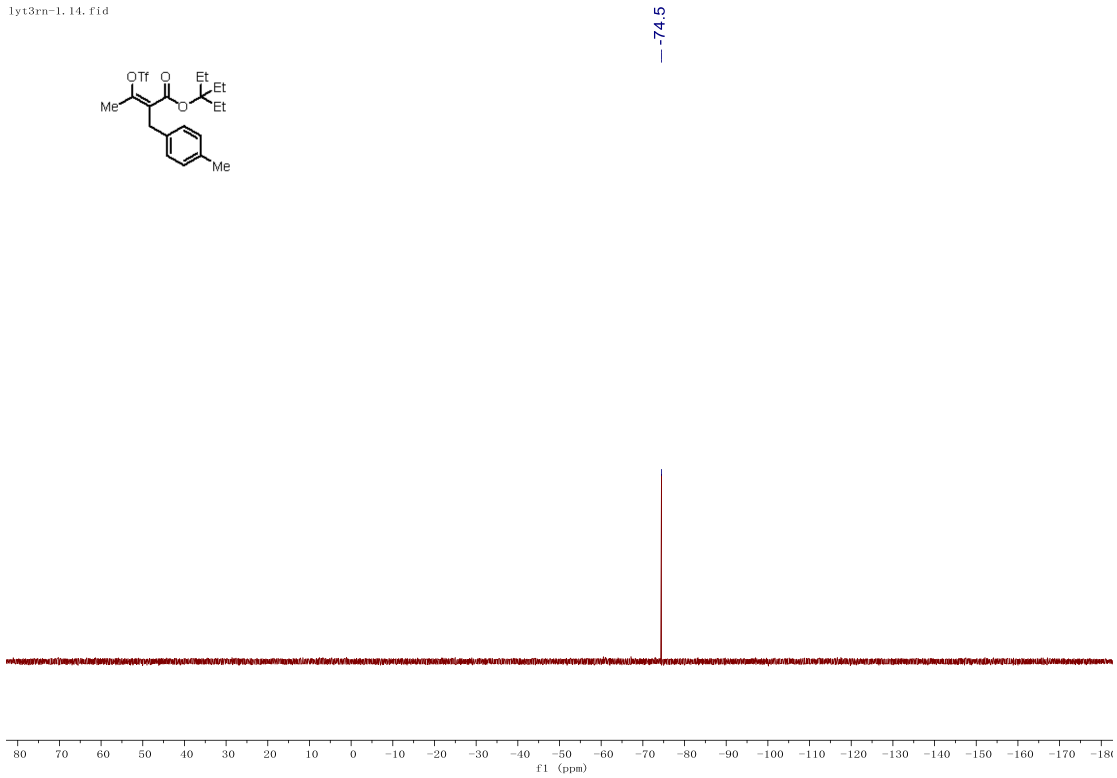

^{19}F NMR spectrum for compound **2p**

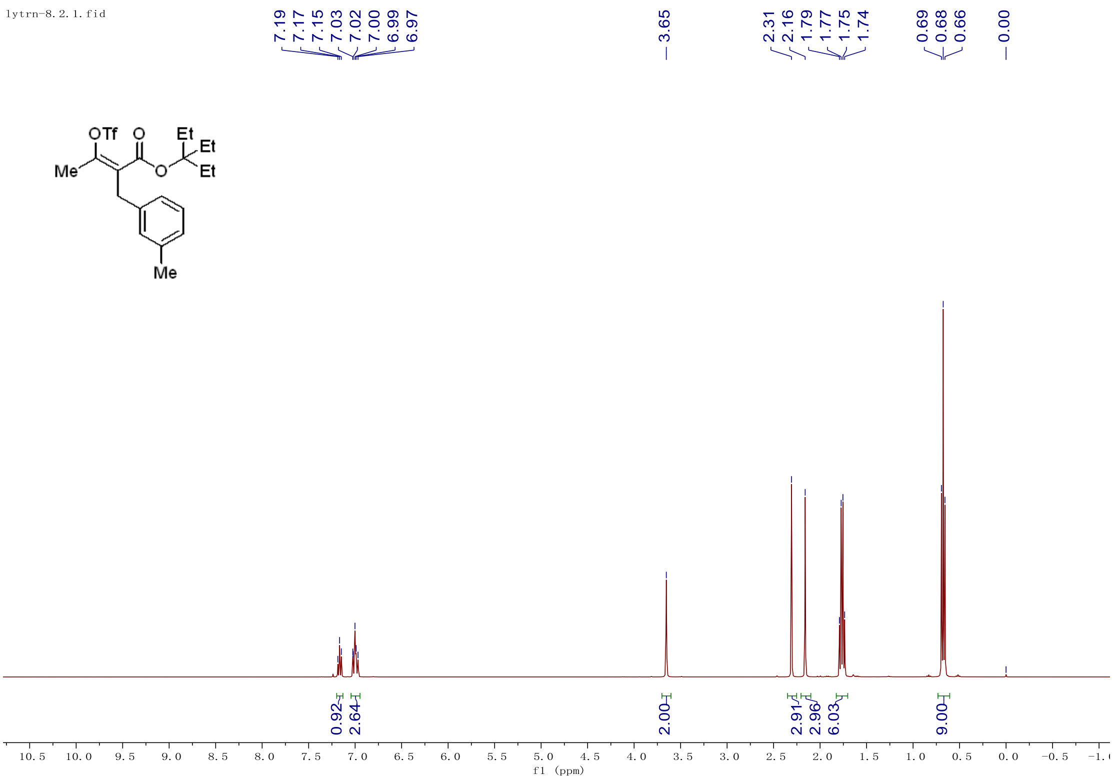

^{1}H NMR spectrum for compound **2q**

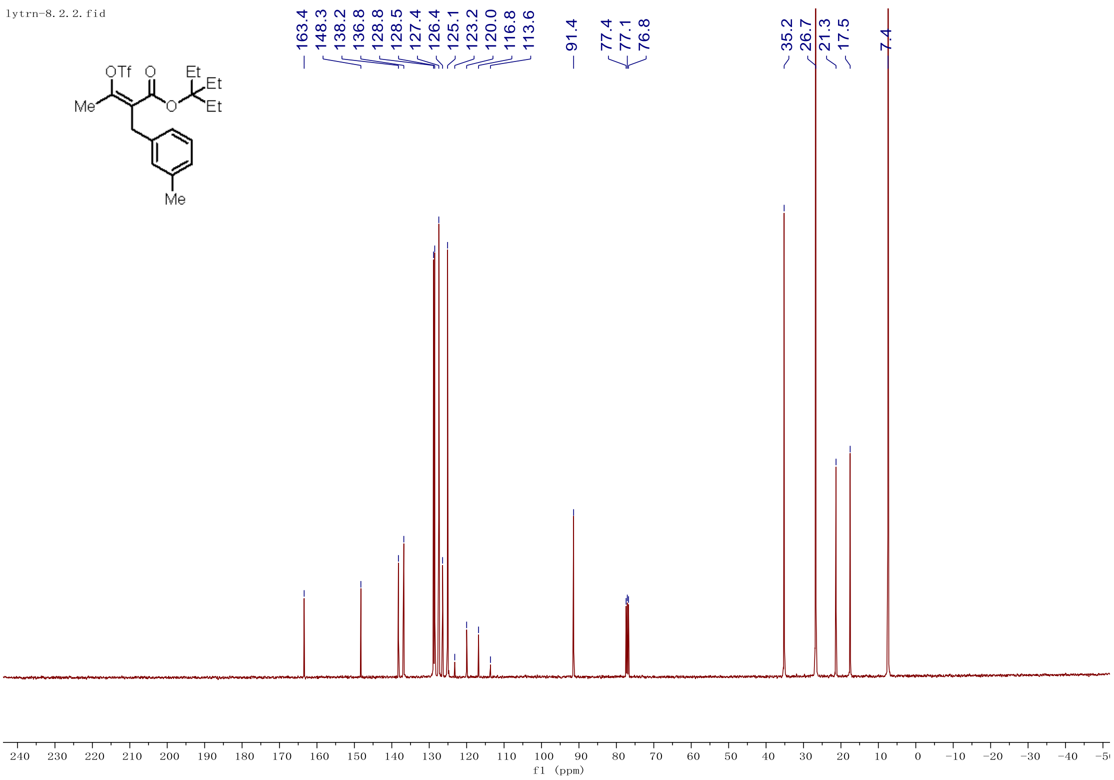

^{13}C NMR spectrum for compound **2q**

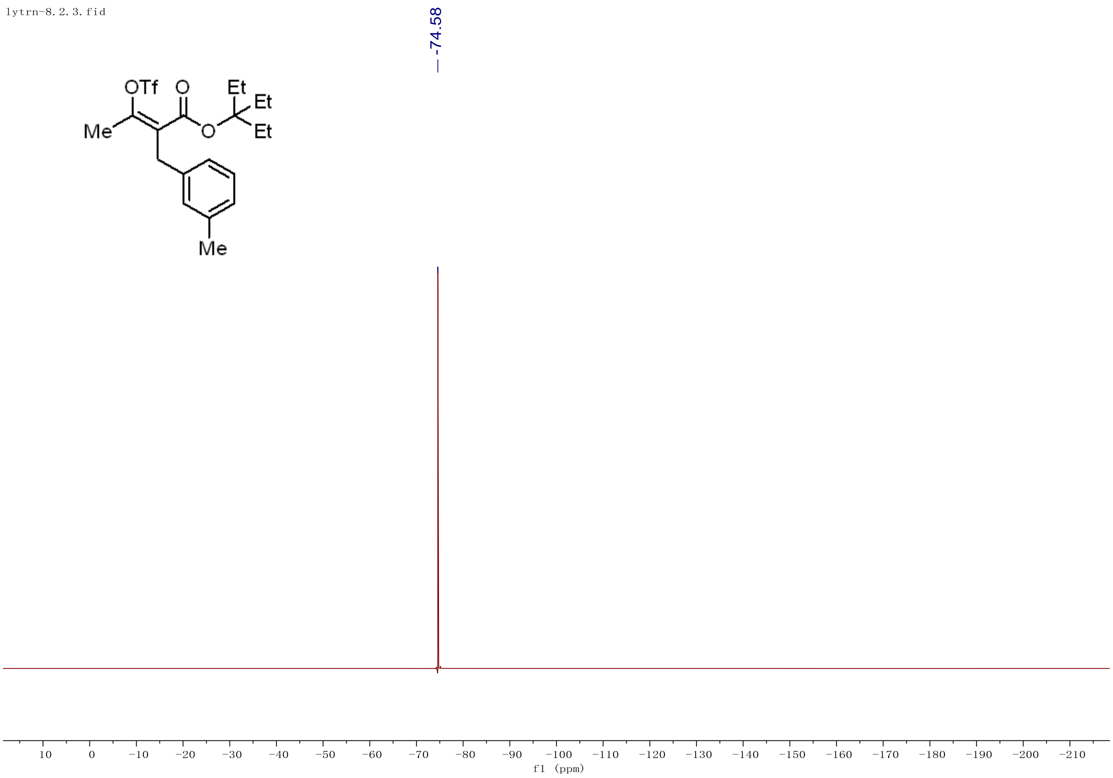

^{19}F NMR spectrum for compound **2q**

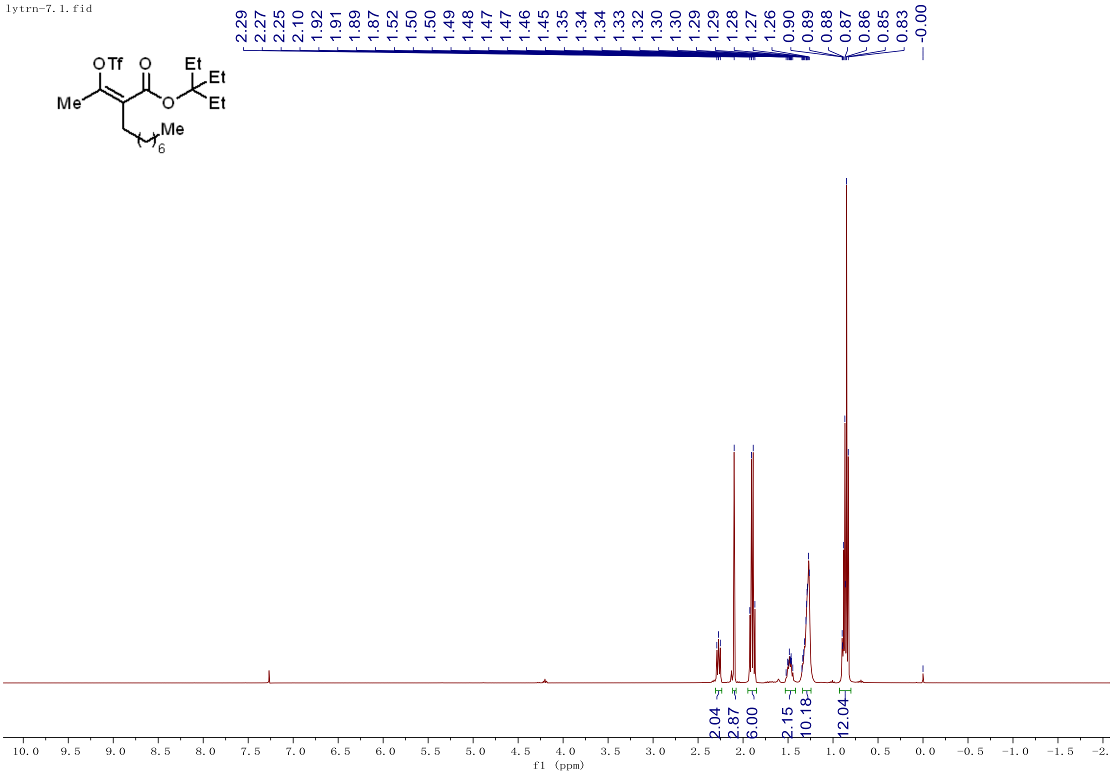

^{1}H NMR spectrum for compound **2r**

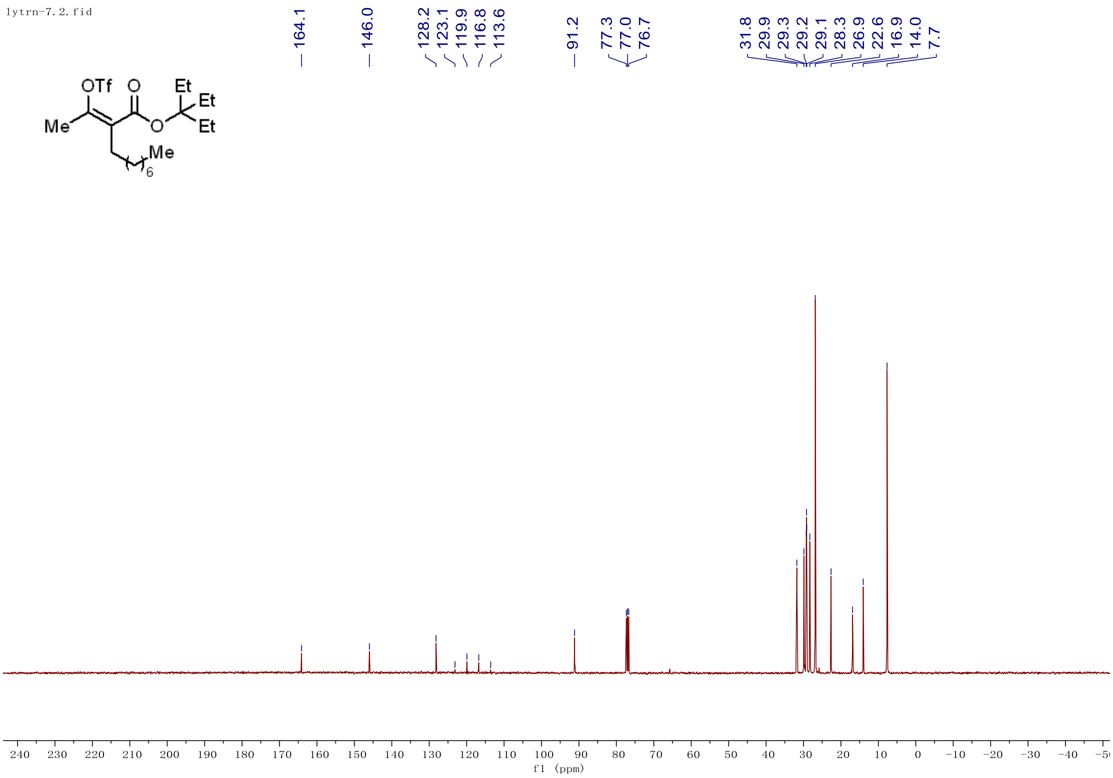

^{13}C NMR spectrum for compound **2r**

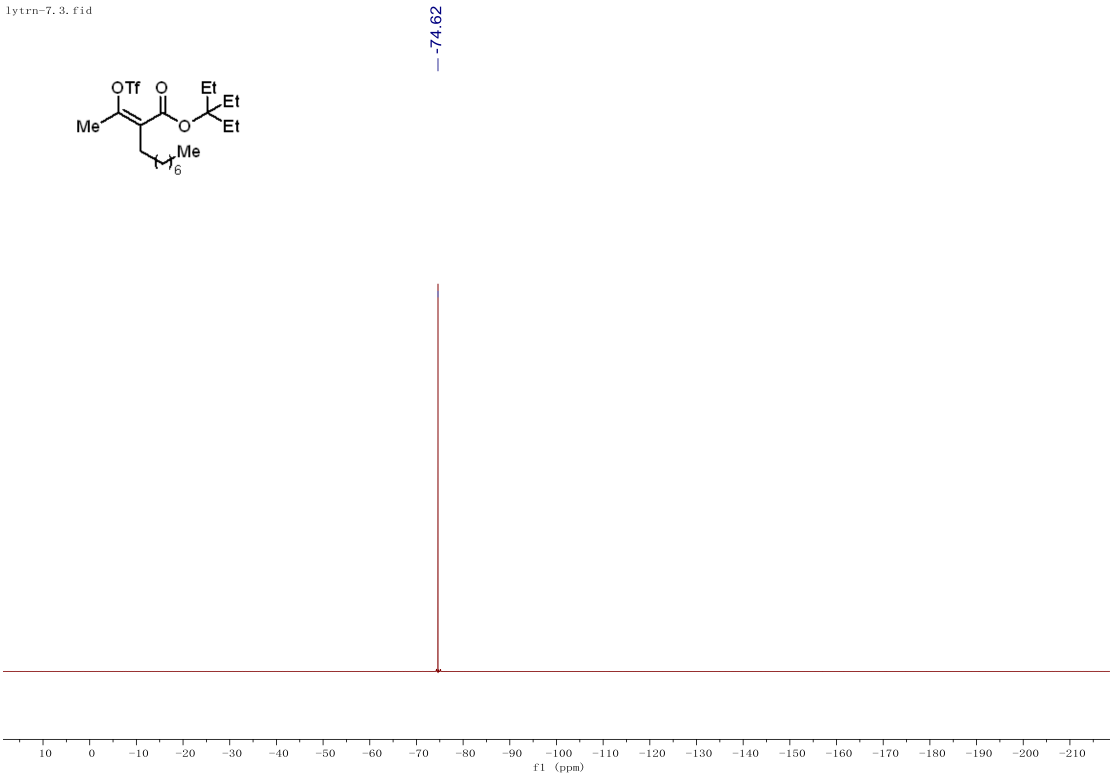

^{19}F NMR spectrum for compound **2r**

7. NMR spectrum of products

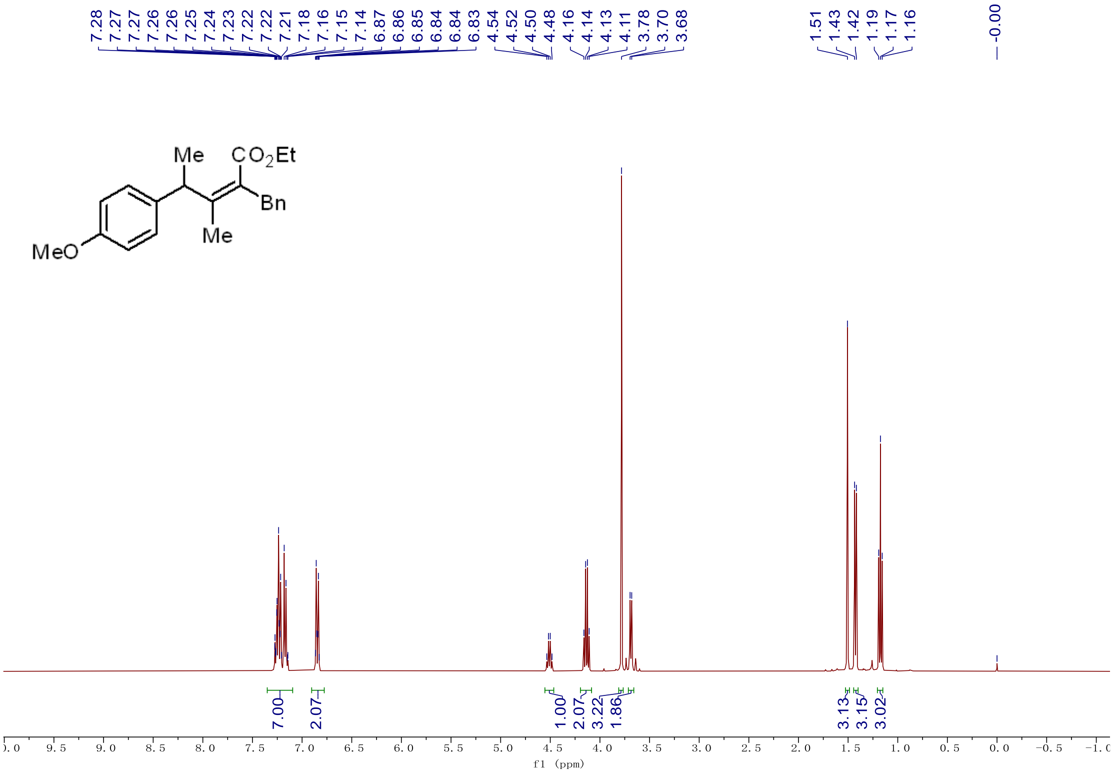

^{1}H NMR spectrum for compound **3a**

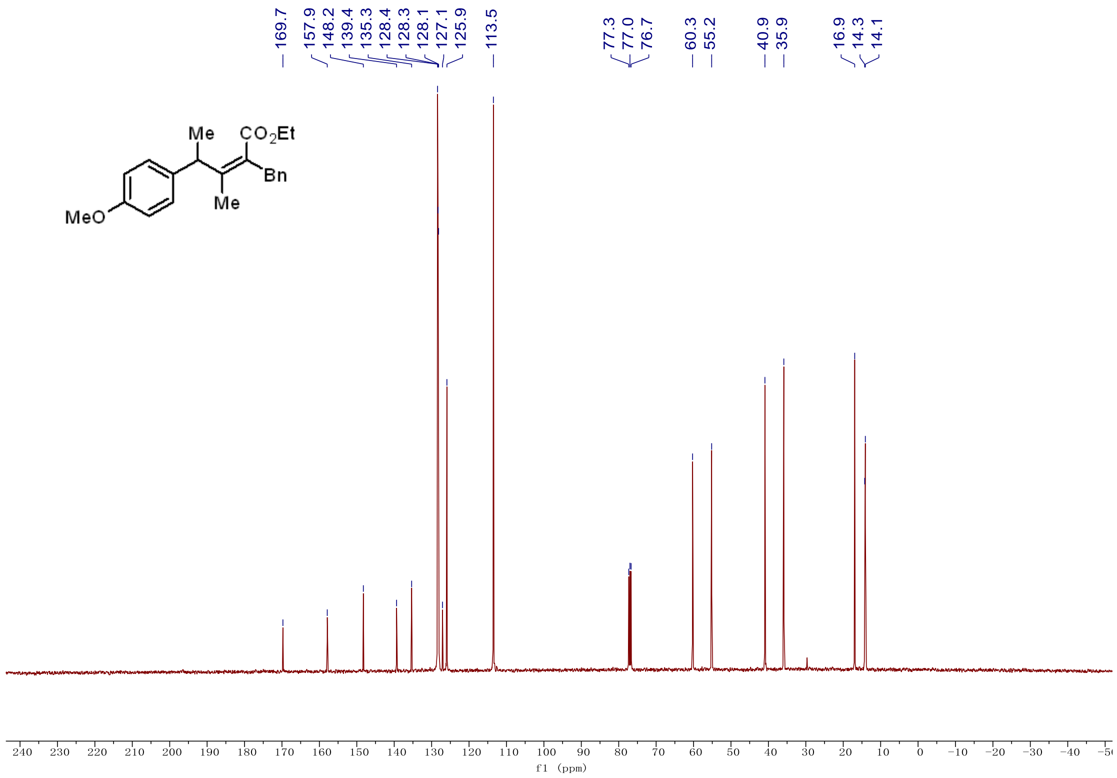

^{13}C NMR spectrum for compound **3a**

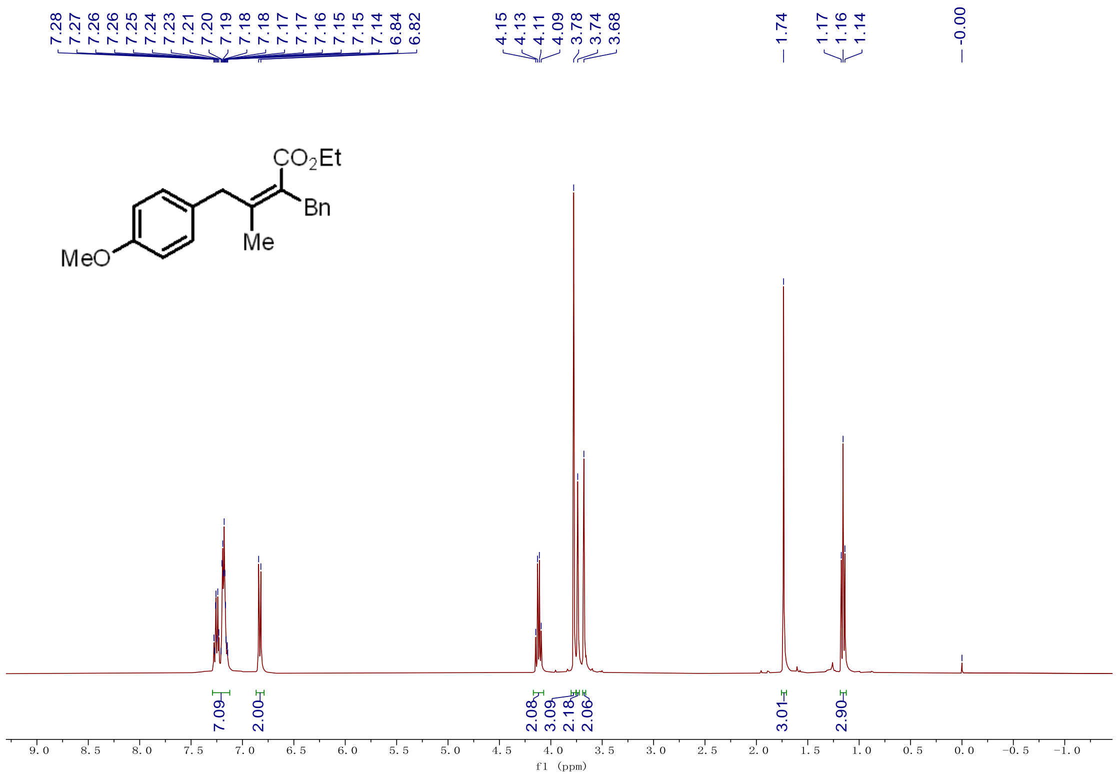

^{1}H NMR spectrum for compound **3b**

.

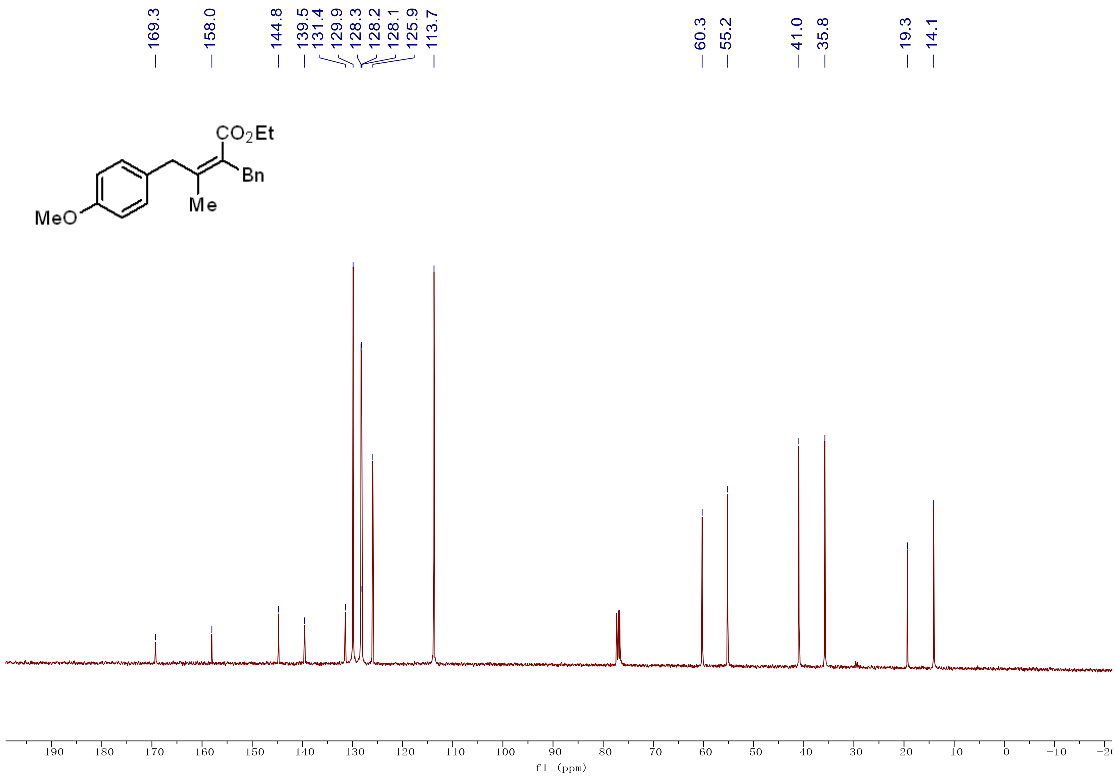

^{13}C NMR spectrum for compound **3b**

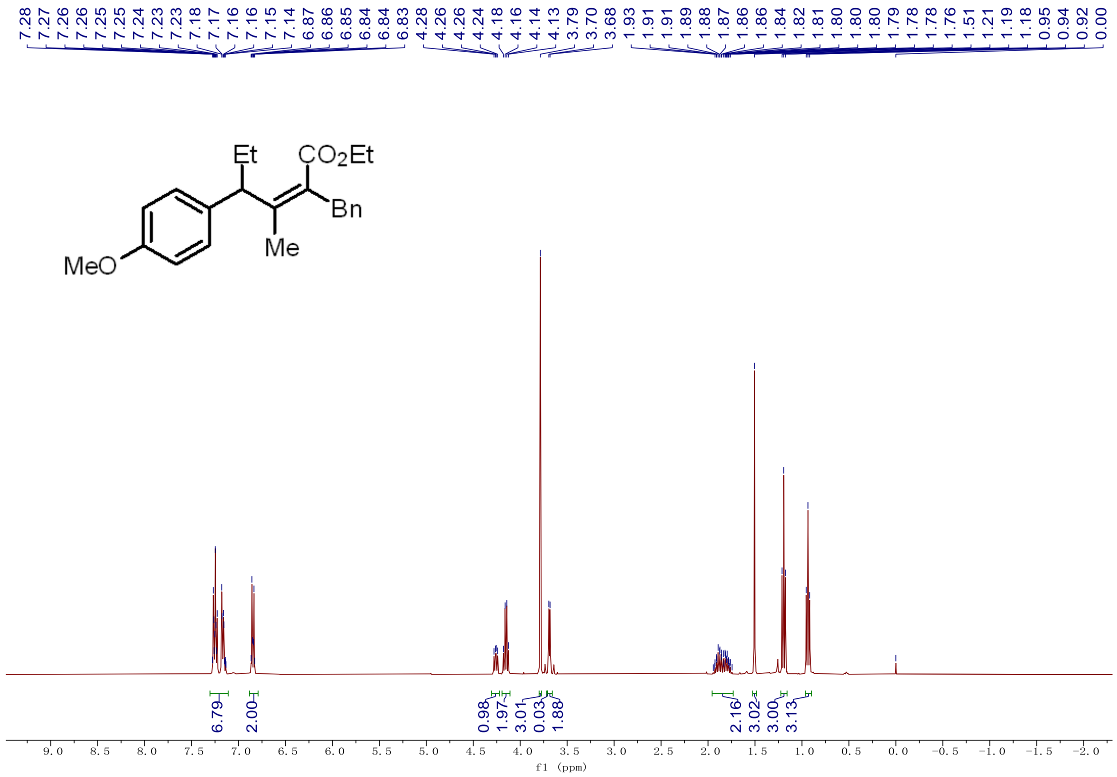

^{1}H NMR spectrum for compound **3c**

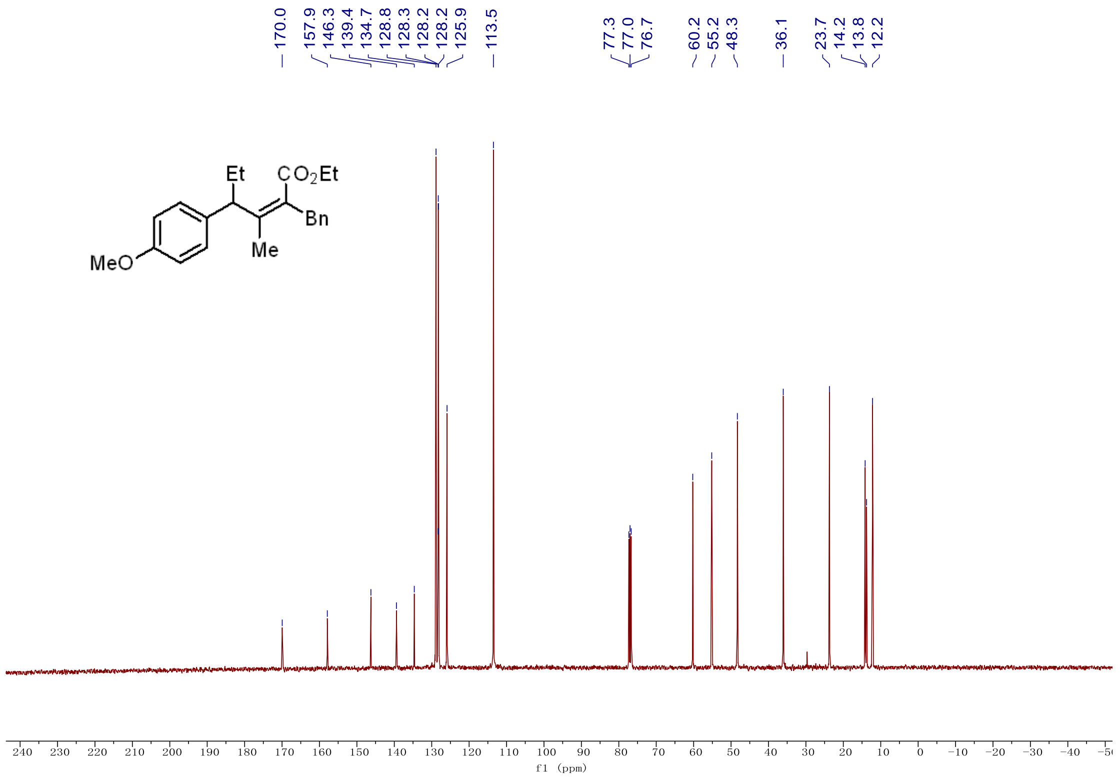

^{13}C NMR spectrum for compound **3c**

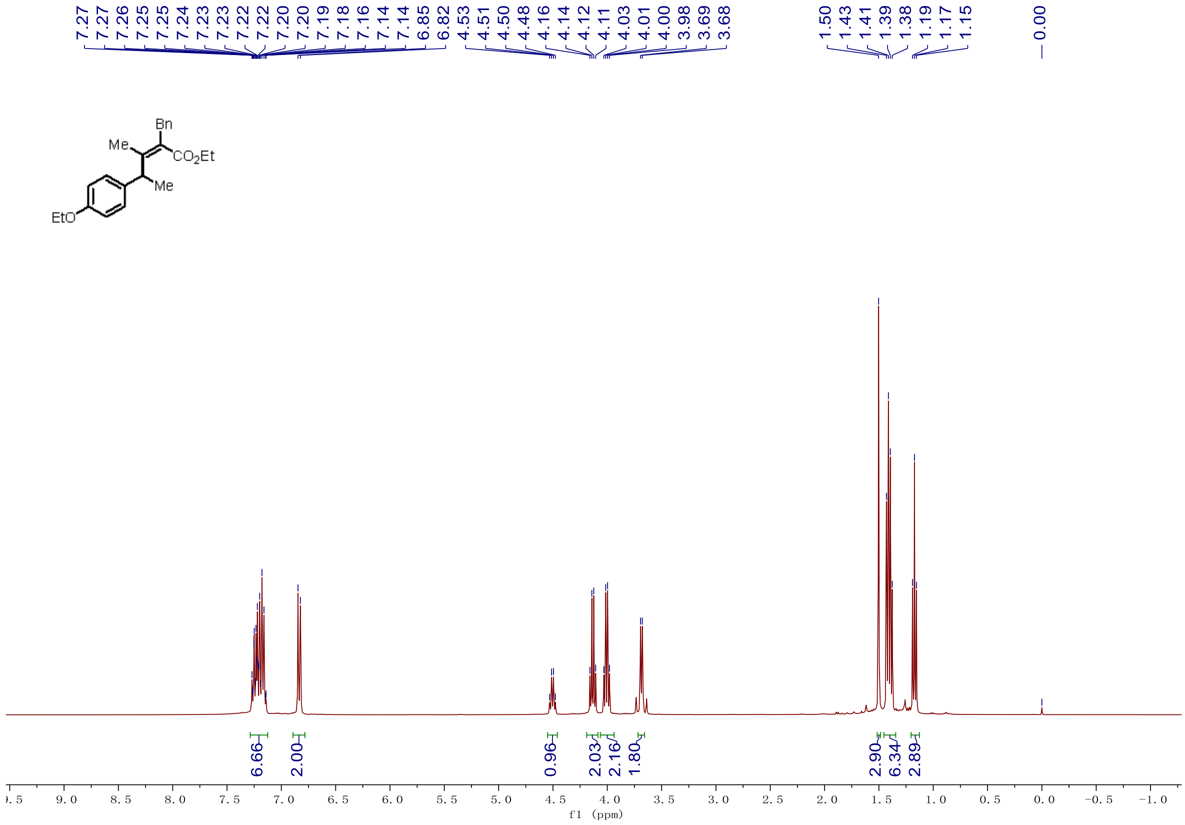

^{1}H NMR spectrum for compound **3d**

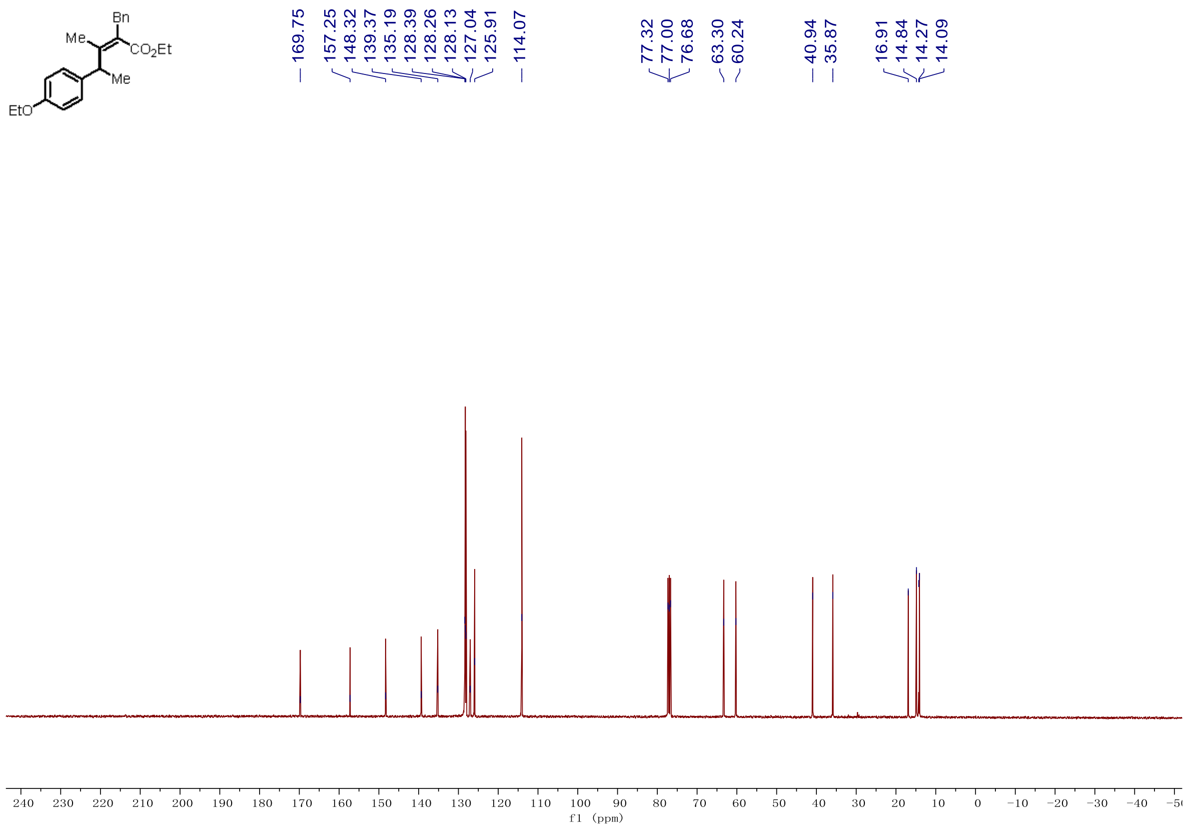

^{13}C NMR spectrum for compound **3d**

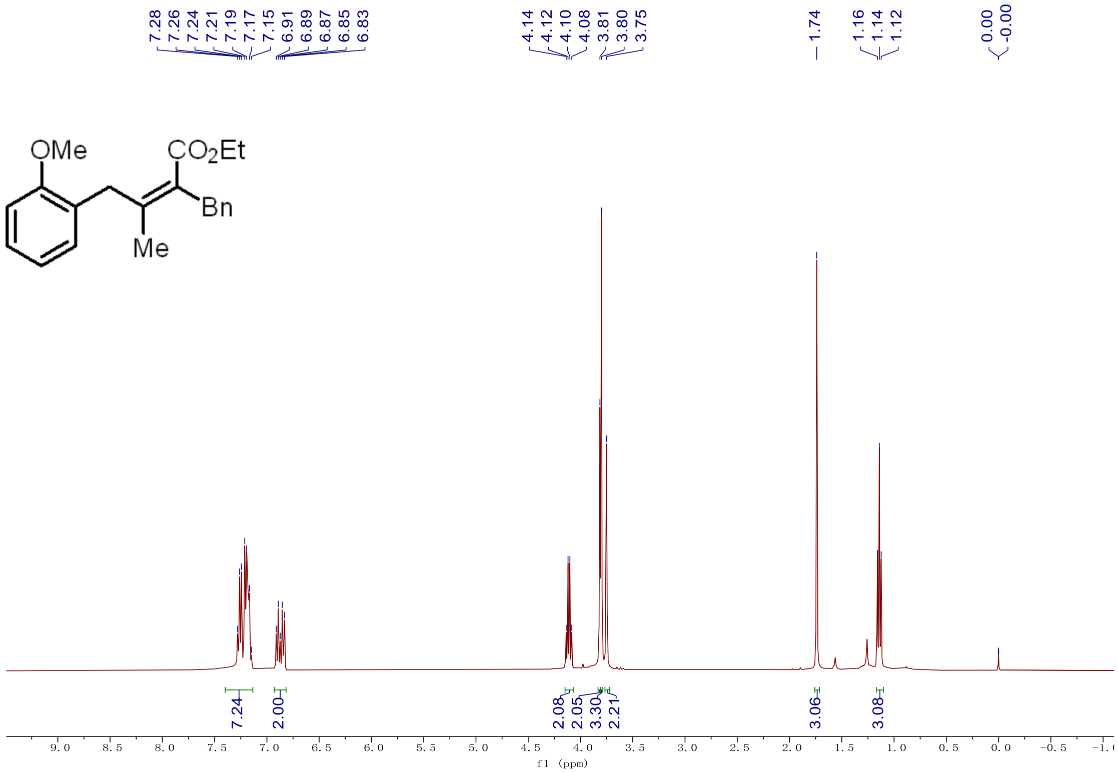

^{1}H NMR spectrum for compound **3e**

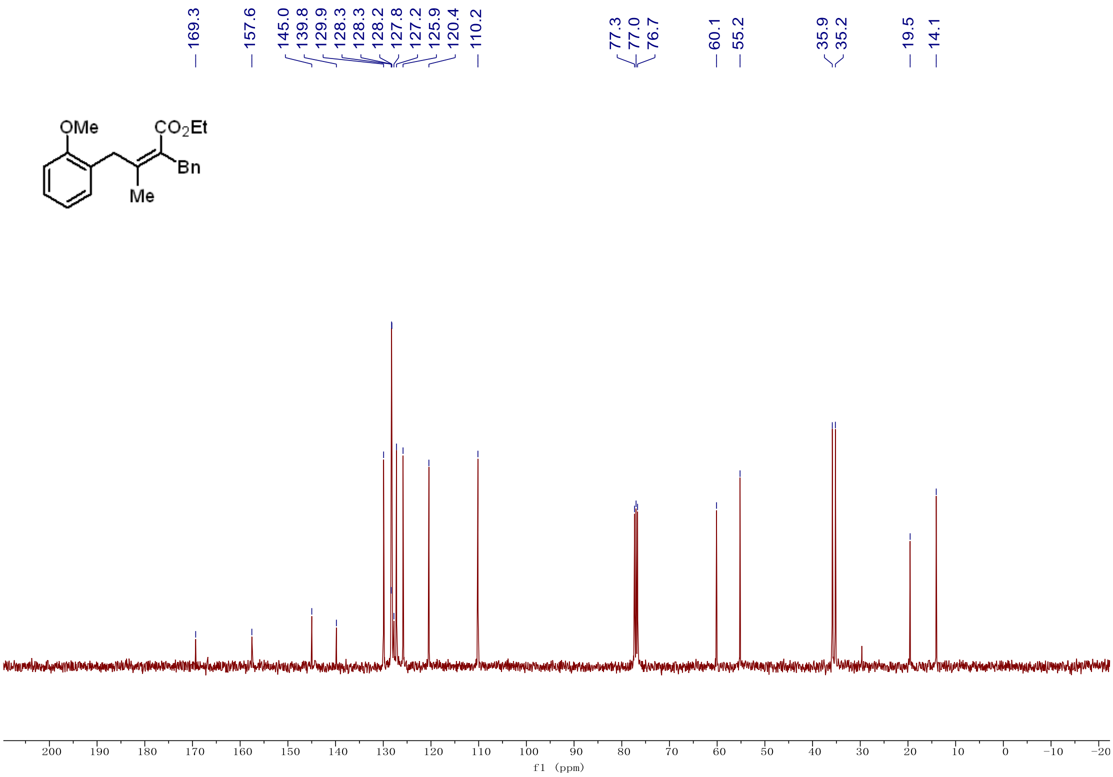

^{13}C NMR spectrum for compound **3e**

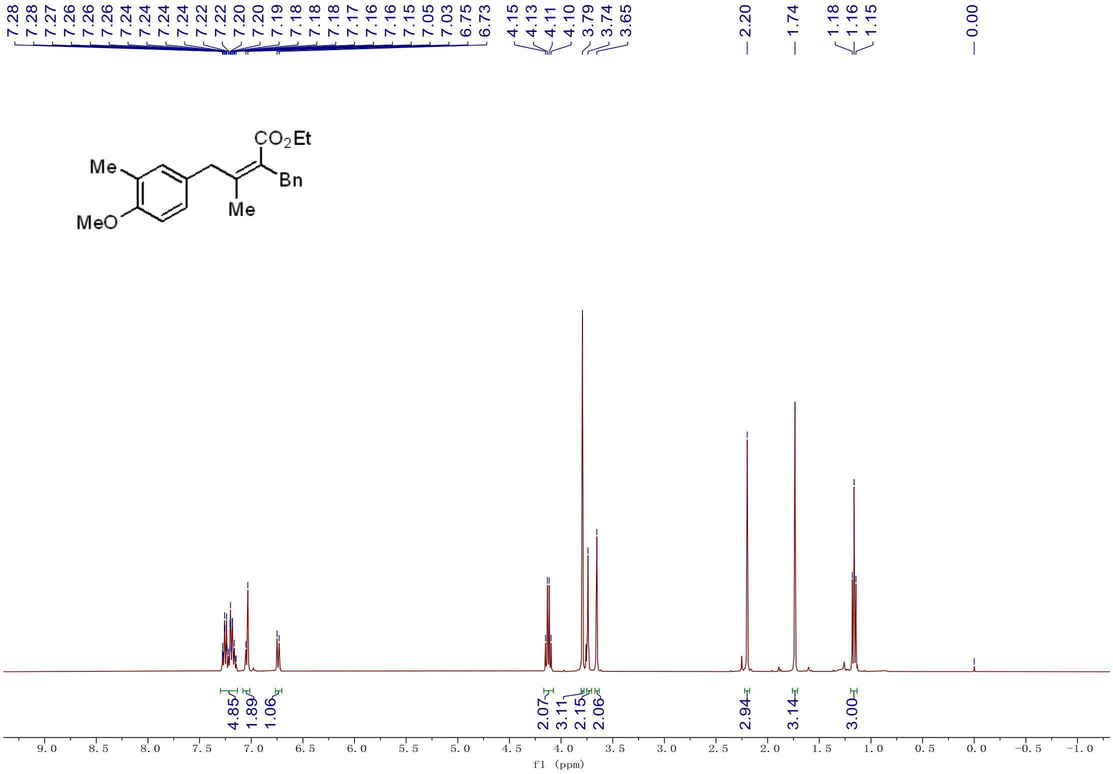

^{1}H NMR spectrum for compound **3f**

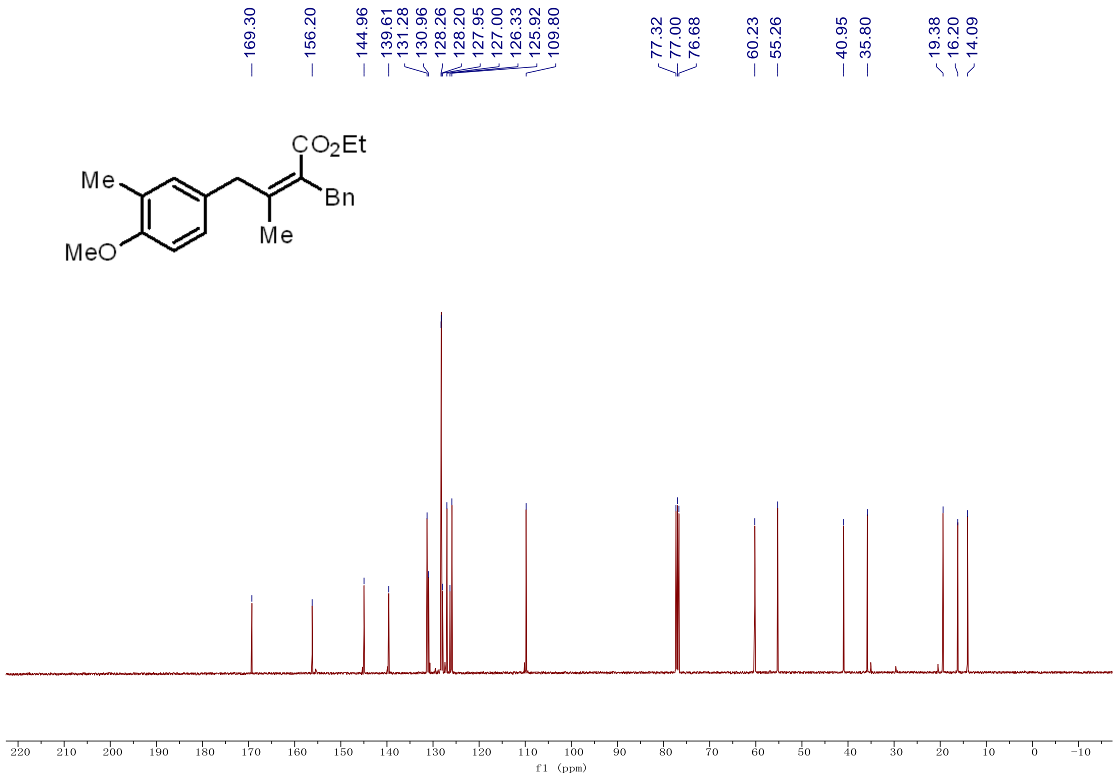

^{13}C NMR spectrum for compound **3f**

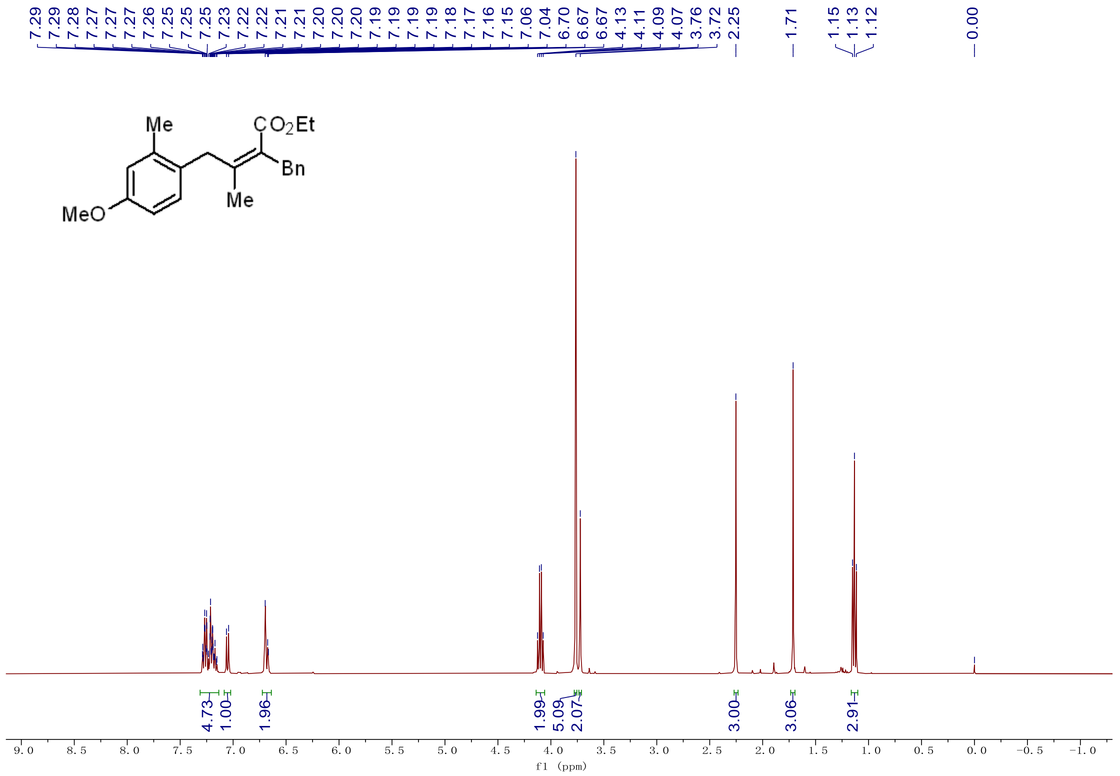

^{1}H NMR spectrum for compound **3g**

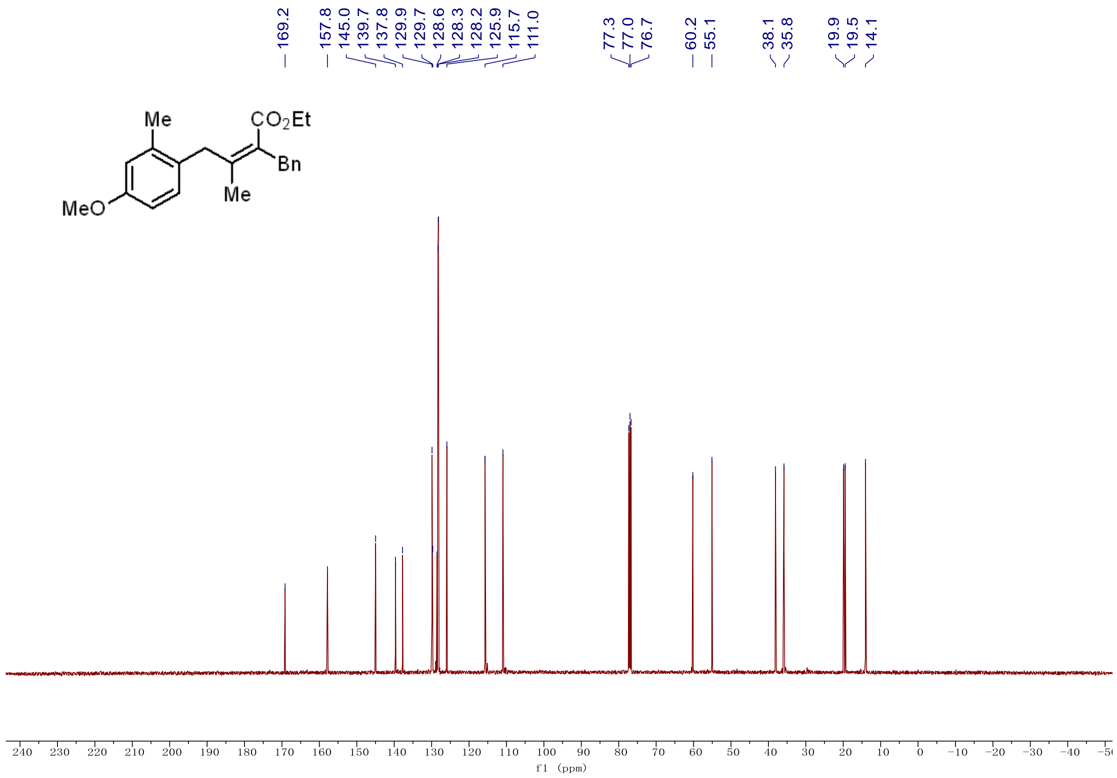

^{13}C NMR spectrum for compound **3g**

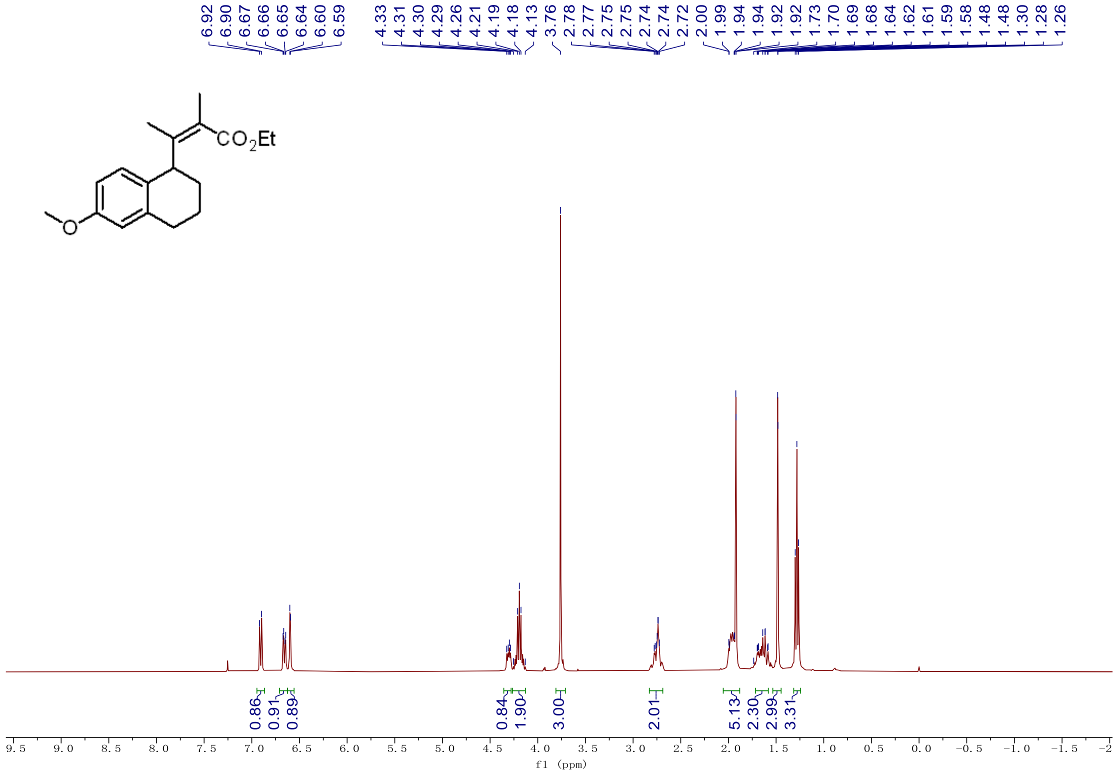

^{1}H NMR spectrum for compound **3h**

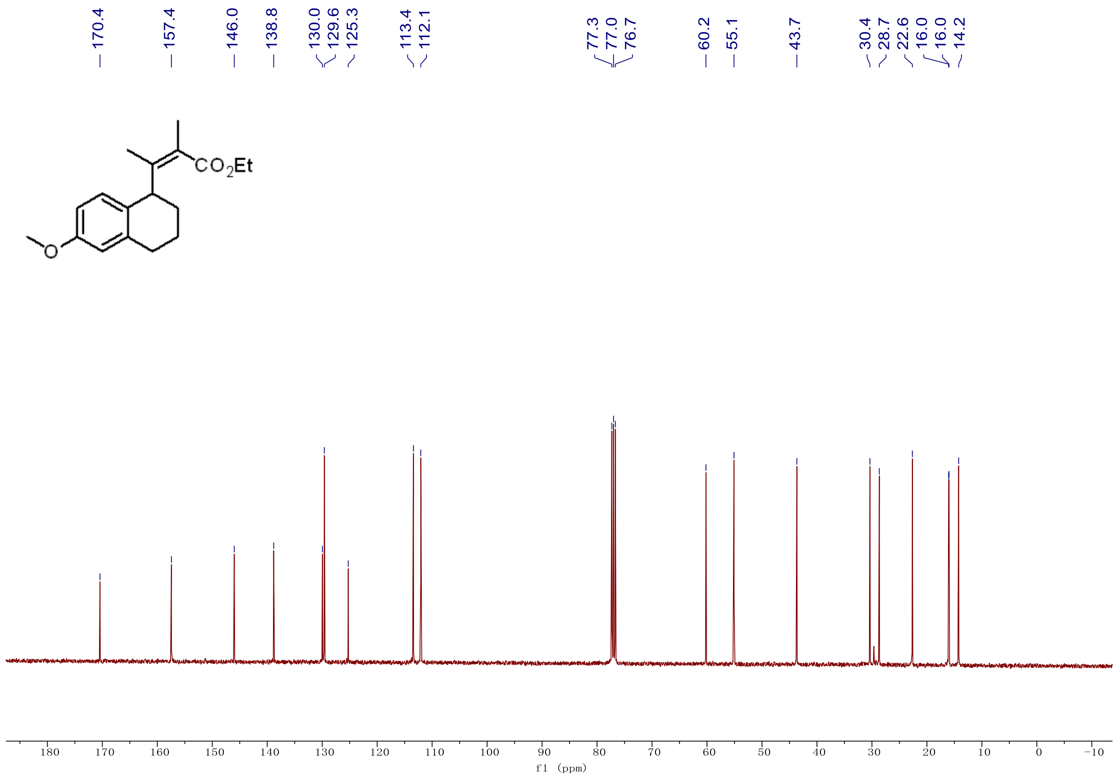

^{13}C NMR spectrum for compound **3h**

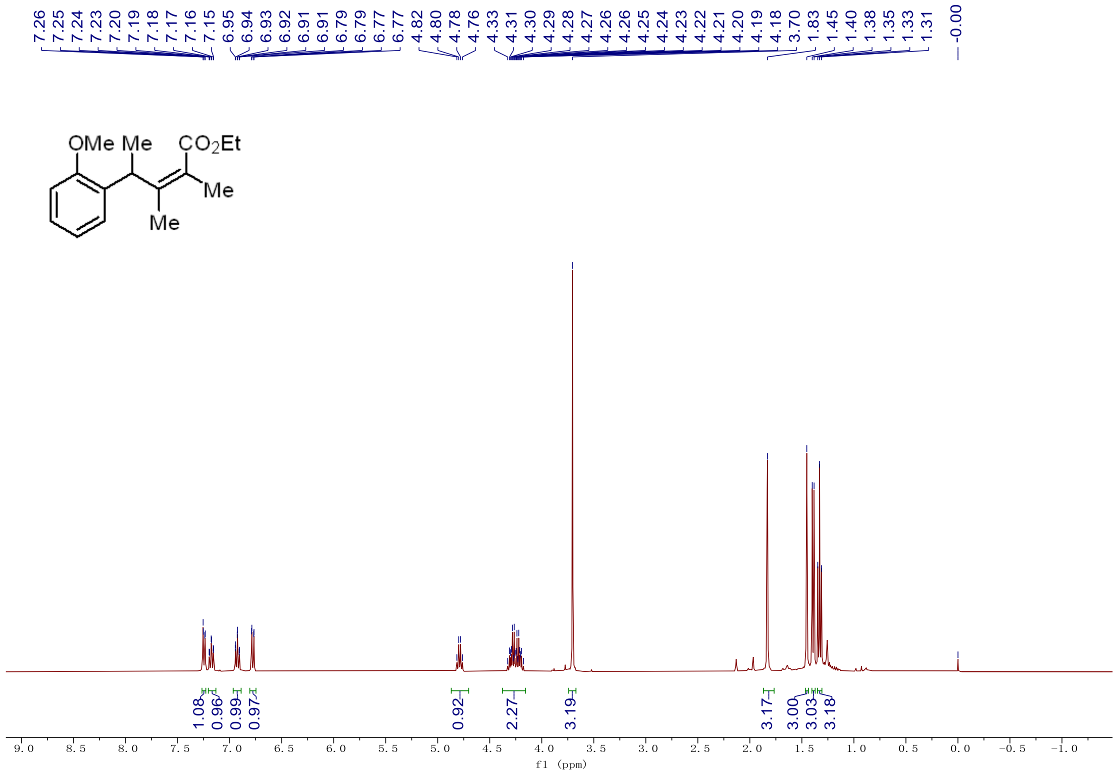

^{1}H NMR spectrum for compound **3i**

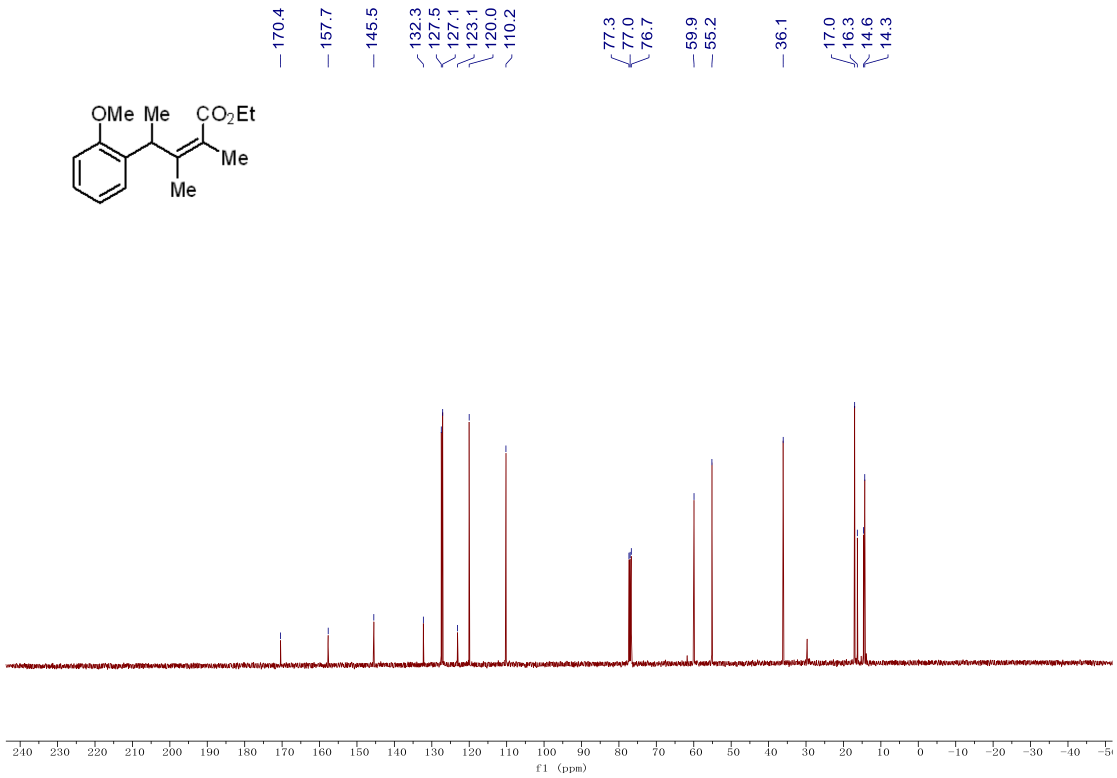

^{13}C NMR spectrum for compound **3i**

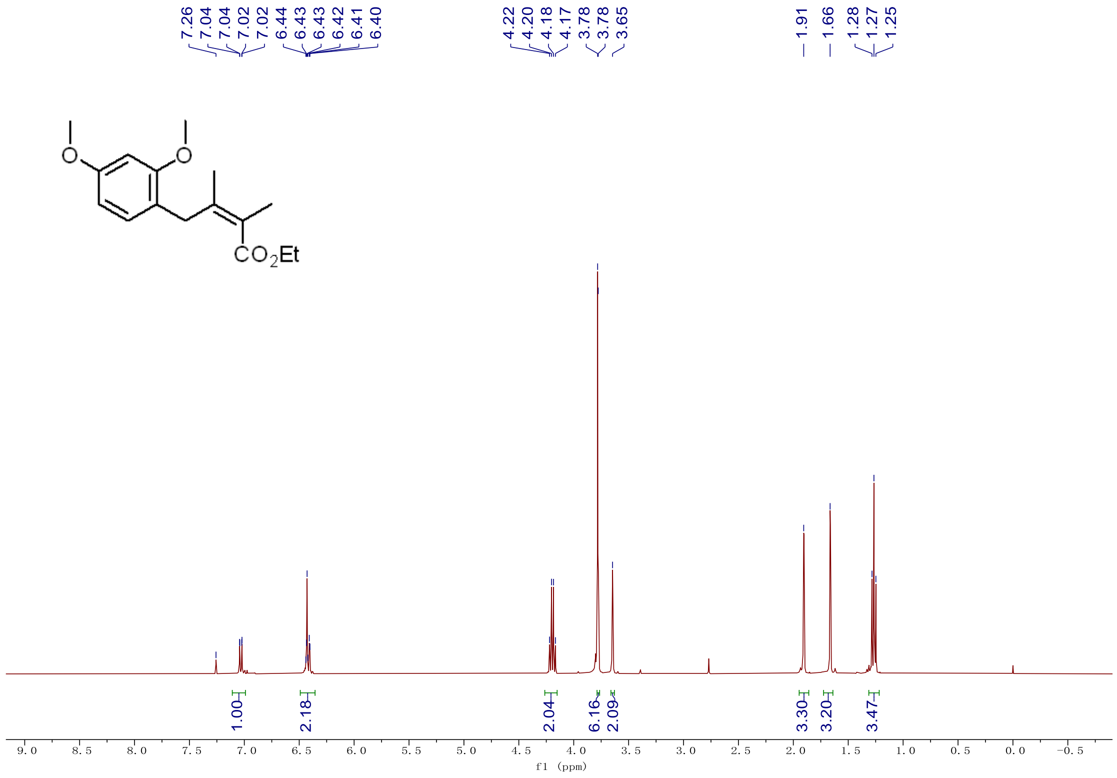

^{1}H NMR spectrum for compound **3j**

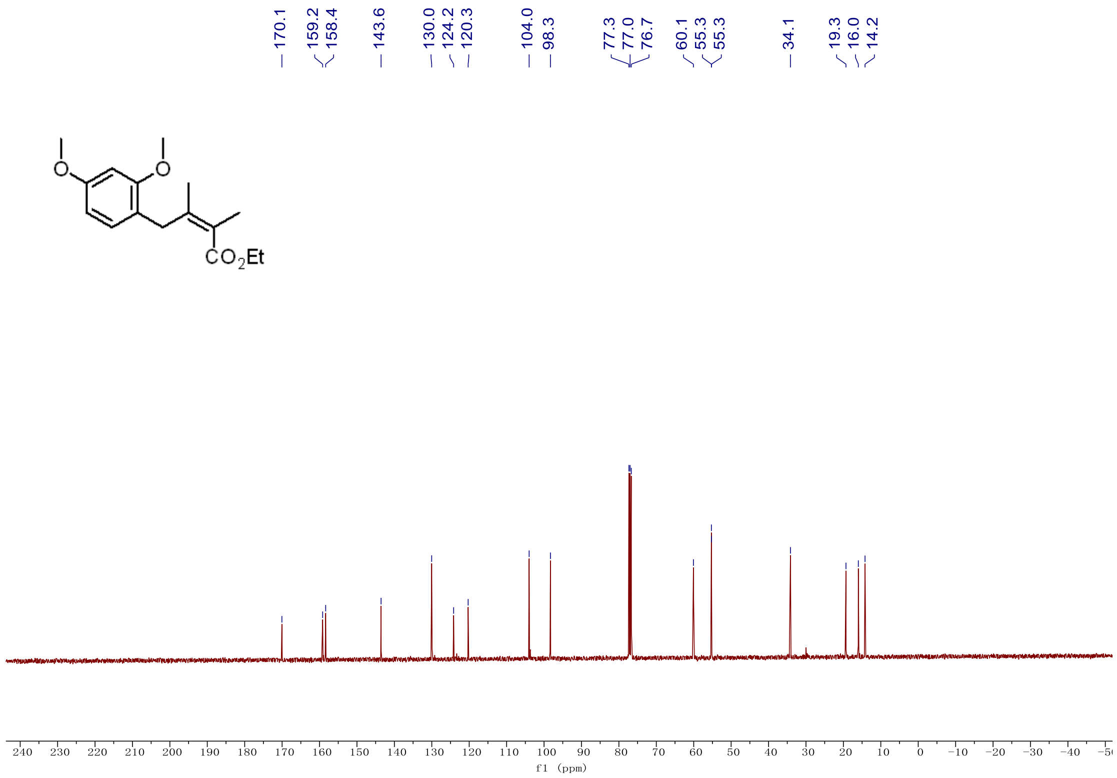

^{13}C NMR spectrum for compound **3j**

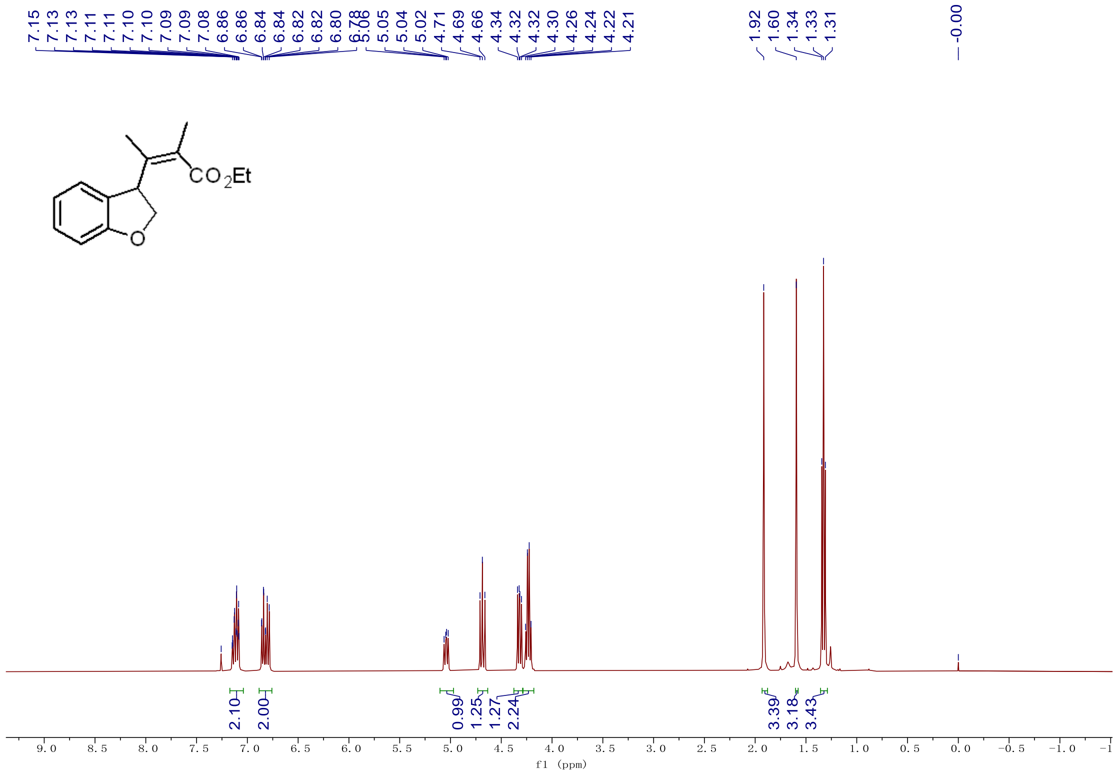

^{1}H NMR spectrum for compound **3k**

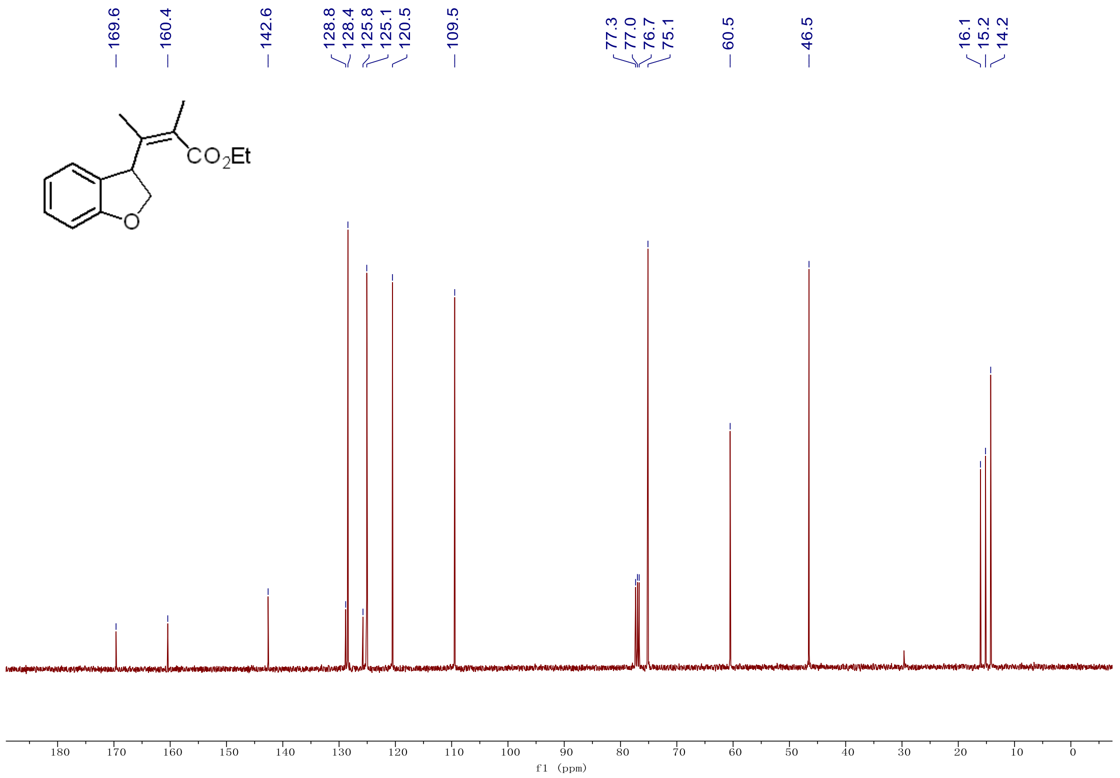

^{13}C NMR spectrum for compound **3**

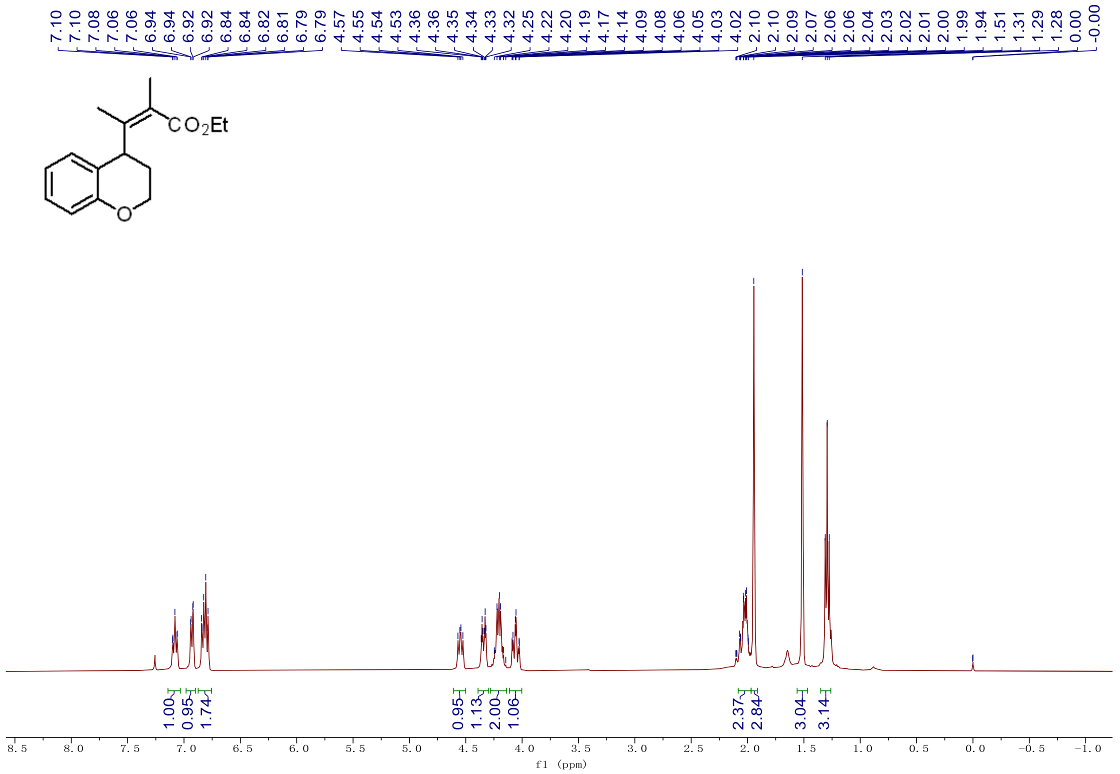

^{1}H NMR spectrum for compound **3l**

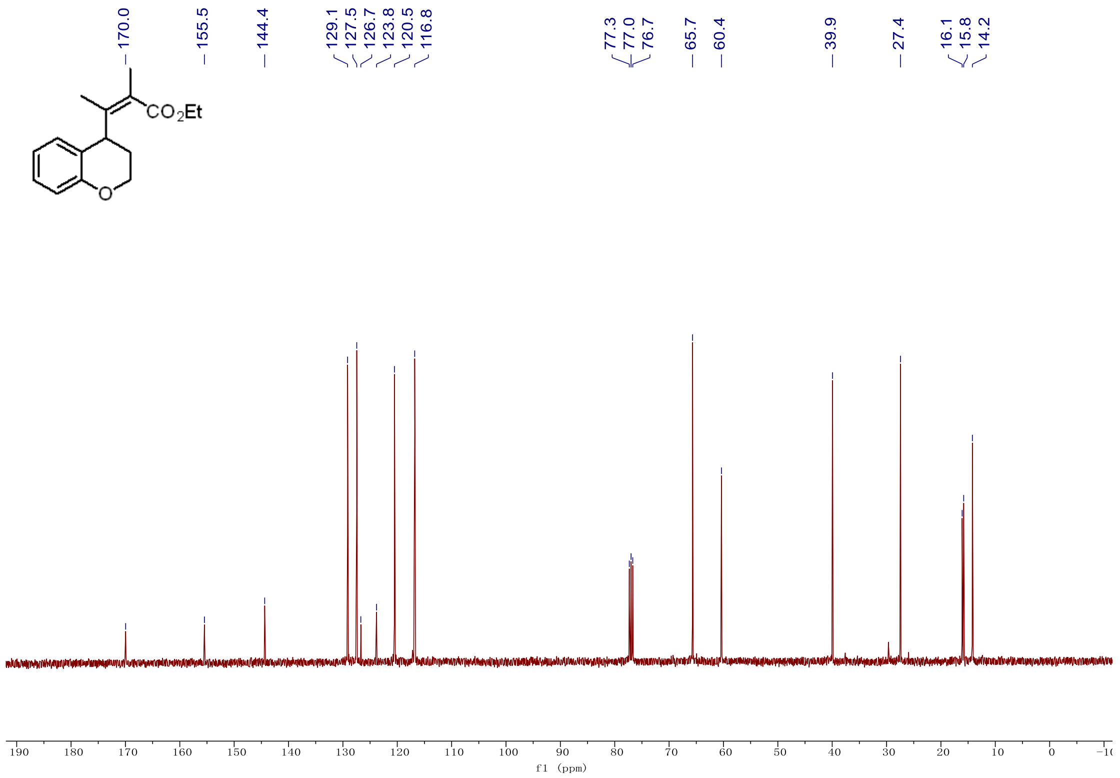

^{13}C NMR spectrum for compound **3l**

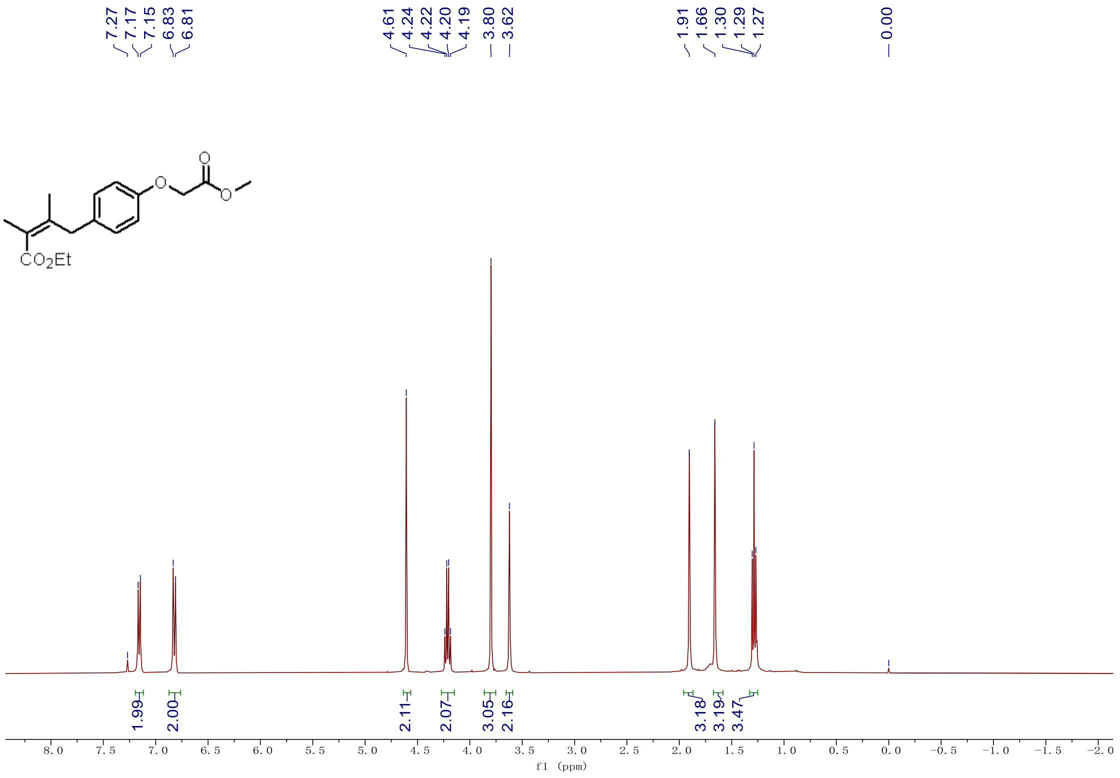

^{1}H NMR spectrum for compound **3m**

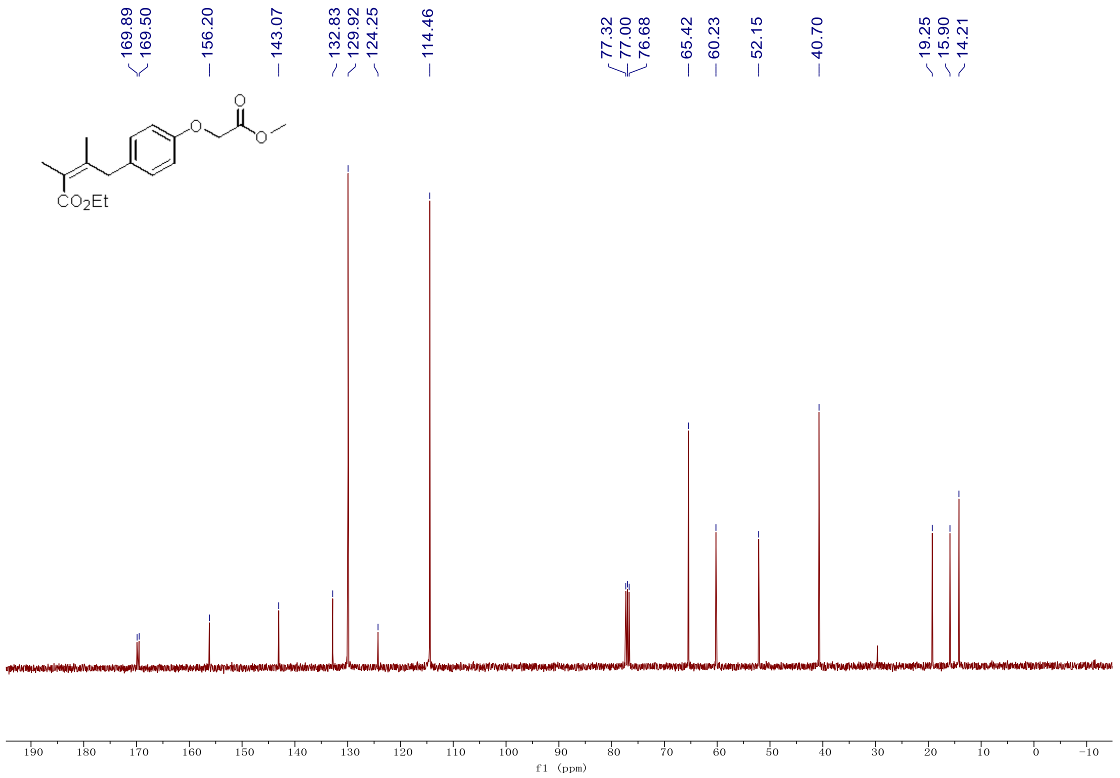

^{13}C NMR spectrum for compound **3m**

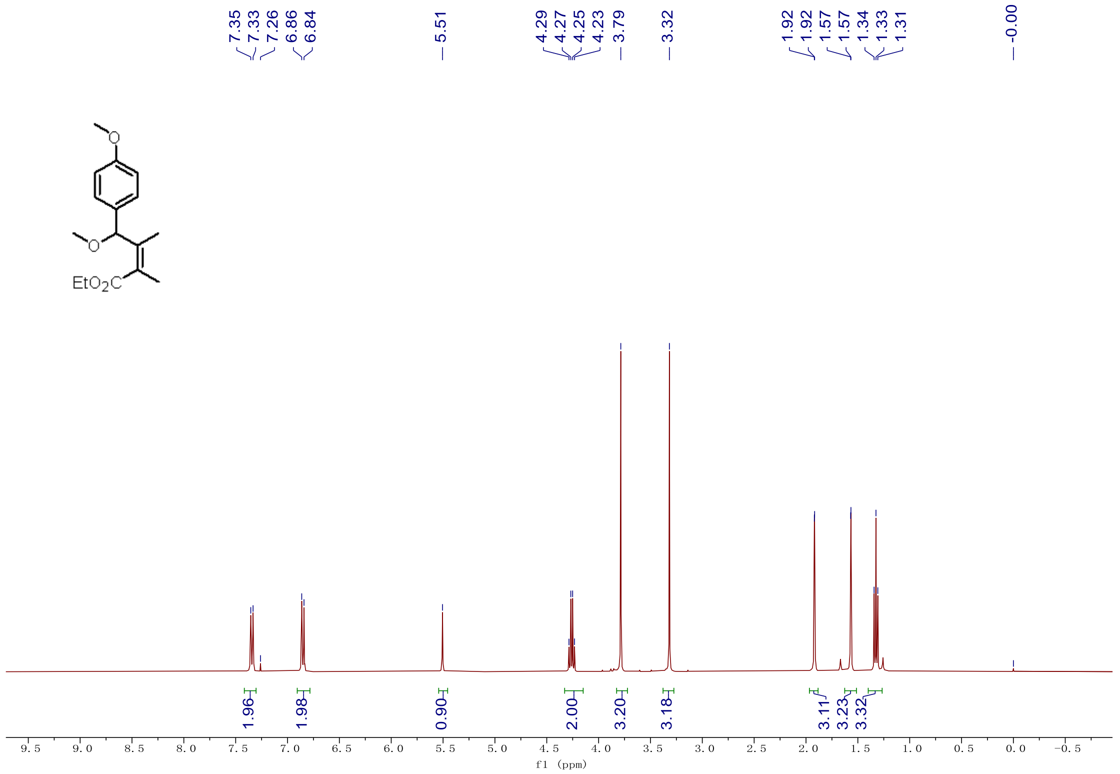

^{1}H NMR spectrum for compound **3n**

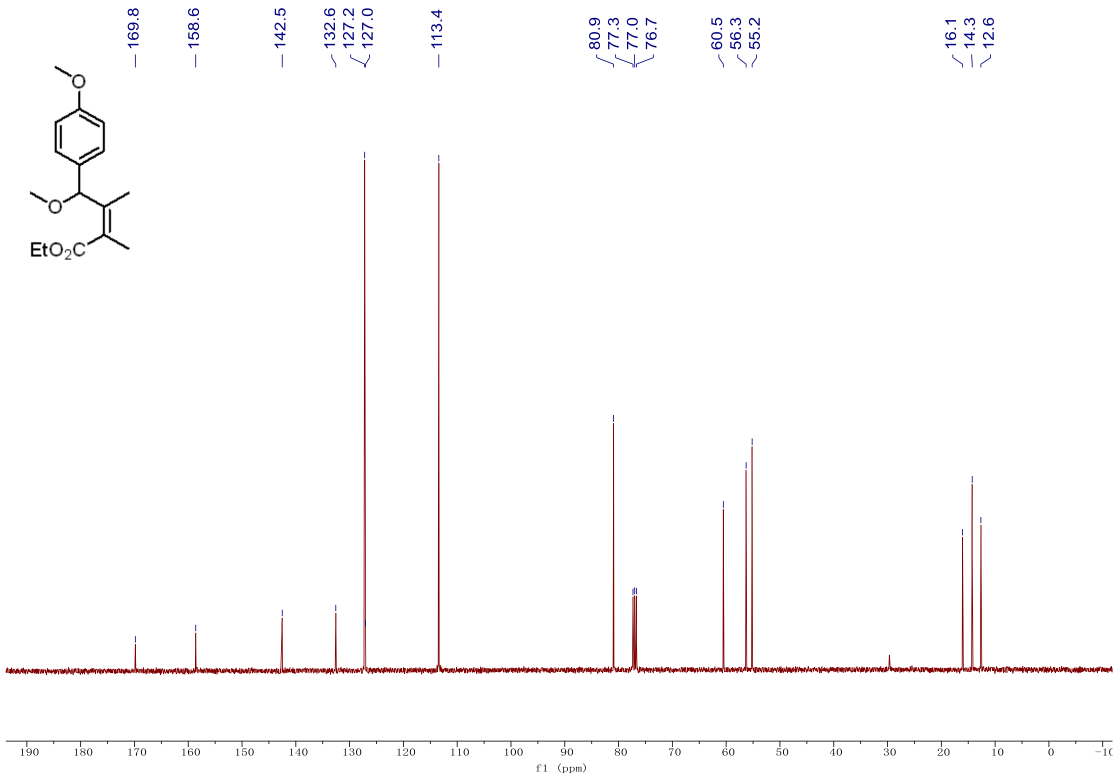

^{13}C NMR spectrum for compound **3n**

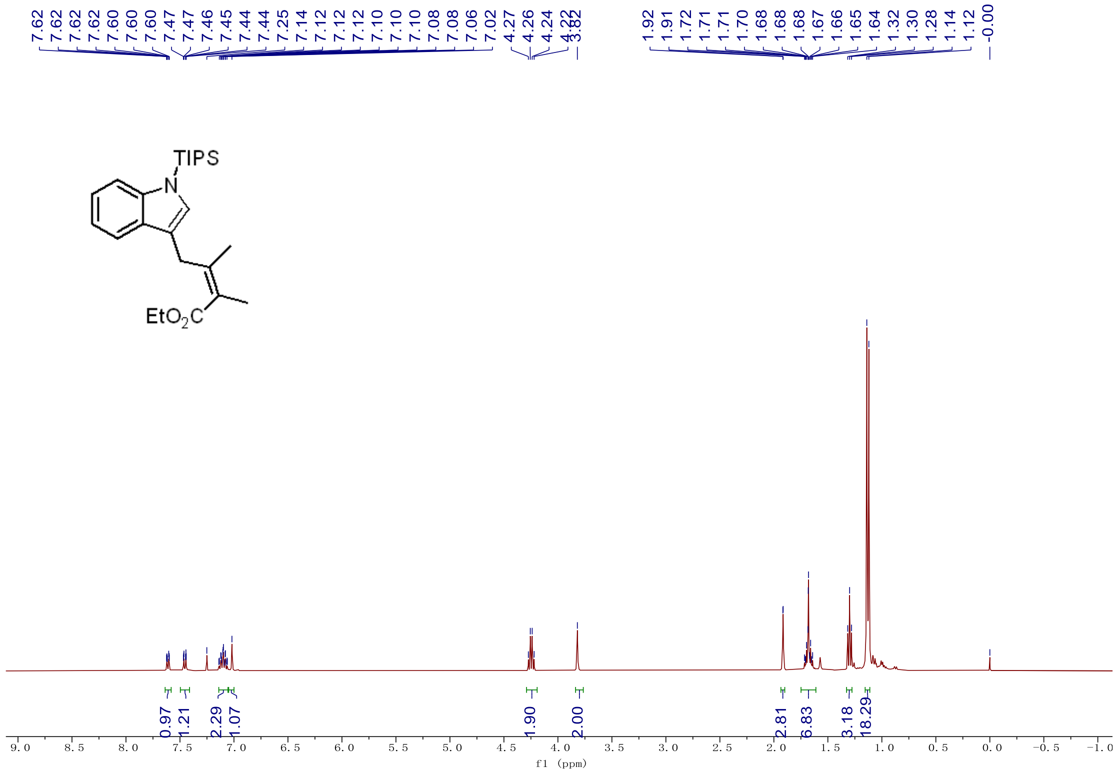

^{1}H NMR spectrum for compound **3o**

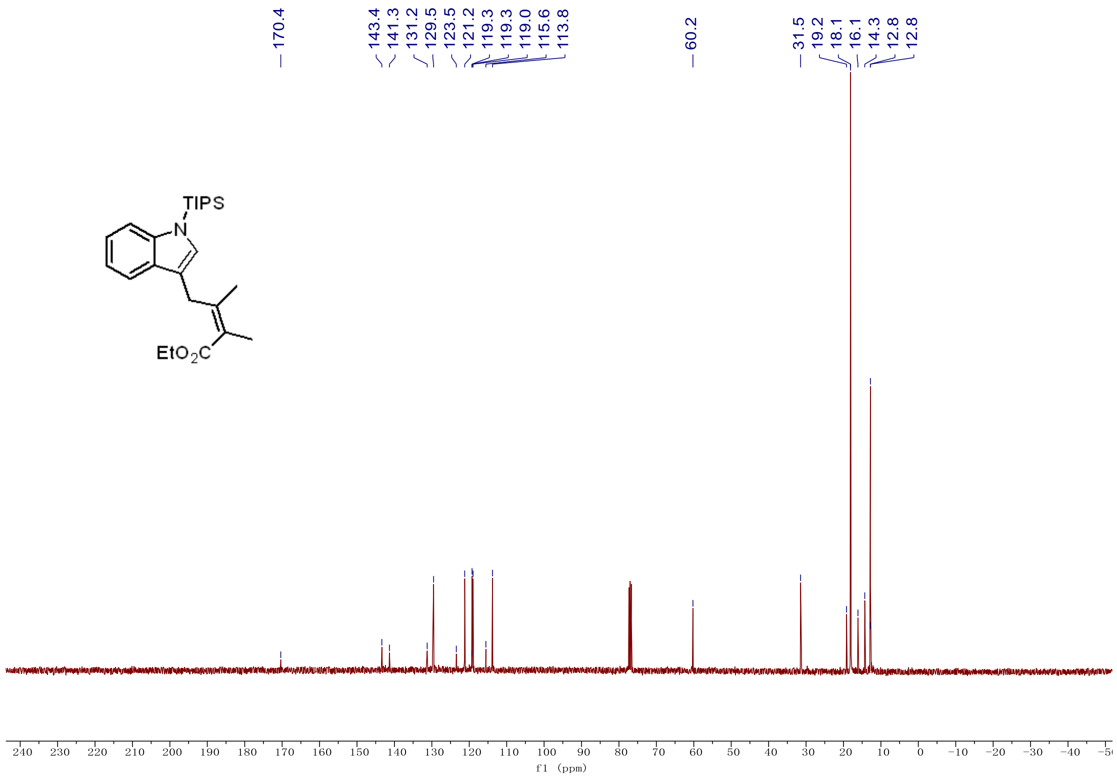

^{13}C NMR spectrum for compound **3o**

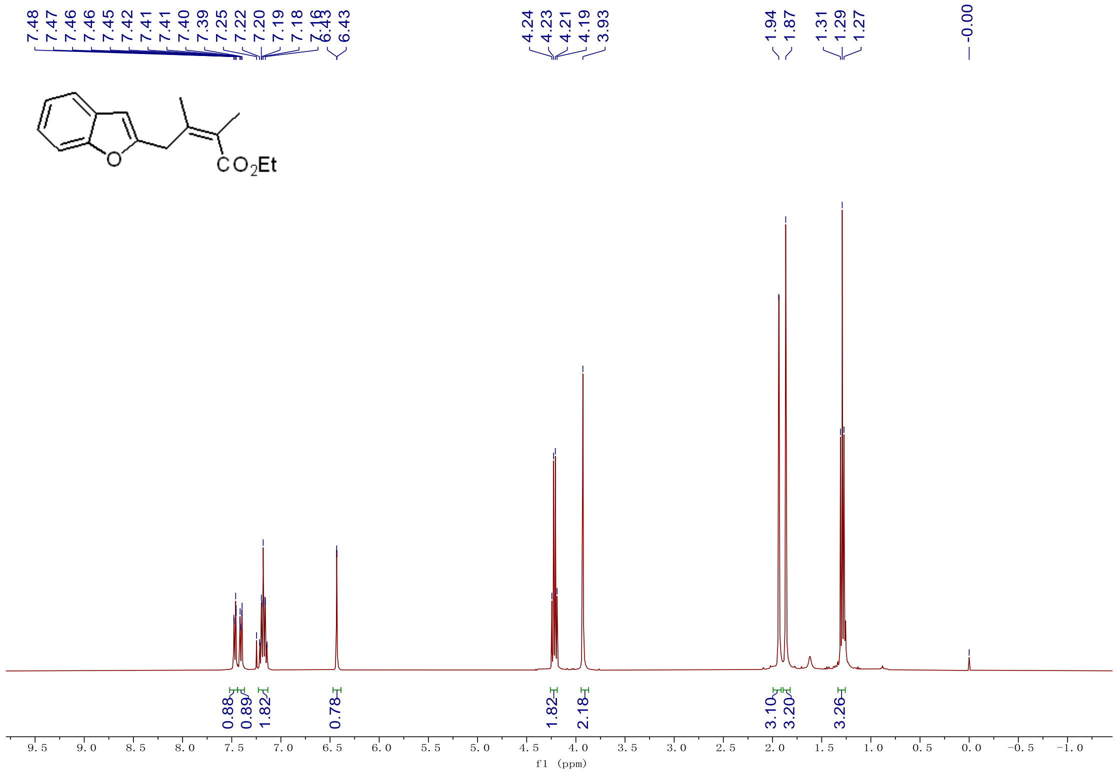

^{1}H NMR spectrum for compound **3p**

·

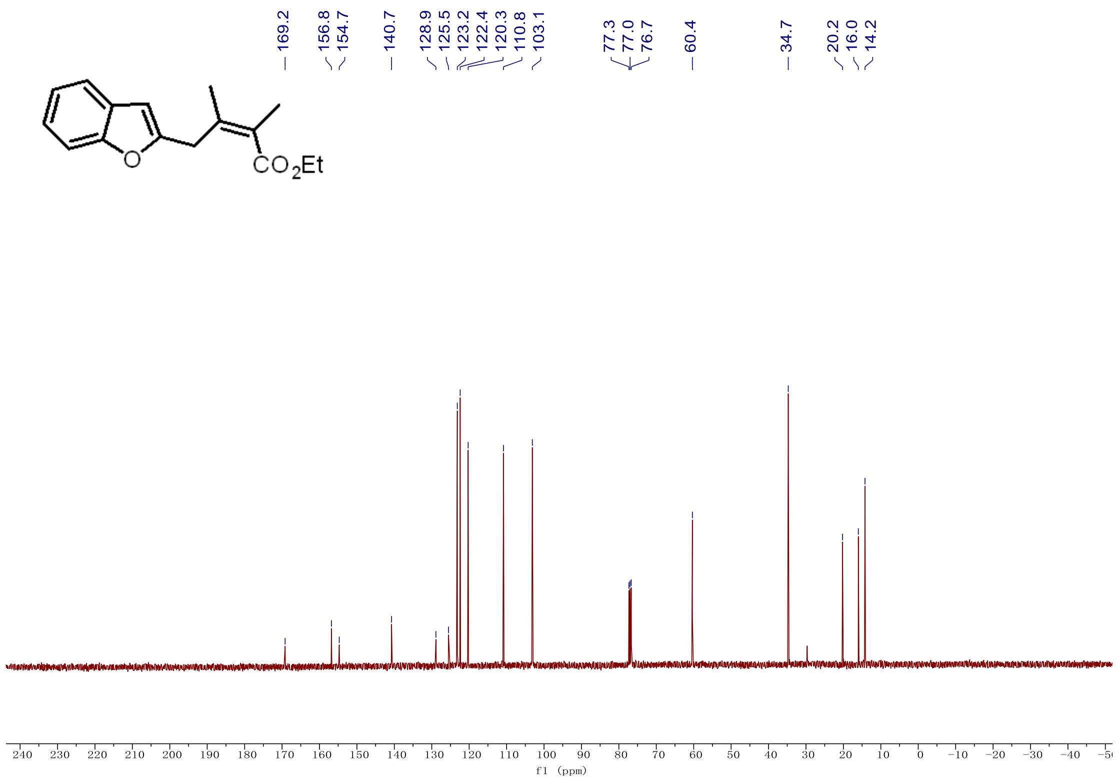

^{13}C NMR spectrum for compound **3p**

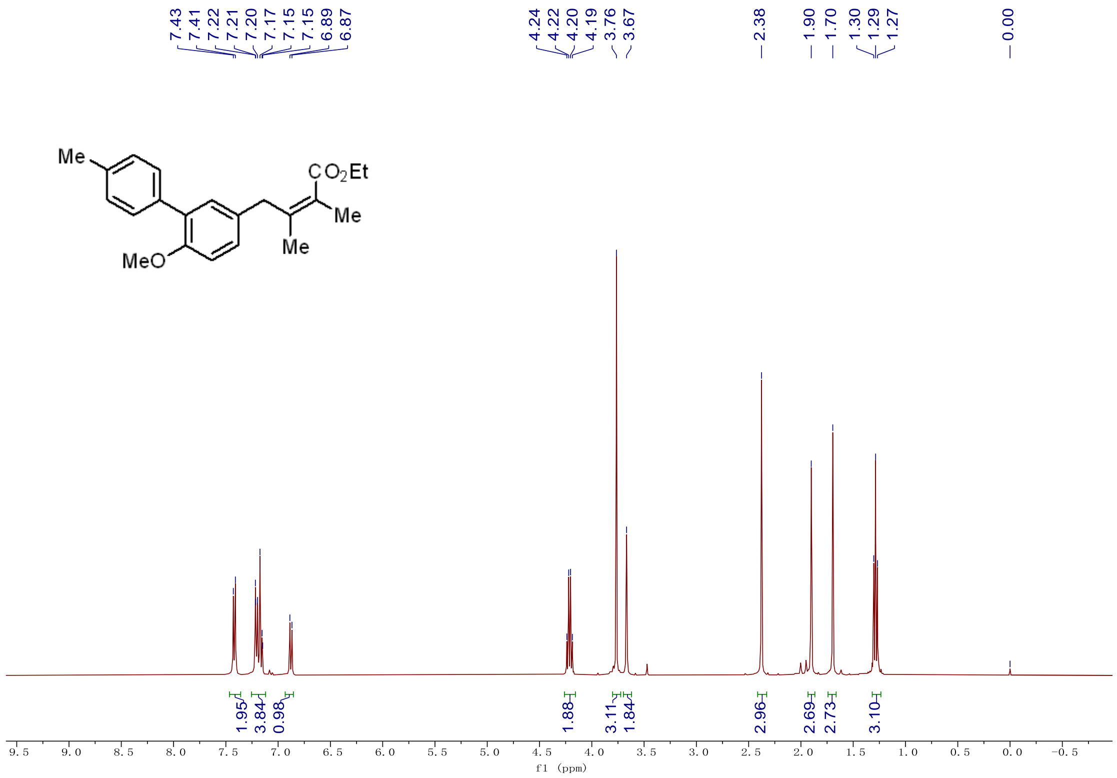

^{1}H NMR spectrum for compound **3q**

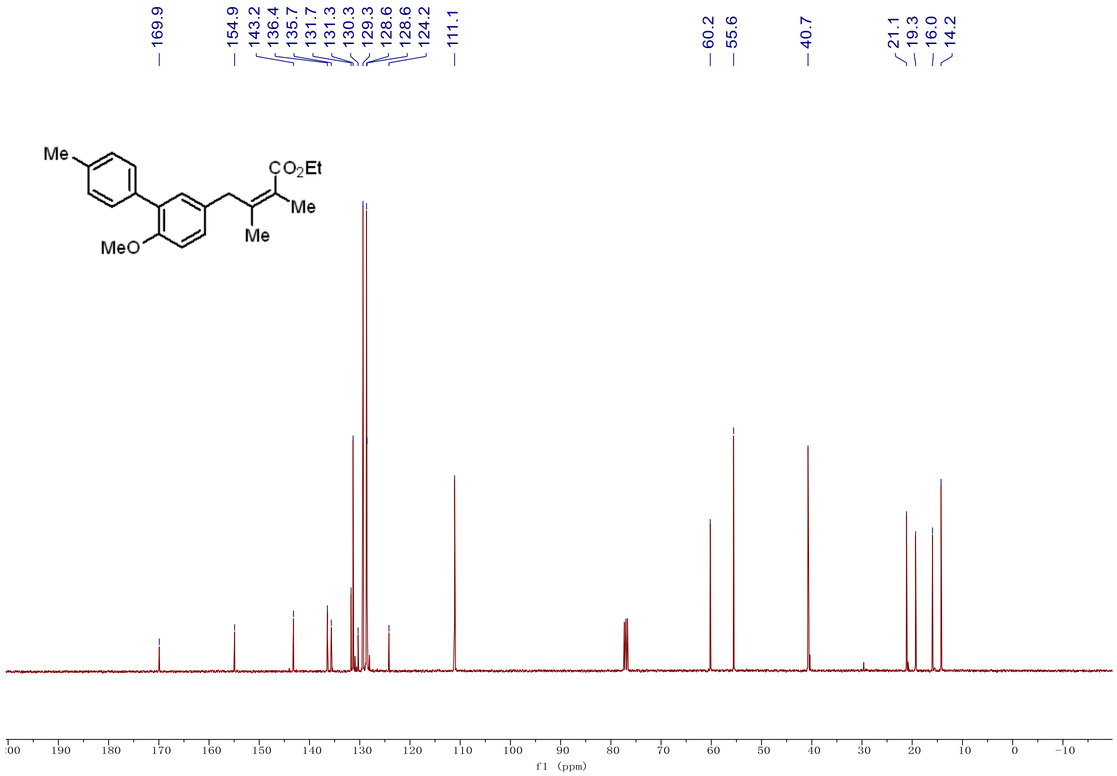

^{13}C NMR spectrum for compound **3q**

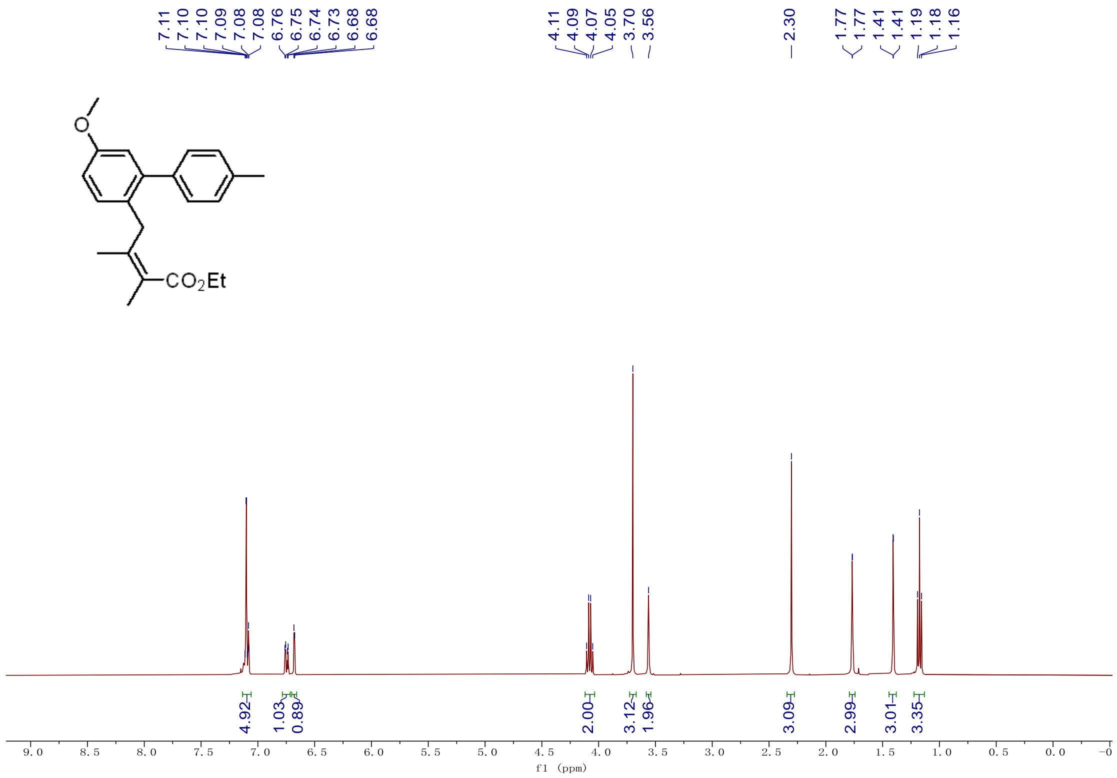

^{1}H NMR spectrum for compound **3r**

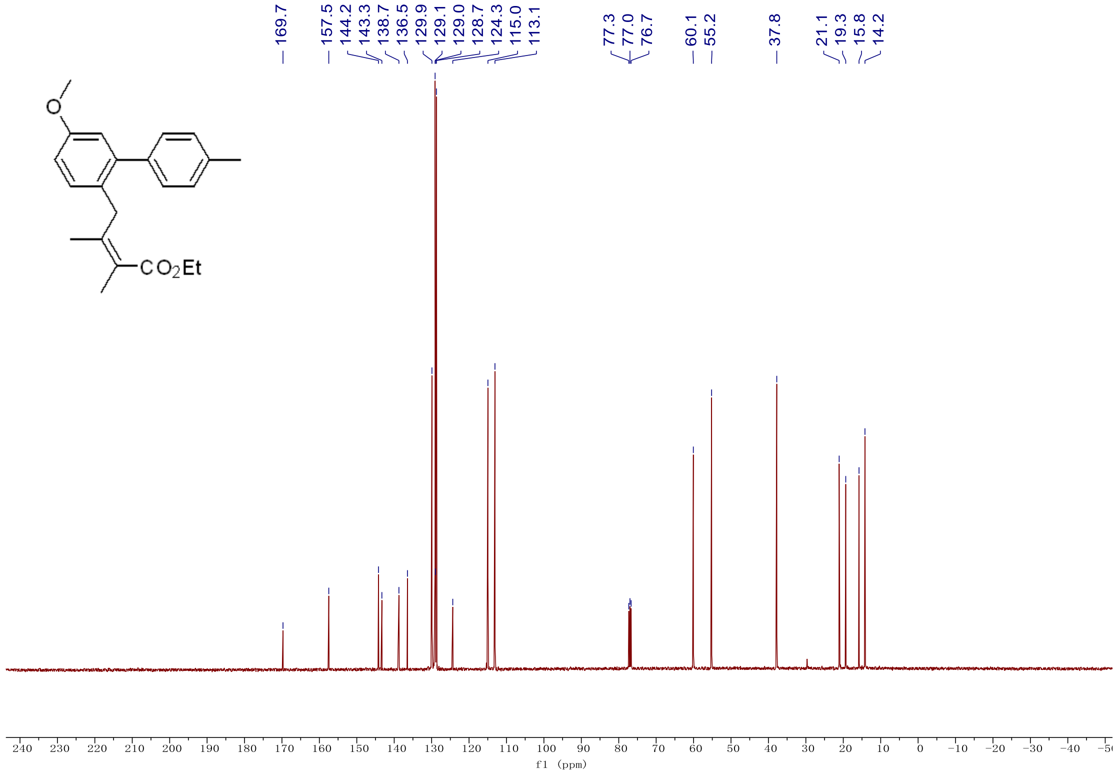

^{13}C NMR spectrum for compound **3r**

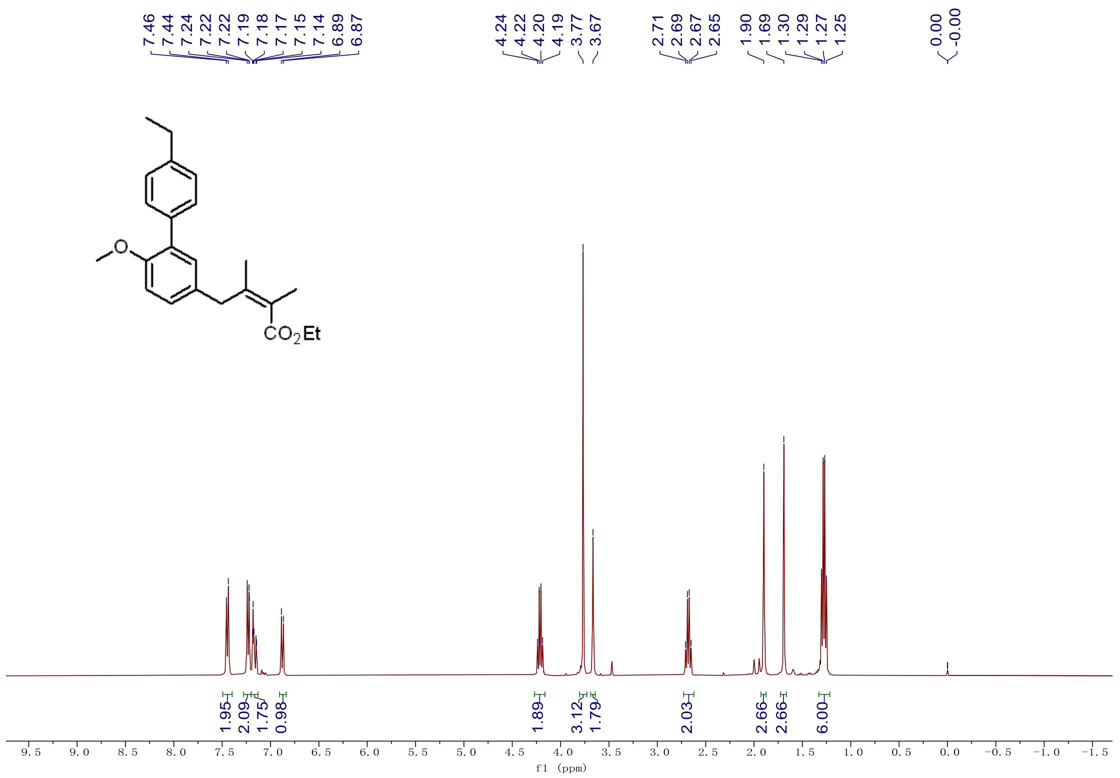

^{1}H NMR spectrum for compound **3s**

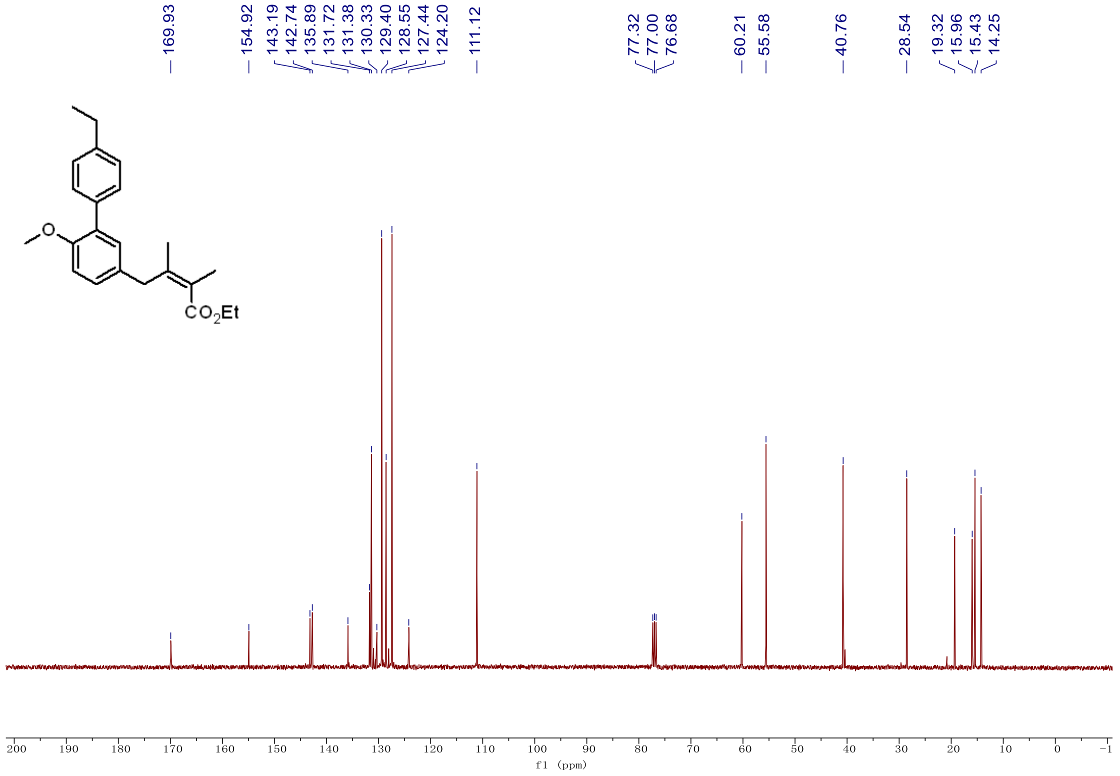

^{13}C NMR spectrum for compound **3s**

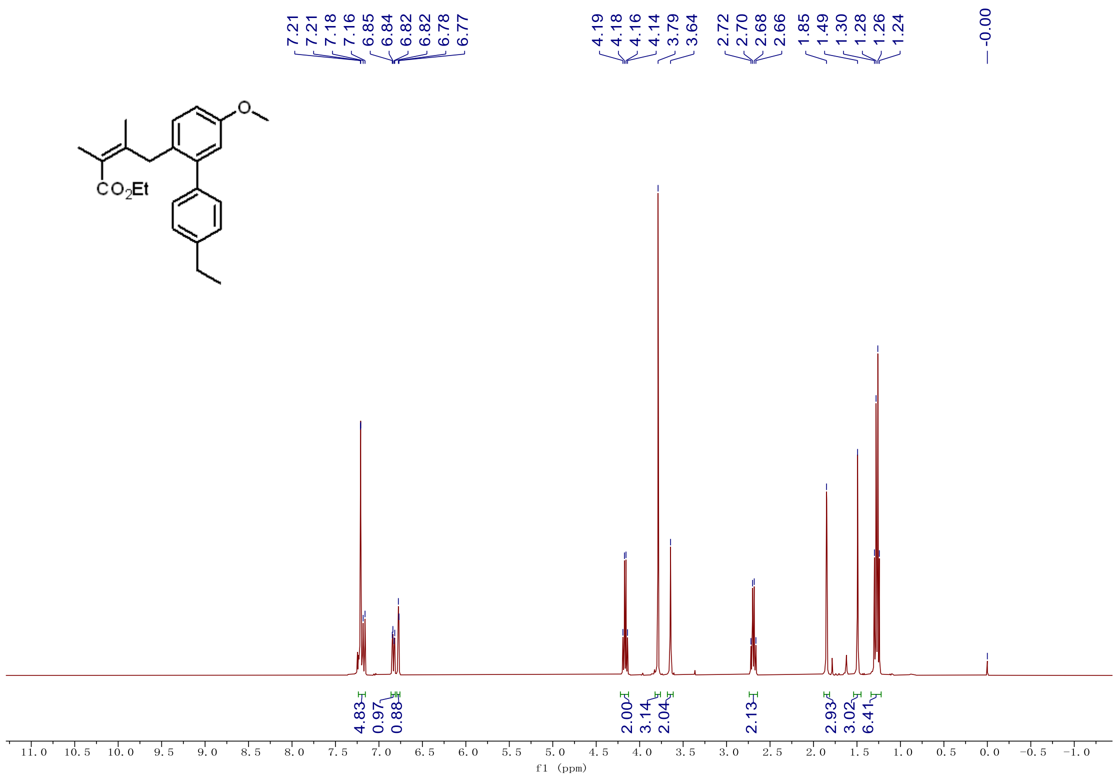

^1H NMR spectrum for compound **3t**

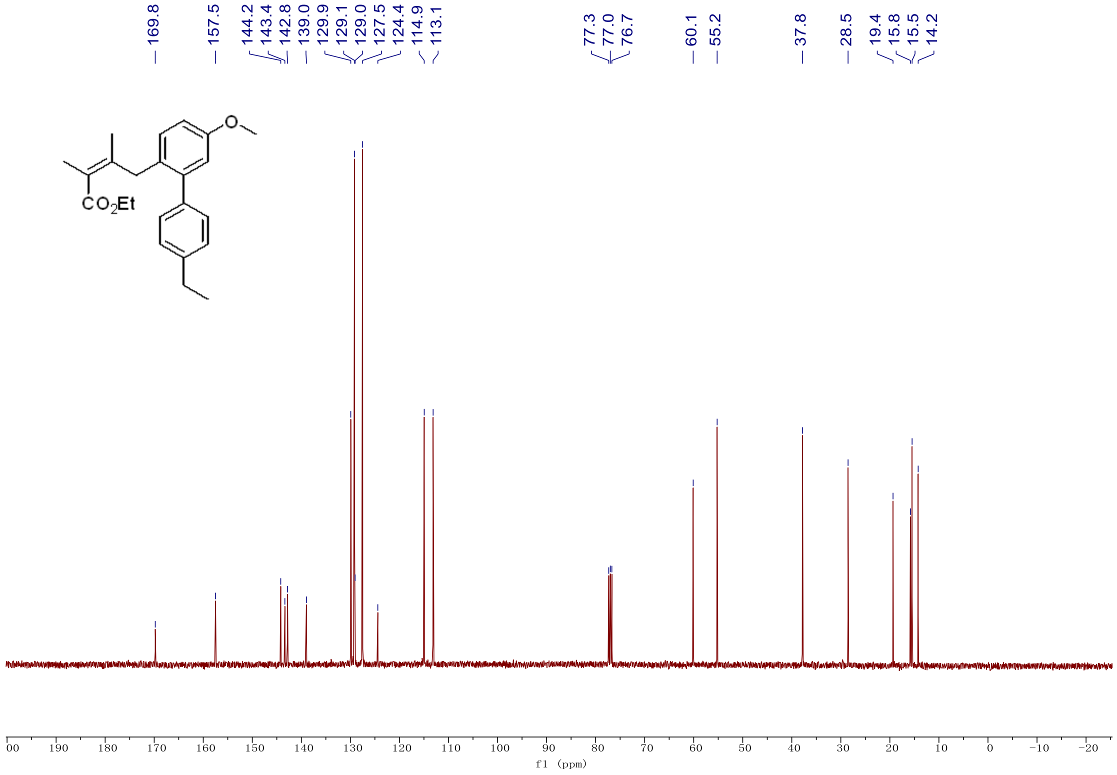

^{13}C NMR spectrum for compound **3t**

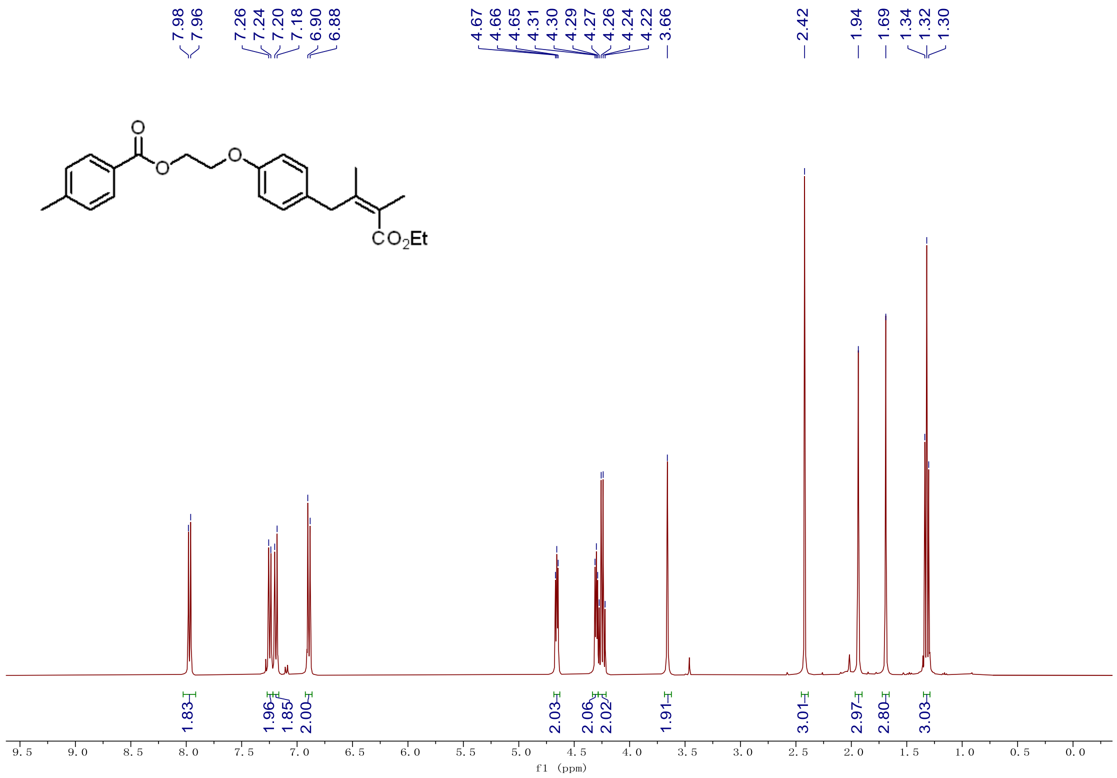

^{1}H NMR spectrum for compound **3u**

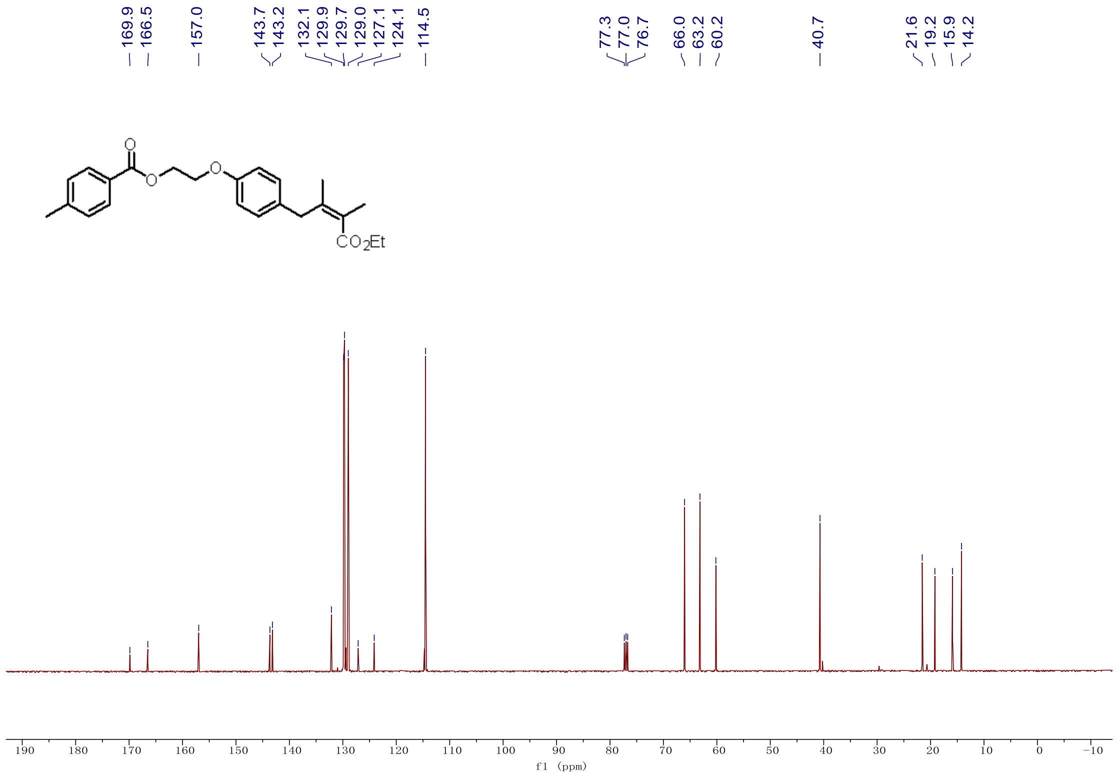

^{13}C NMR spectrum for compound **3u**

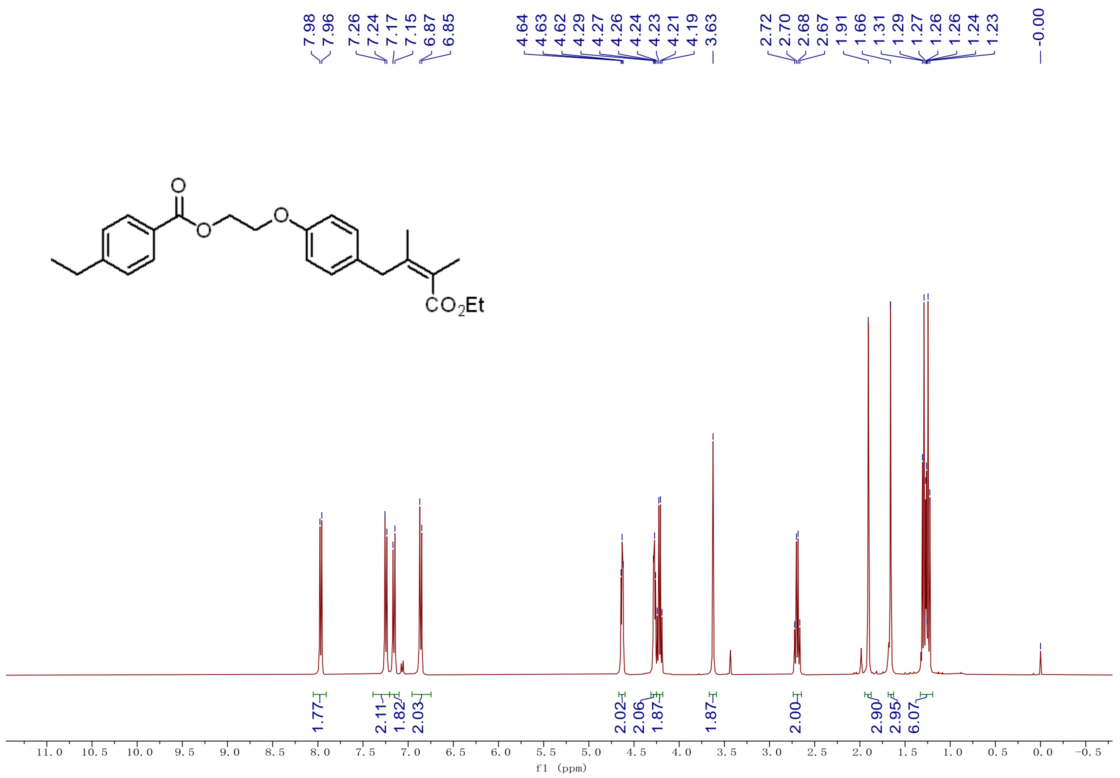

^{1}H NMR spectrum for compound **3v**

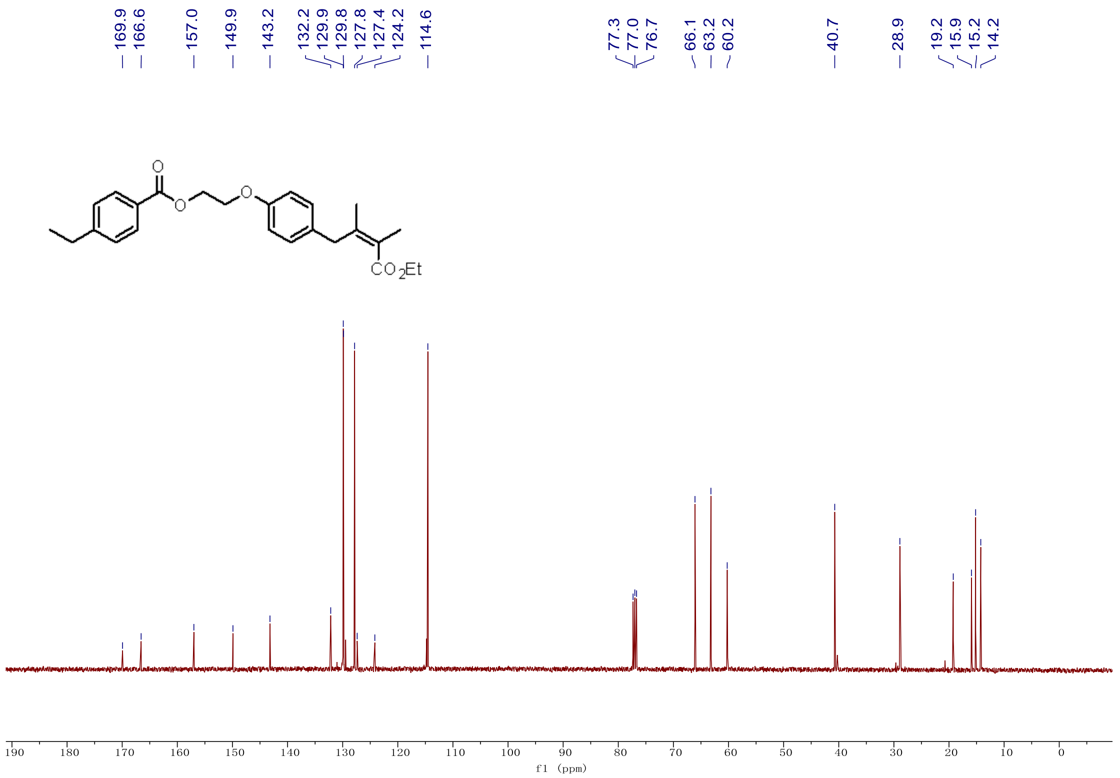

^{13}C NMR spectrum for compound **3v**

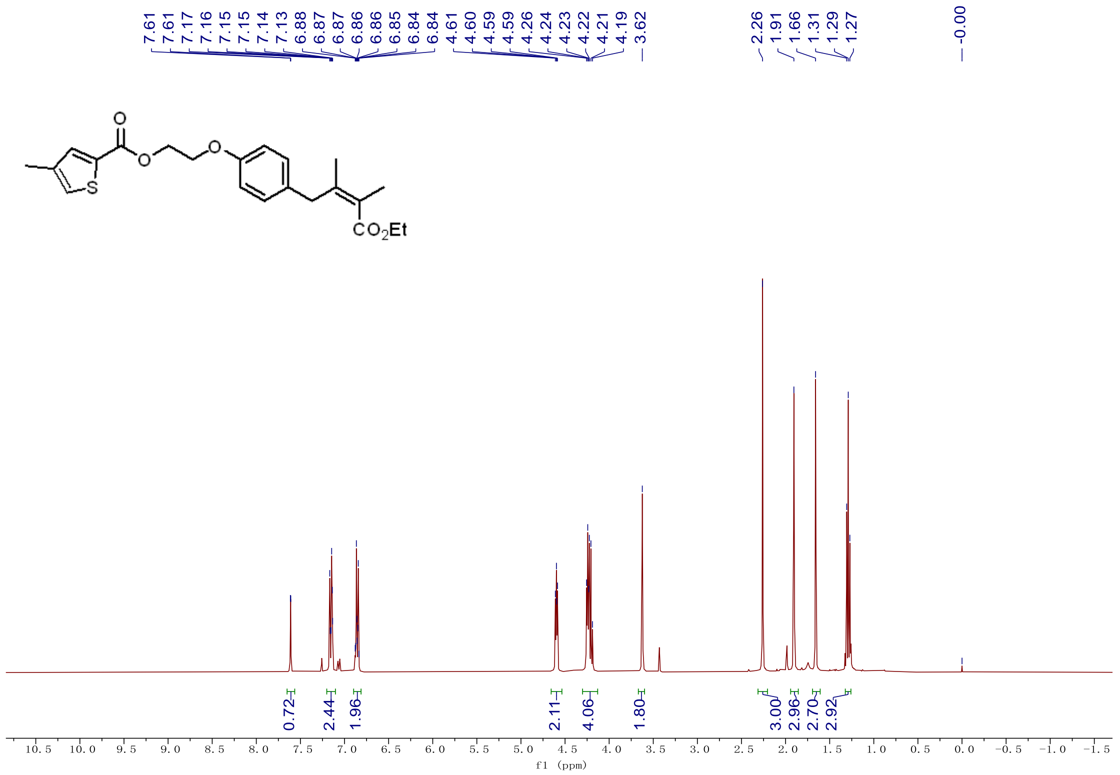

^{1}H NMR spectrum for compound **3w**

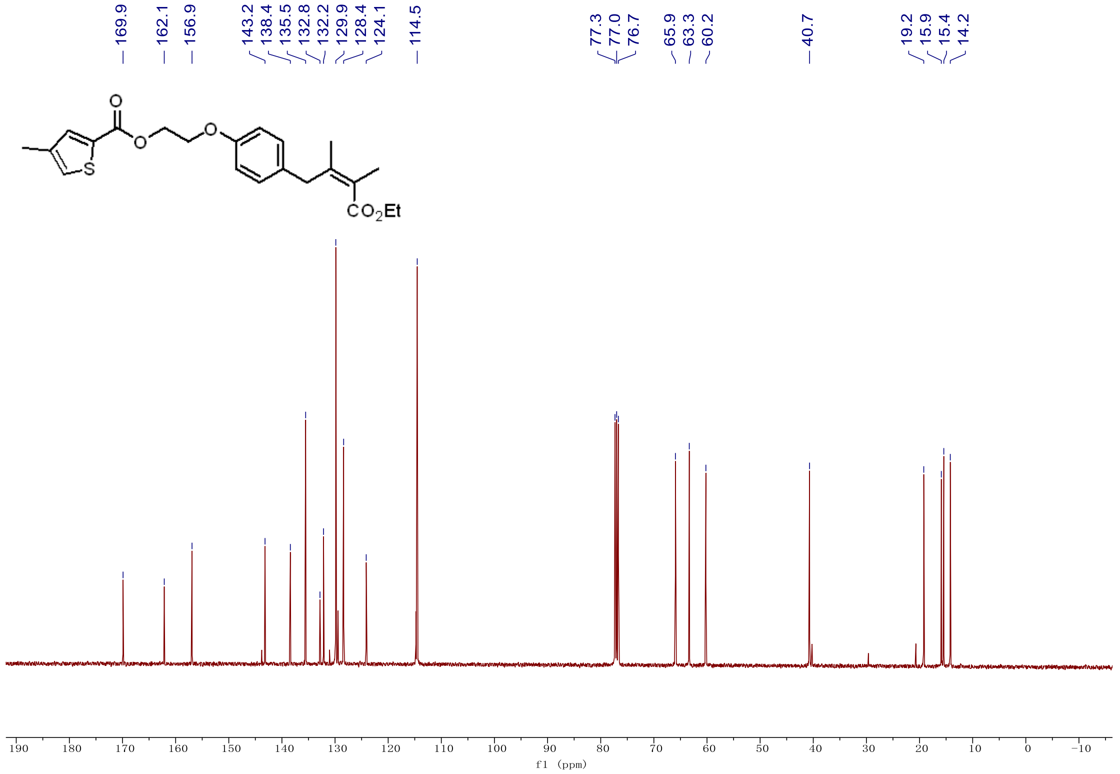

^{13}C NMR spectrum for compound **3w**

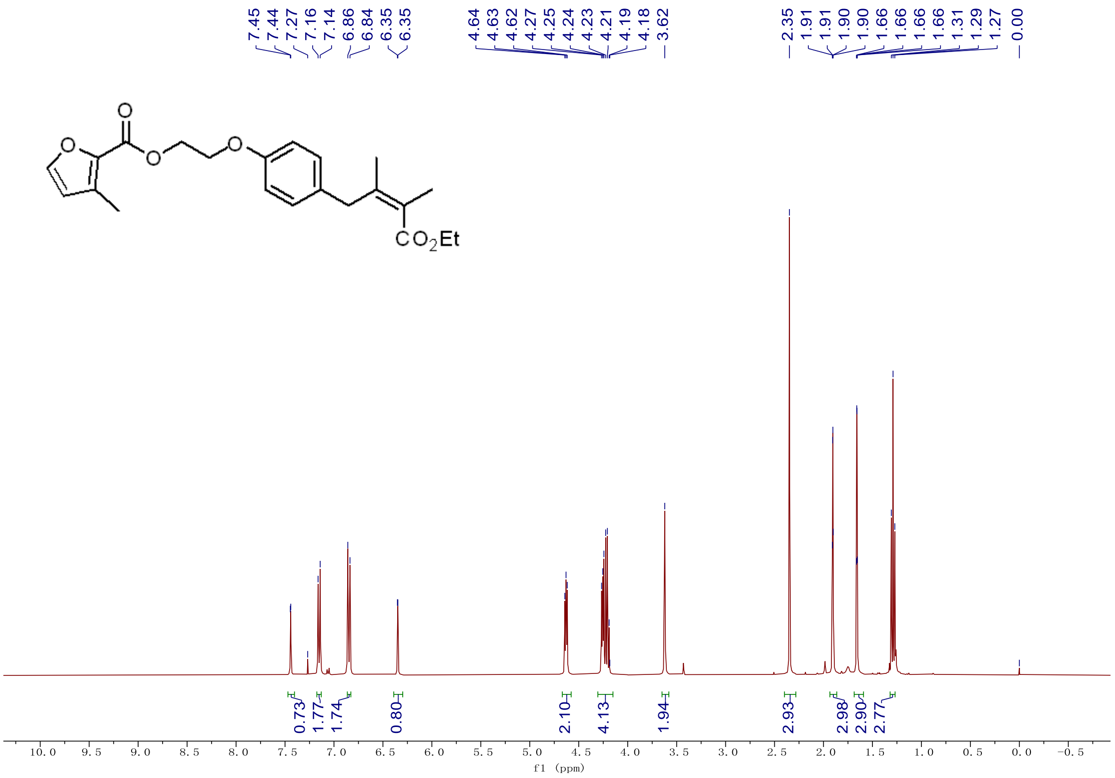

^{1}H NMR spectrum for compound **3x**

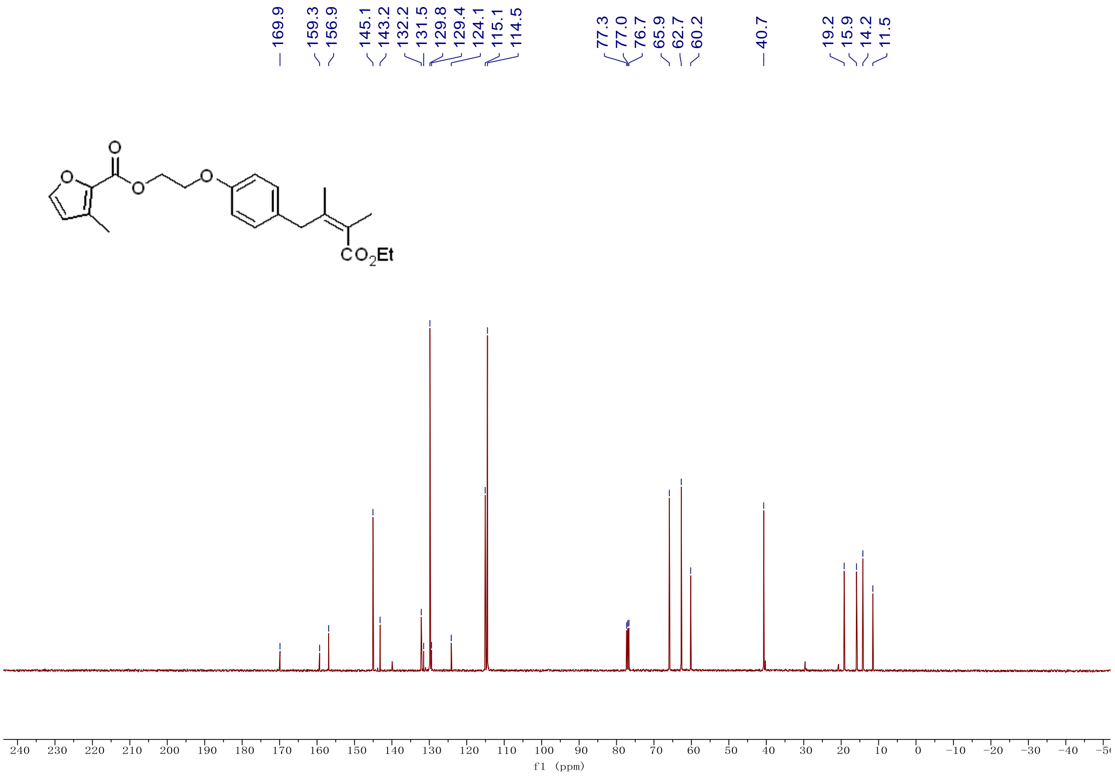

^{13}C NMR spectrum for compound **3x**

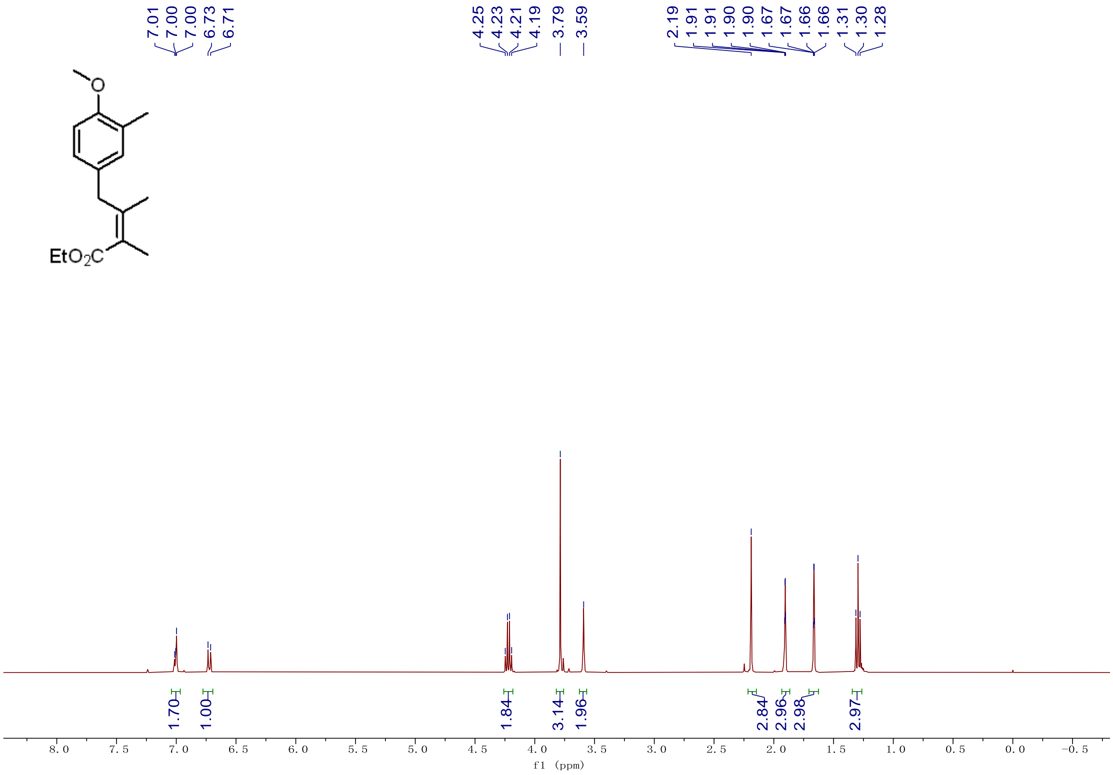

^{1}H NMR spectrum for compound **3y**

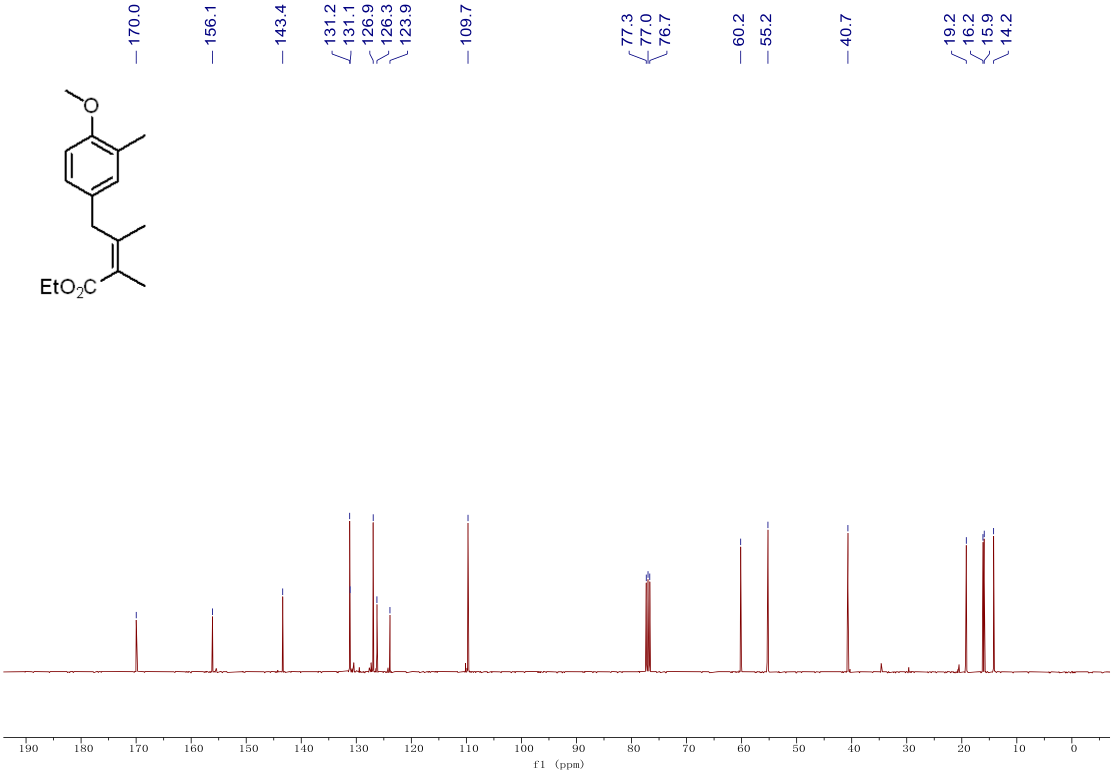

^{13}C NMR spectrum for compound **3y**

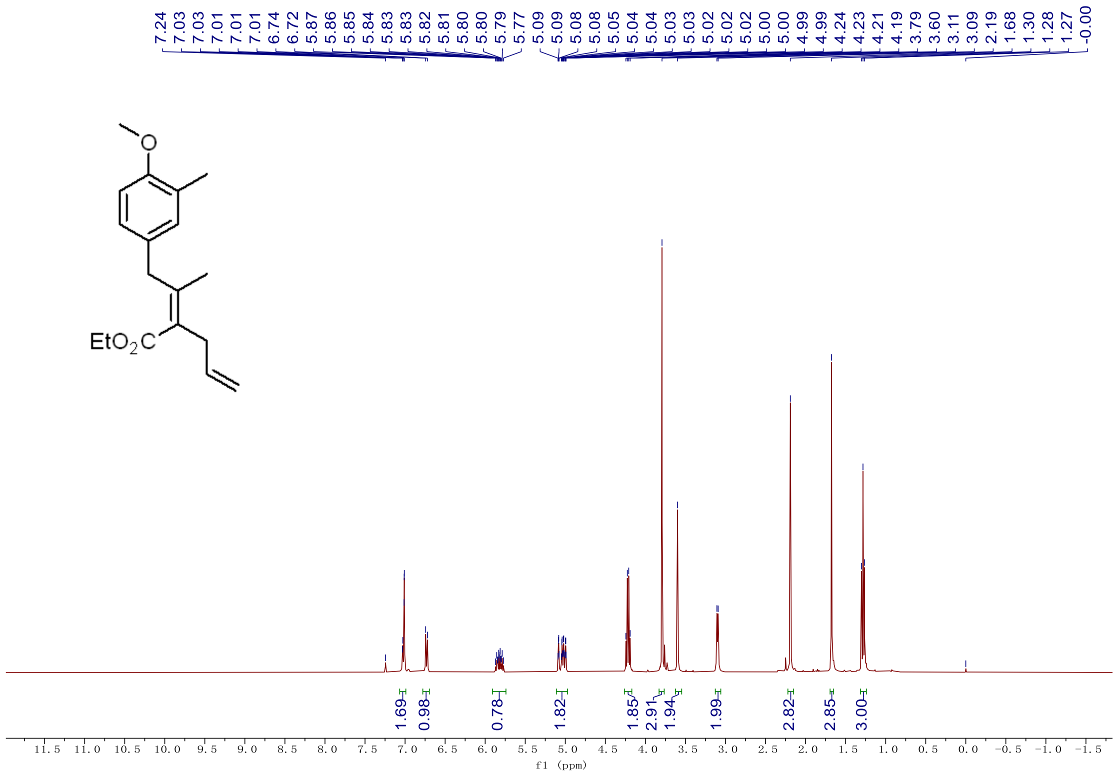

^{1}H NMR spectrum for compound **3z**

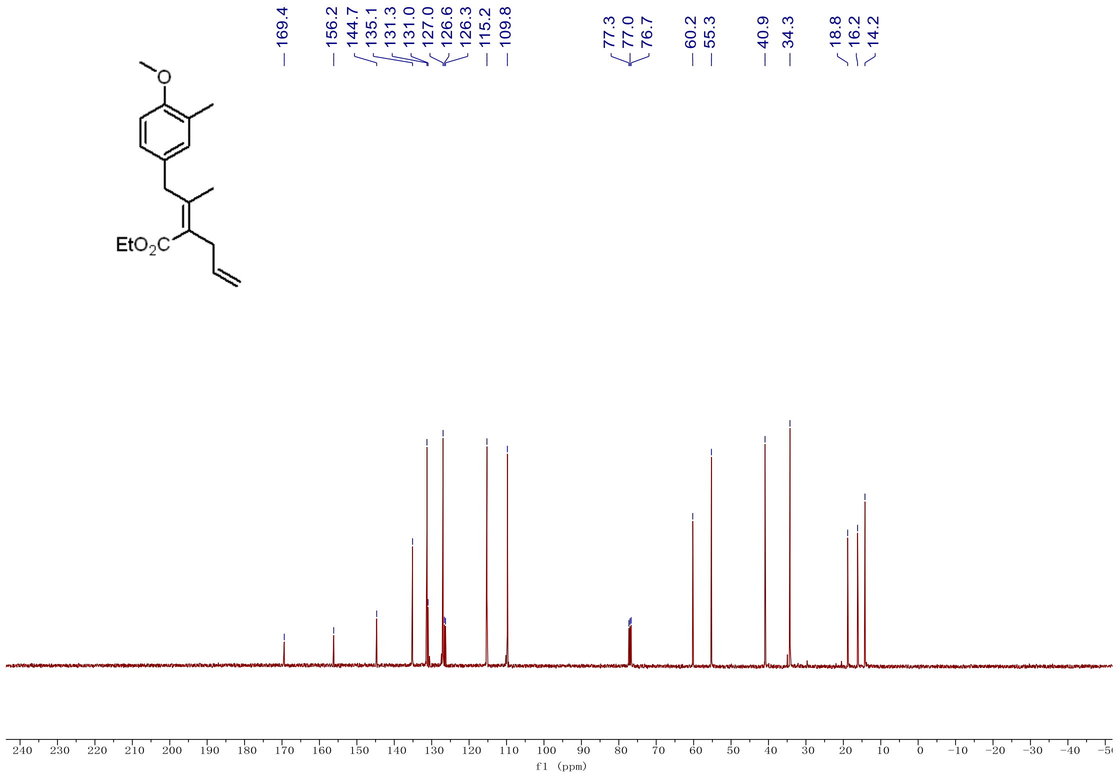

^{13}C NMR spectrum for compound **3z**

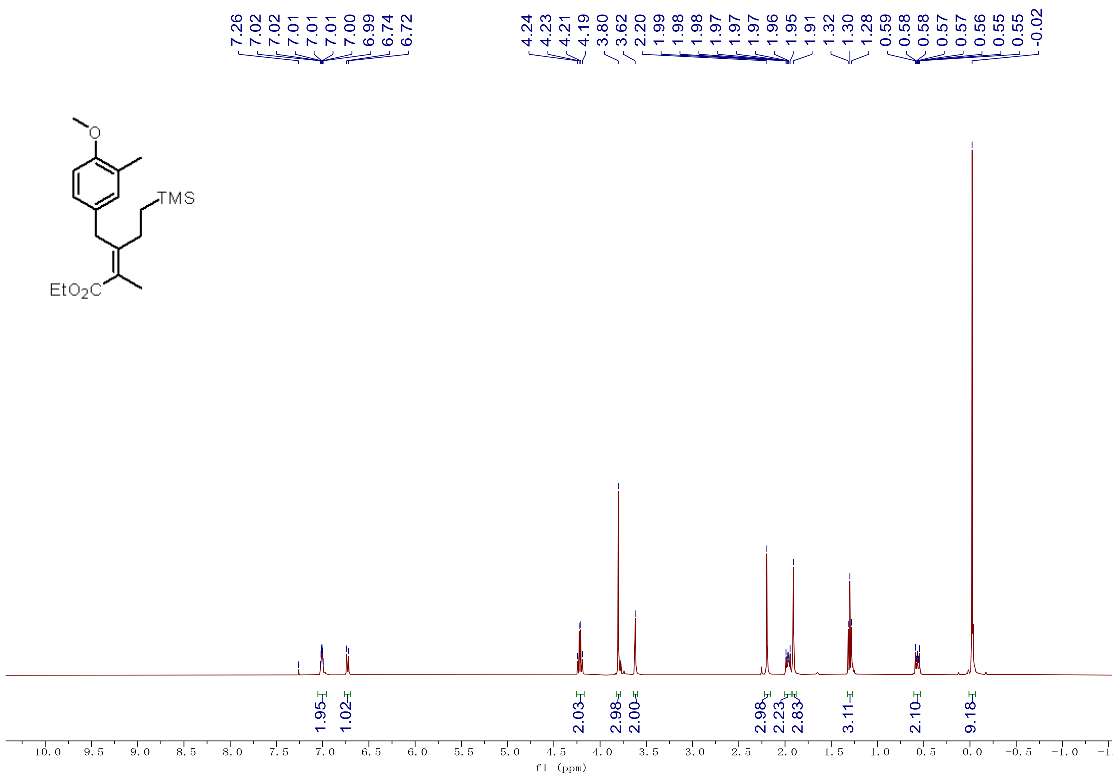

^{1}H NMR spectrum for compound **3aa**

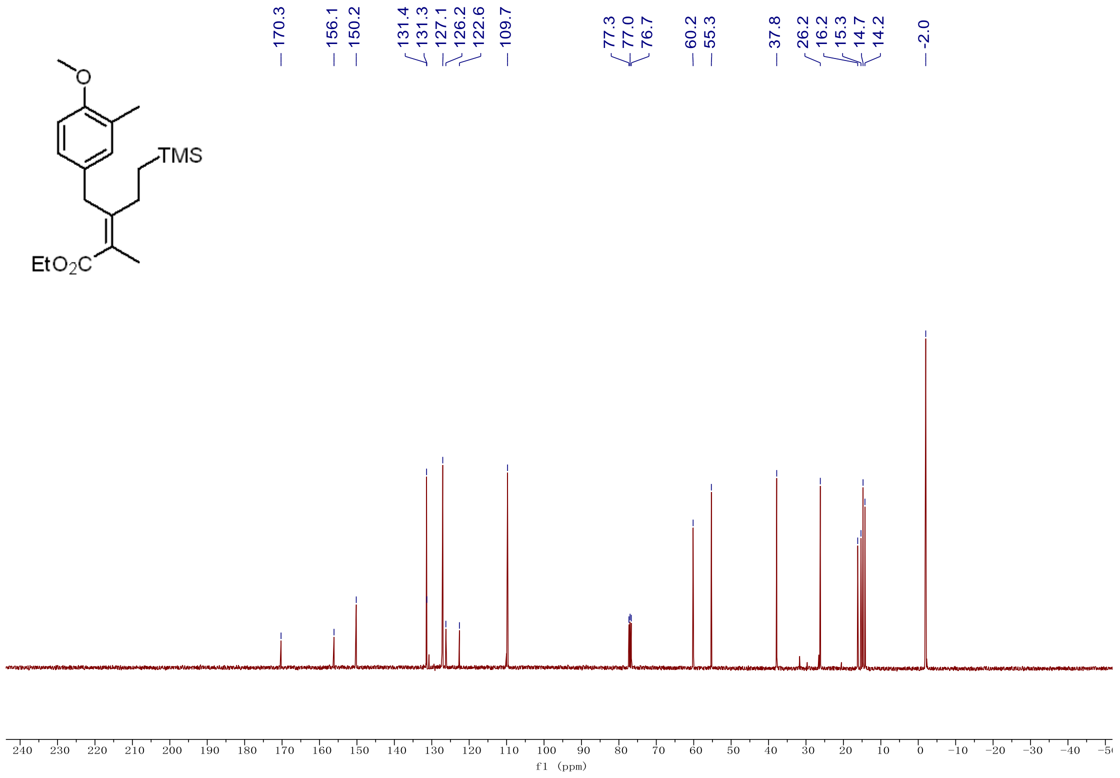

^{13}C NMR spectrum for compound **3aa**

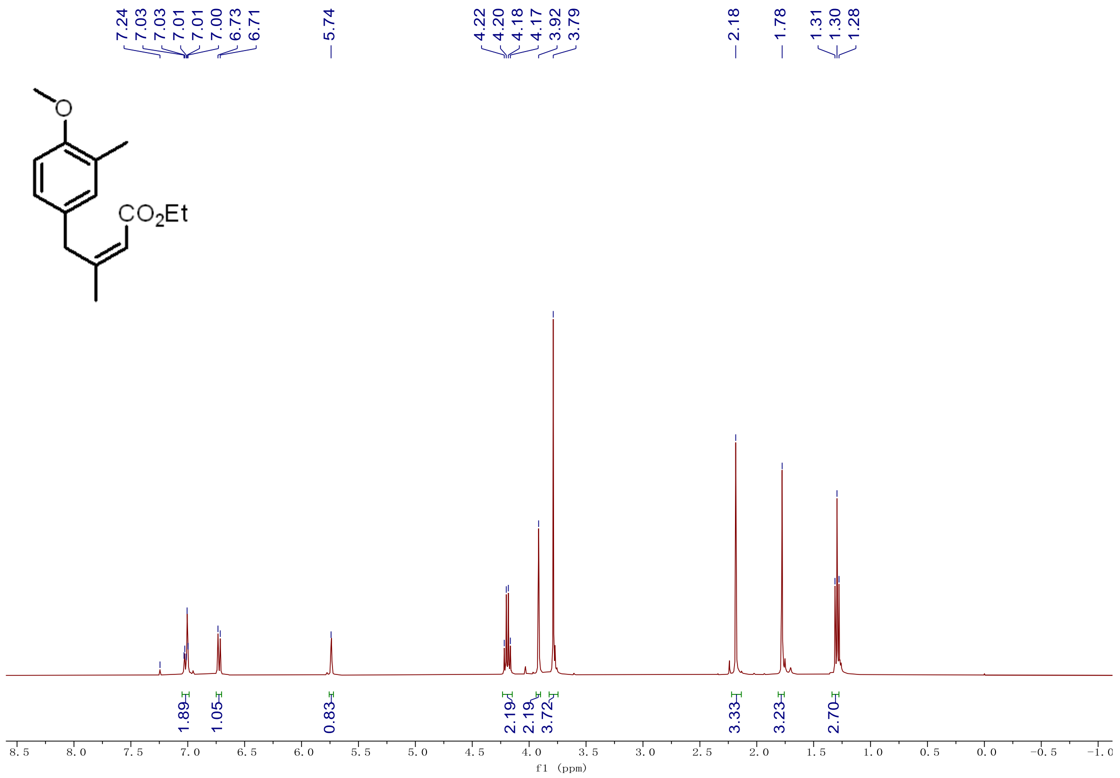

^{1}H NMR spectrum for compound **3bb**

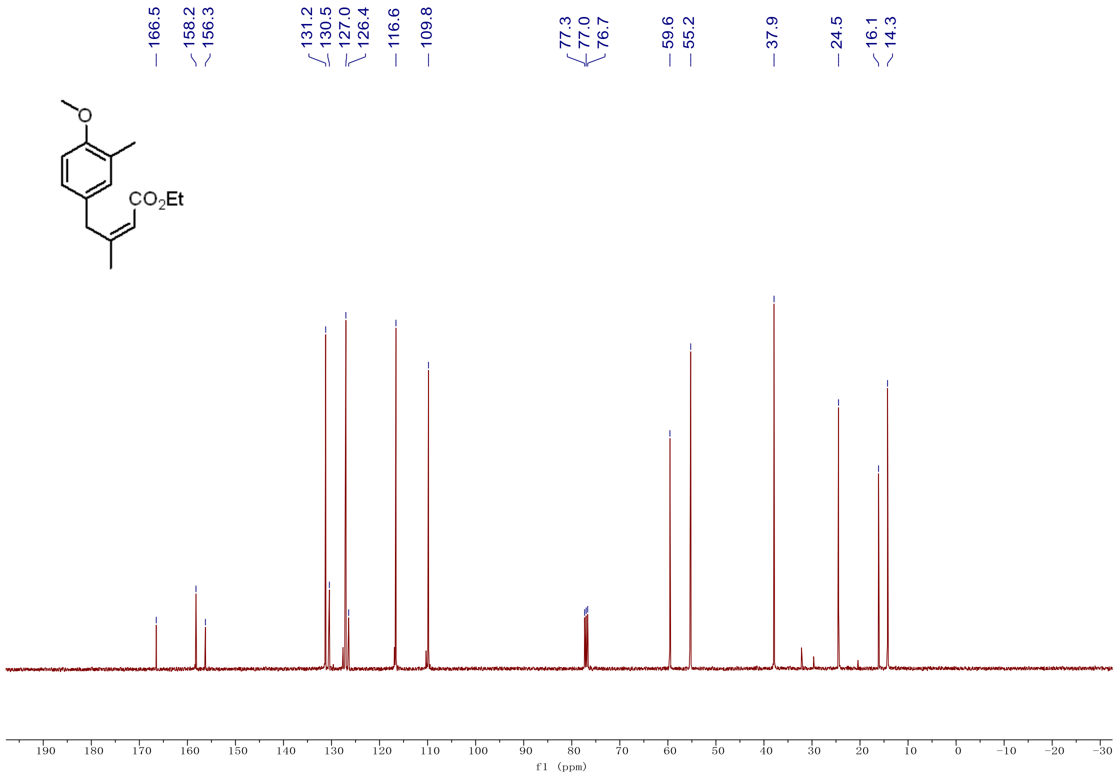

^{13}C NMR spectrum for compound **3bb**

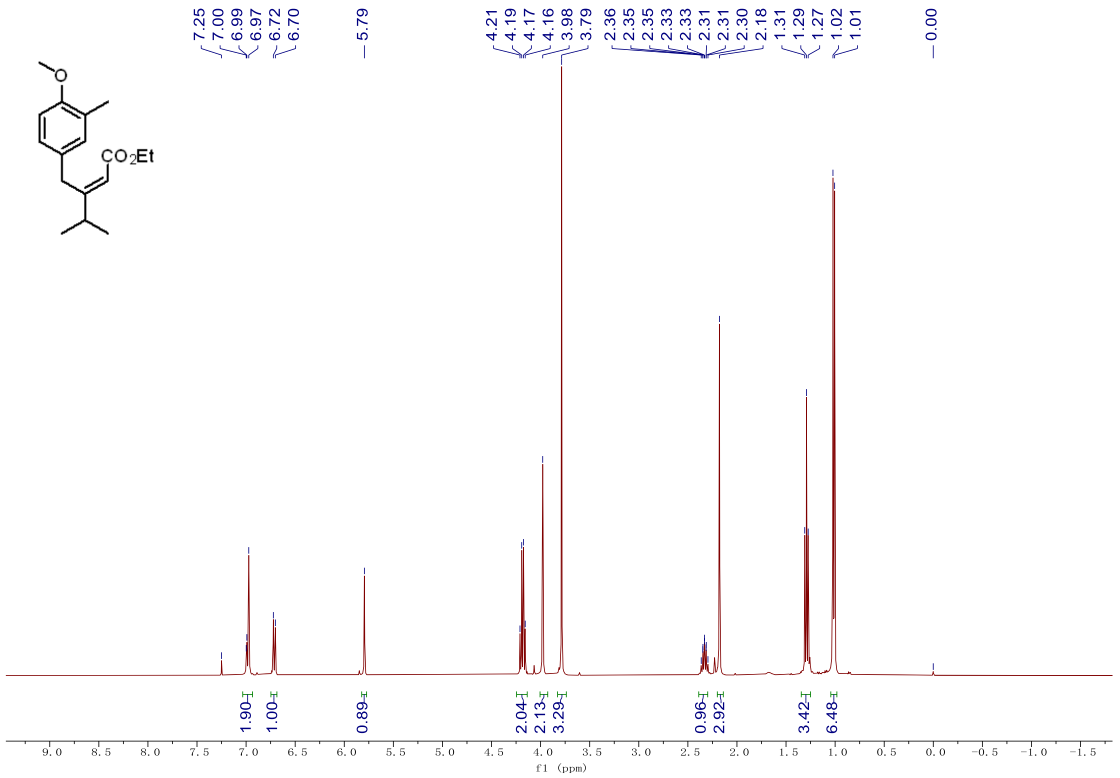

^{1}H NMR spectrum for compound **3cc**

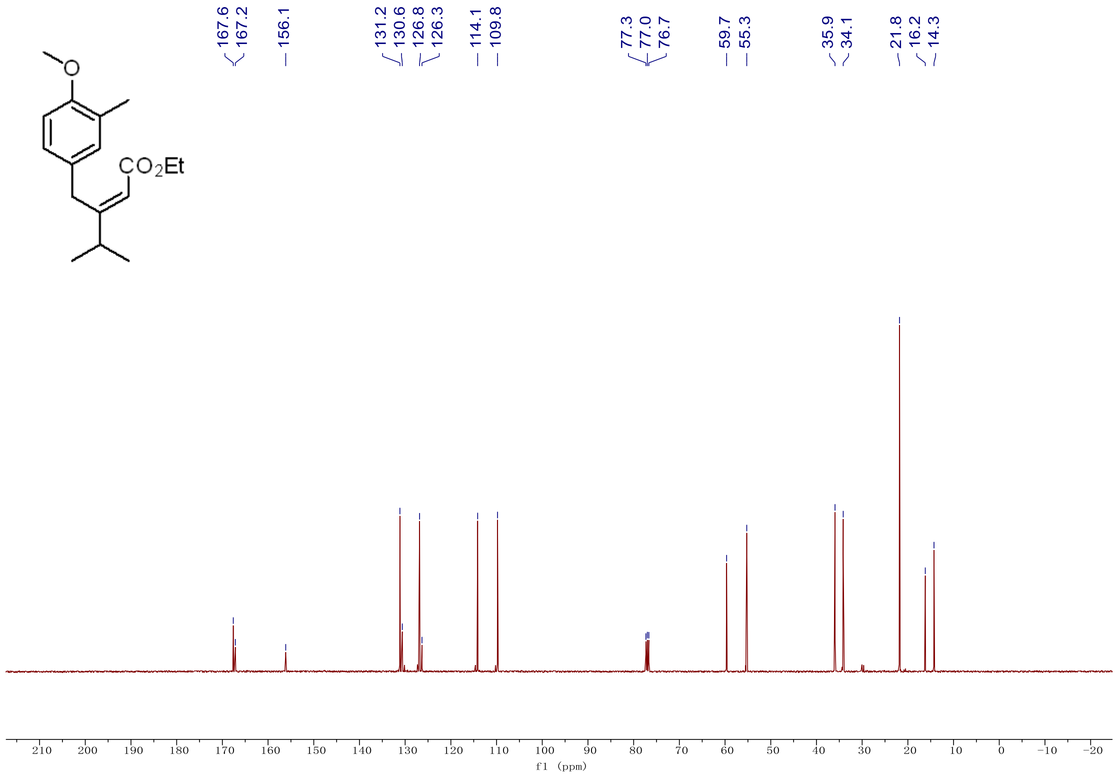

^{13}C NMR spectrum for compound **3cc**

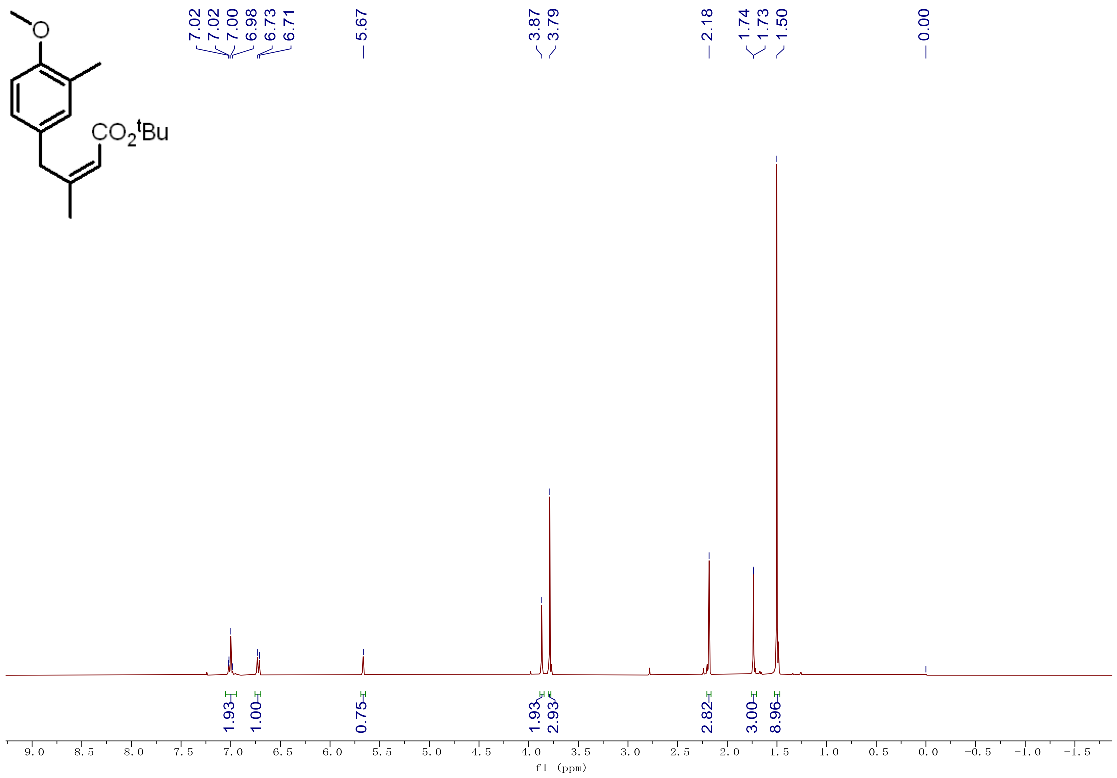

^{1}H NMR spectrum for compound **3dd**

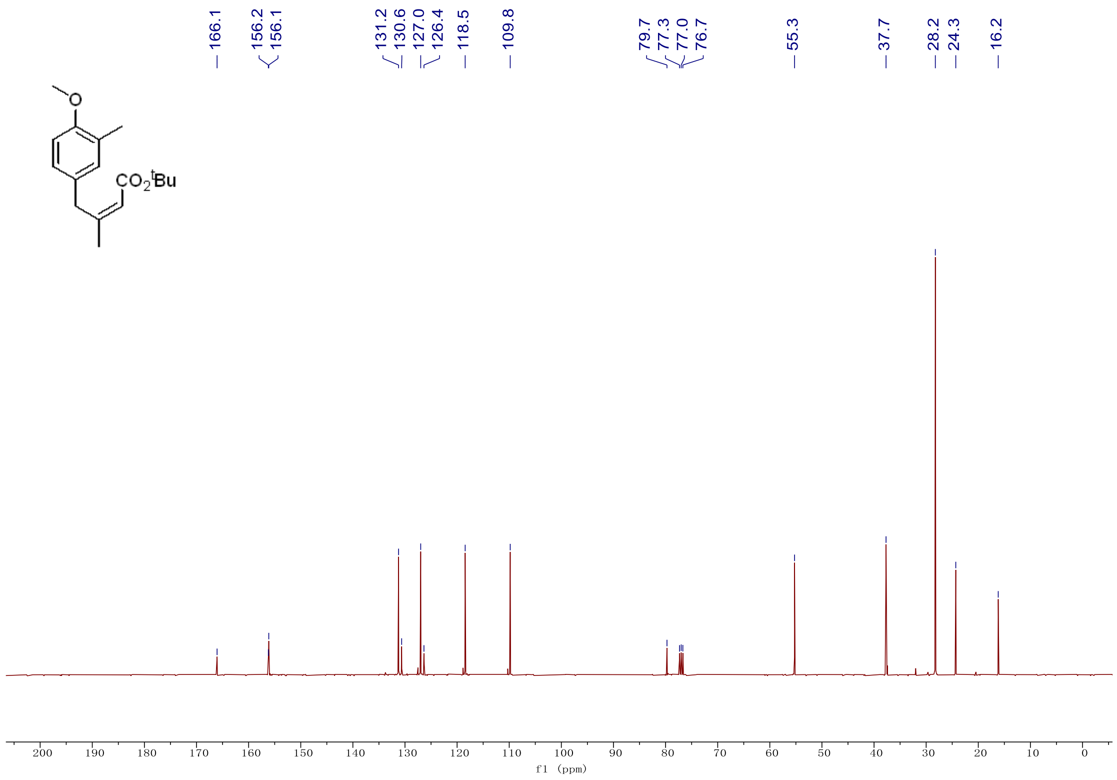

^{13}C NMR spectrum for compound **3dd**

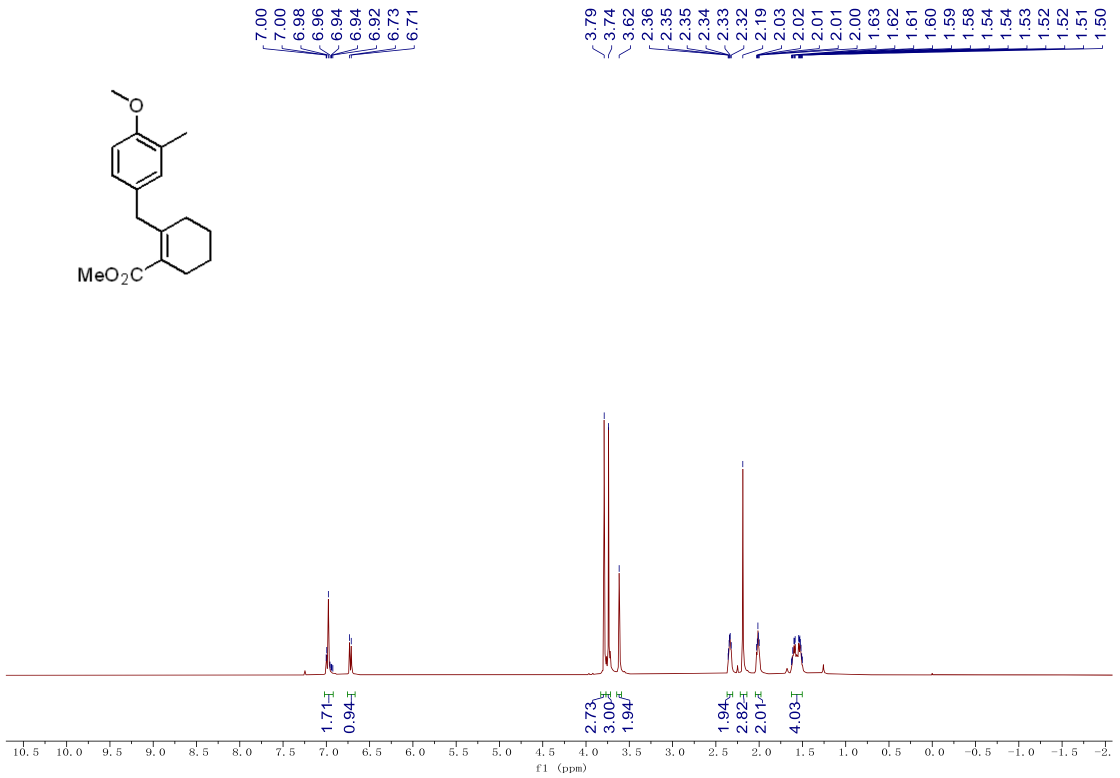

^{1}H NMR spectrum for compound **3ee**

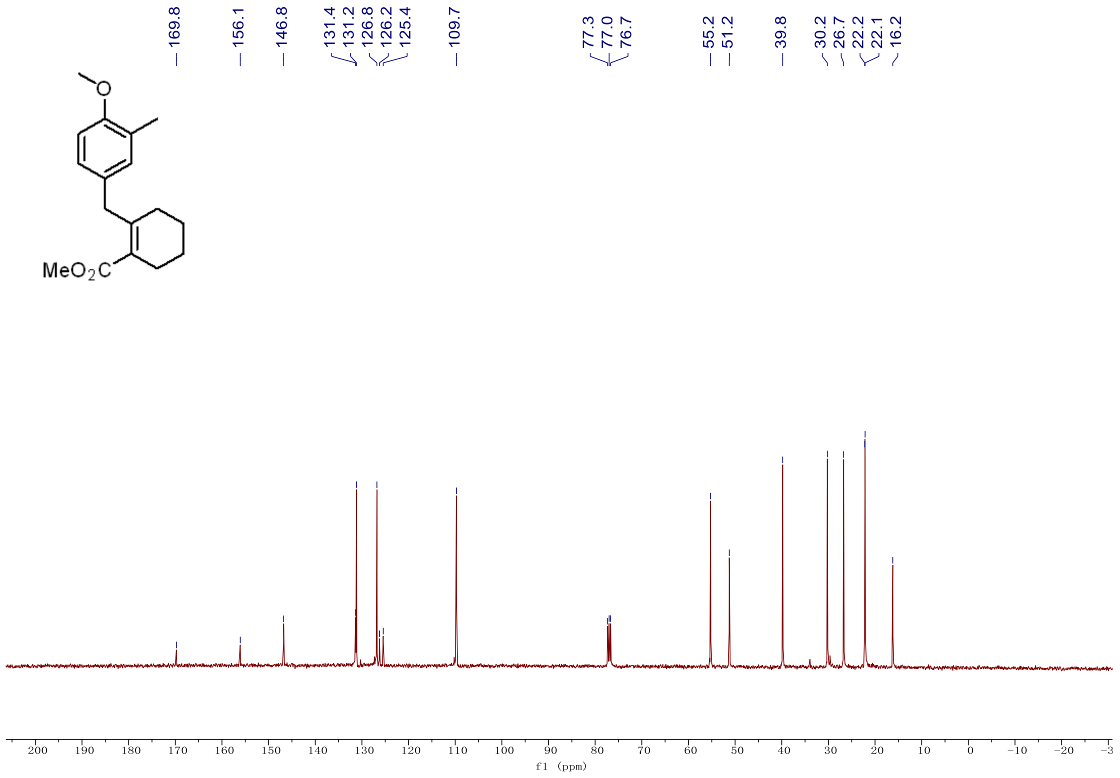

^{13}C NMR spectrum for compound **3ee**

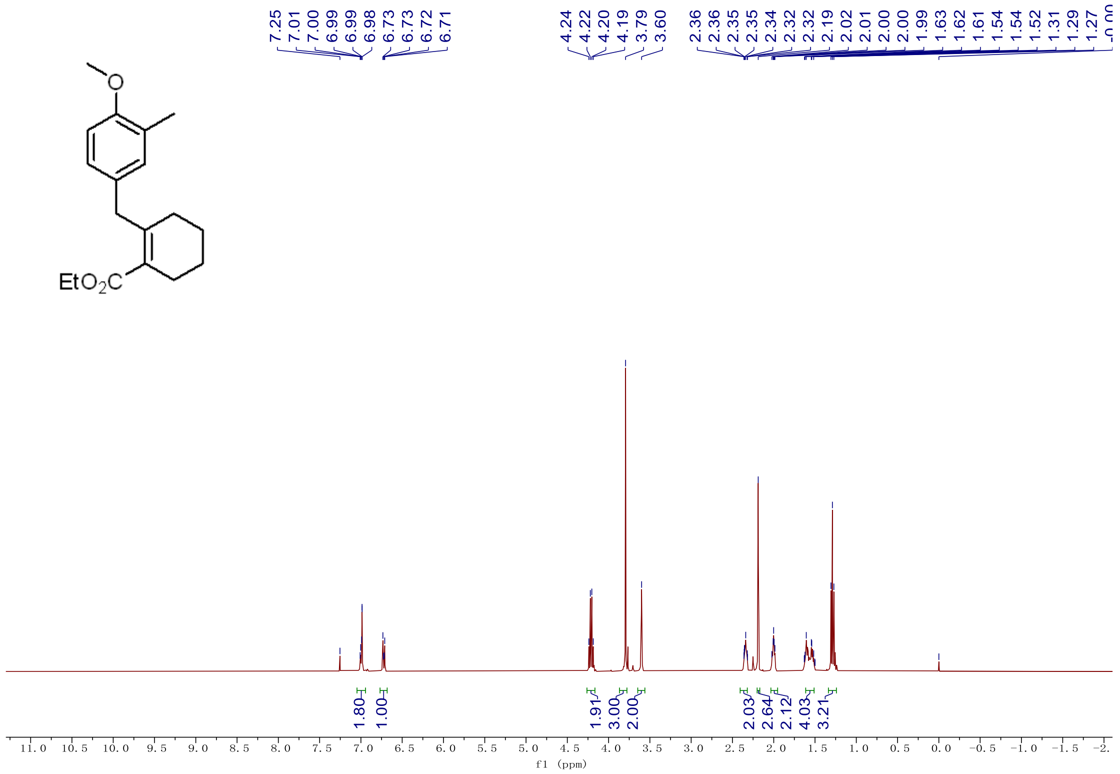

^{1}H NMR spectrum for compound **3ff**

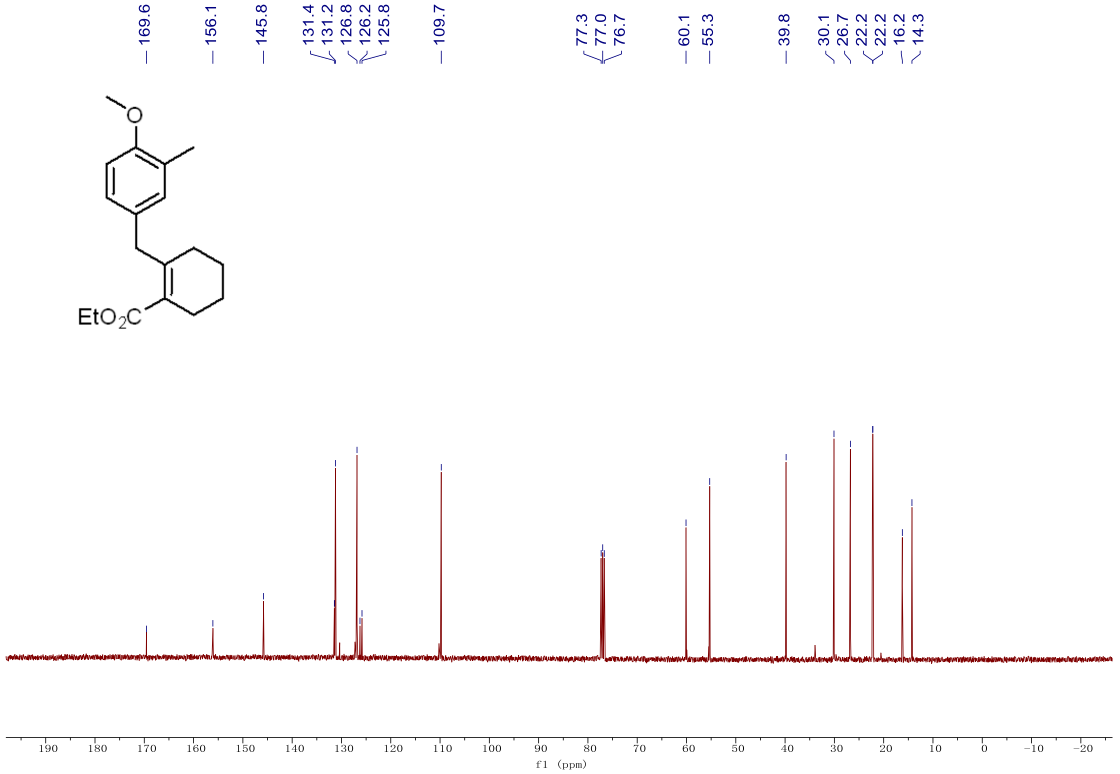

^{13}C NMR spectrum for compound **3ff**

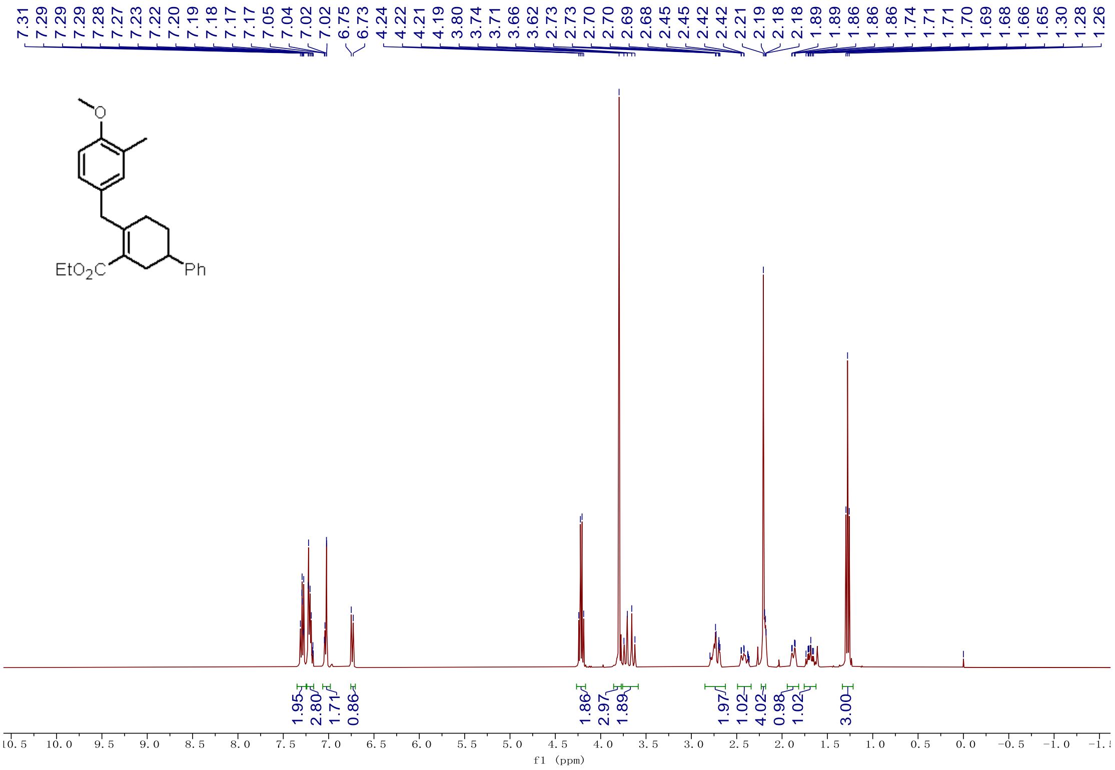

^{1}H NMR spectrum for compound **3gg**

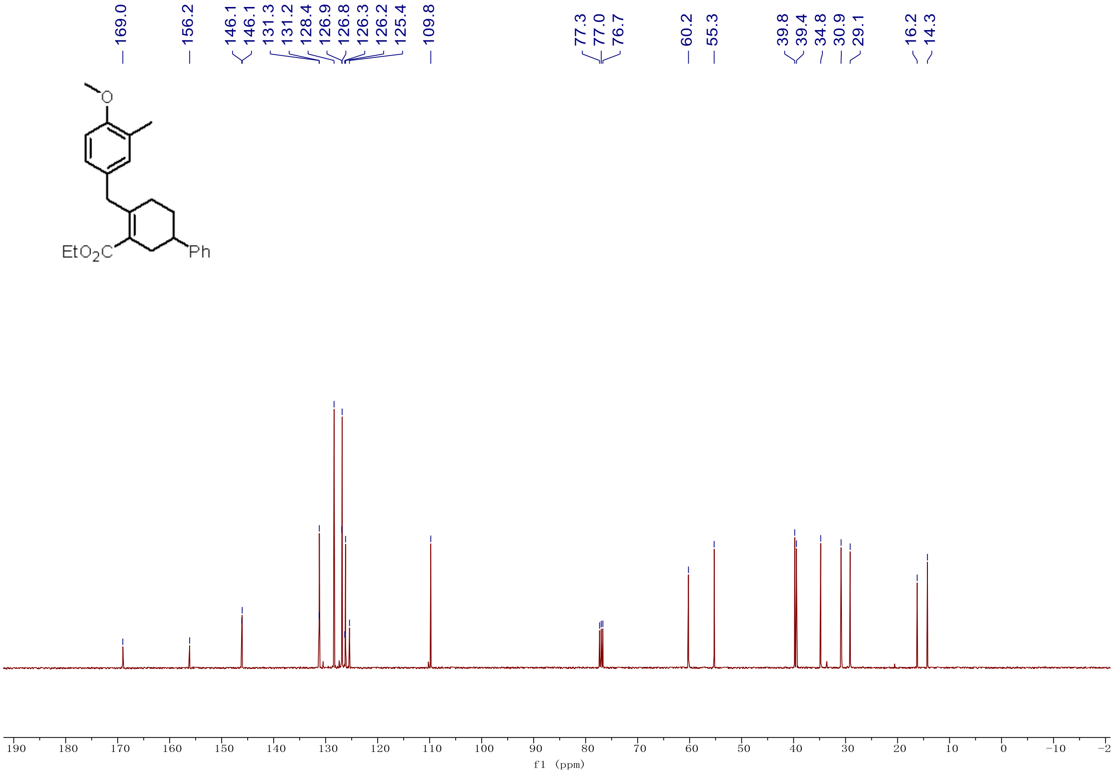

^{13}C NMR spectrum for compound **3gg**

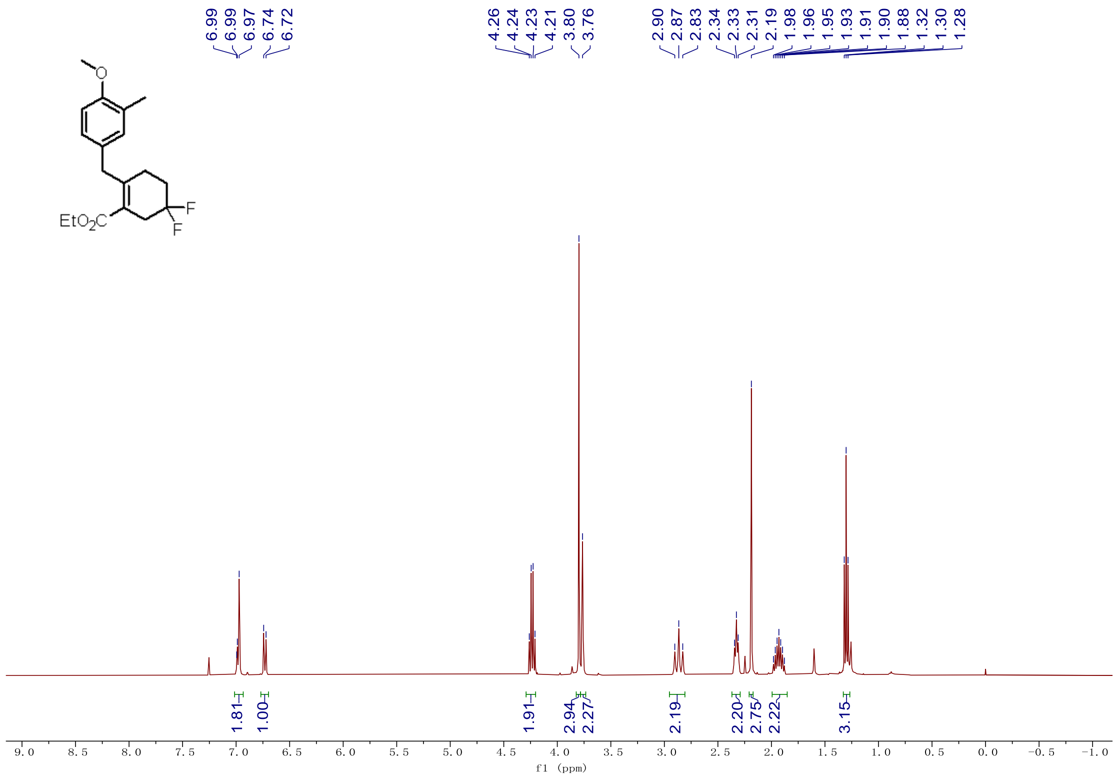

^{1}H NMR spectrum for compound **3hh**

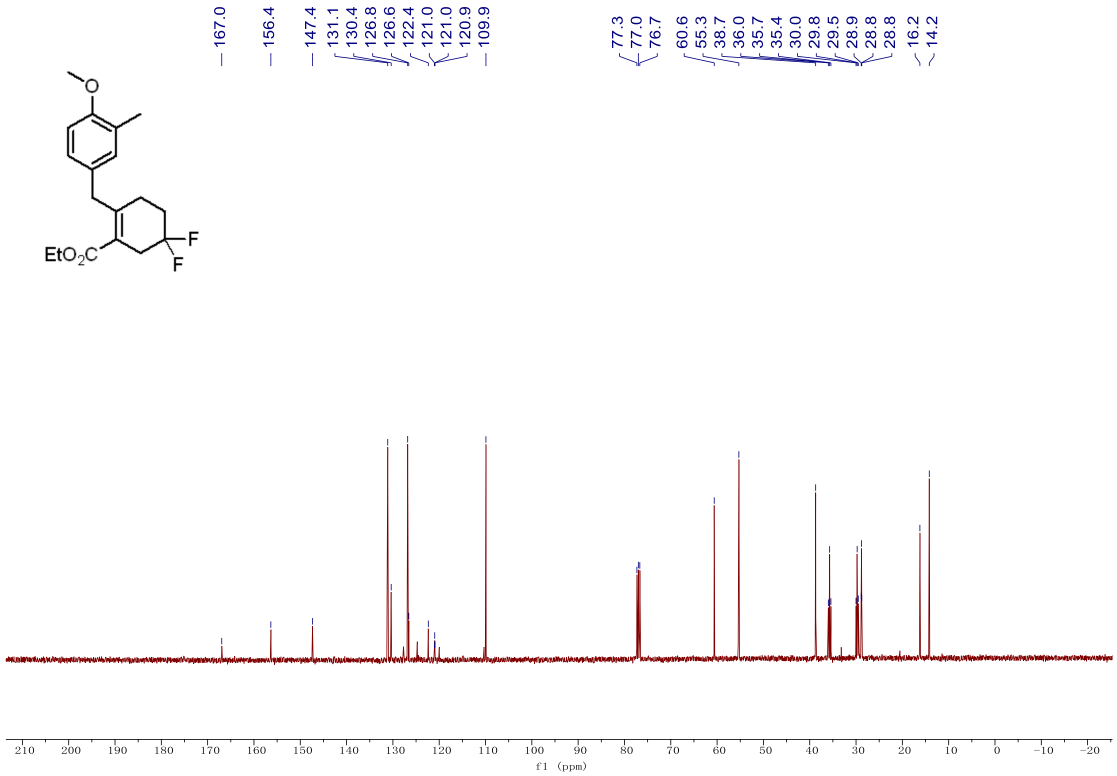

^{13}C NMR spectrum for compound **3hh**

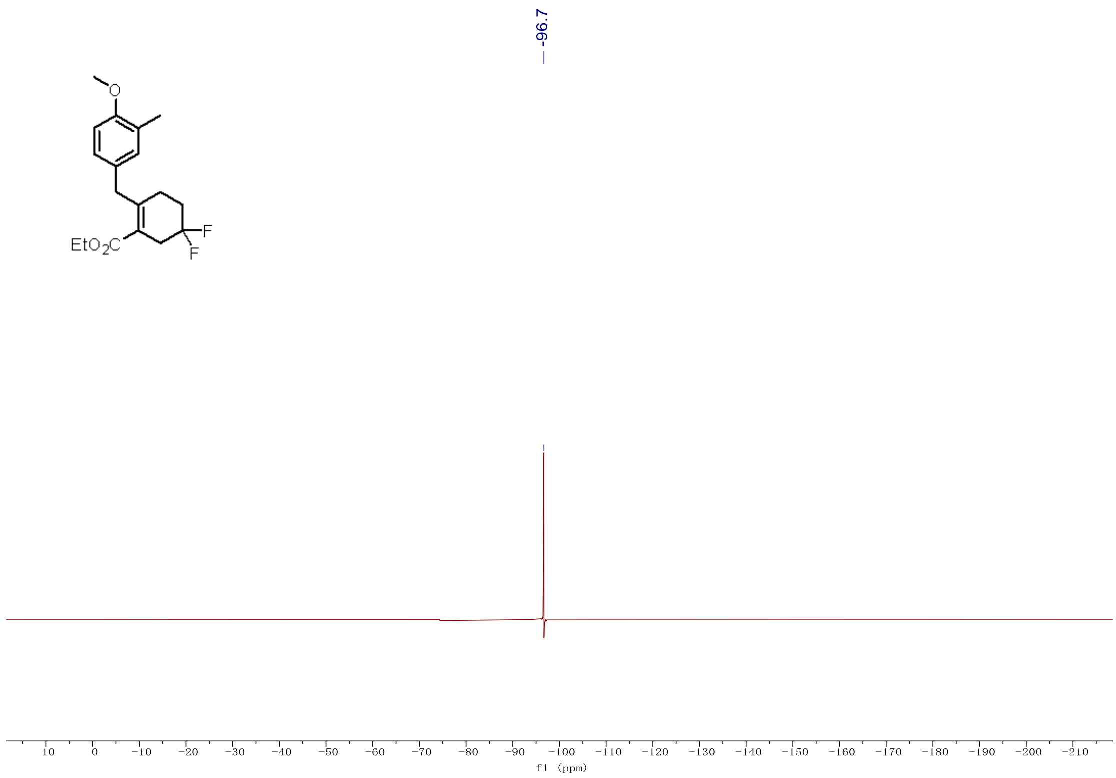

^{19}F NMR spectrum for compound **3hh**

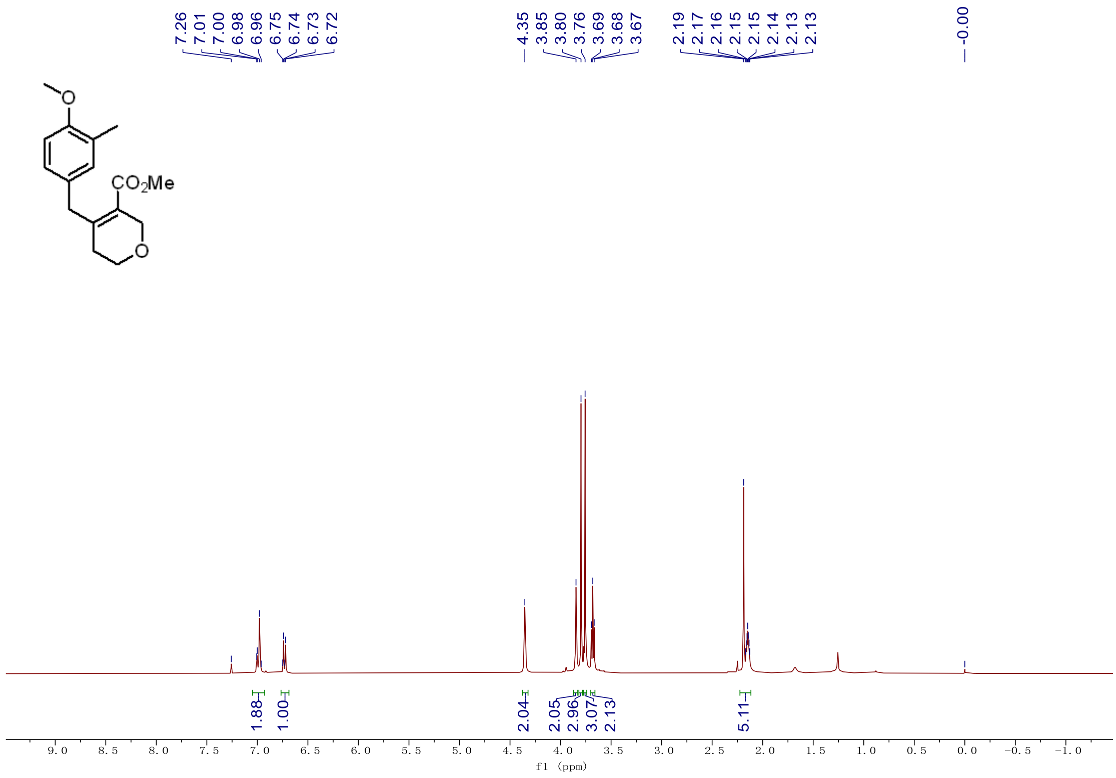

^{1}H NMR spectrum for compound **3ii**

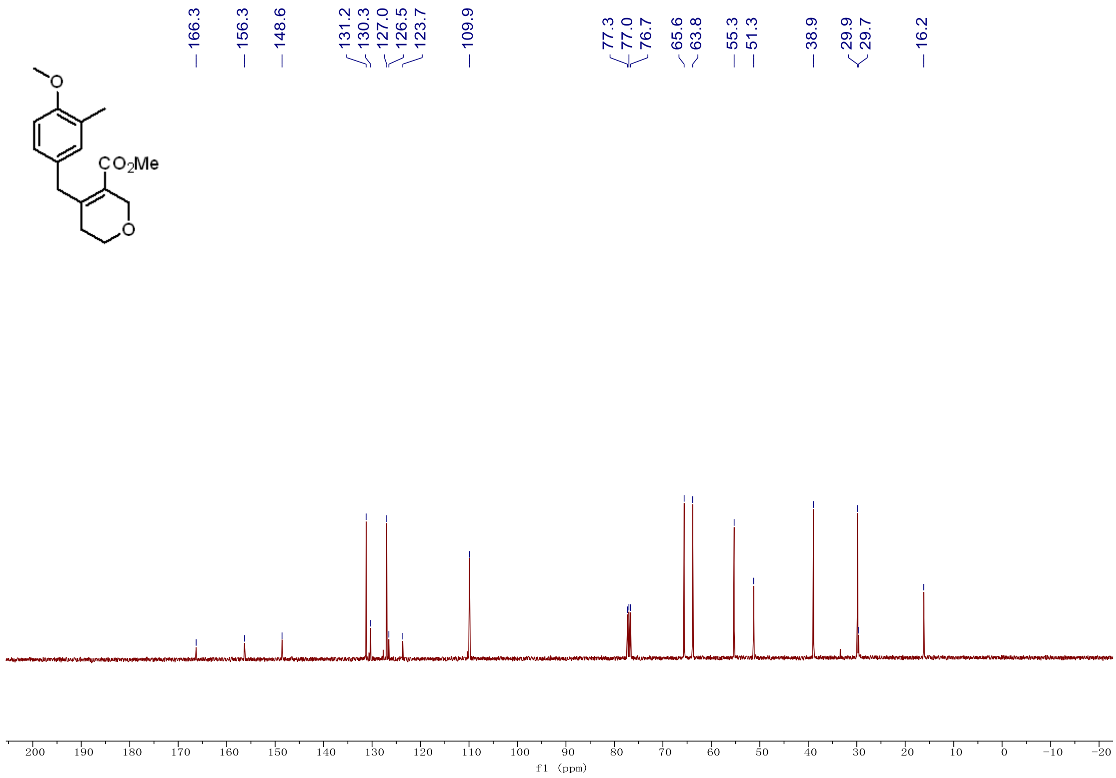

^{13}C NMR spectrum for compound **3ii**

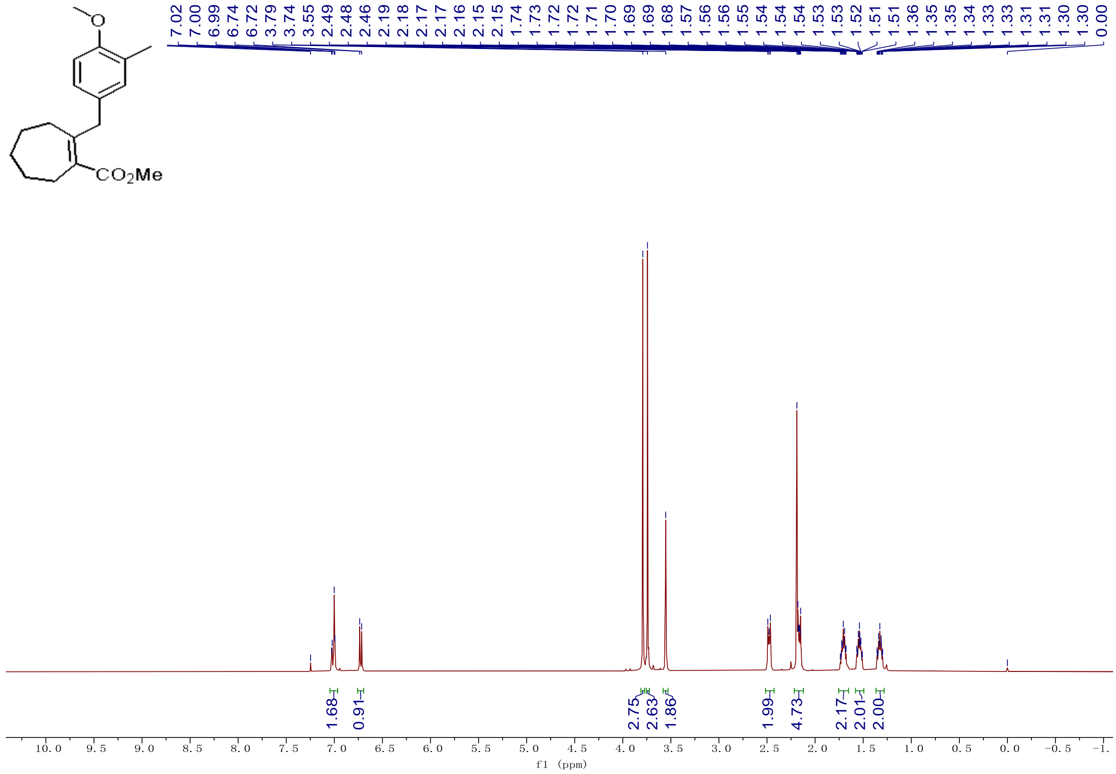

^{1}H NMR spectrum for compound **3jj**

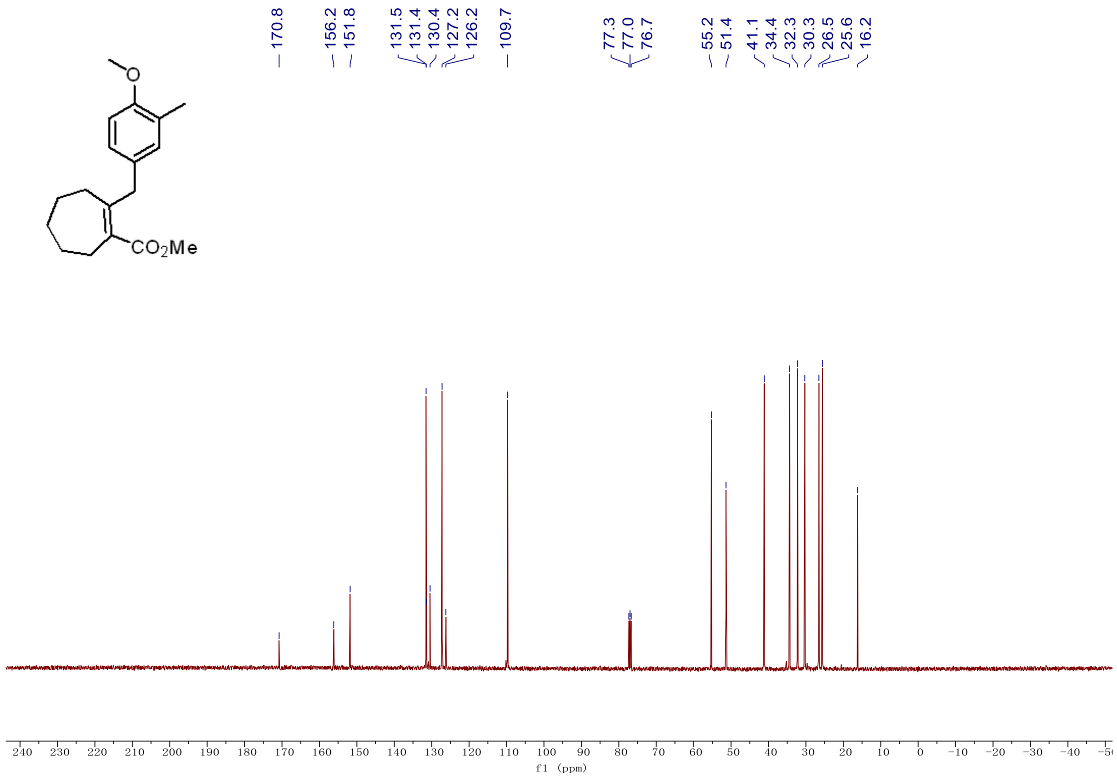

^{13}C NMR spectrum for compound **3jj**

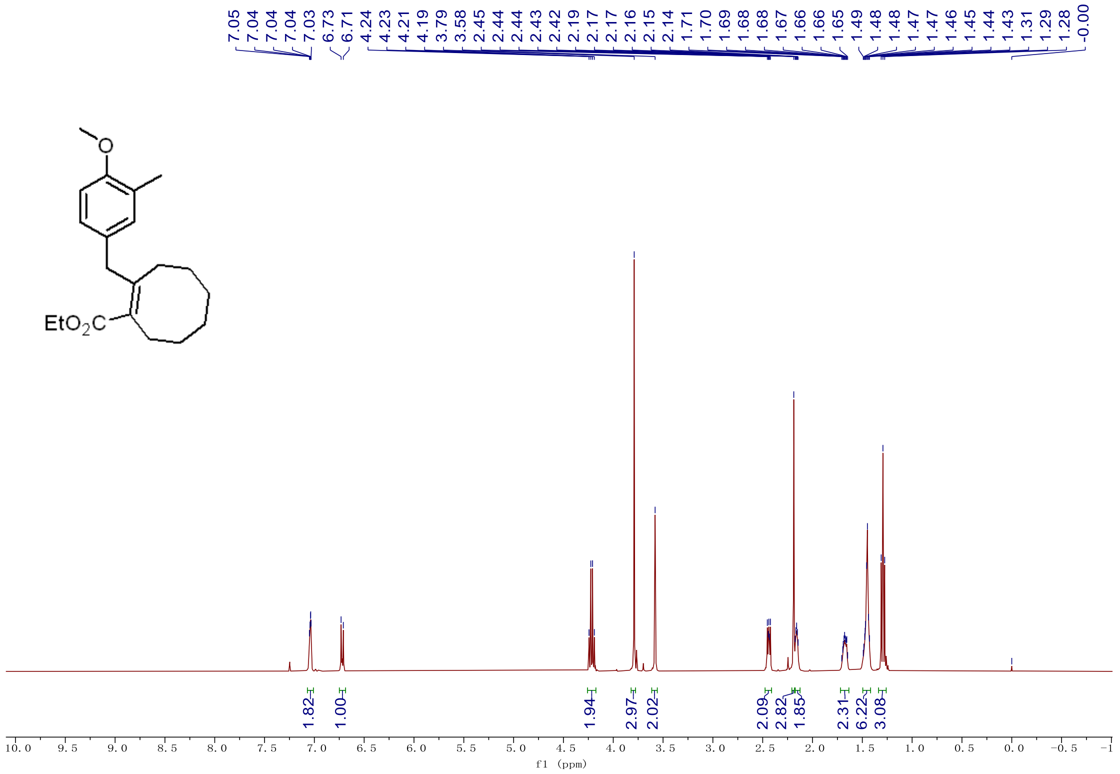

^{1}H NMR spectrum for compound **3kk**

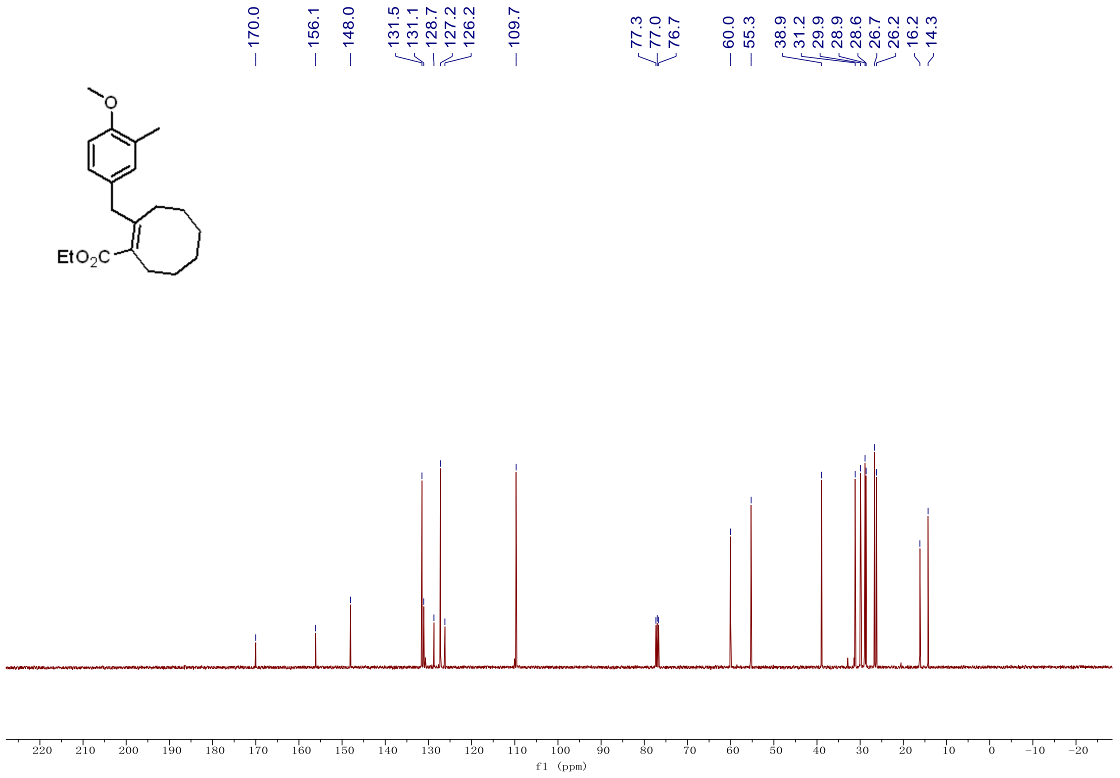

^{13}C NMR spectrum for compound **3kk**

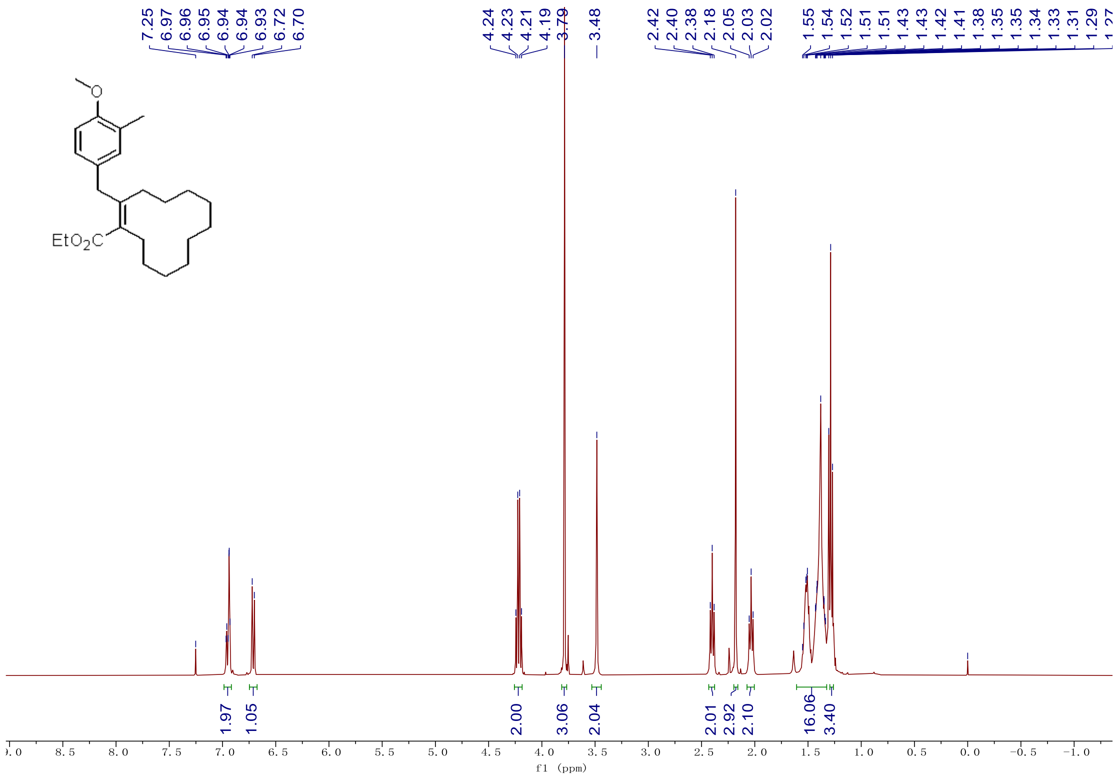

^{1}H NMR spectrum for compound **3ll**

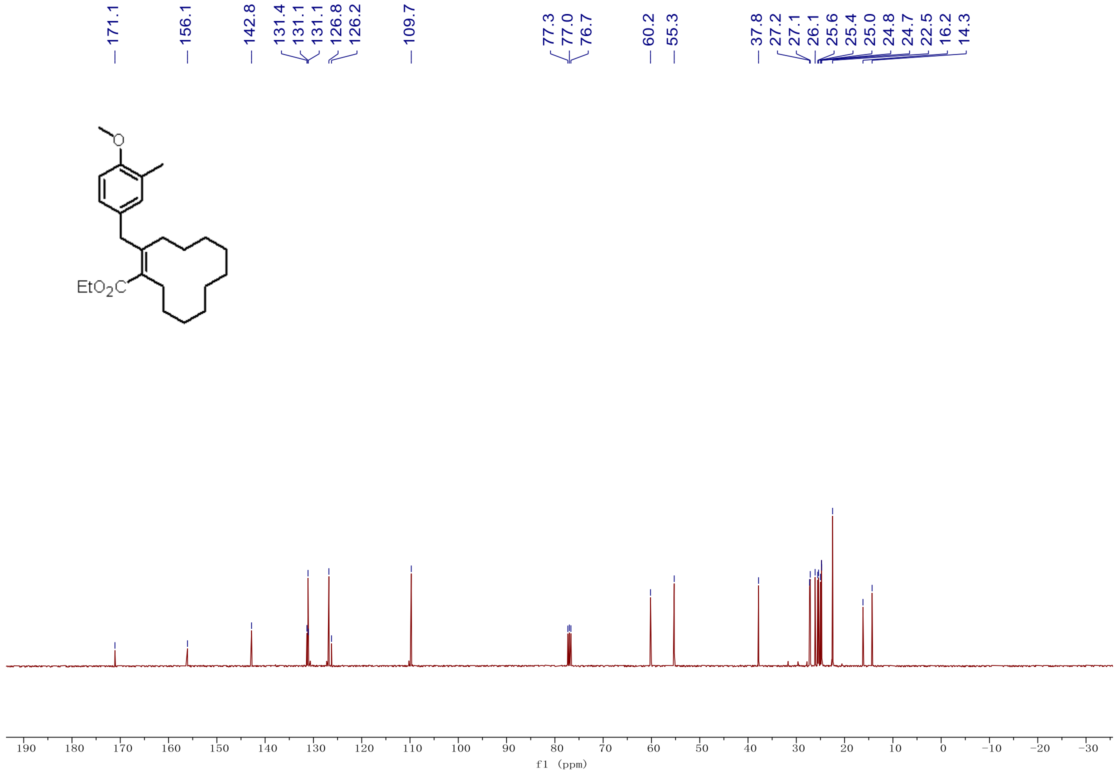

^{13}C NMR spectrum for compound **3ll**

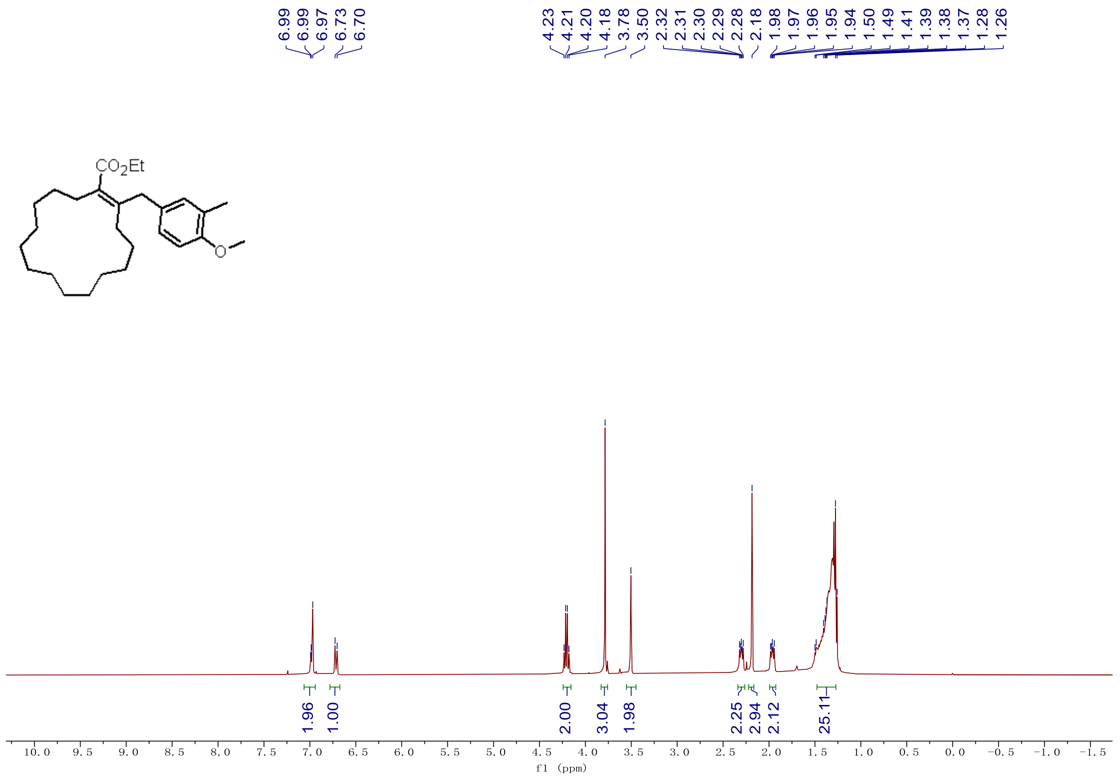

^{1}H NMR spectrum for compound **3mm**

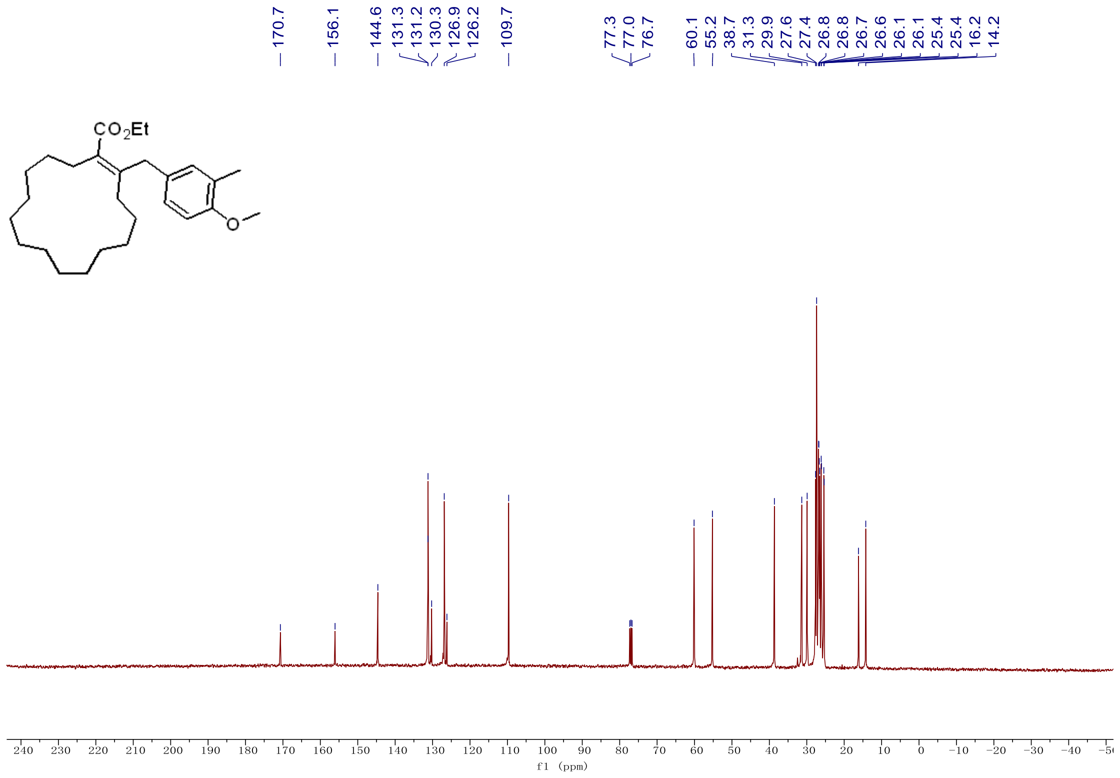

^{13}C NMR spectrum for compound **3mm**

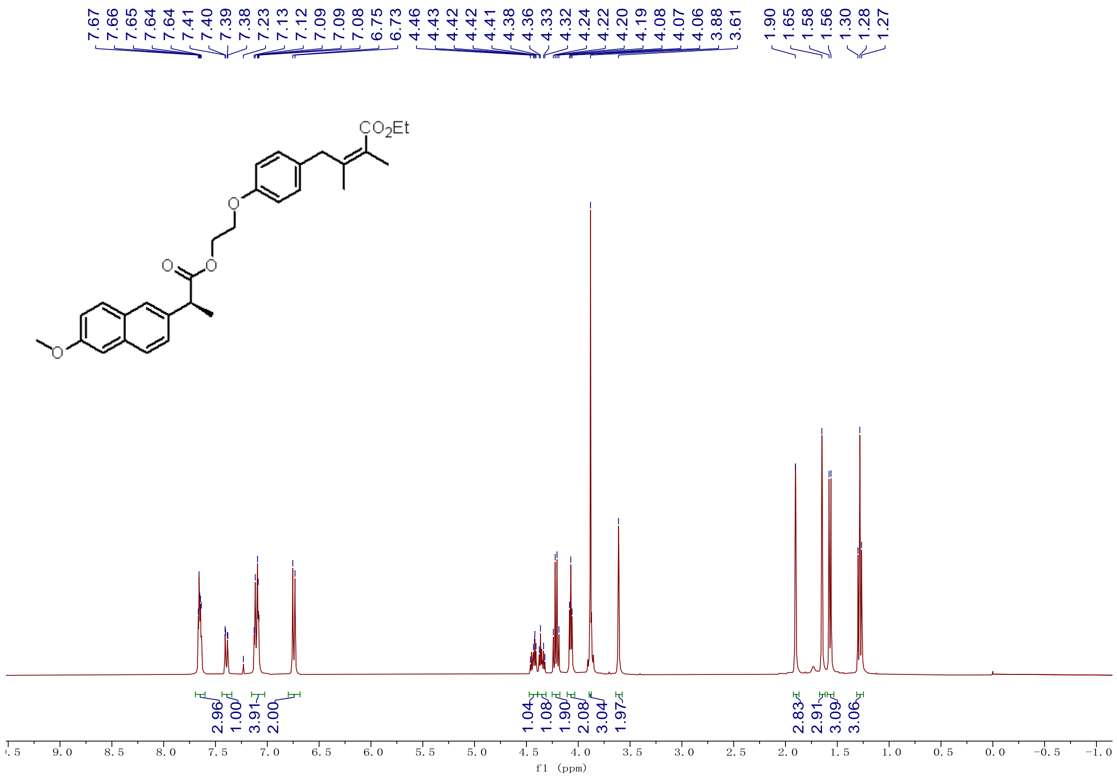

^{1}H NMR spectrum for compound **4a**

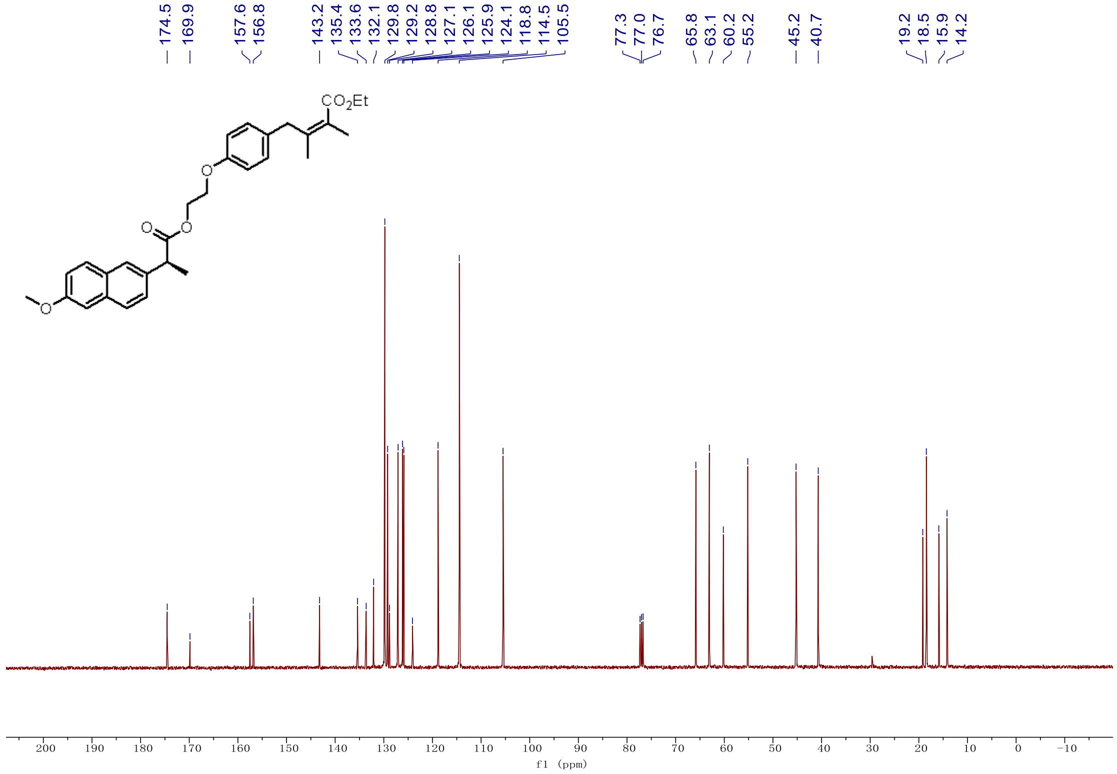

^{13}C NMR spectrum for compound **4a**

.

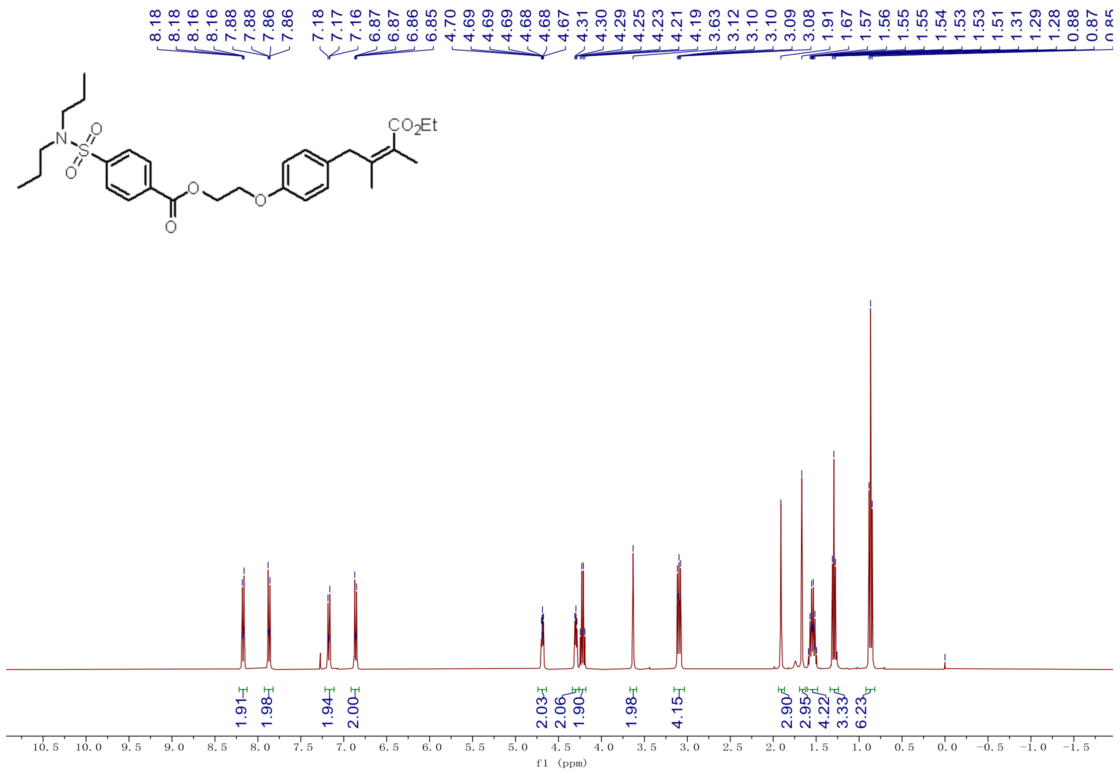

^{1}H NMR spectrum for compound **4b**

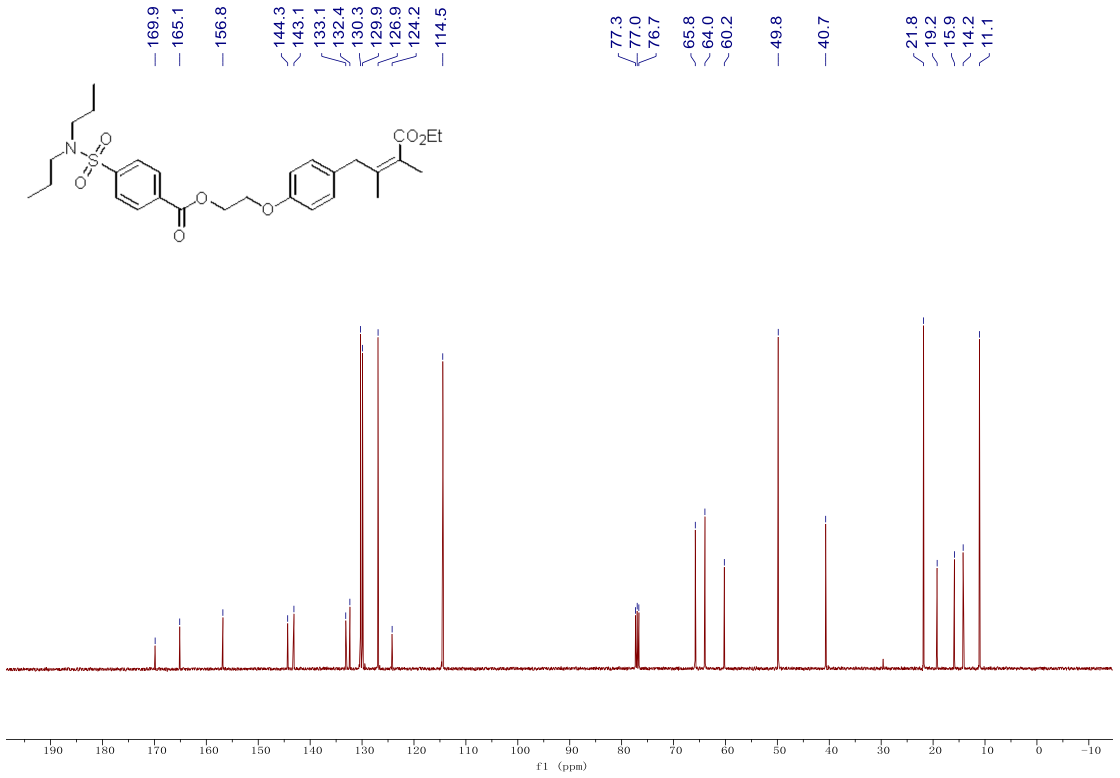

^{13}C NMR spectrum for compound **4b**

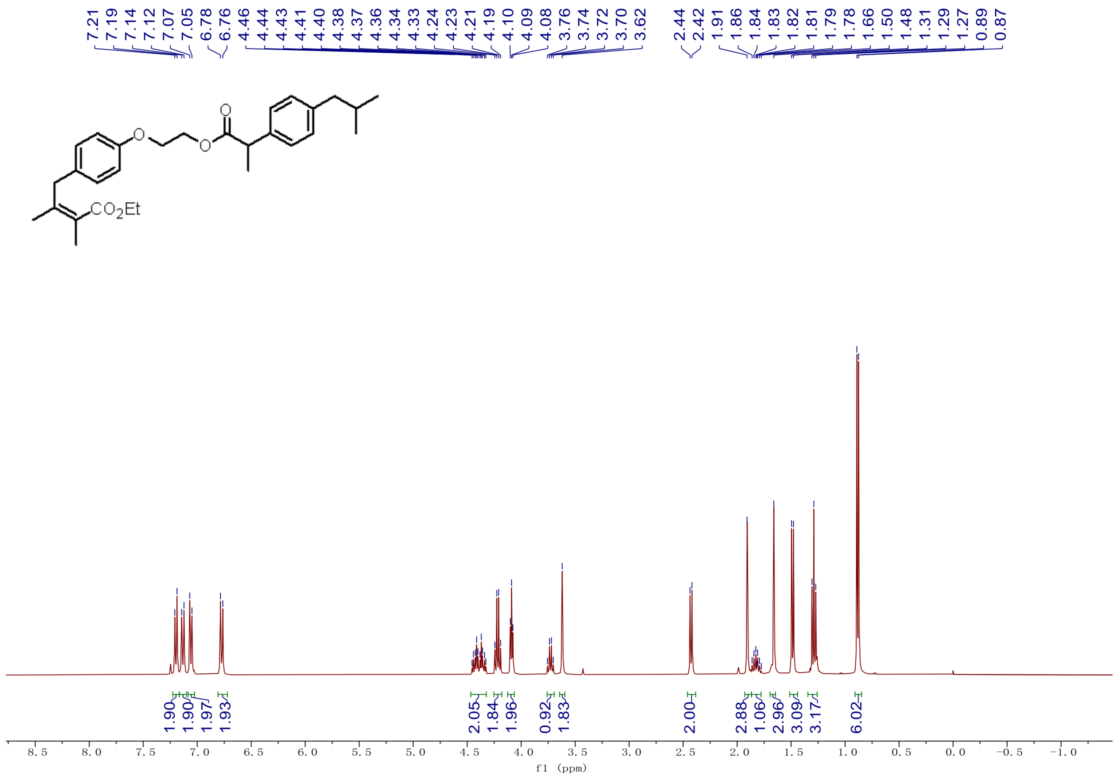

^{1}H NMR spectrum for compound **4c**

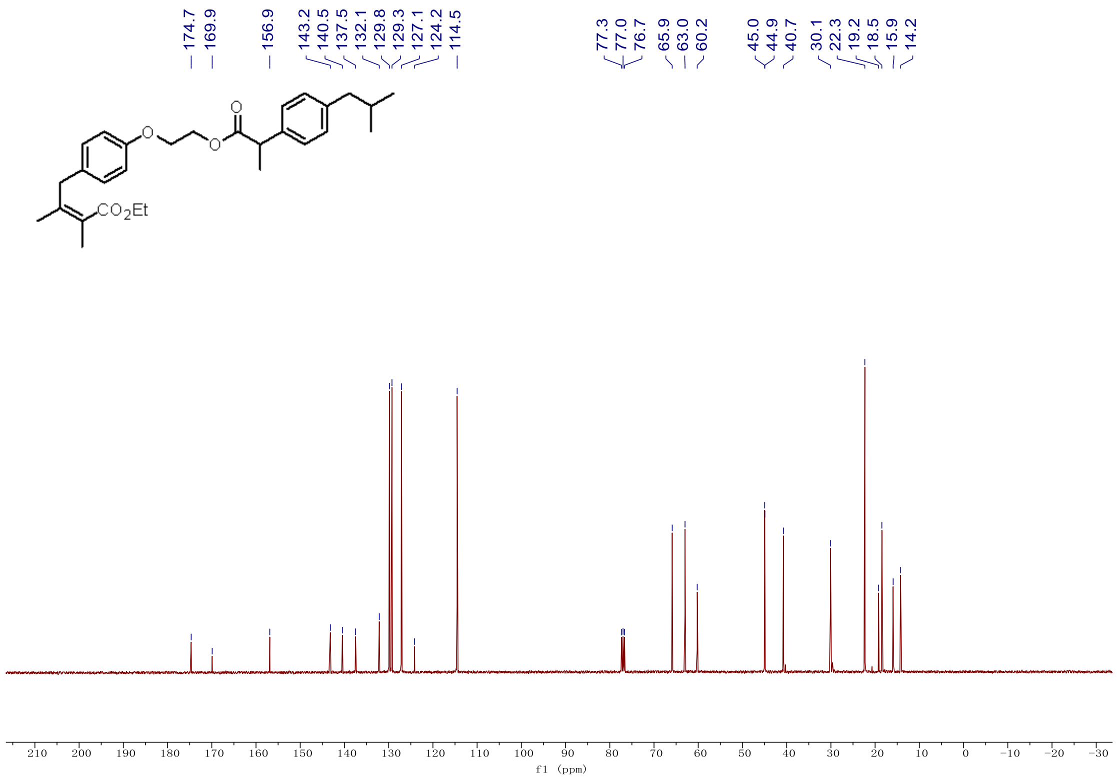

^{13}C NMR spectrum for compound **4c**

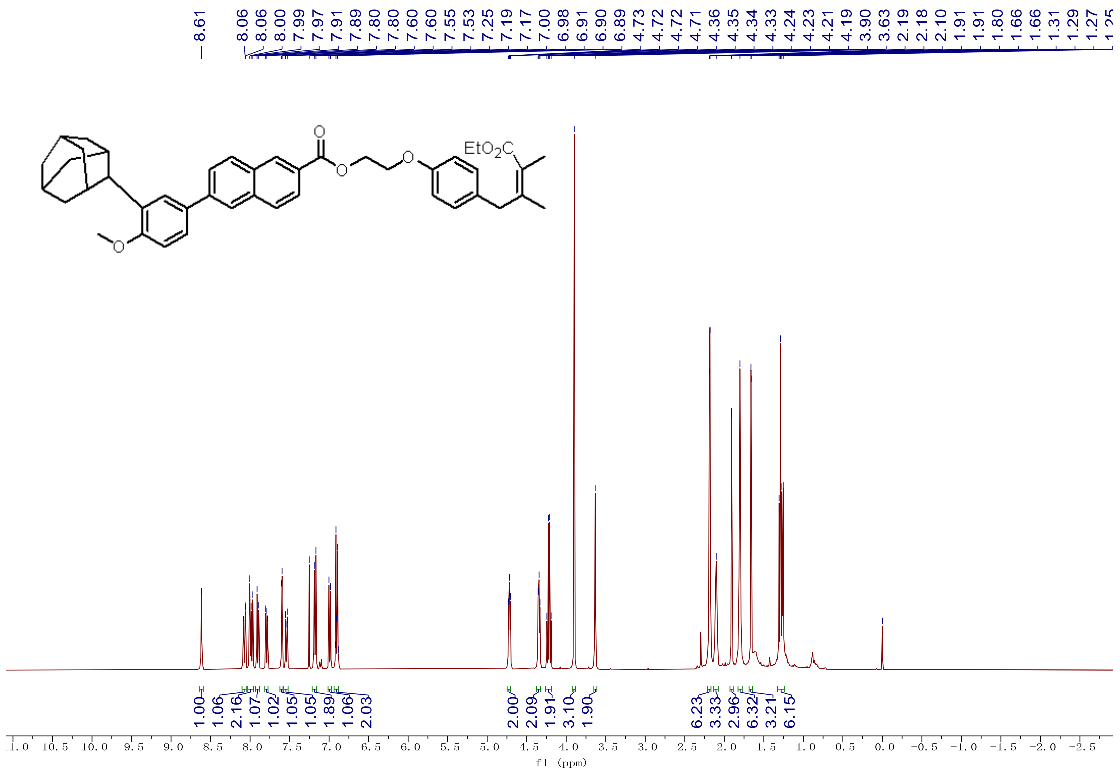

^{1}H NMR spectrum for compound **4d**

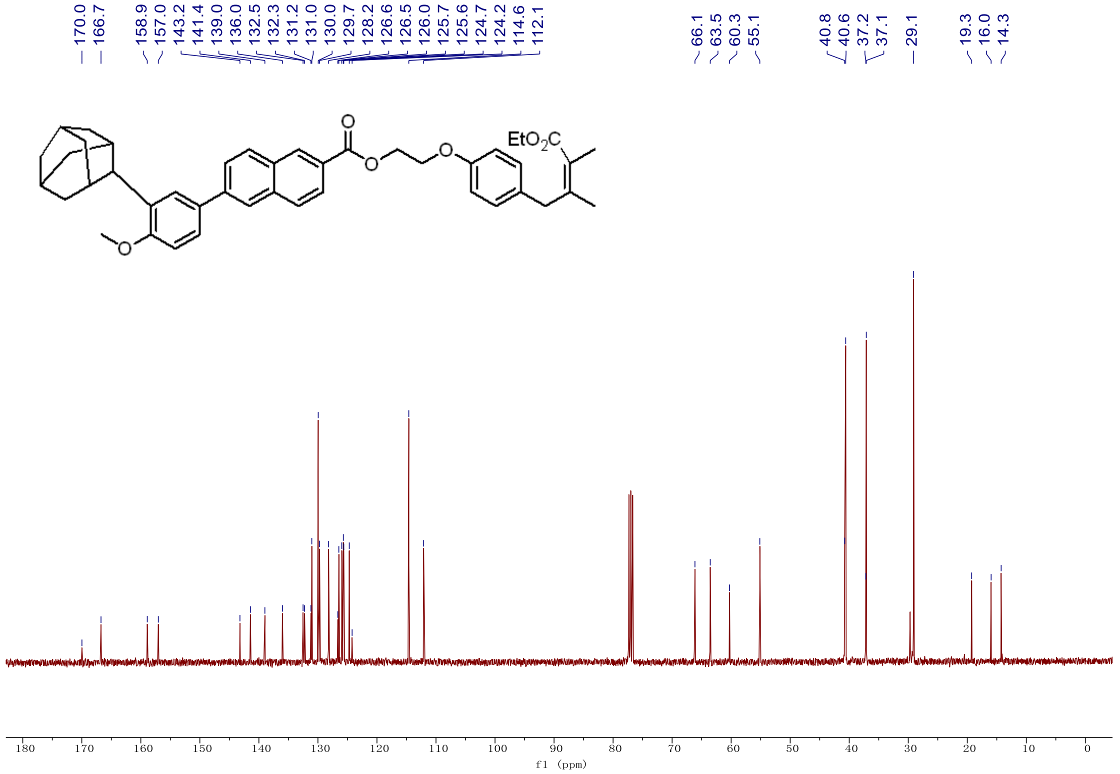

^{13}C NMR spectrum for compound **4d**

.

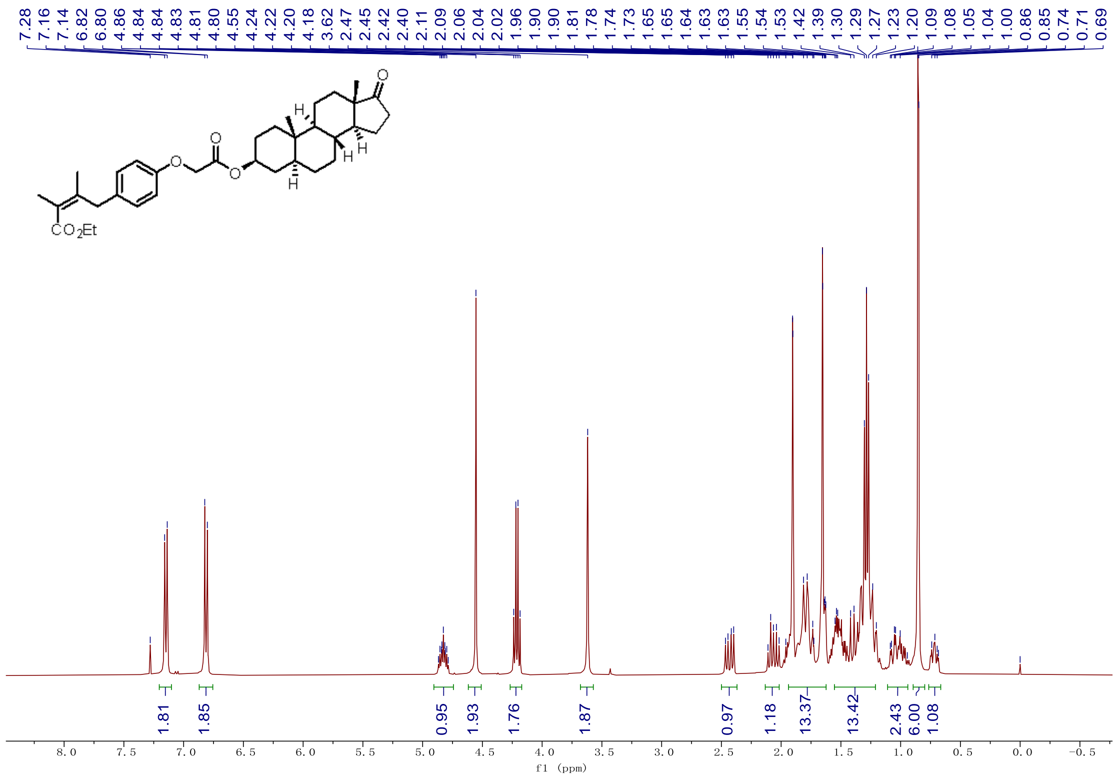

^{1}H NMR spectrum for compound **4e**

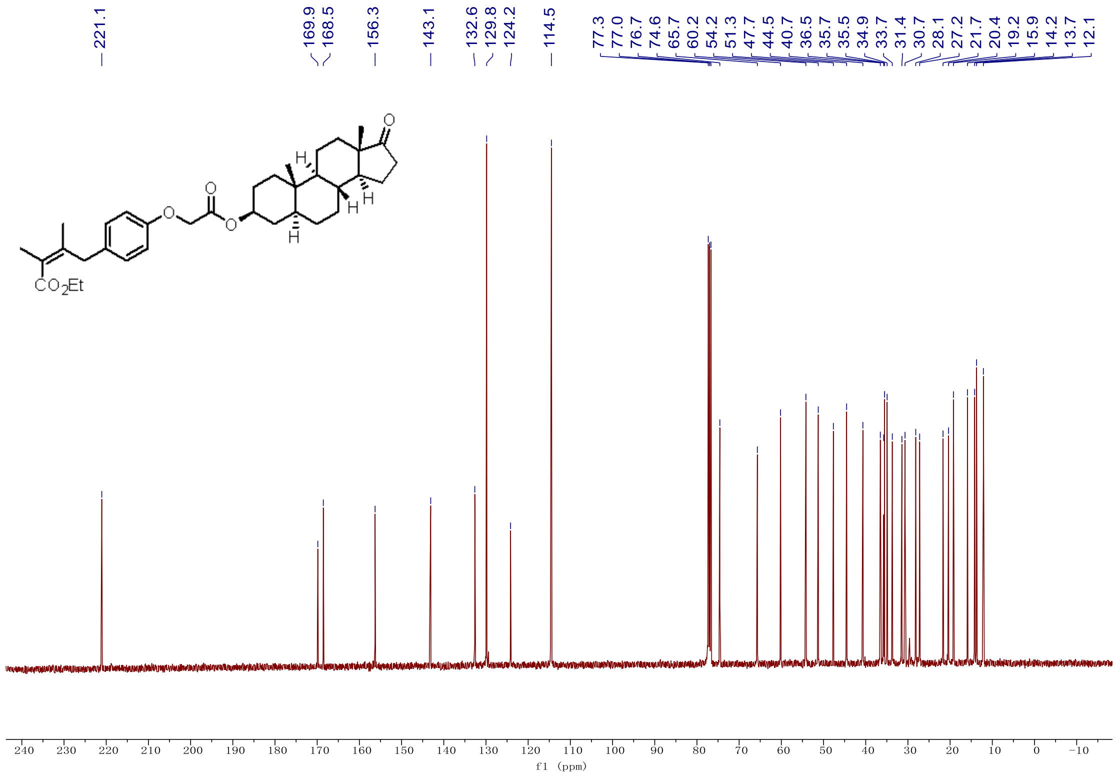

^{13}C NMR spectrum for compound **4e**

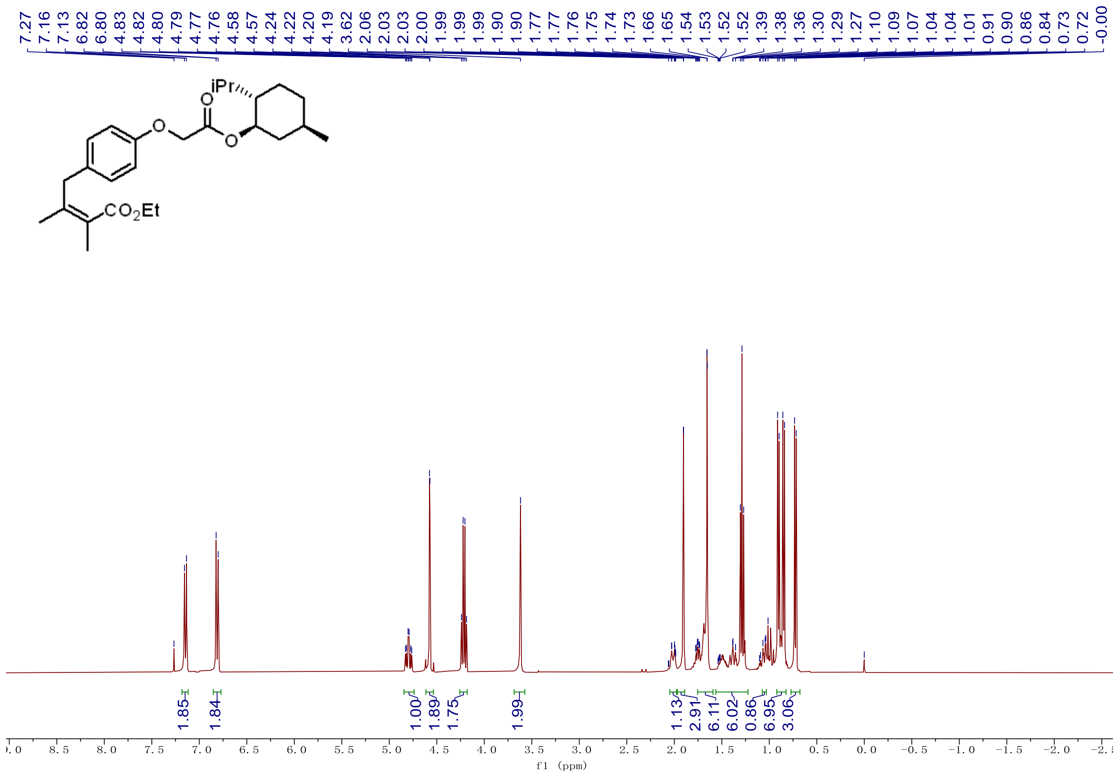

^{1}H NMR spectrum for compound **4f**

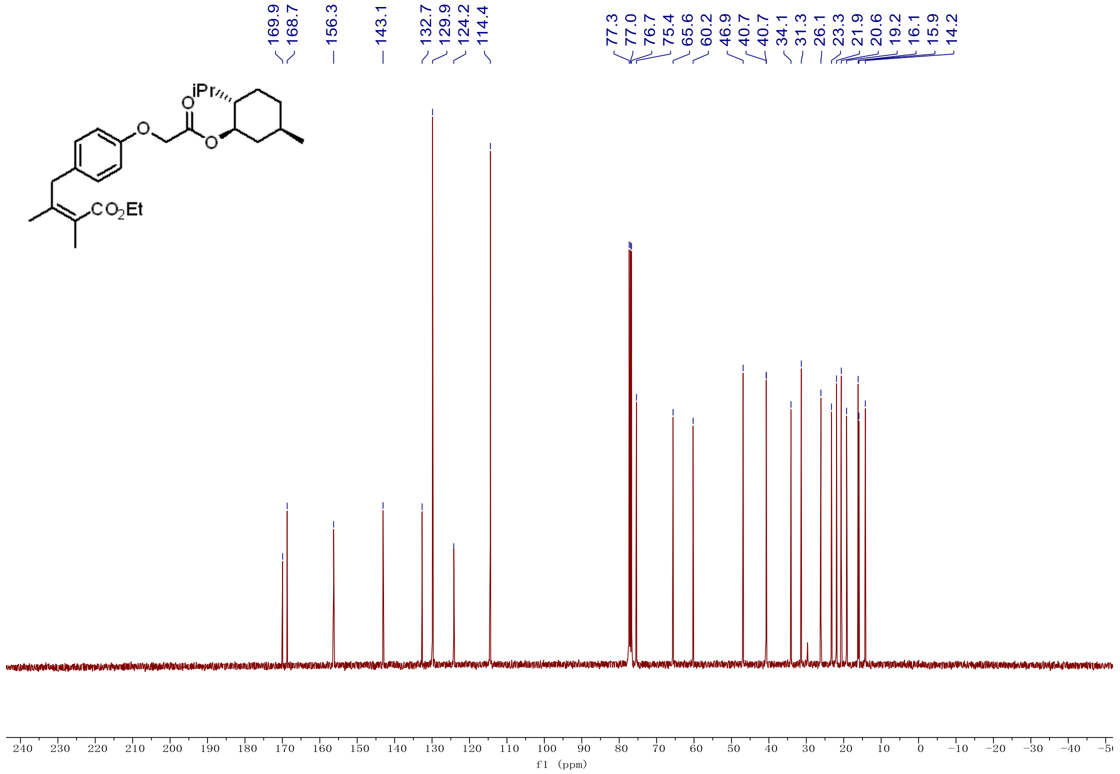

^{13}C NMR spectrum for compound **4f**

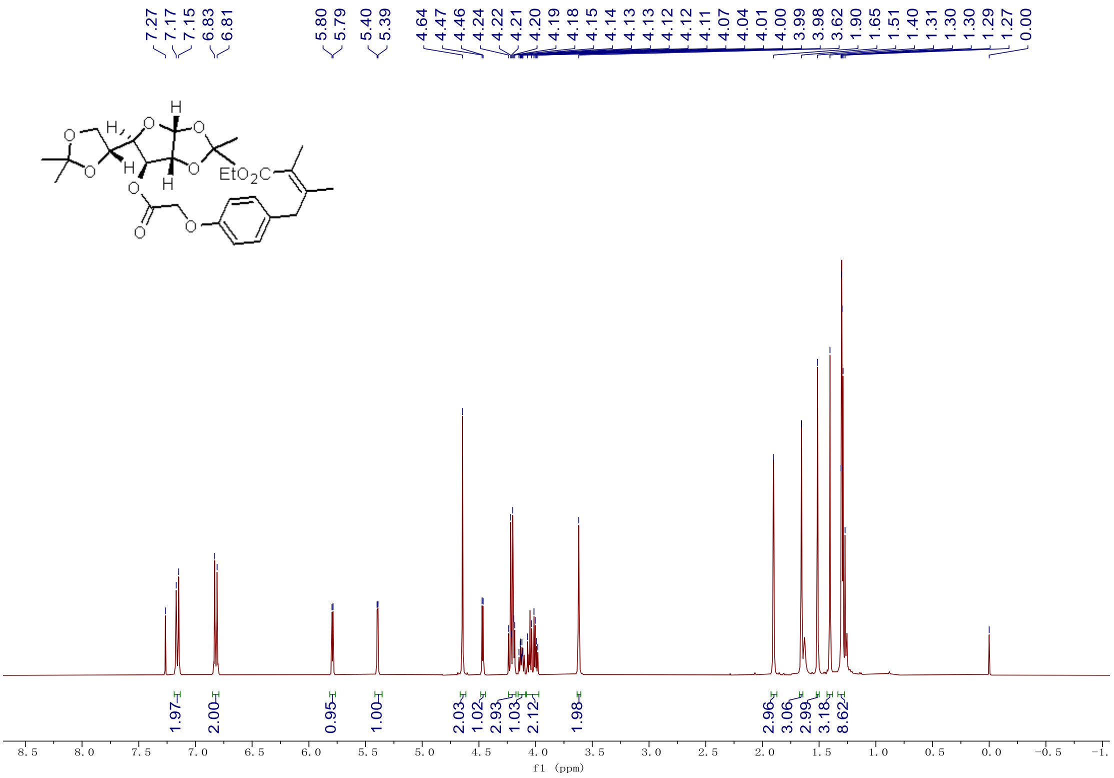

^{1}H NMR spectrum for compound **4g**

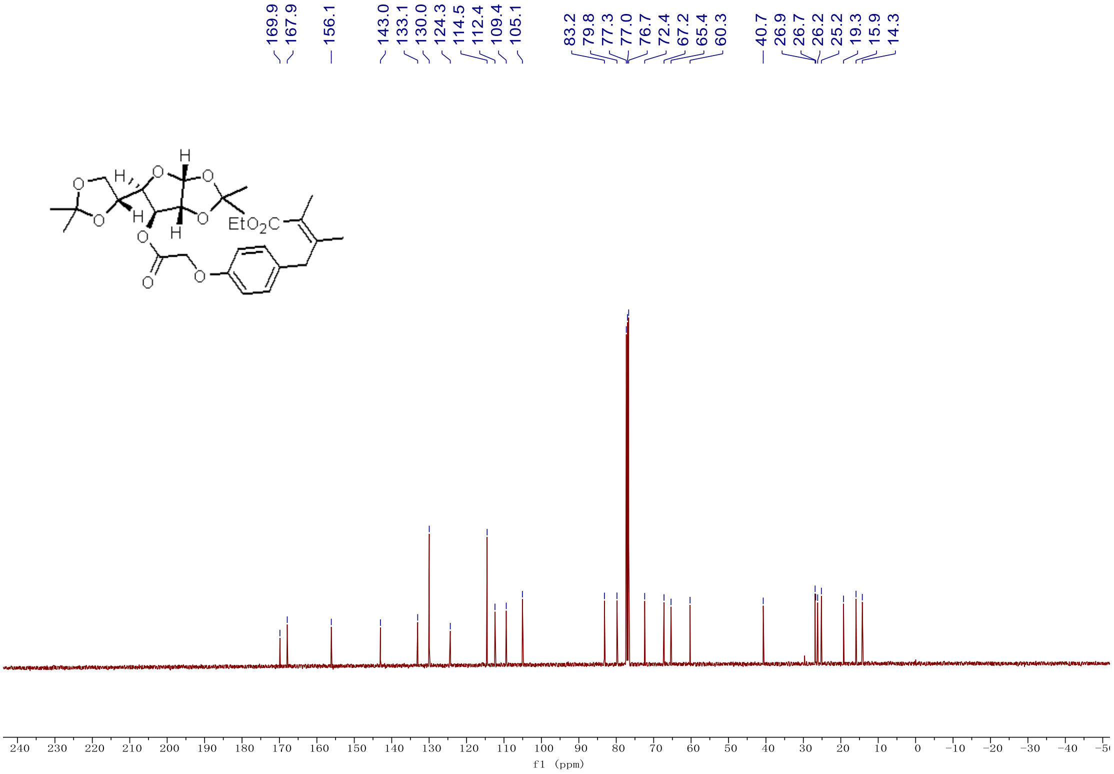

^{13}C NMR spectrum for compound **4g**

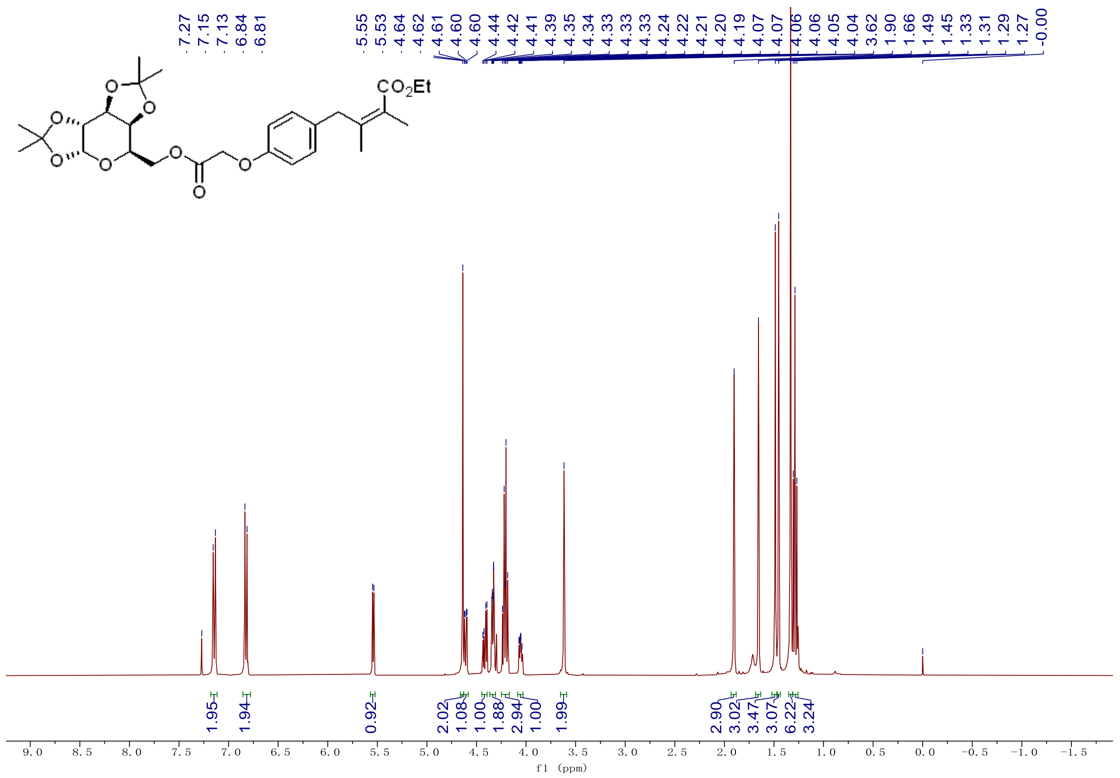

^{1}H NMR spectrum for compound **4h**

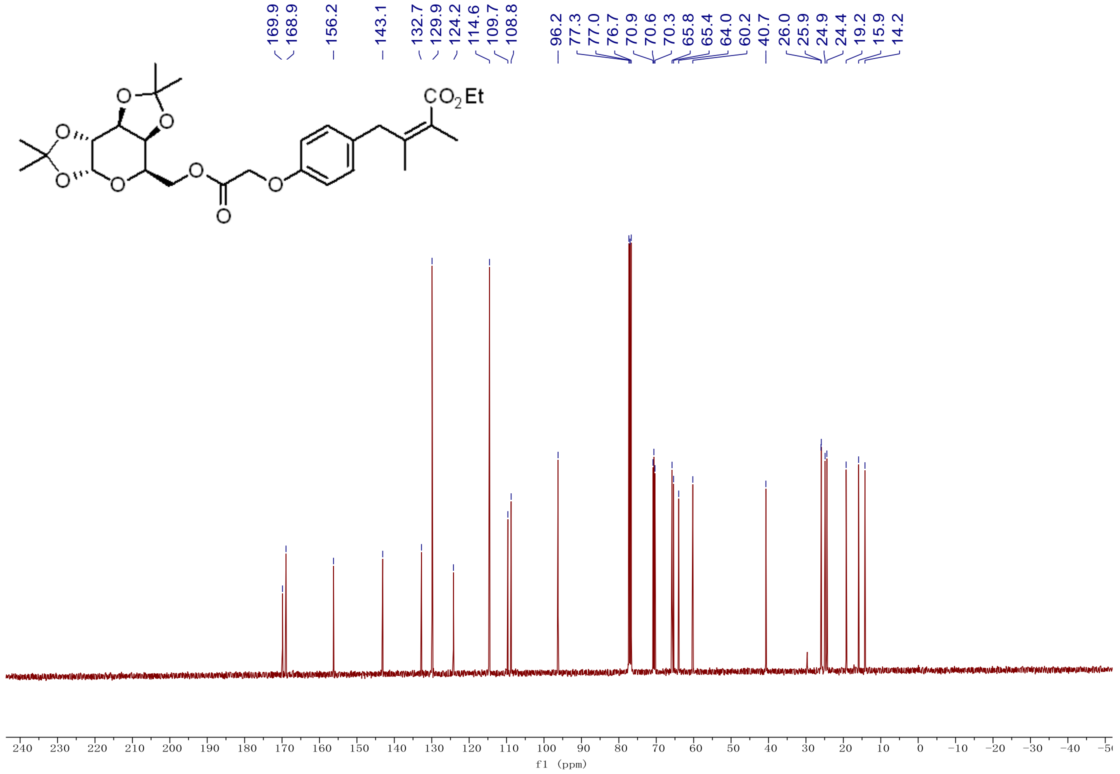

^{13}C NMR spectrum for compound **4h**

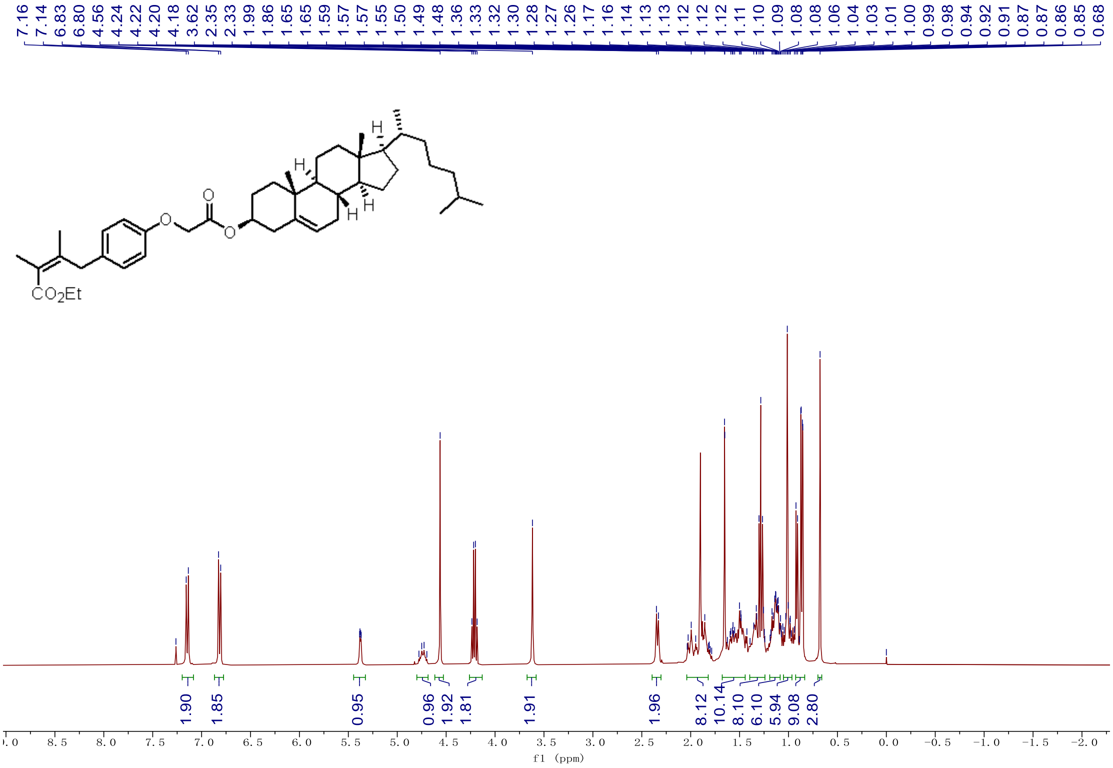

^{1}H NMR spectrum for compound **4i**

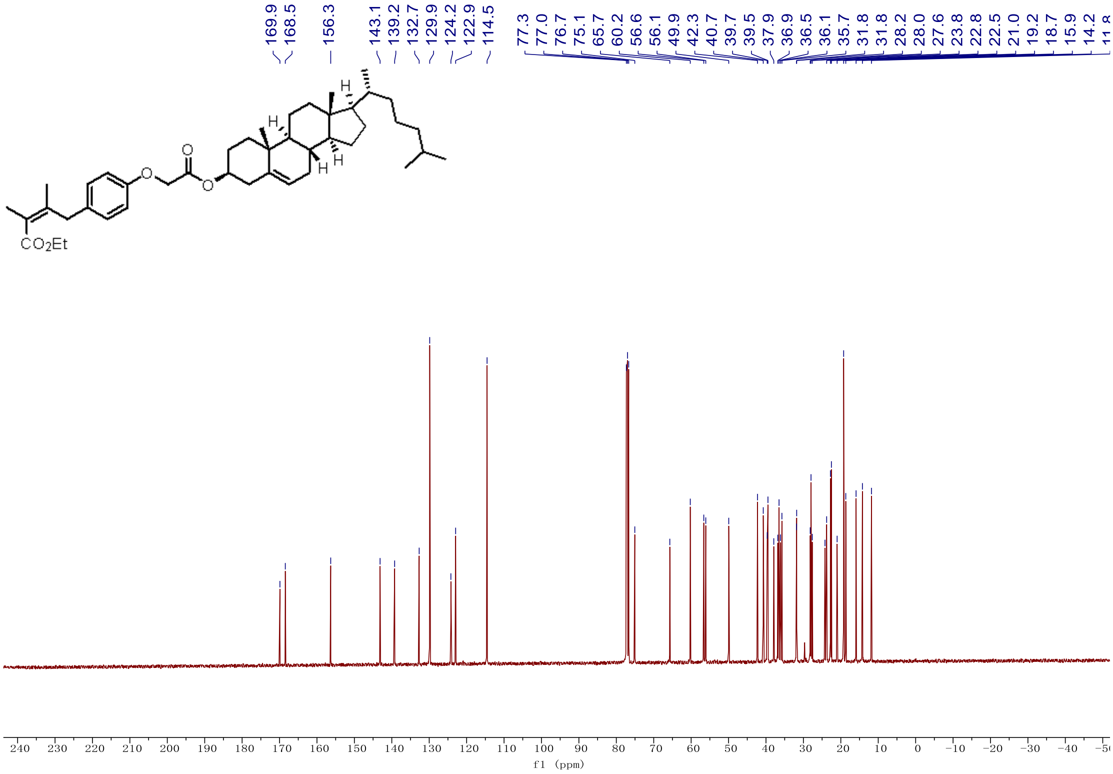

^{13}C NMR spectrum for compound **4i**

.

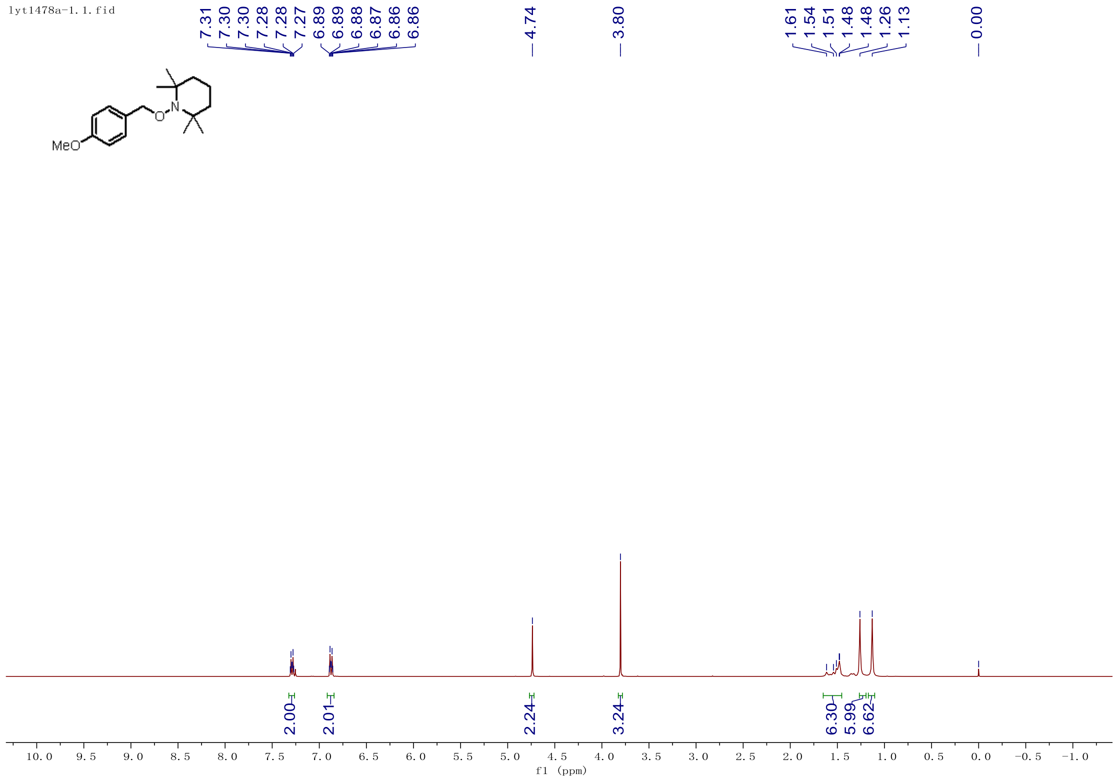

^{1}H NMR spectrum for compound **8**

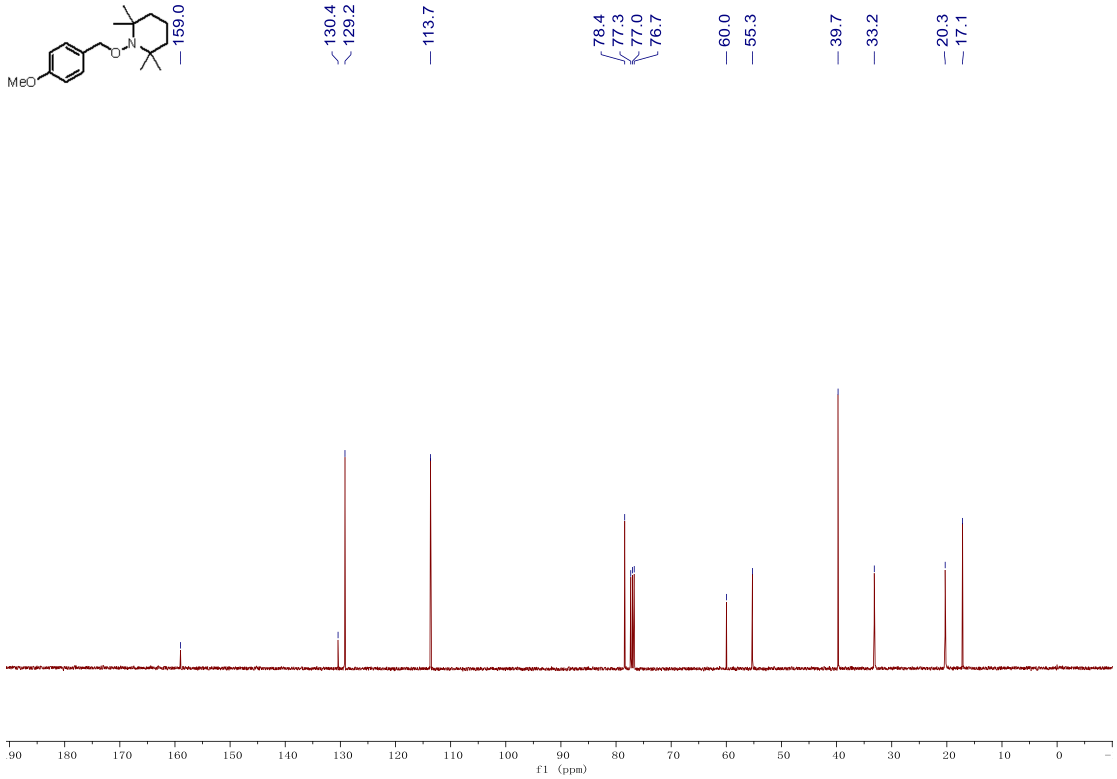

^{13}C NMR spectrum for compound **8**

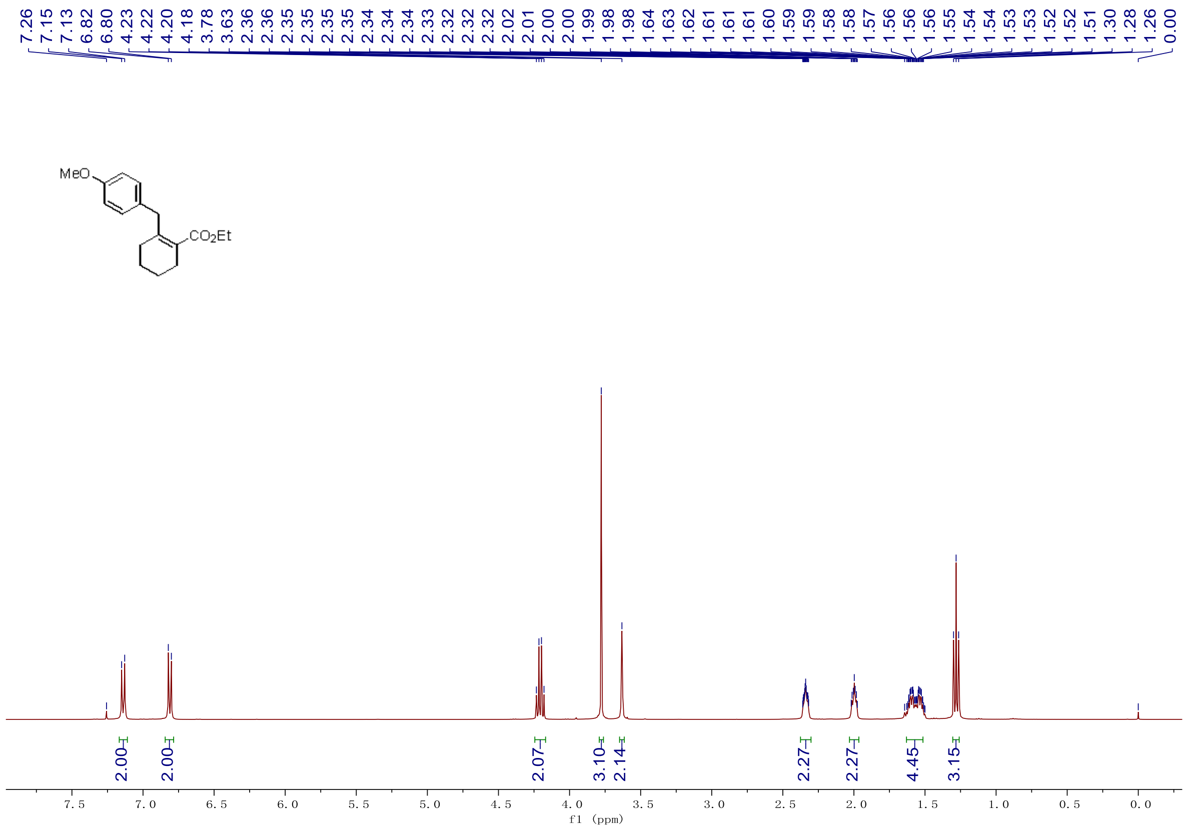

^{1}H NMR spectrum for compound **10**

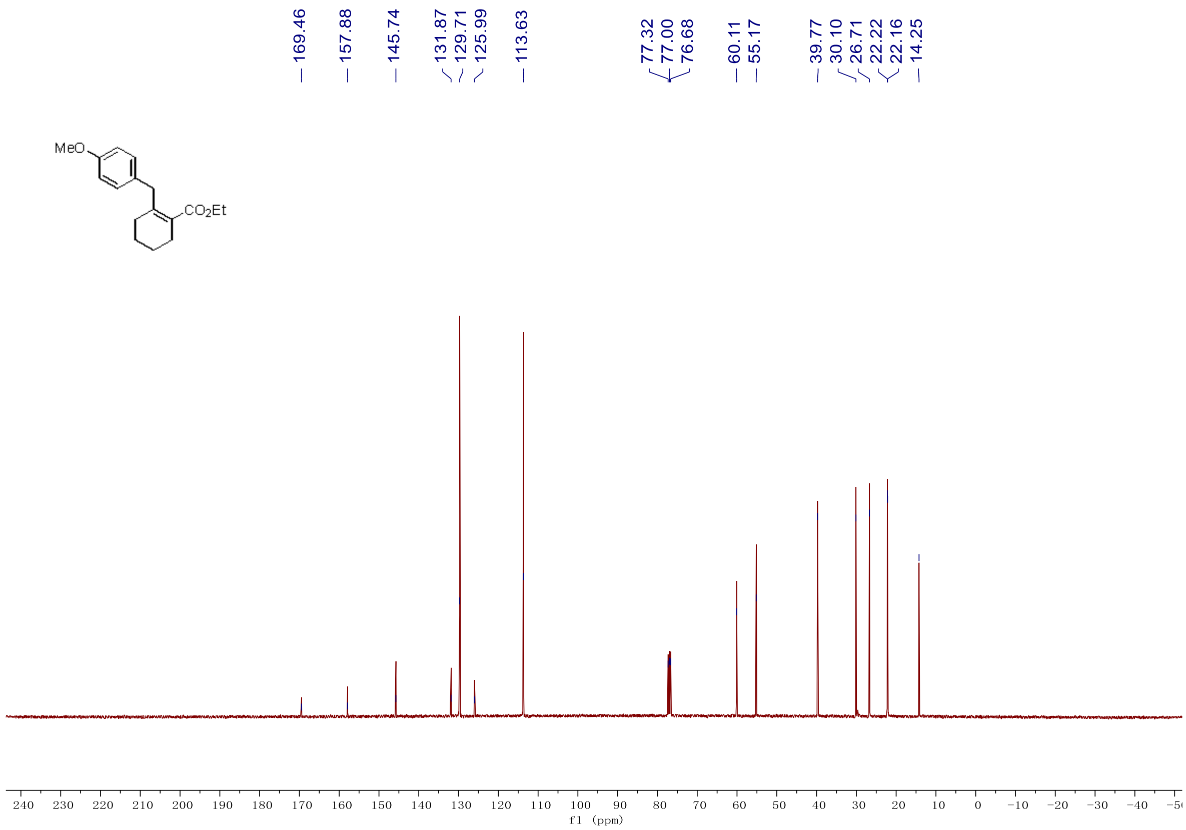

^{13}C NMR spectrum for compound **10**

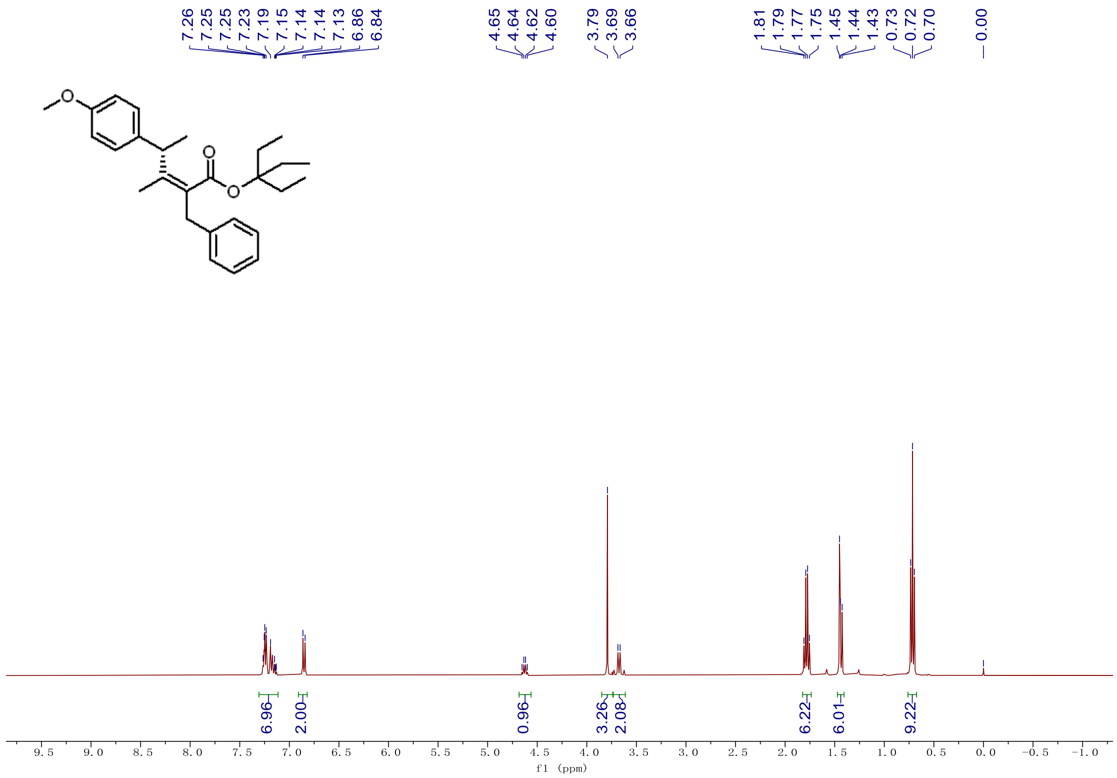

^{1}H NMR spectrum for compound **5a**

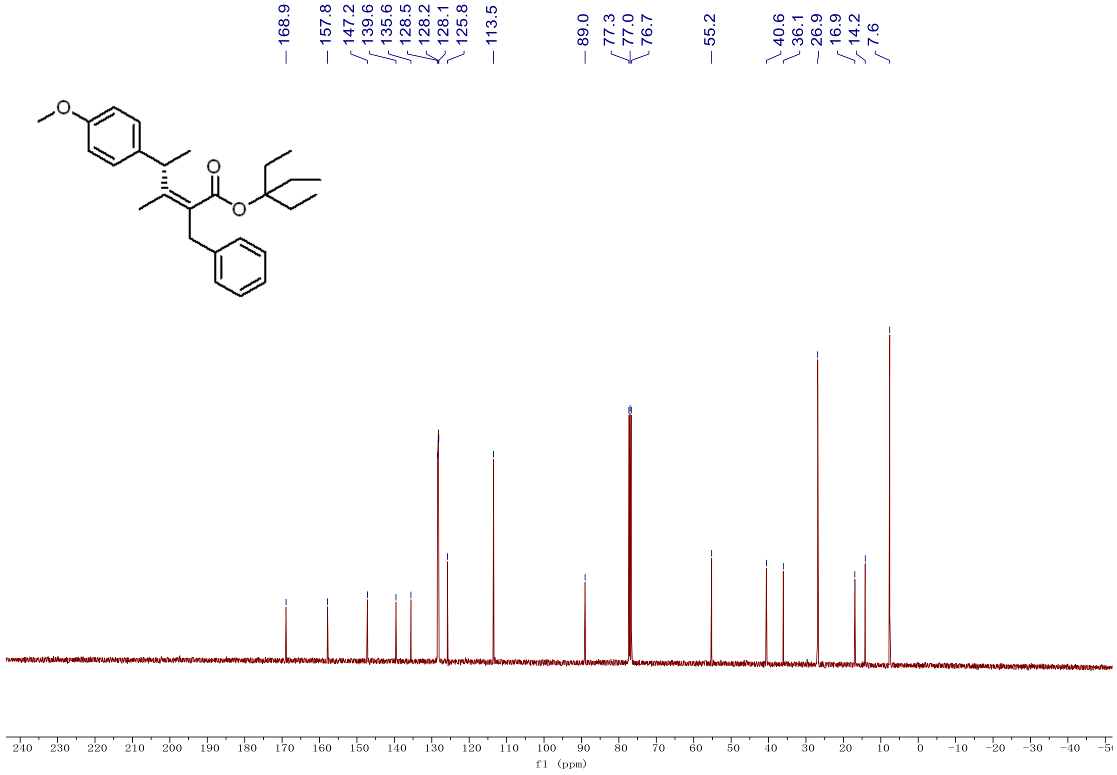

^{13}C NMR spectrum for compound **5a**

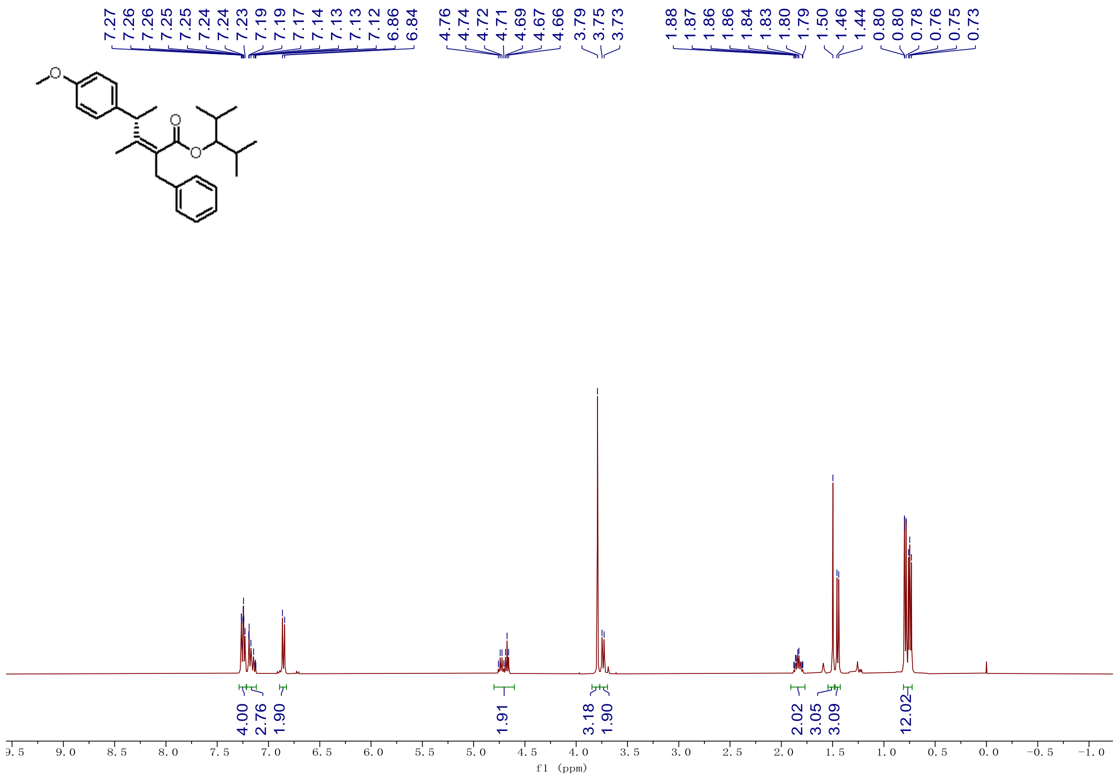

^{1}H NMR spectrum for compound **5b**

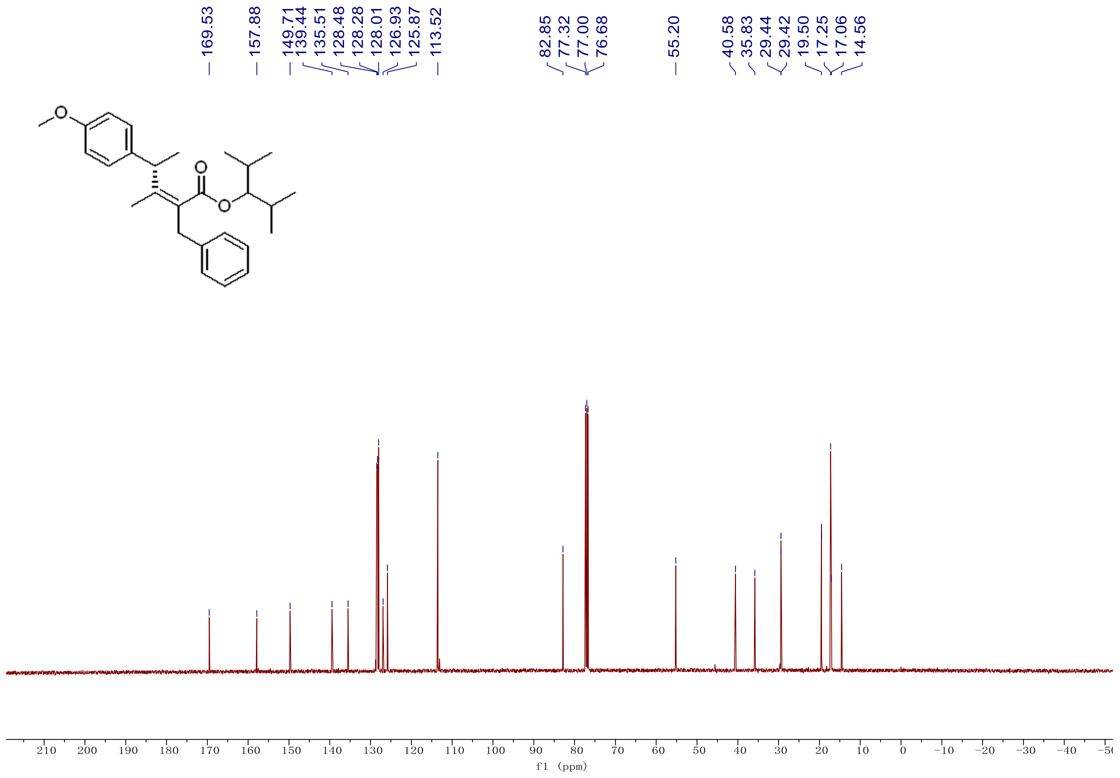

^{13}C NMR spectrum for compound **5b**

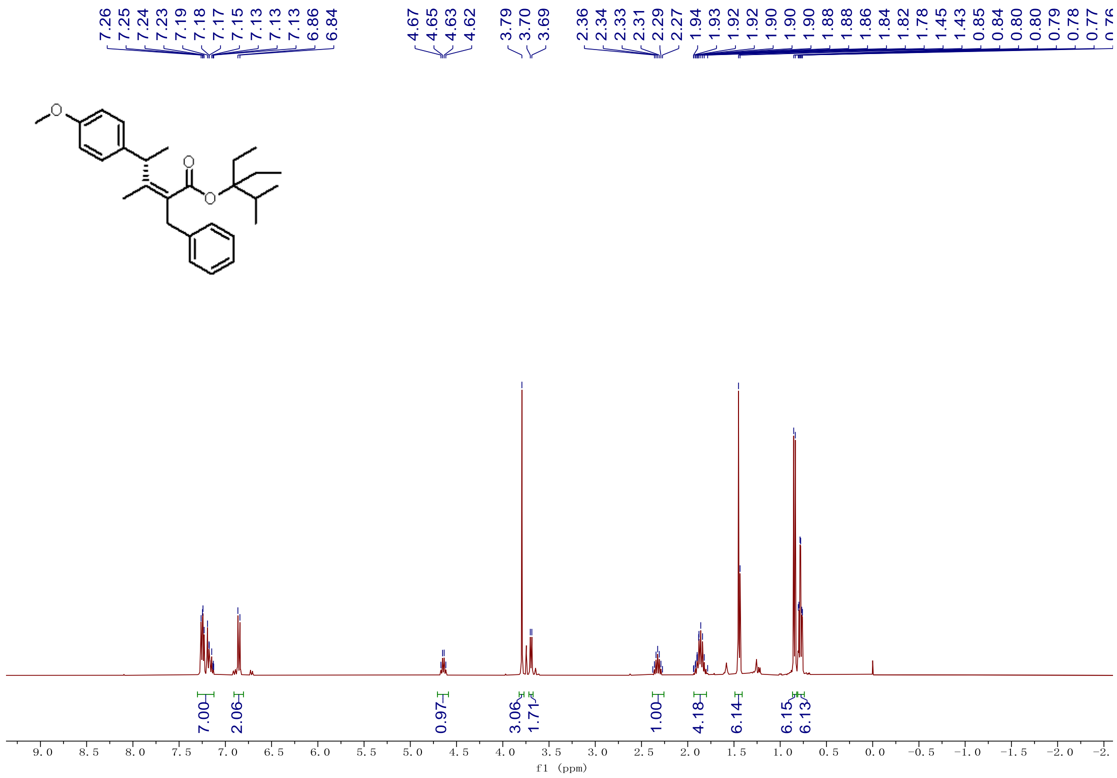

^{1}H NMR spectrum for compound **5c**

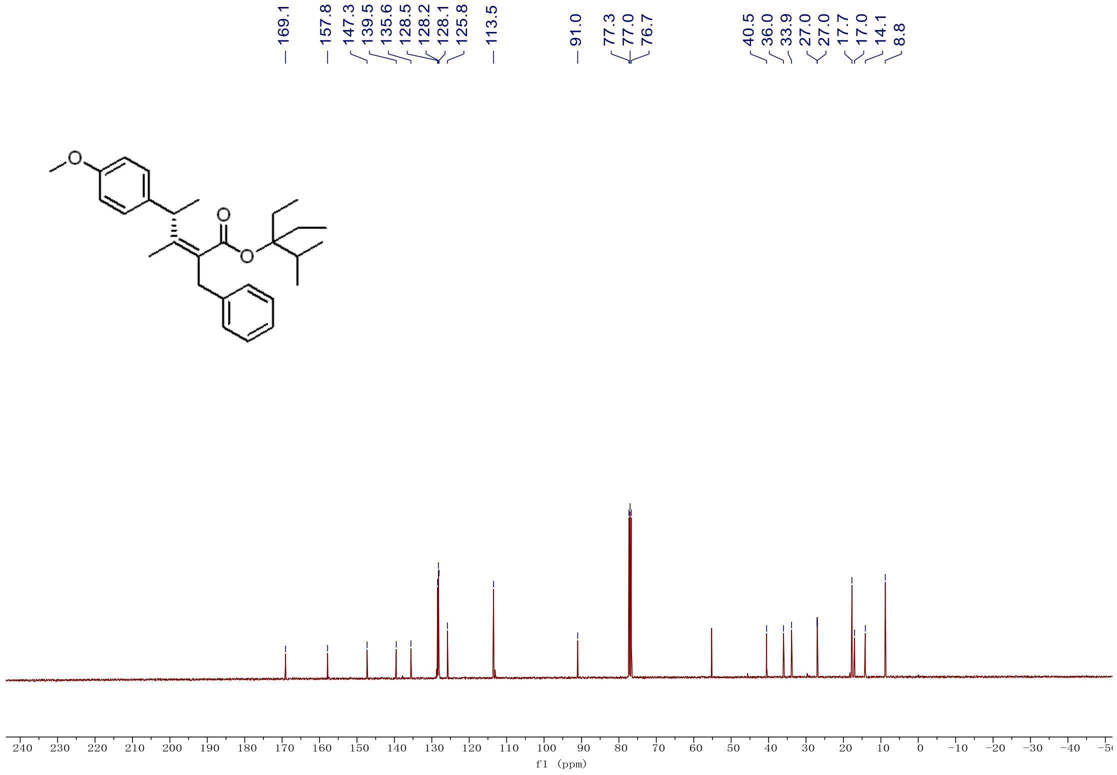

^{13}C NMR spectrum for compound **5c**

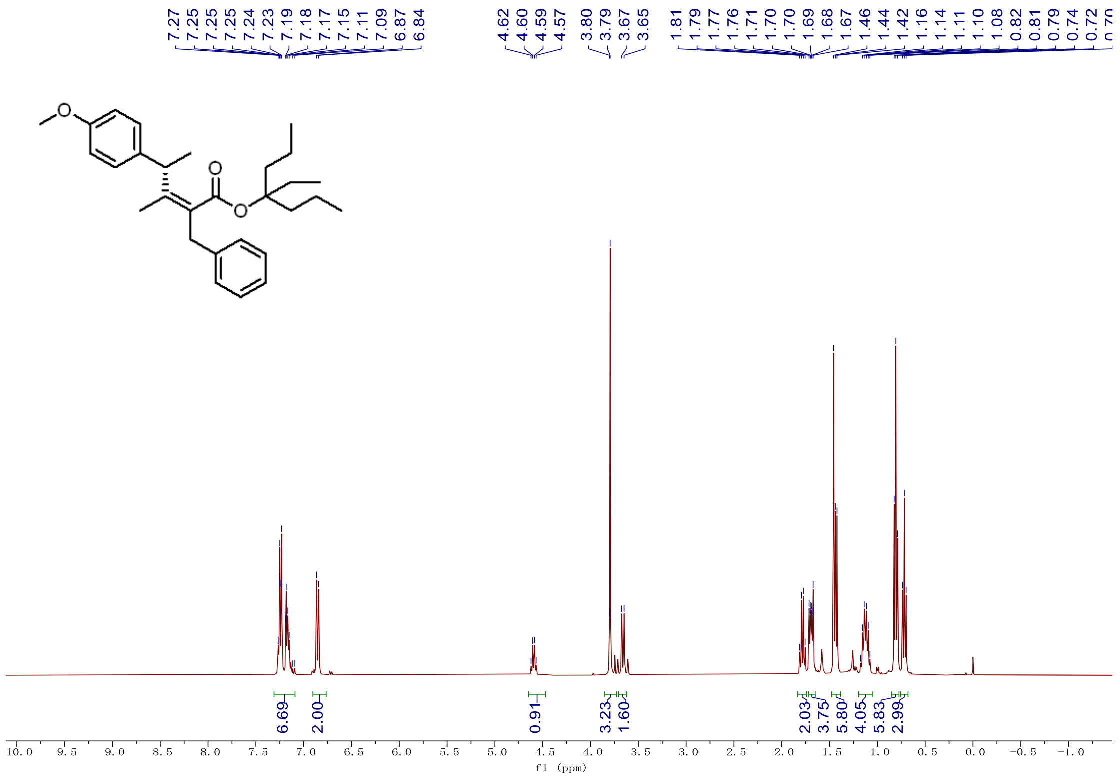

^{1}H NMR spectrum for compound **5d**

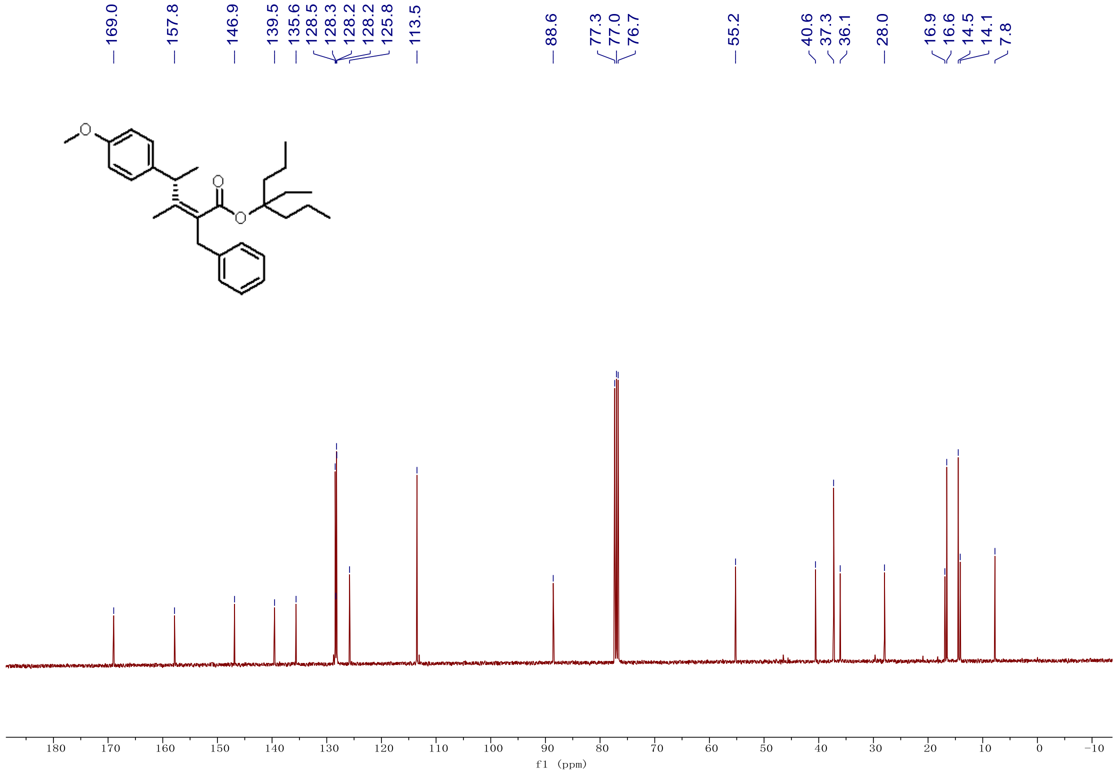

^{13}C NMR spectrum for compound **5d**

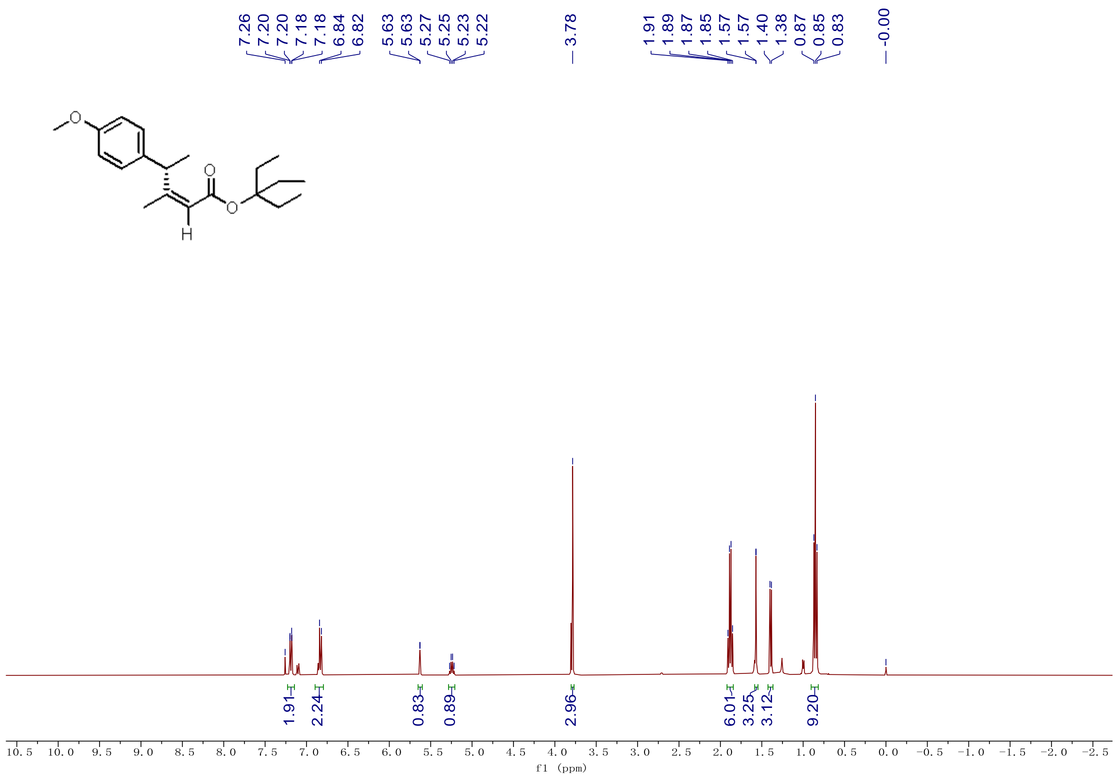

^{1}H NMR spectrum for compound **5e**

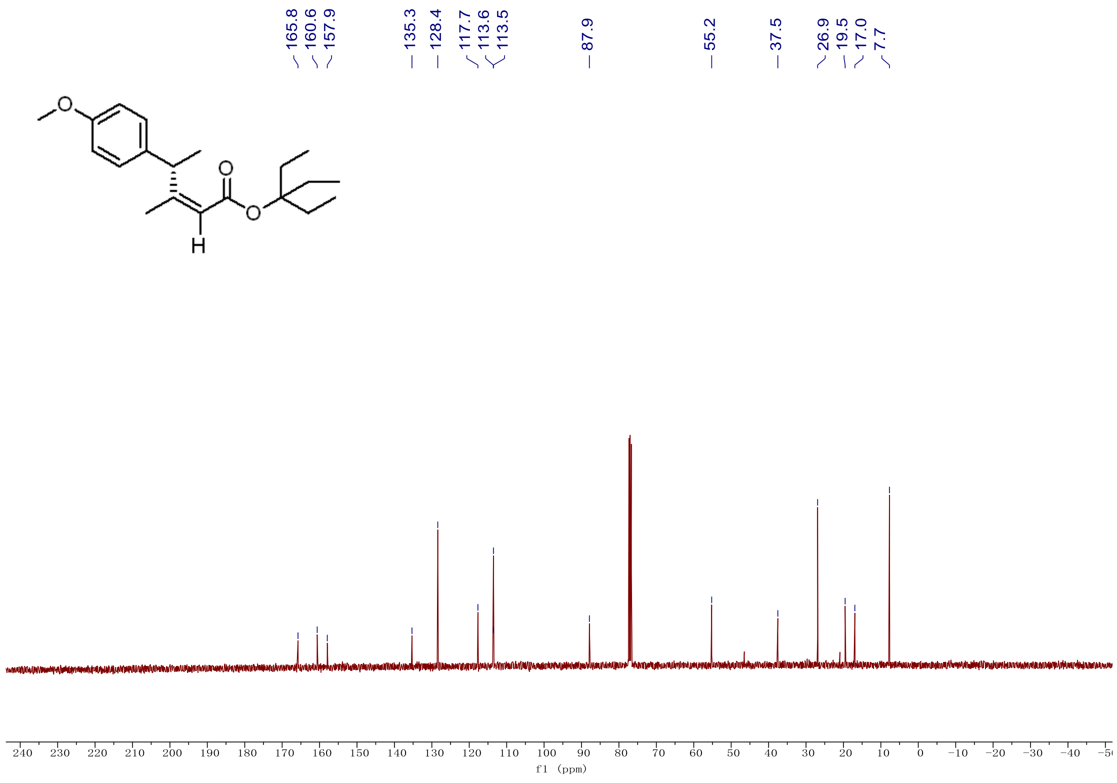

^{13}C NMR spectrum for compound **5e**

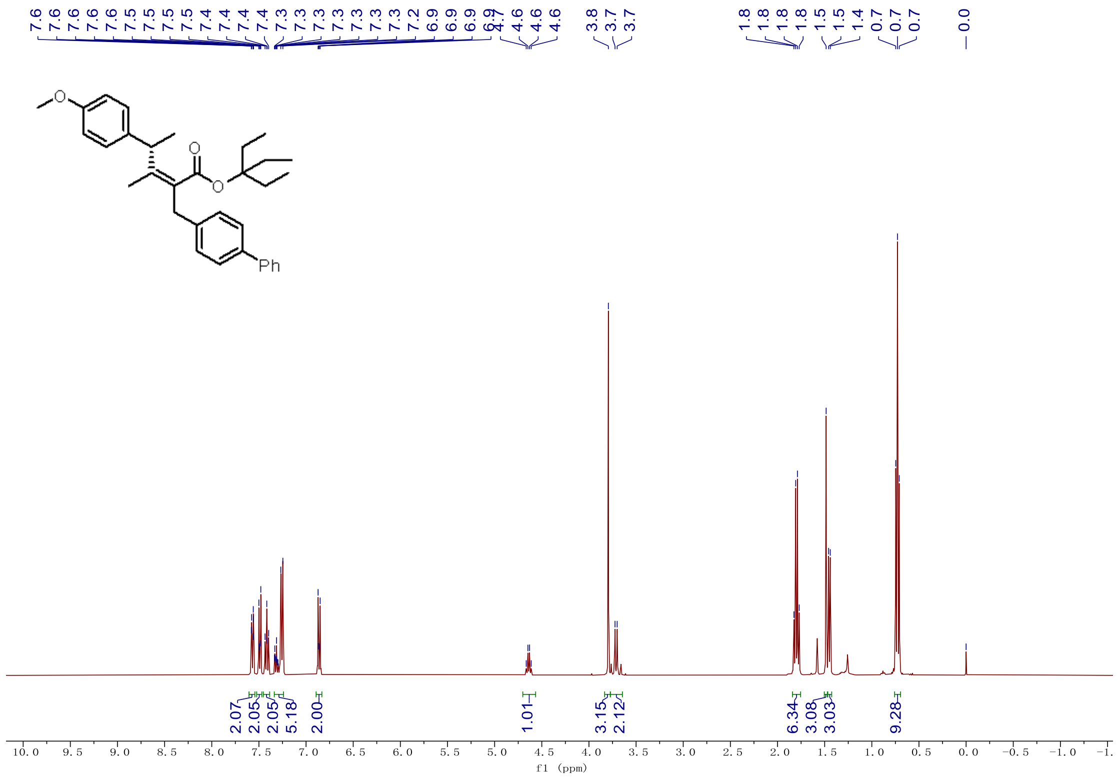

^{1}H NMR spectrum for compound **5f**

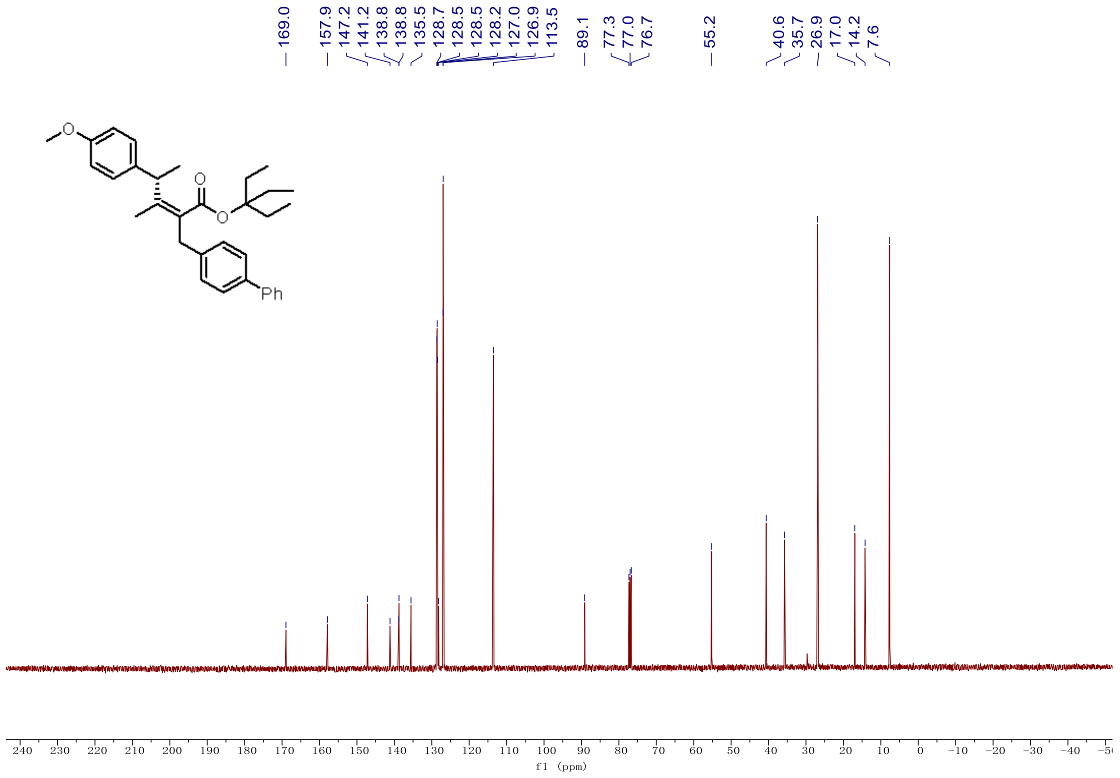

^{13}C NMR spectrum for compound **5f**

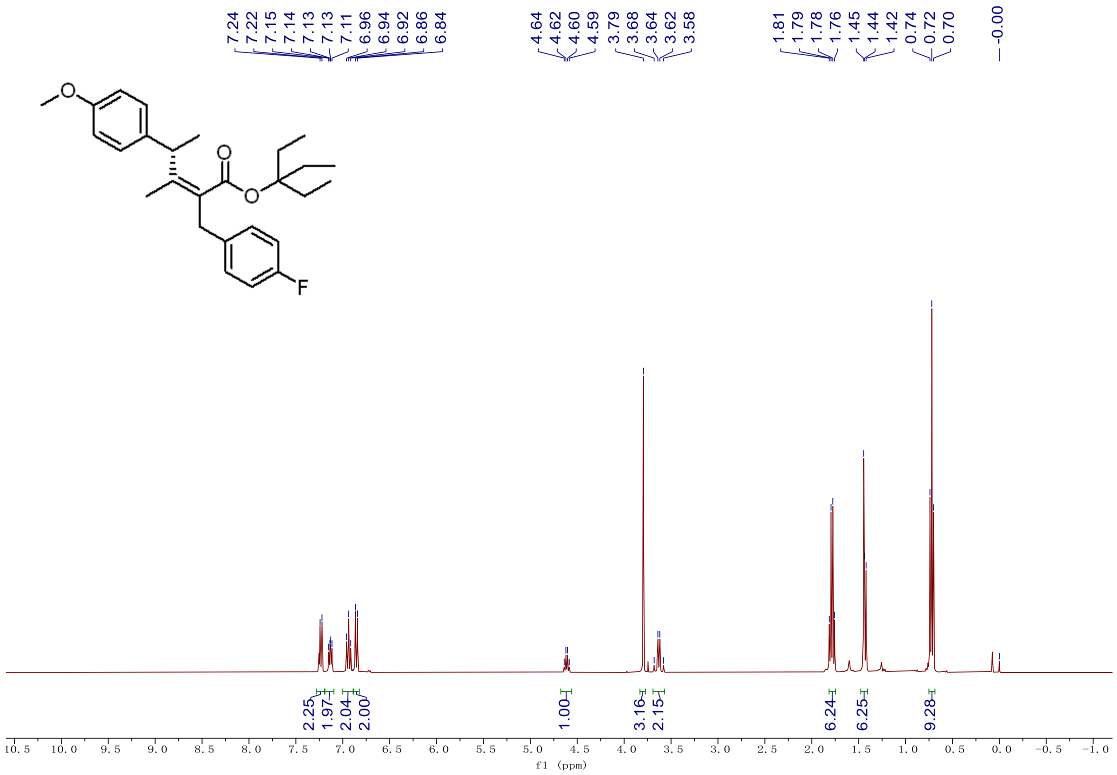

^{1}H NMR spectrum for compound **5g**

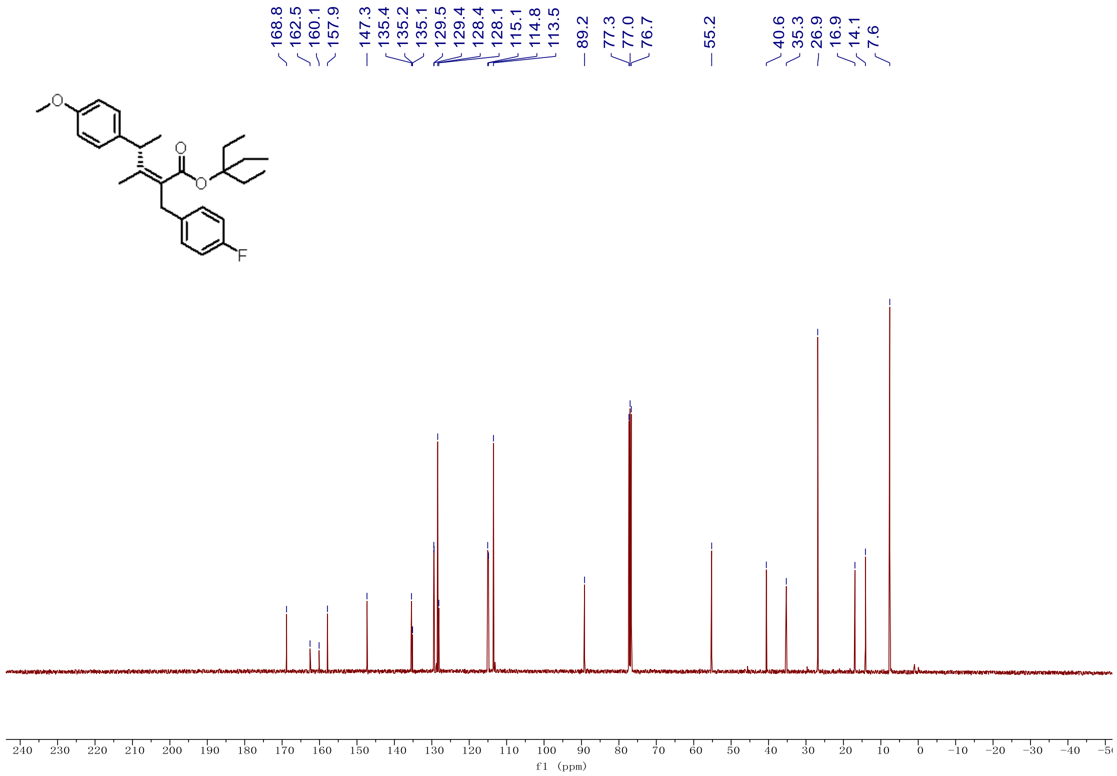

^{13}C NMR spectrum for compound **5g**

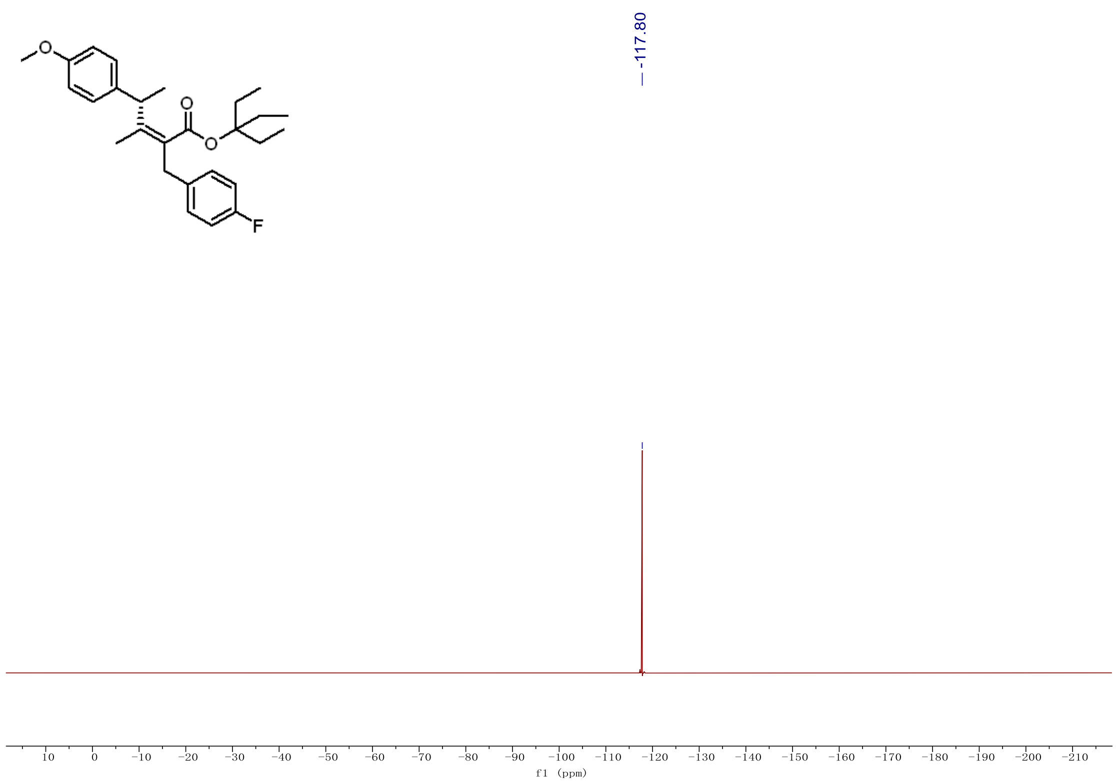

^{19}F NMR spectrum for compound **5g**

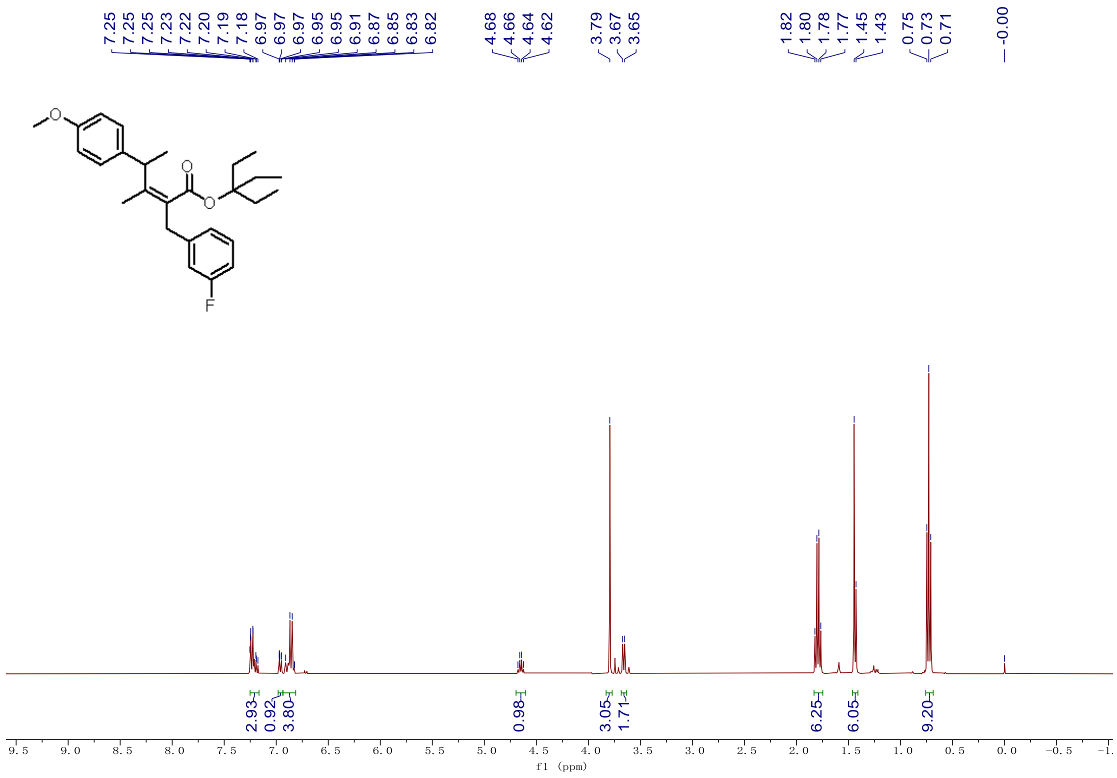

^{1}H NMR spectrum for compound **5h**

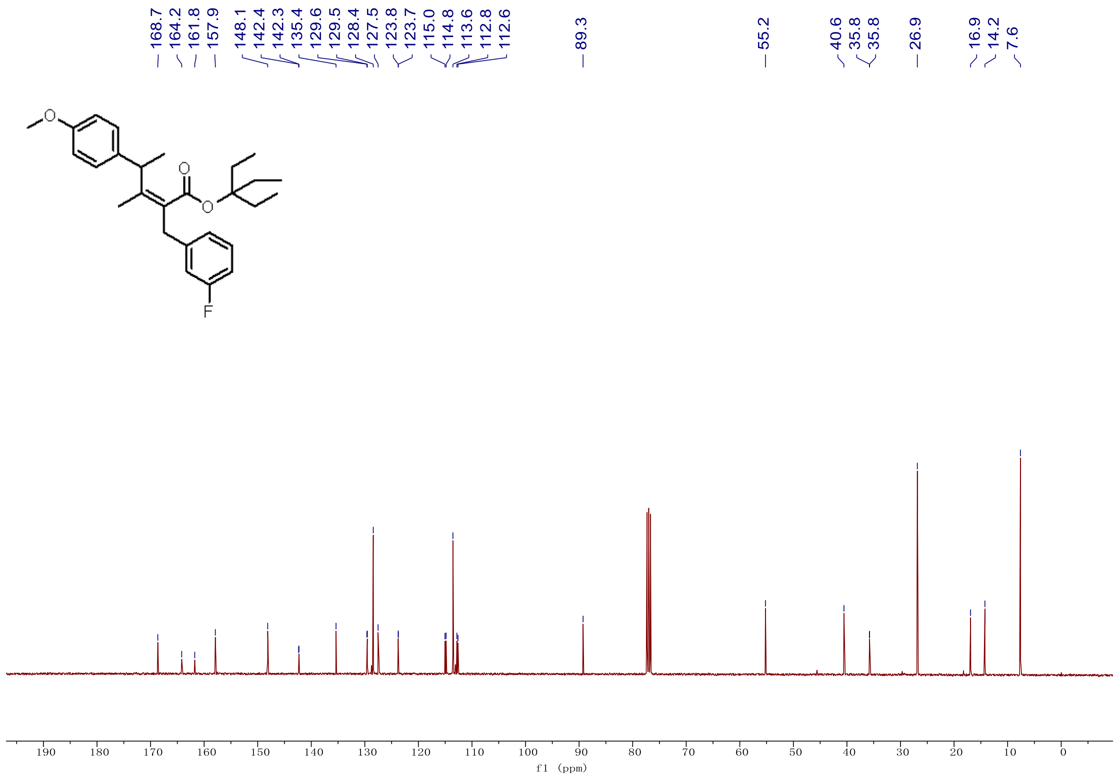

^{13}C NMR spectrum for compound **5h**

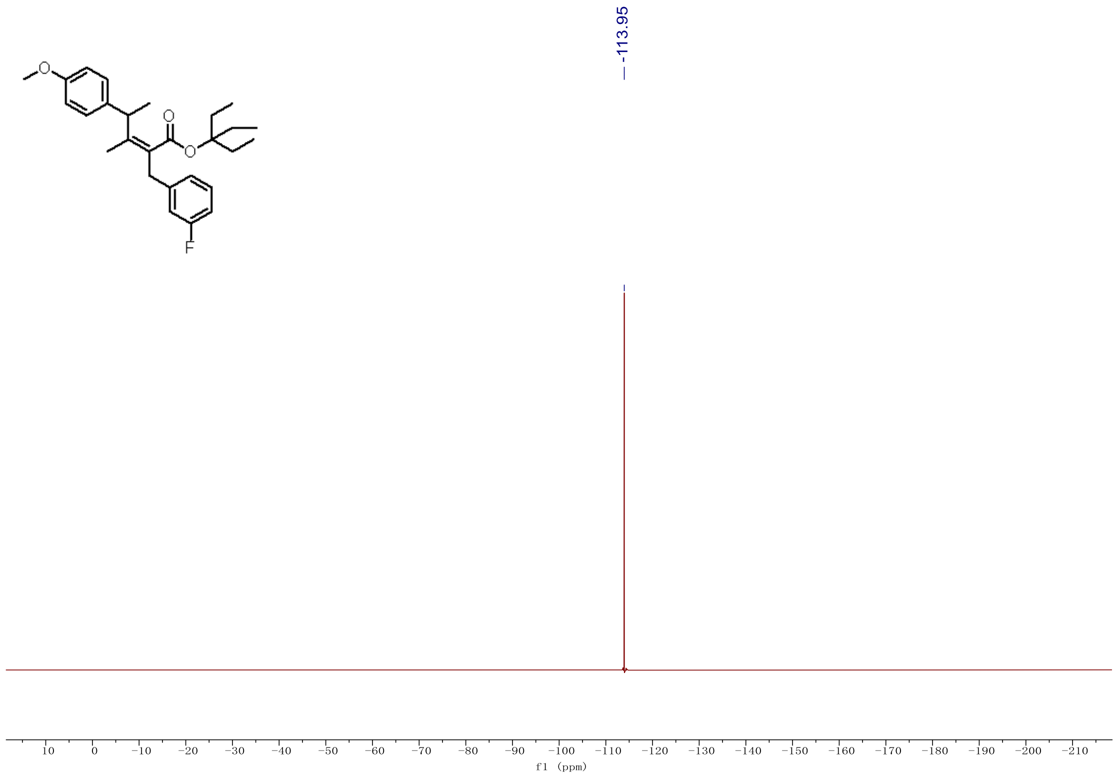

^{19}F NMR spectrum for compound **5h**

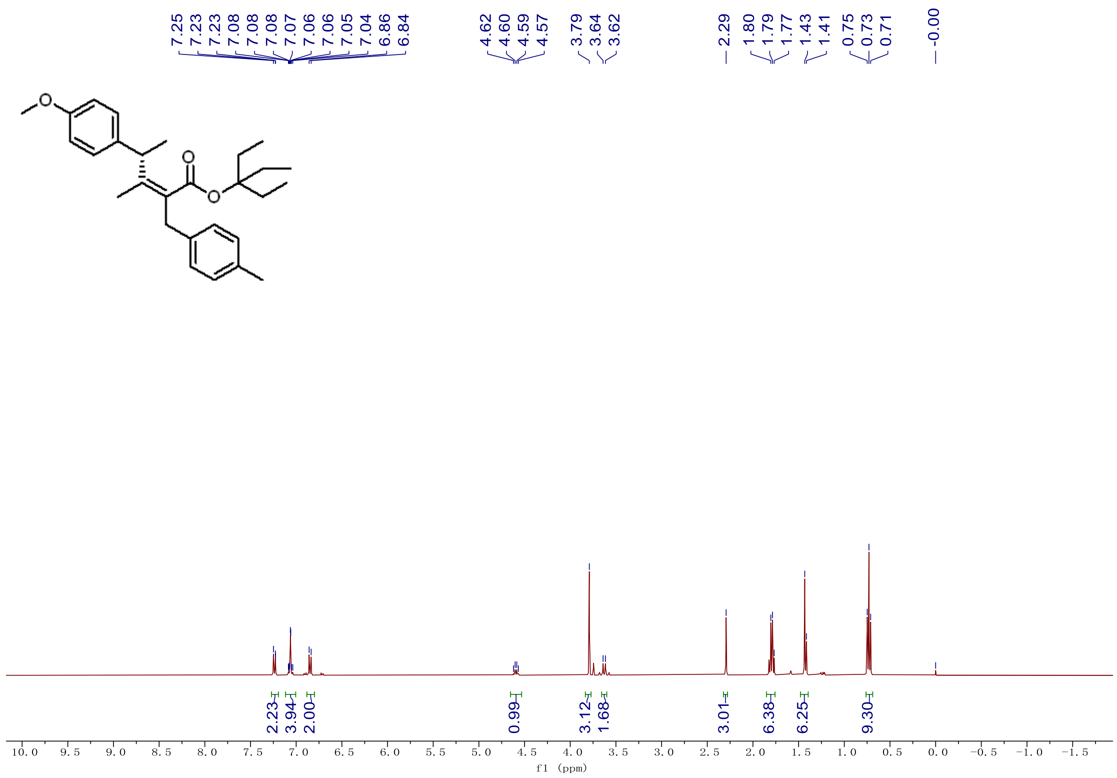

^{1}H NMR spectrum for compound **5i**

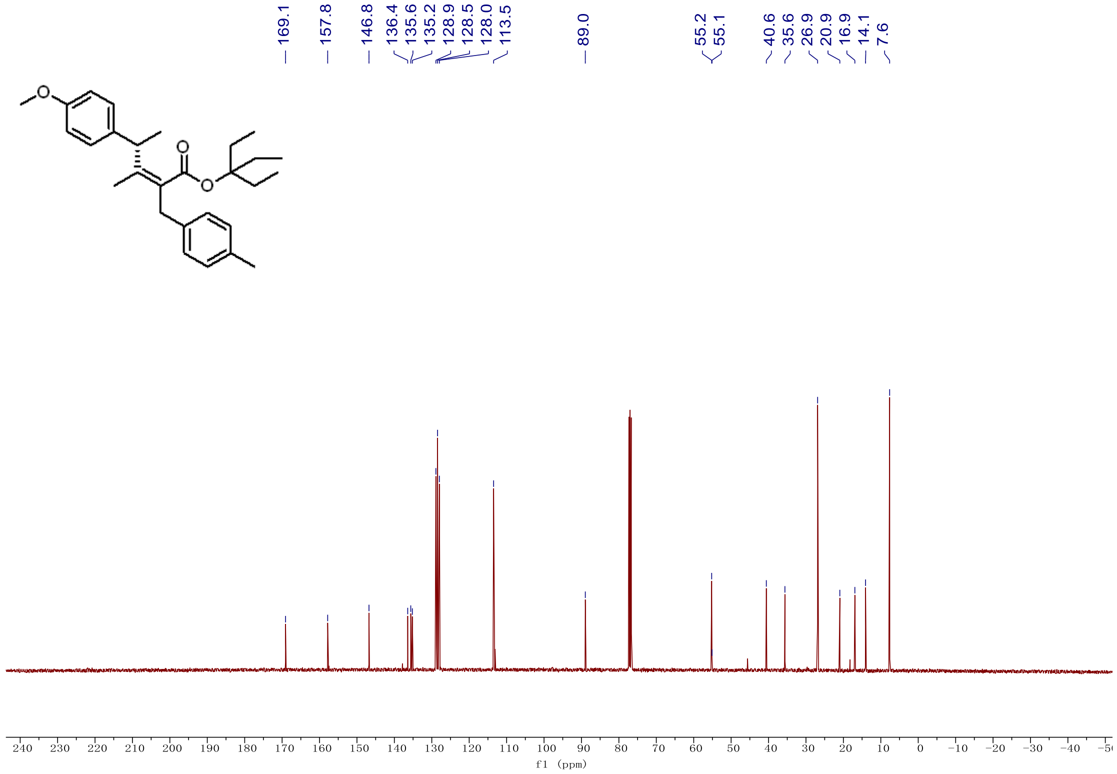

^{13}C NMR spectrum for compound **5i**

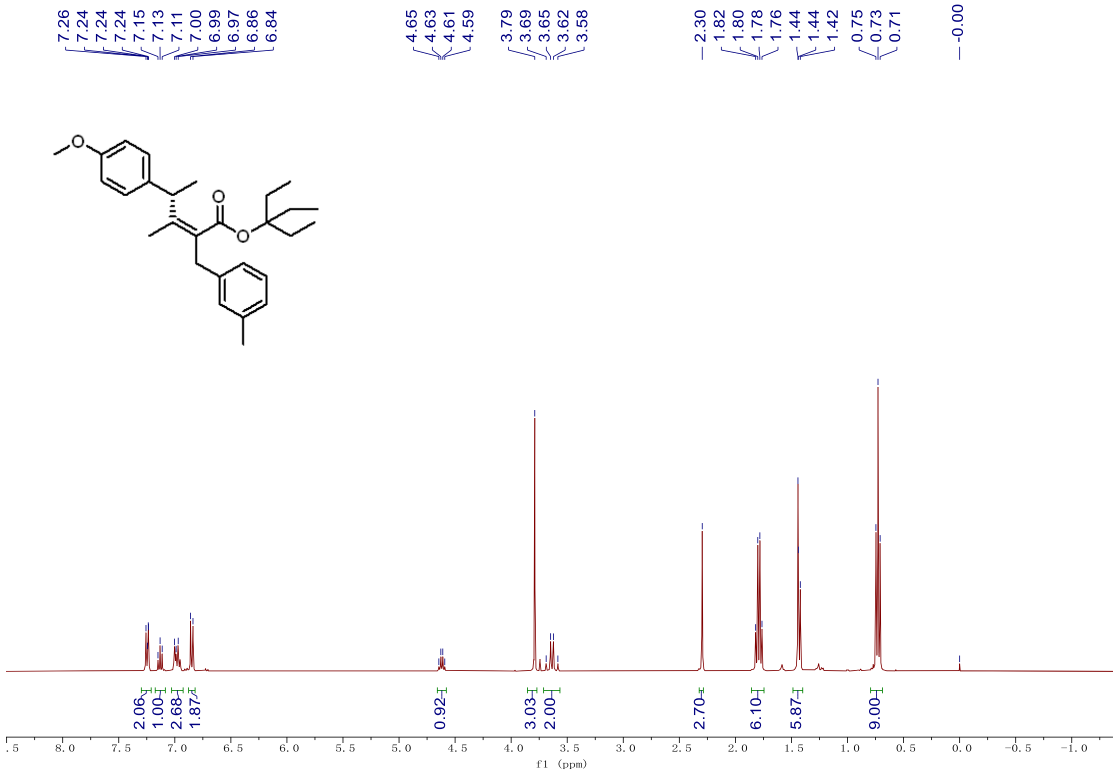

^{1}H NMR spectrum for compound **5j**

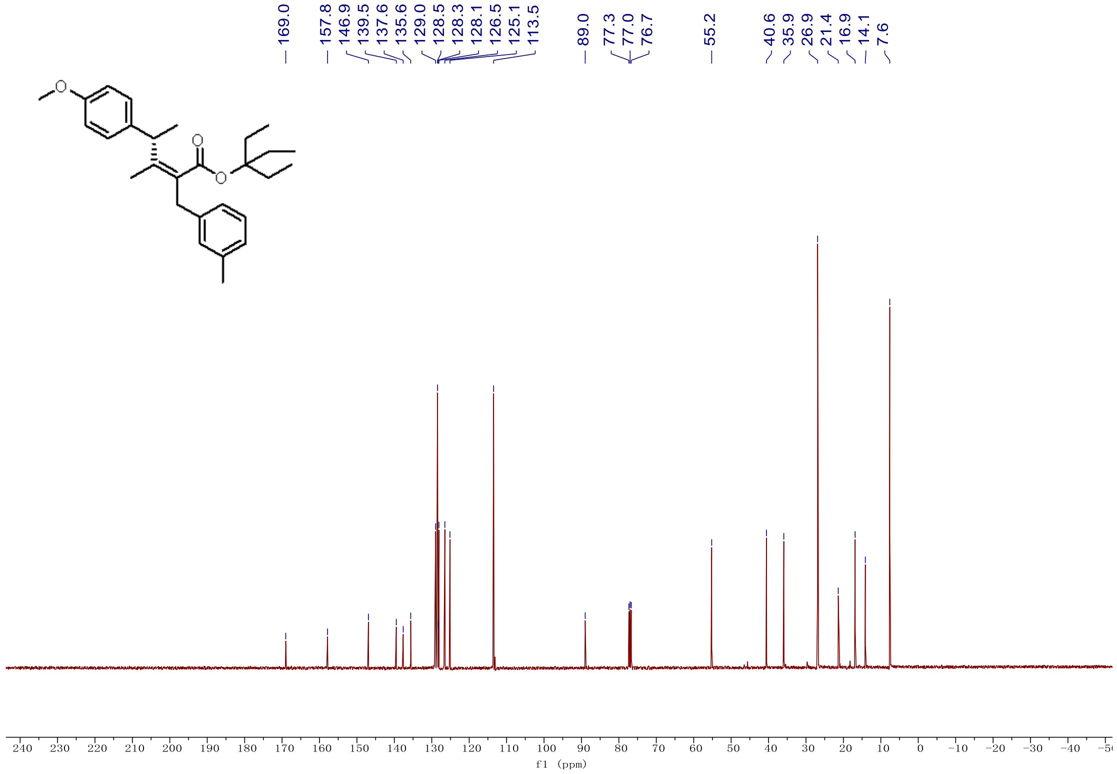

^{13}C NMR spectrum for compound **5j**

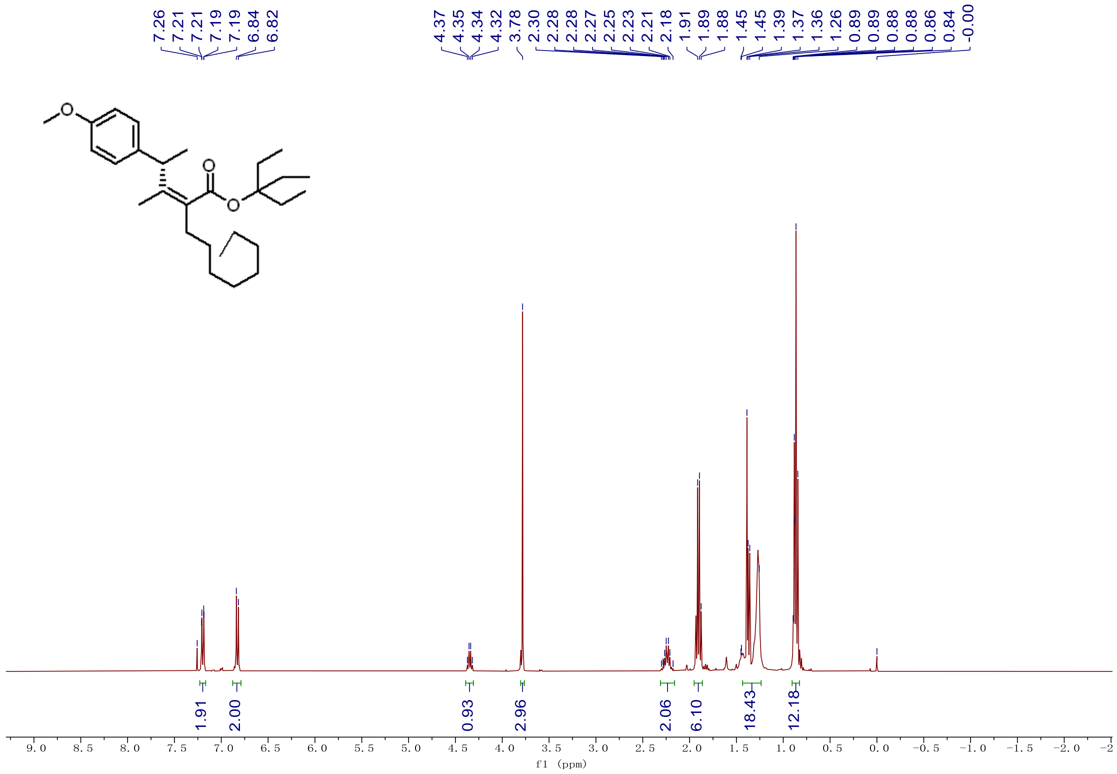

^{1}H NMR spectrum for compound **5k**

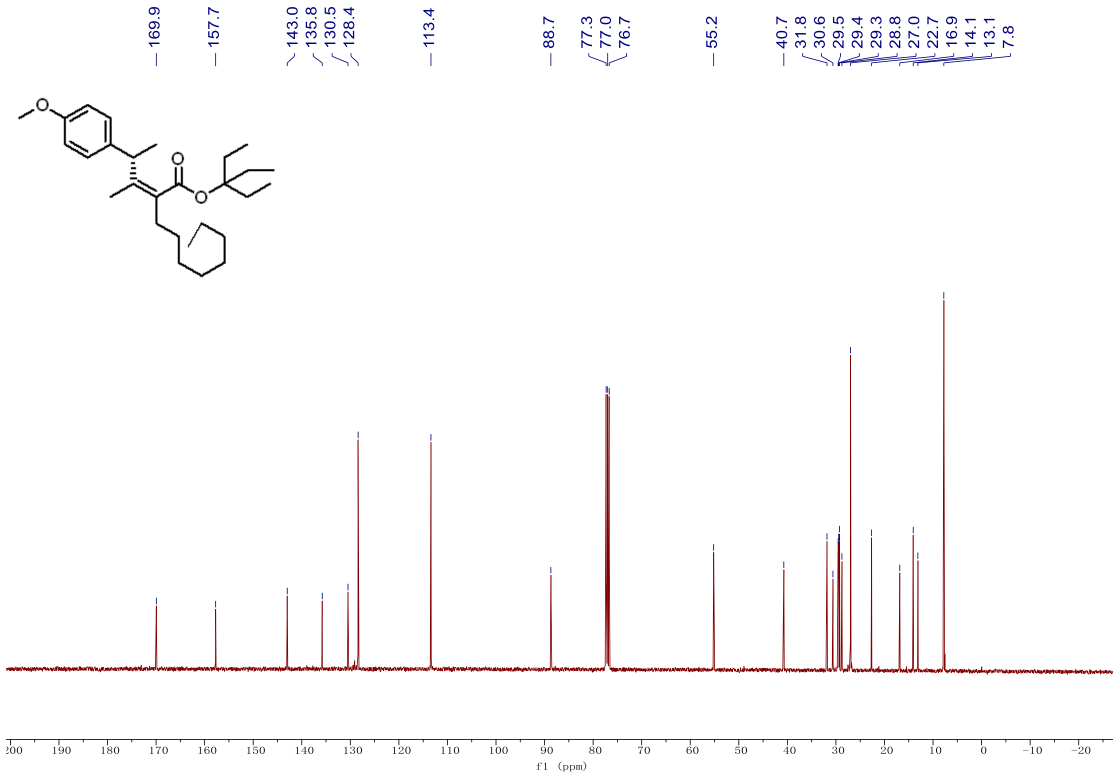

^{13}C NMR spectrum for compound **5k**

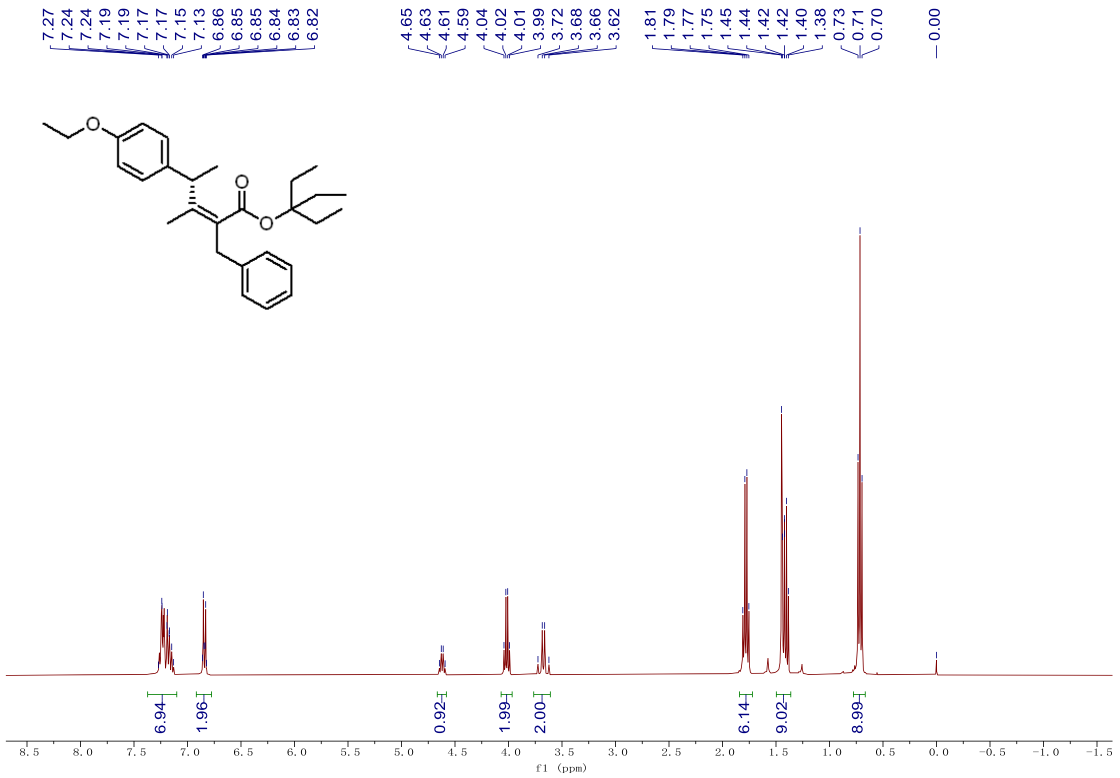

^{1}H NMR spectrum for compound **5l**

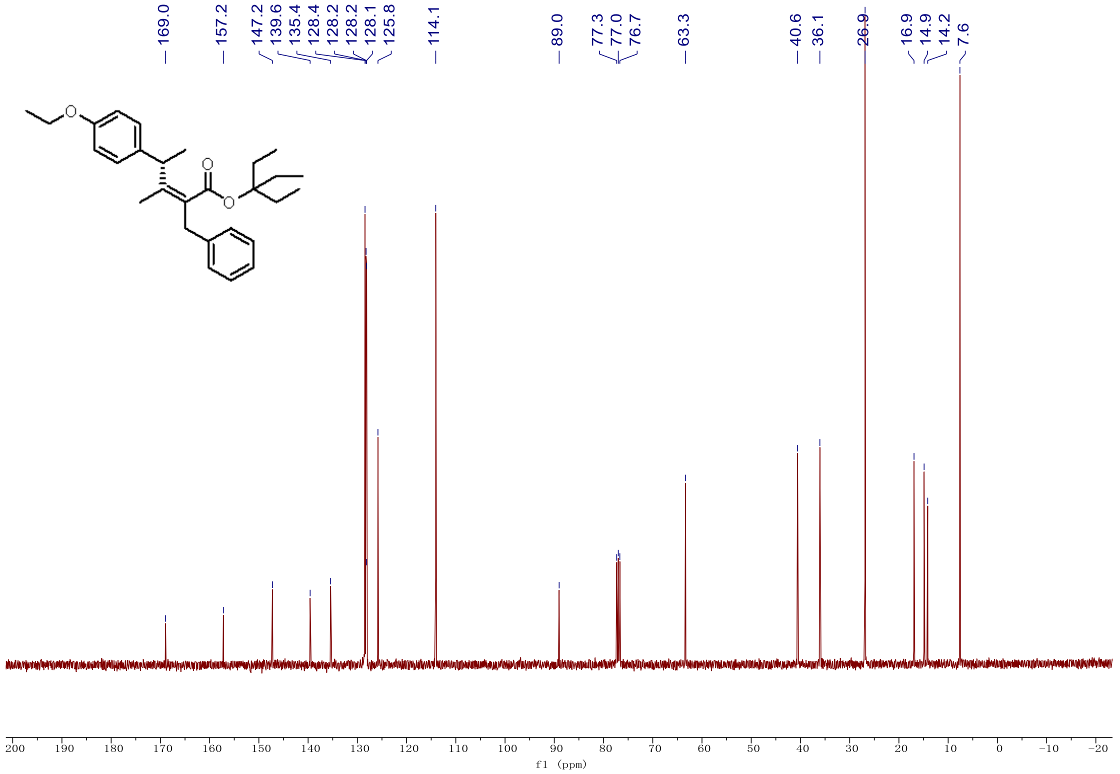

^{13}C NMR spectrum for compound **5l**

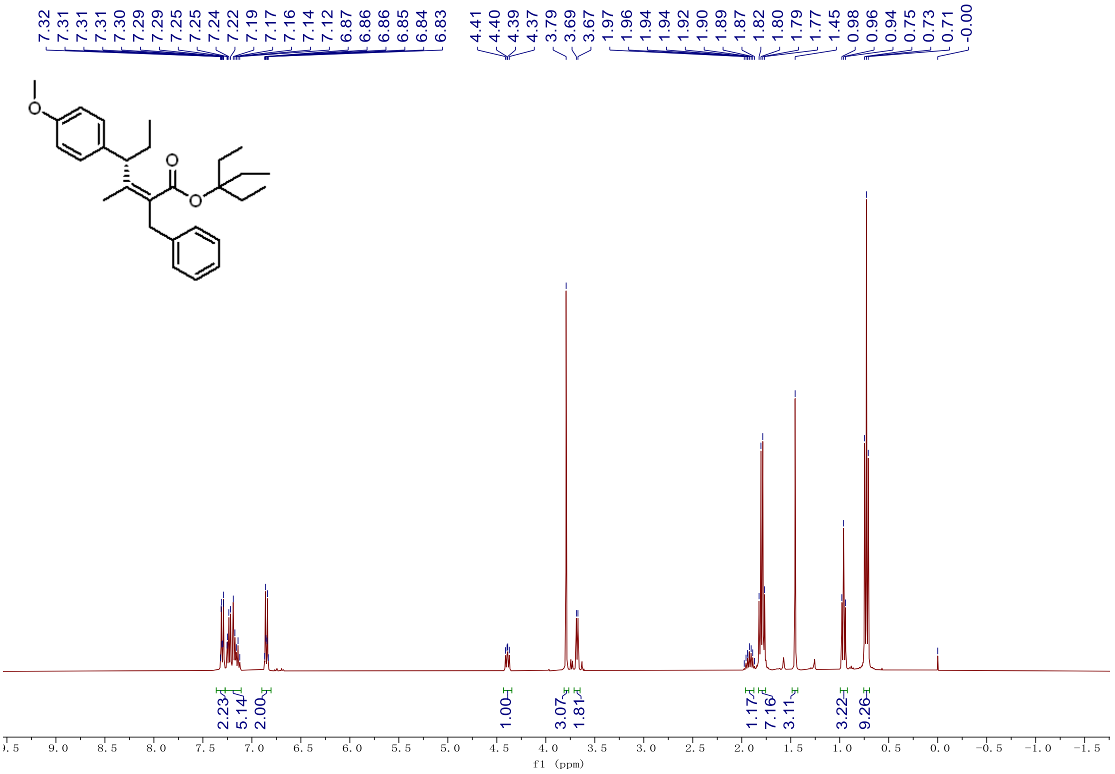

^{1}H NMR spectrum for compound **5m**

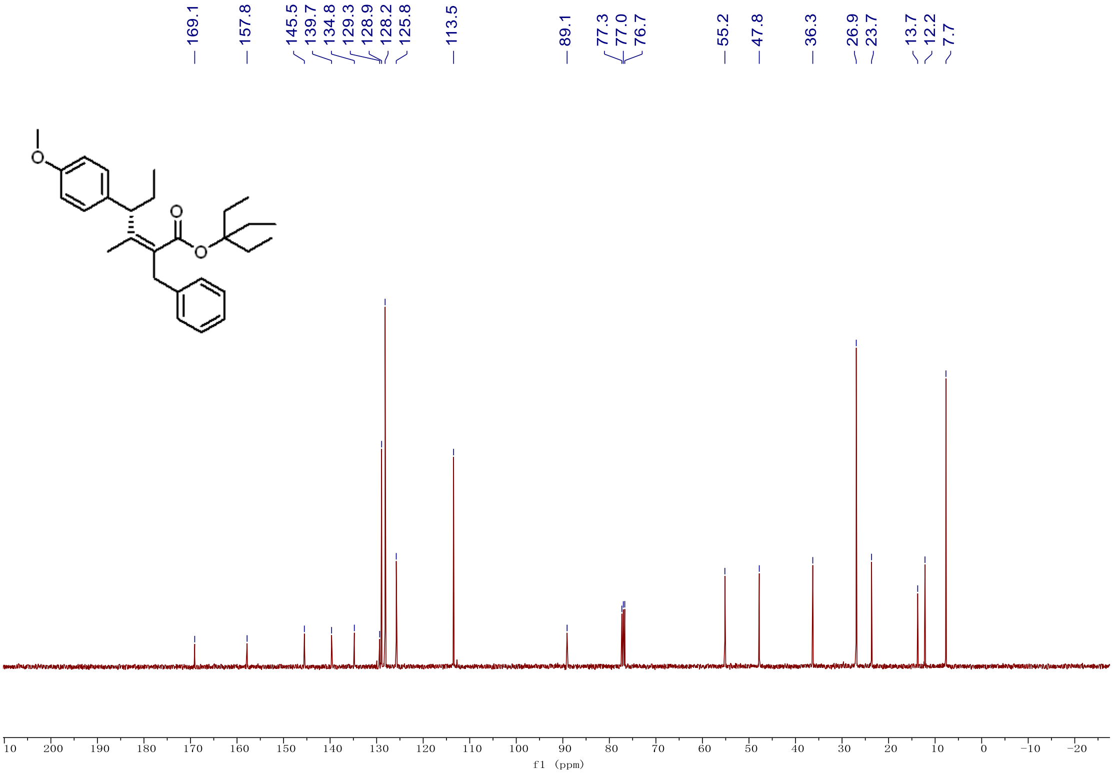

^{13}C NMR spectrum for compound **5m**

8. HPLC analysis.

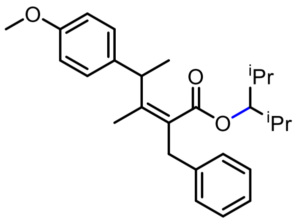

HPLC using two AD-H columns (hexane: i-PrOH=98.5:1.5, 0.5 mL/min)

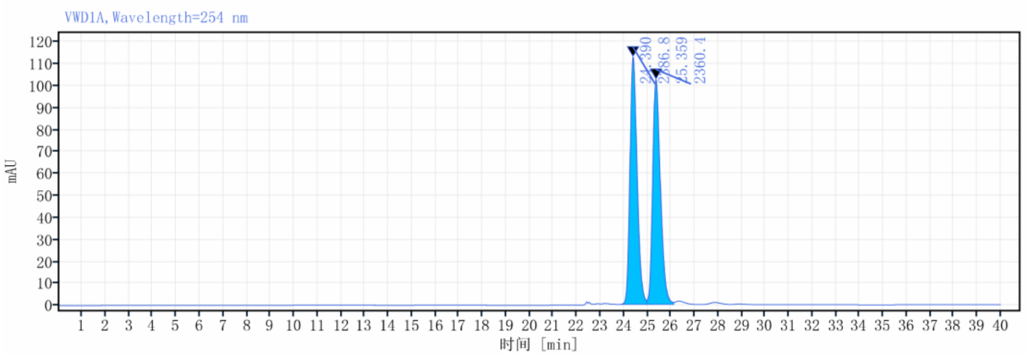

信号: VWD1A, Wavelength=254 nm

保留时间 [min]	类型	峰宽 [min]	峰面积	峰高	峰面积%	名称
24.390	BV	1.17	2386.75	112.69	50.28	
25.359	VV	1.15	2360.38	102.51	49.72	
		**总和**	**4747.13**			

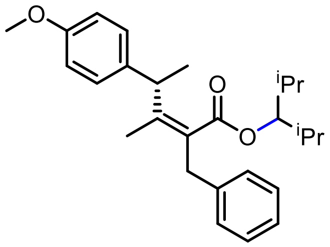

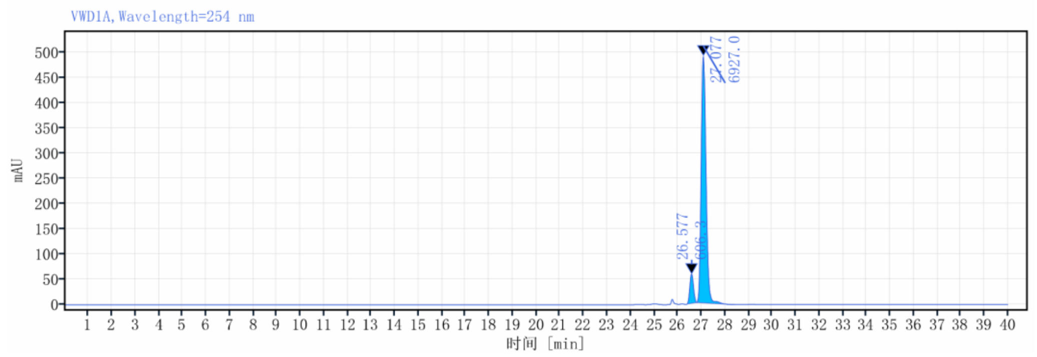

信号: VWD1A, Wavelength=254 nm

保留时间 [min]	类型	峰宽 [min]	峰面积	峰高	峰面积%	名称
26.577	MM m	0.40	606.27	57.45	8.05	
27.077	MM m	1.64	6927.01	488.16	91.95	
		**总和**	**7533.28**			

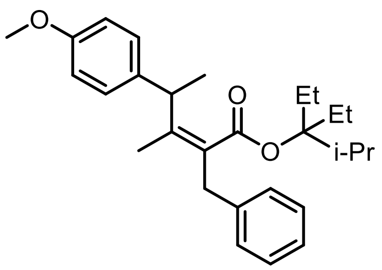

HPLC using two AD-H columns (hexane: i-PrOH=98.5:1.5, 0.5 mL/min)

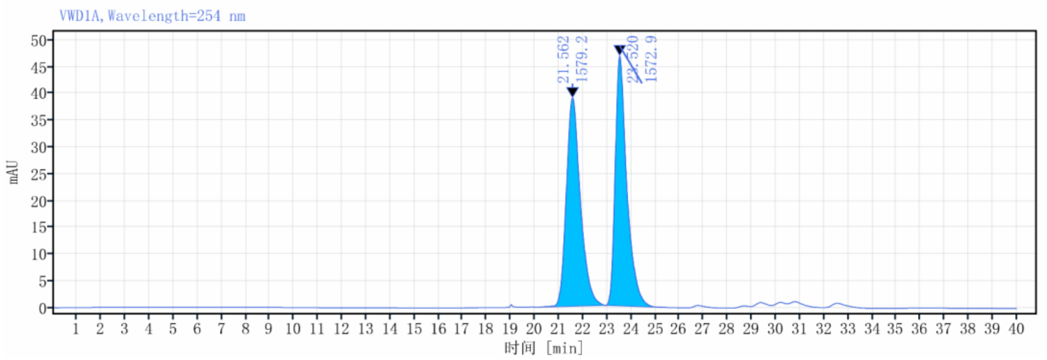

信号: VWD1A, Wavelength=254 nm

保留时间 [min]	类型	峰宽 [min]	峰面积	峰高	峰面积%	名称
21.562	BB	2.71	1579.22	38.93	50.10	
23.520	BB	2.70	1572.91	46.75	49.90	
		**总和**	**3152.14**			

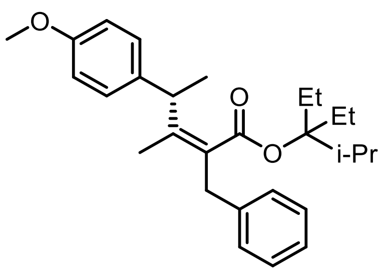

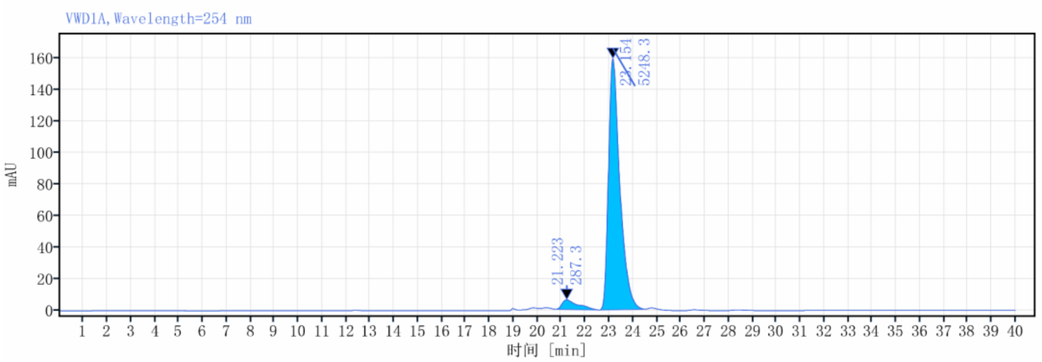

信号: VWD1A, Wavelength=254 nm

保留时间 [min]	类型	峰宽 [min]	峰面积	峰高	峰面积%	名称
21.223	BB	1.81	287.26	6.12	5.19	
23.154	BB	1.92	5248.28	159.26	94.81	
		总和	5535.54			

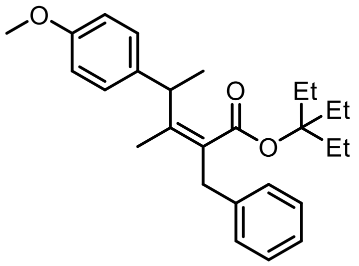

HPLC using two AD-H columns (hexane: i-PrOH=98.5:1.5, 0.5 mL/min)

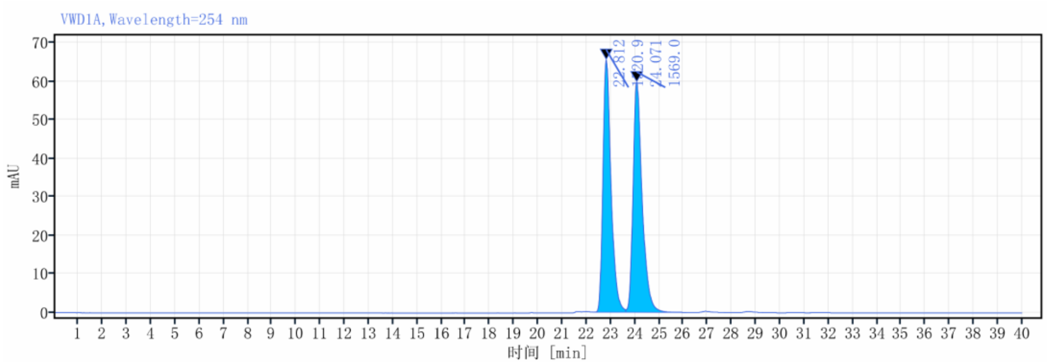

信号: VWD1A, Wavelength=254 nm

保留时间 [min]	类型	峰宽 [min]	峰面积	峰高	峰面积%	名称
22.812	BV	1.33	1520.94	65.48	49.22	
24.071	VB	2.49	1569.02	59.74	50.78	
		**总和**	**3089.96**			

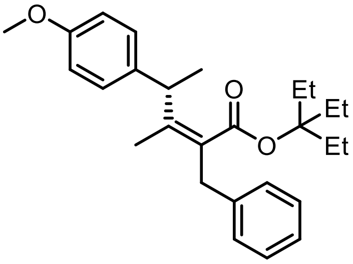

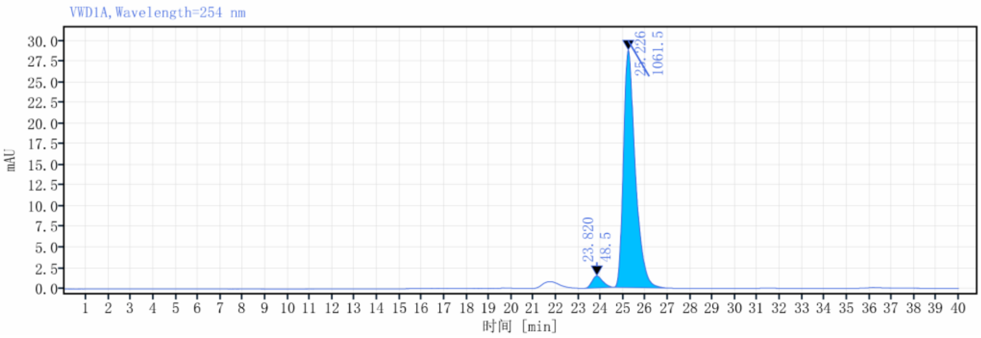

信号: VWD1A, Wavelength=254 nm

保留时间 [min]	类型	峰宽 [min]	峰面积	峰高	峰面积%	名称
23.820	MM m	1.28	48.49	1.44	4.37	
25.226	BB	2.61	1061.51	28.72	95.63	
		**总和**	**1110.00**			

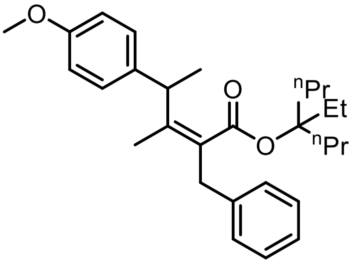

HPLC using two AD-H columns (hexane: i-PrOH=99:1, 0.5 mL/min)

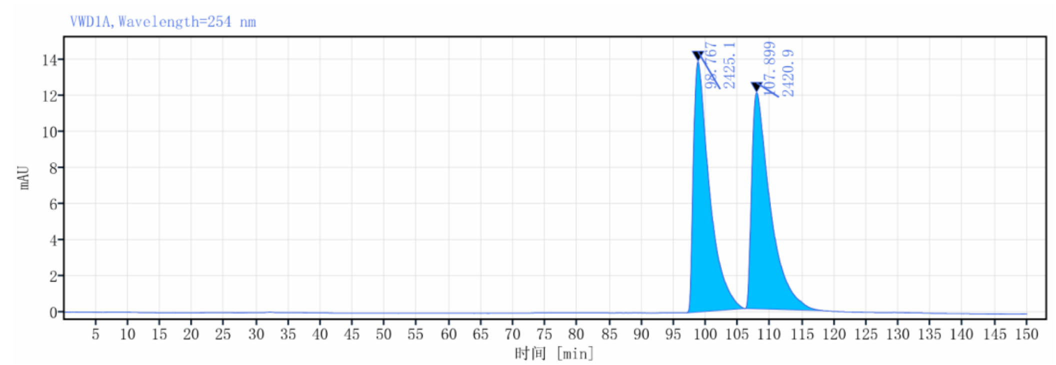

信号: VWD1A, Wavelength=254 nm

保留时间 [min]	类型	峰宽 [min]	峰面积	峰高	峰面积%	名称
98.767	BB	9.09	2425.07	13.86	50.04	
107.899	MM m	12.25	2420.94	11.97	49.96	
		**总和**	**4846.01**			

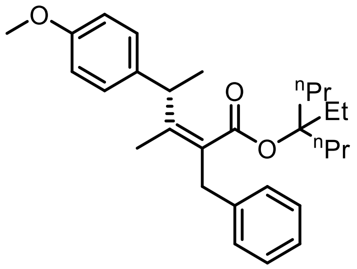

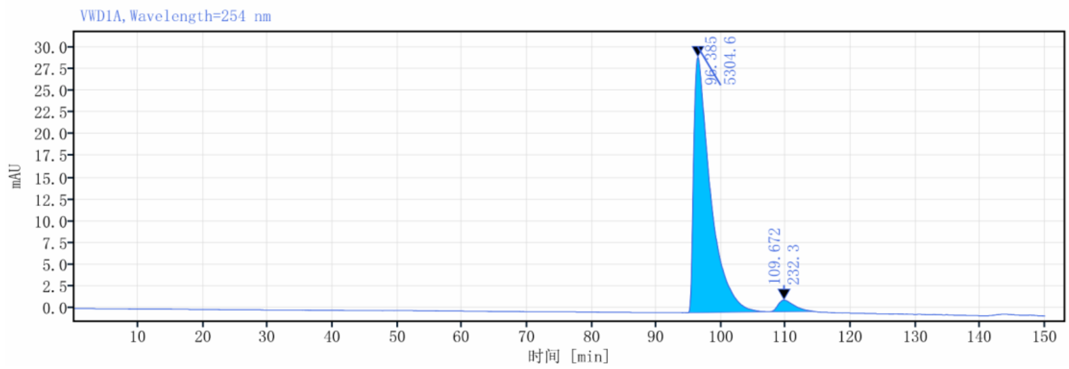

信号: VWD1A, Wavelength=254 nm

保留时间 [min]	类型	峰宽 [min]	峰面积	峰高	峰面积%	名称
96.385	BM m	12.31	5304.60	29.35	95.81	
109.672	MM m	7.21	232.27	1.33	4.19	
		**总和**	**5536.87**			

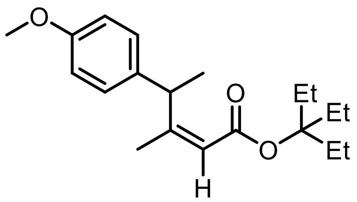

HPLC using two AD-H columns (hexane: i-PrOH=99:1, 0.5 mL/min)

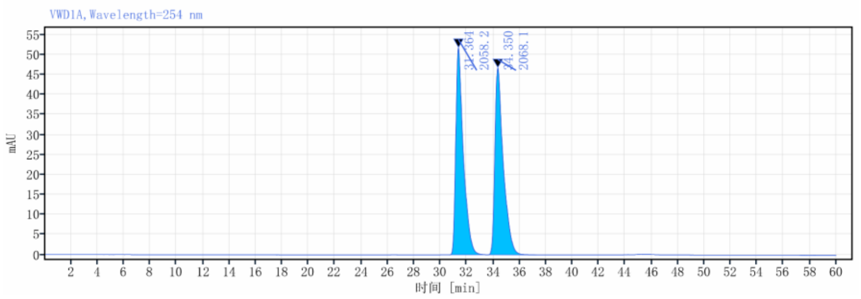

信号: VWD1A, Wavelength=254 nm

保留时间 [min]	类型	峰宽 [min]	峰面积	峰高	峰面积%	名称
31.364	BB	2.92	2058.18	51.81	49.88	
34.350	BB	3.70	2068.12	46.79	50.12	
		**总和**	**4126.29**			

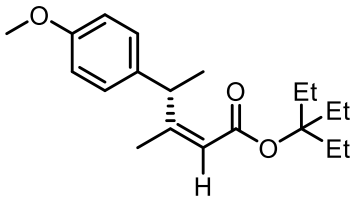

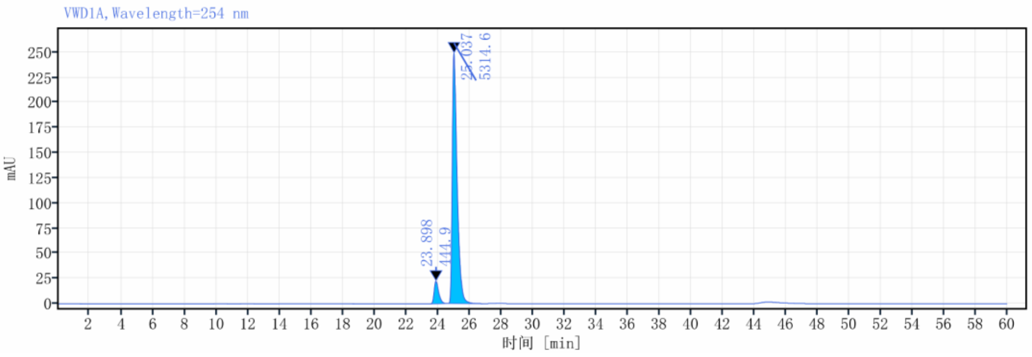

信号: VWD1A, Wavelength=254 nm

保留时间 [min]	类型	峰宽 [min]	峰面积	峰高	峰面积%	名称
23.898	MM m	1.11	444.86	22.48	7.72	
25.037	MM m	2.19	5314.57	249.06	92.28	
		**总和**	**5759.43**			

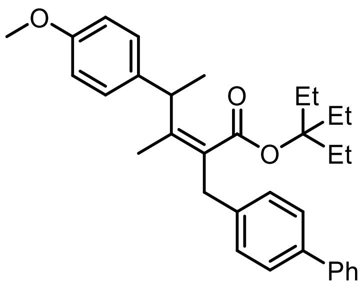

HPLC using two AD-H columns (hexane: i-PrOH=98.5:1.5, 0.5 mL/min)

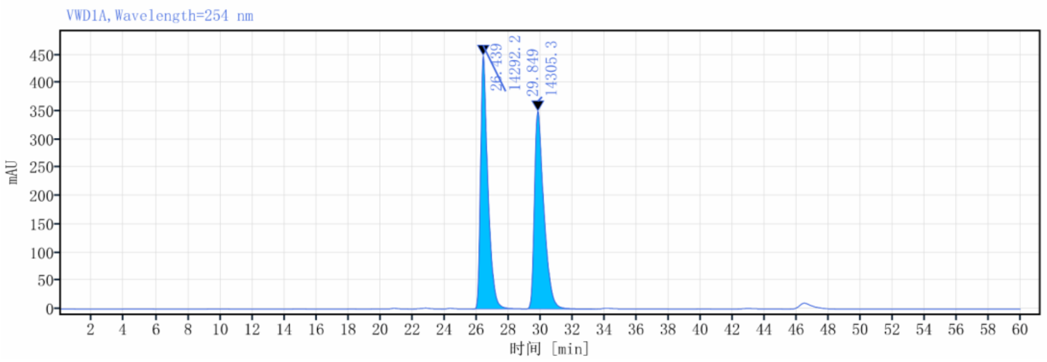

信号: VWD1A, Wavelength=254 nm

保留时间 [min]	类型	峰宽 [min]	峰面积	峰高	峰面积%	名称
26.439	BB	3.33	14292.21	448.14	49.98	
29.849	BB	4.34	14305.32	349.61	50.02	
		总和	28597.53			

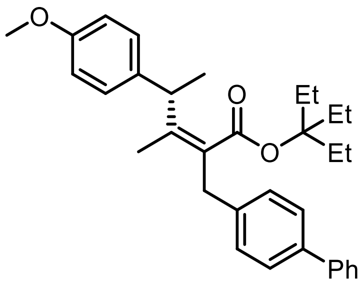

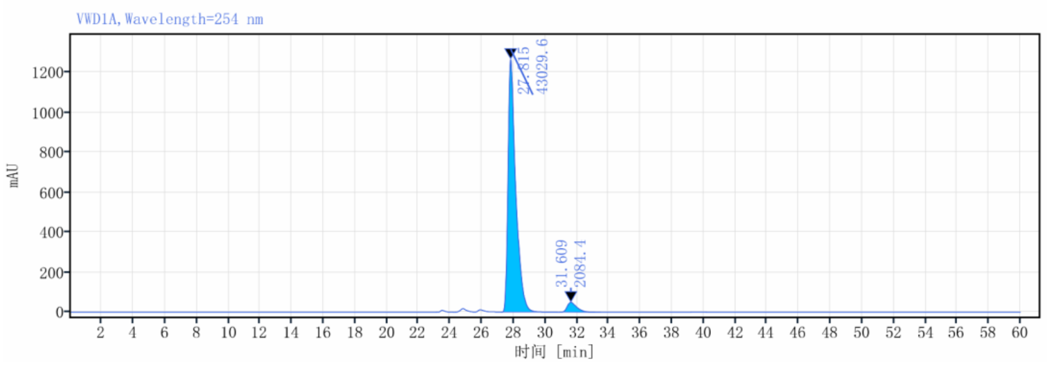

信号: VWD1A, Wavelength=254 nm

保留时间 [min]	类型	峰宽 [min]	峰面积	峰高	峰面积%	名称
27.815	MM m	3.52	43029.57	1267.63	95.38	
31.609	MM m	2.54	2084.39	49.75	4.62	
		**总和**	**45113.96**			

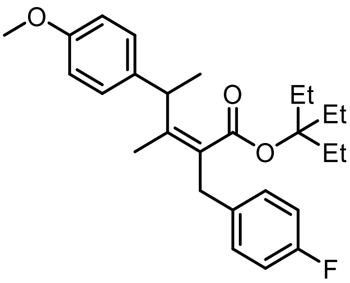

HPLC using two AD-H columns (hexane: i-PrOH=98.5:1.5, 0.5 mL/min)

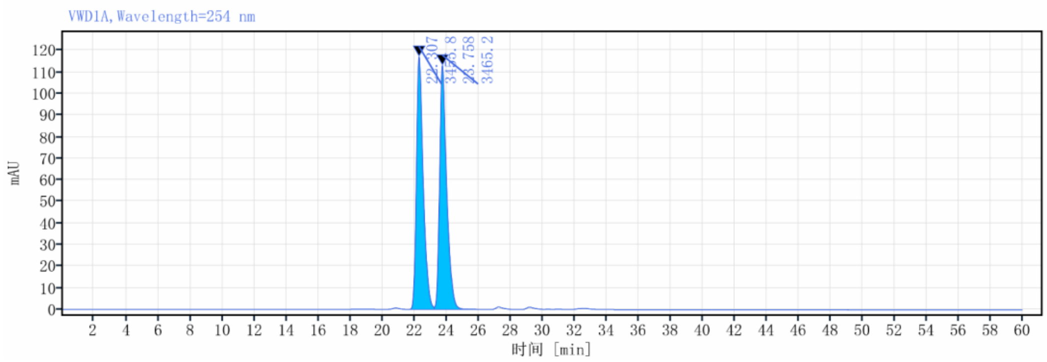

信号: VWD1A, Wavelength=254 nm

保留时间 [min]	类型	峰宽 [min]	峰面积	峰高	峰面积%	名称
22.307	BV	1.59	3455.80	117.25	49.93	
23.758	VB	2.78	3465.19	113.16	50.07	
		**总和**	**6921.00**			

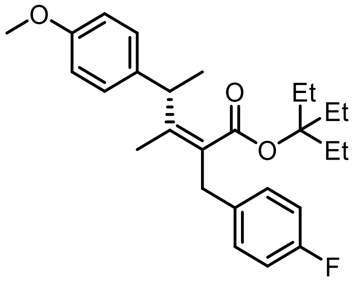

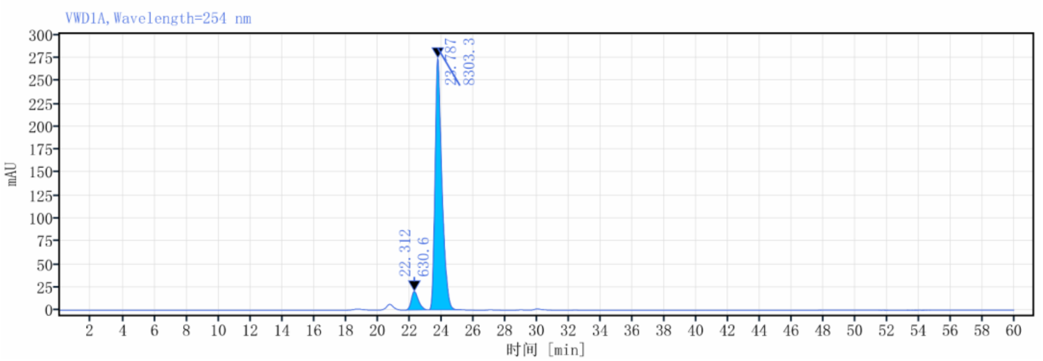

信号: VWD1A, Wavelength=254 nm

保留时间 [min]	类型	峰宽 [min]	峰面积	峰高	峰面积%	名称
22.312	MM m	1.34	630.56	20.22	7.06	
23.787	MM m	2.24	8303.26	274.24	92.94	
		**总和**	**8933.82**			

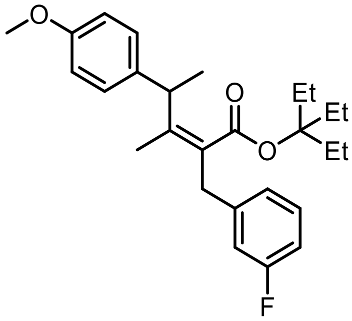

HPLC using two AD-H columns (hexane: i-PrOH=98.5:1.5, 0.5 mL/min)

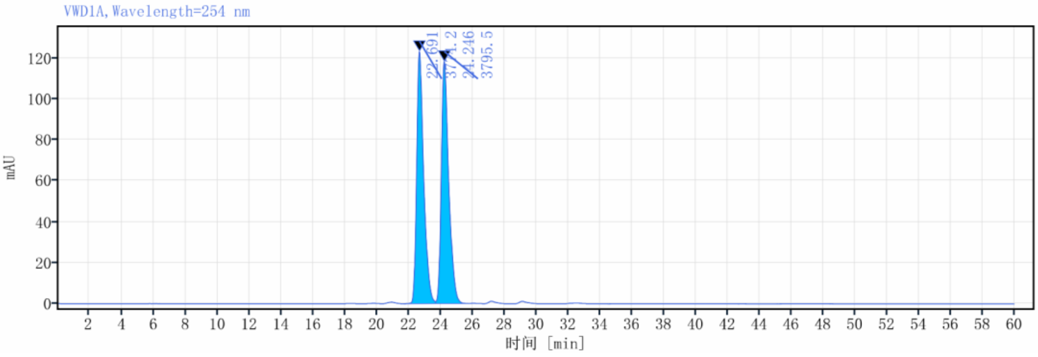

信号: VWD1A, Wavelength=254 nm

保留时间 [min]	类型	峰宽 [min]	峰面积	峰高	峰面积%	名称
22.691	BV	1.99	3774.25	123.37	49.86	
24.246	VB	2.03	3795.51	118.42	50.14	
		**总和**	**7569.76**			

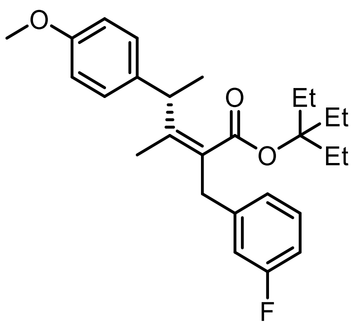

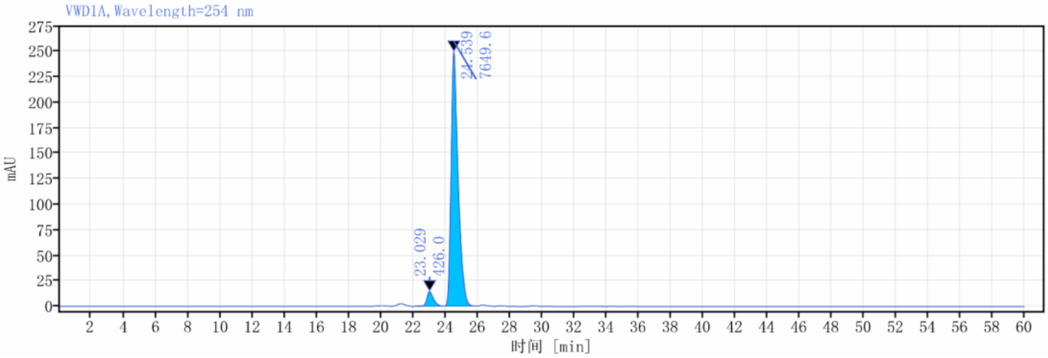

信号: VWD1A, Wavelength=254 nm

保留时间 [min]	类型	峰宽 [min]	峰面积	峰高	峰面积%	名称
23.029	MM m	1.87	426.03	14.64	5.28	
24.539	MB m	2.11	7649.61	250.02	94.72	
		**总和**	**8075.65**			

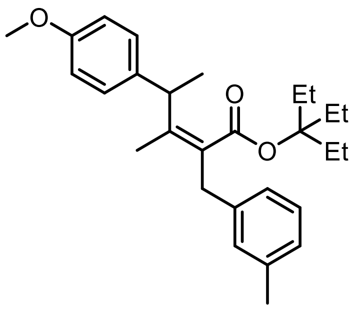

HPLC using two AD-H columns (hexane: i-PrOH=98.5:1.5, 0.5 mL/min)

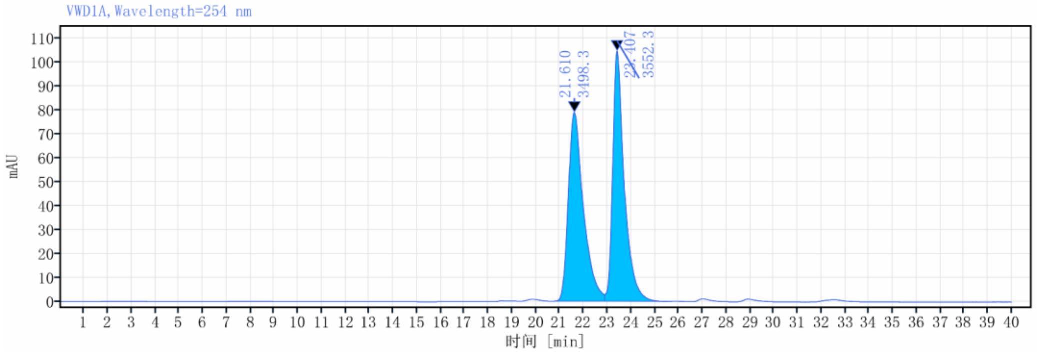

信号: VWD1A, Wavelength=254 nm

保留时间 [min]	类型	峰宽 [min]	峰面积	峰高	峰面积%	名称
21.610	BV	2.24	3498.29	79.13	49.62	
23.407	VB	2.63	3552.32	104.90	50.38	
		总和	7050.60			

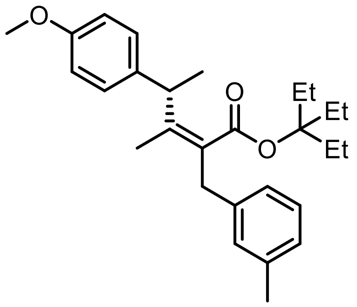

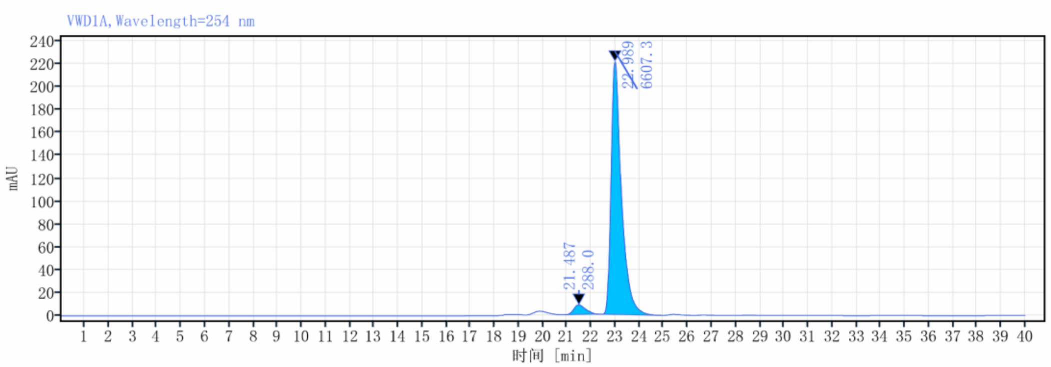

信号: VWD1A, Wavelength=254 nm

保留时间 [min]	类型	峰宽 [min]	峰面积	峰高	峰面积%	名称
21.487	BM m	1.34	287.97	8.53	4.18	
22.989	MB m	2.73	6607.34	221.18	95.82	
		**总和**	**6895.31**			

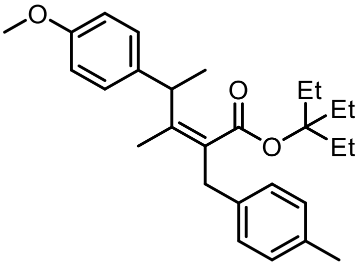

HPLC using two AD-H columns (hexane: i-PrOH=98.5:1.5, 0.5 mL/min)

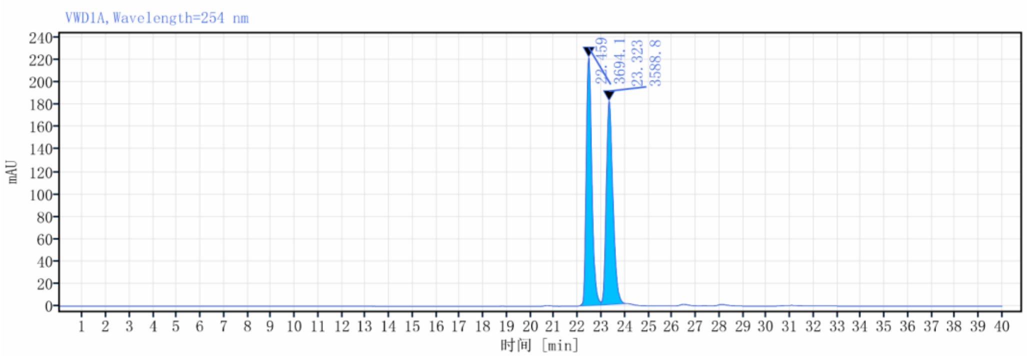

信号: VWD1A, Wavelength=254 nm

保留时间 [min]	类型	峰宽 [min]	峰面积	峰高	峰面积%	名称
22.459	BV	0.93	3694.14	221.98	50.72	
23.323	VB	1.07	3588.84	181.97	49.28	
		**总和**	**7282.97**			

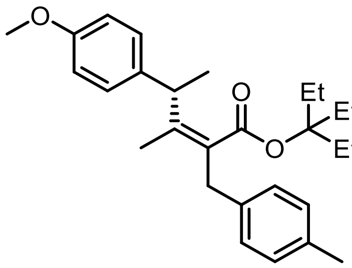

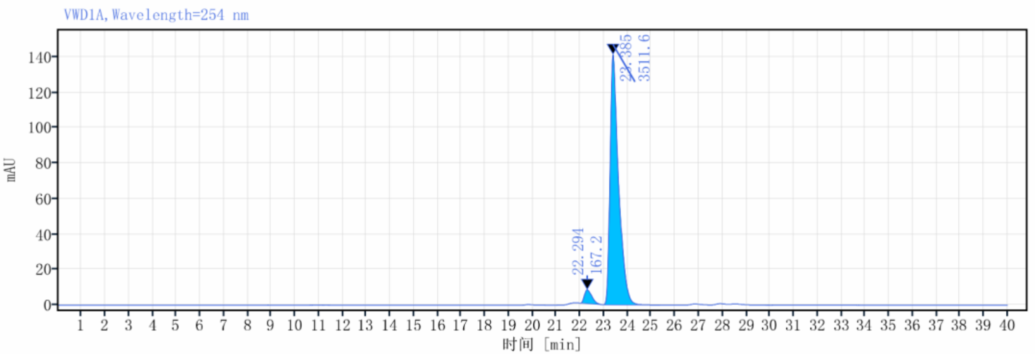

信号: VWD1A, Wavelength=254 nm

保留时间 [min]	类型	峰宽 [min]	峰面积	峰高	峰面积%	名称
22.294	MM m	0.84	167.21	7.76	4.55	
23.385	MB m	2.39	3511.64	141.21	95.45	
		**总和**	**3678.84**			

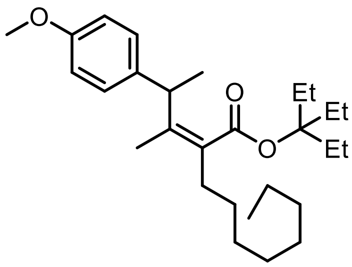

HPLC using two AD-H columns (hexane: i-PrOH=99:1, 0.5 mL/min)

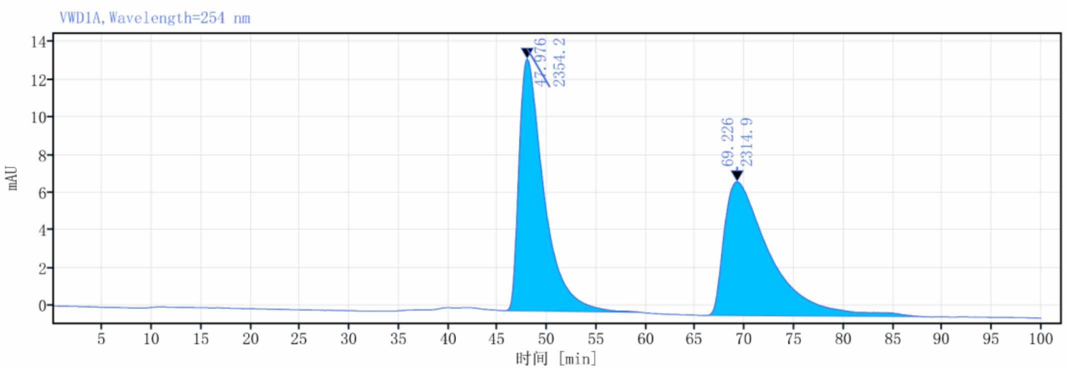

信号: VWD1A, Wavelength=254 nm

保留时间 [min]	类型	峰宽 [min]	峰面积	峰高	峰面积%	名称
47.976	MM m	14.08	2354.15	13.38	50.42	
69.226	MM m	22.42	2314.87	7.12	49.58	
		**总和**	**4669.02**			

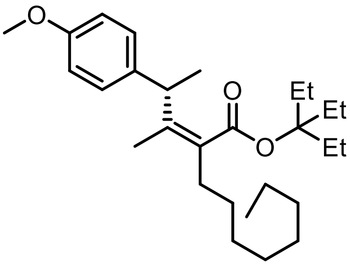

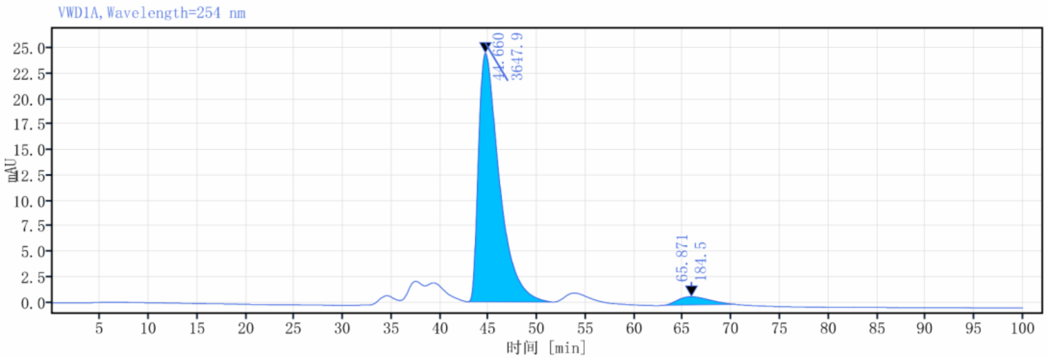

信号: VWD1A, Wavelength=254 nm

保留时间 [min]	类型	峰宽 [min]	峰面积	峰高	峰面积%	名称
44.660	BB	8.68	3647.85	24.44	95.18	
65.871	MM m	7.32	184.54	0.81	4.82	
		**总和**	**3832.39**			

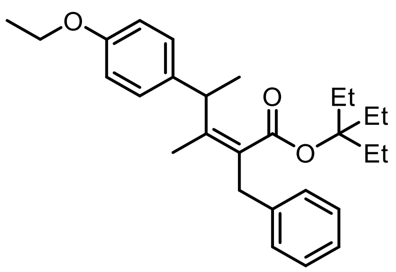

HPLC using two OD-H columns (hexane: i-PrOH=99:1, 0.5 mL/min)

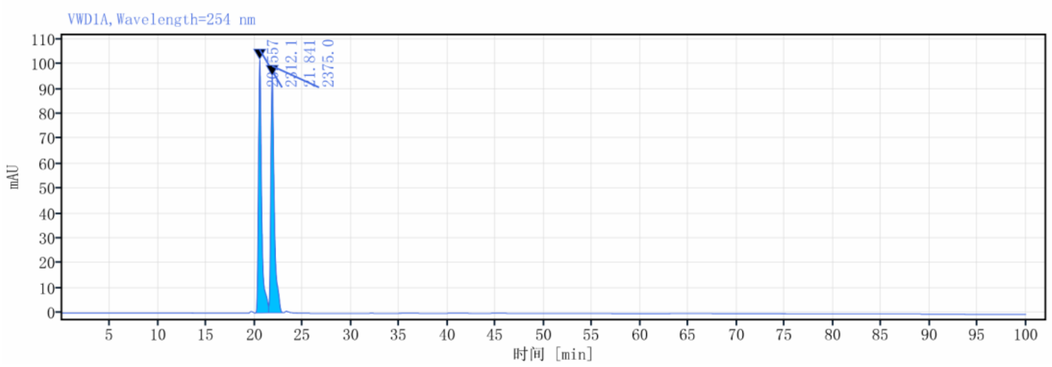

信号: VWD1A, Wavelength=254 nm

保留时间 [min]	类型	峰宽 [min]	峰面积	峰高	峰面积%	名称
20.557	BV	1.41	2312.13	101.71	49.33	
21.841	VB	1.54	2374.99	95.10	50.67	
		总和	4687.12			

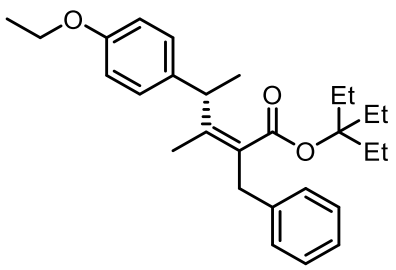

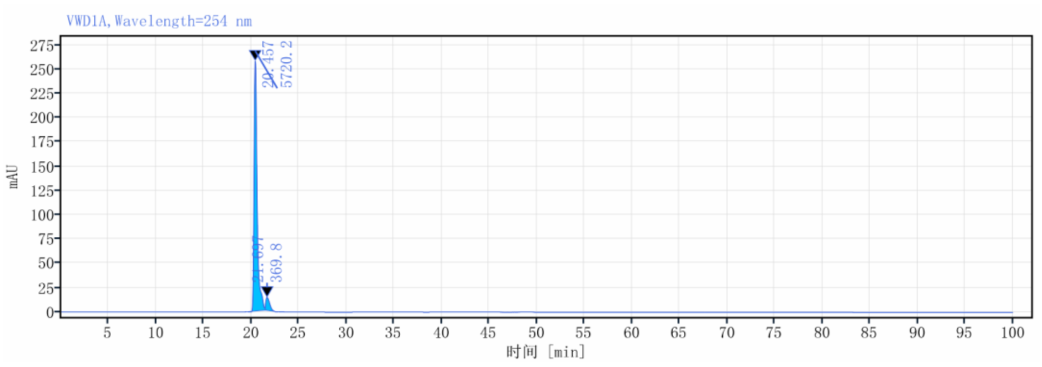

信号: VWD1A, Wavelength=254 nm

保留时间 [min]	类型	峰宽 [min]	峰面积	峰高	峰面积%	名称
20.457	MM m	1.79	5720.16	257.49	93.93	
21.697	MM m	0.96	369.78	13.49	6.07	
		总和	6089.94			

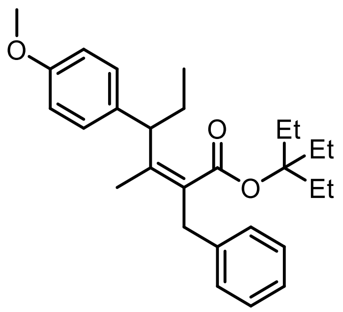

HPLC using two AD-H columns (hexane: i-PrOH=99:1, 0.5 mL/min)

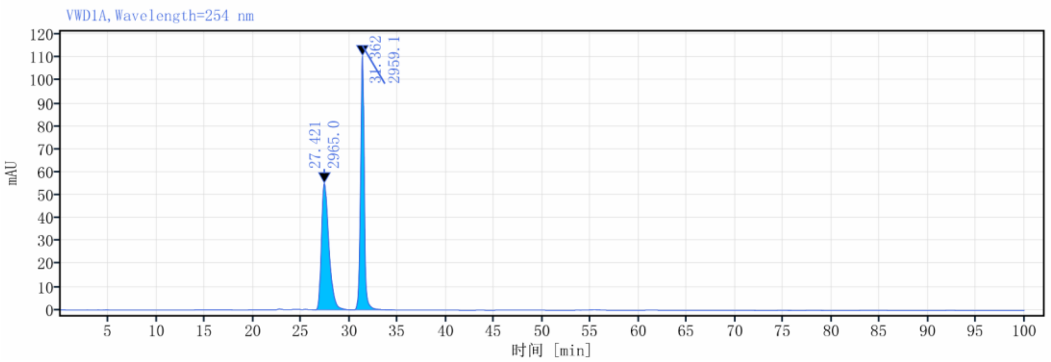

信号: VWD1A, Wavelength=254 nm

保留时间 [min]	类型	峰宽 [min]	峰面积	峰高	峰面积%	名称
27.421	BB	3.92	2964.98	55.12	50.05	
31.362	BB	4.02	2959.10	110.68	49.95	
		**总和**	**5924.08**			

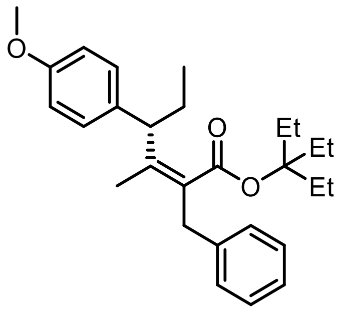

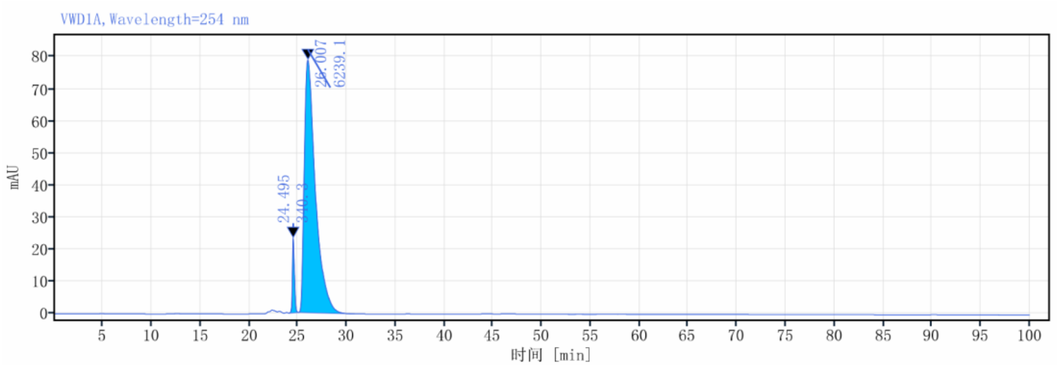

信号: VWD1A, Wavelength=254 nm

保留时间 [min]	类型	峰宽 [min]	峰面积	峰高	峰面积%	名称
24.495	BB	0.97	340.34	23.35	5.17	
26.007	BB	5.71	6239.13	78.76	94.83	
		**总和**	**6579.47**			

9. Definition of the absolute configuration

The absolute configuration of derivative **7**

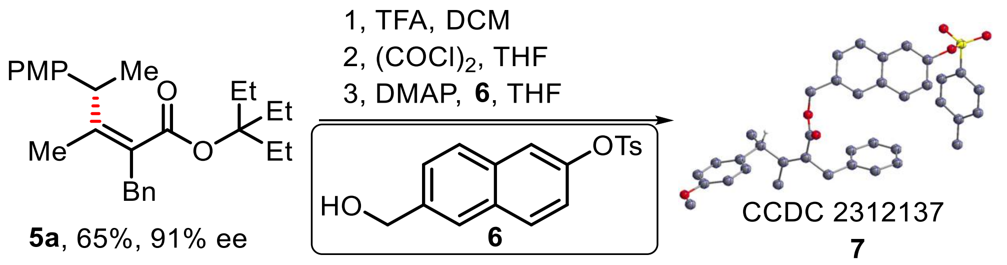

To a stirred solution of **5a** (245 mg, 0.6 mmol) in DCM (4 mL) was added TFA (0.8 mL) dropwise at 0 °C. After being stirred for 1 h, the reaction mixture was warmed up to room temperature. After 3 h, the reaction mixture was concentrated in vacuo. After the addition of hexane to the residue, the solvent was evaporated at 40 °C to remove the residual TFA. The same manipulation was conducted once again with hexane and twice with toluene;

Under Ar, to a stirred solution of the acid in THF, was added $(COCl)_2$ (51 μL, 0.6 mmol), and a drop of DMF at 0 ºC, after being stirred for 1 h, the solvent was removed in vacuo. To a stirred solution of DMAP (110 mg, 0.9 mmol), **6** in THF, the mixture prepared last step in THF was added dropwise at 0 ºC. After being stirred overnight, the reaction mixture was concentrated in vacuo and was purified by flash chromatography on silica gel to afford the corresponding product in 42% yield (156 mg).

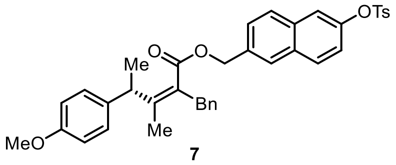

7

^{1}H NMR (400 MHz, Chloroform-*d*) δ 7.65 – 7.62 (m, 2H), 7.56 – 7.50 (m, 3H), 7.36 (d, *J* = 2.4 Hz, 1H), 7.21 – 6.99 (m, 11H), 6.73 (d, *J* = 8.7 Hz, 2H), 5.17 (s, 2H), 4.50 (q, *J* = 7.0 Hz, 1H), 3.69 (s, 3H), 3.64 (d, *J* = 6.1 Hz, 2H), 2.35 (s, 3H), 1.47 (s, 3H), 1.34 (d, *J* = 7.1 Hz, 3H).

^{13}C NMR (101 MHz, Chloroform-*d*) δ 169.3, 157.9, 149.9, 147.4, 145.4, 139.1, 135.1, 134.2, 132.9, 132.4, 131.6, 129.7(3C), 128.5, 128.4, 128.4, 128.1, 128.1, 126.3, 126.6, 126.5, 126.0, 121.4, 119.7, 113.5, 65.9, 55.2, 40.9, 35.8, 21.7, 17.1, 14.5.

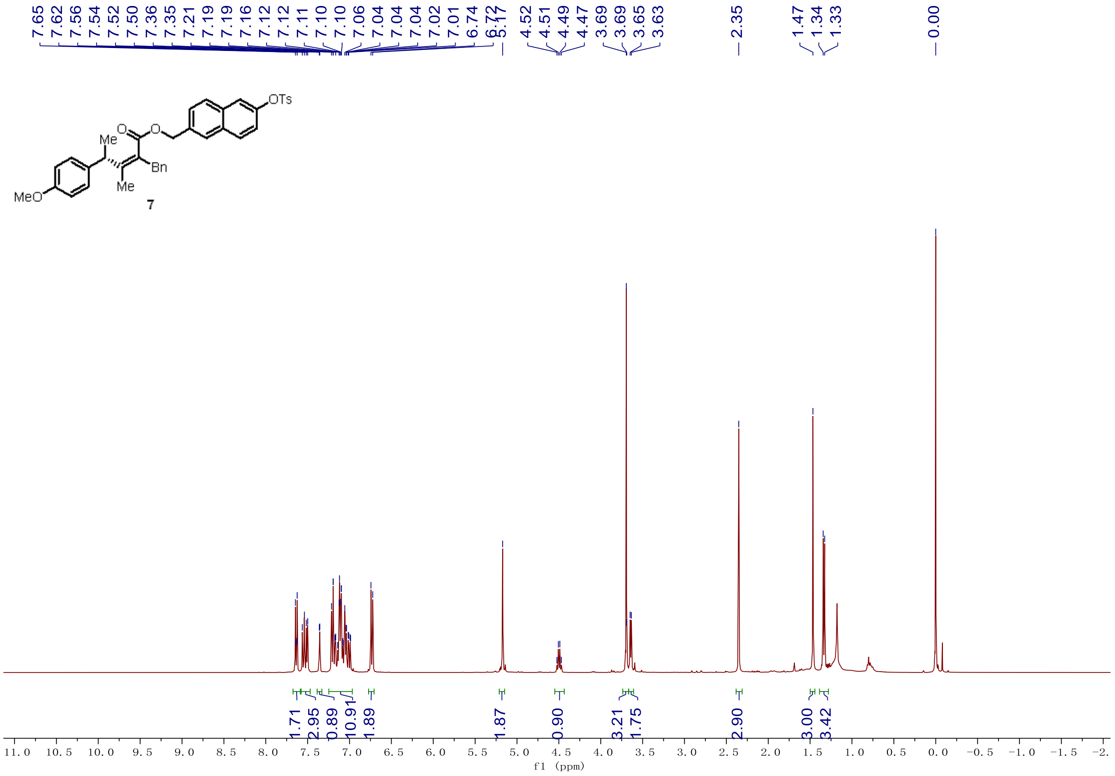

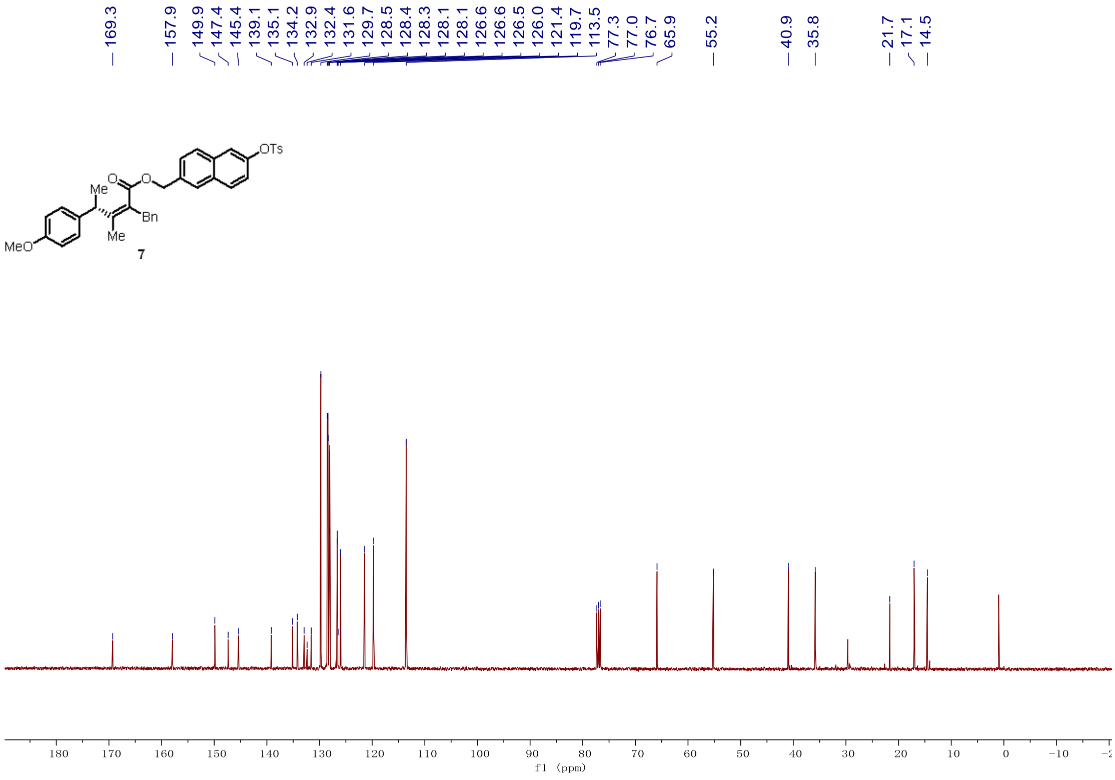

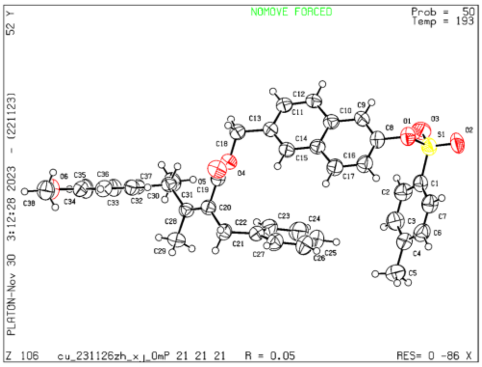

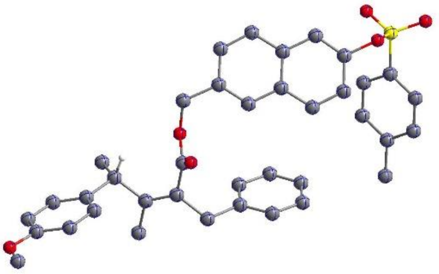

CCDC 2312137

Identification code	cu_231126ZH_XJ_0m
Empirical formula	$C_{38}H_{36}O_6S$
Formula weight	620.73
Temperature/K	193.00
Crystal system	orthorhombic
Space group	$P2_12_12_1$
a/Å	6.1870(17)
b/Å	8.034(2)
c/Å	65.131(17)
α/°	90
β/°	90
γ/°	90
Volume/Å^3	3237.5(15)
Z	4
ρ_{calc}g/cm^3	1.274
μ/mm^{-1}	1.264
F(000)	1312.0
Crystal size/mm^3	0.13 × 0.12 × 0.1
Radiation	CuKα (λ = 1.54178)
2Θ range for data collection/°	5.428 to 136.298
Index ranges	-6 ≤ h ≤ 6, -9 ≤ k ≤ 8, -76 ≤ l ≤ 78
Reflections collected	39975
Independent reflections	5730 [R_{int} = 0.0991, R_{sigma} = 0.0518]
Data/restraints/parameters	5730/0/411
Goodness-of-fit on F^2	1.053
Final R indexes [I>=2σ (I)]	R_1 = 0.0516, wR_2 = 0.1224
Final R indexes [all data]	R_1 = 0.0736, wR_2 = 0.1354
Largest diff. peak/hole / e Å^{-3}	0.24/-0.21
Flack parameter	0.043(13)

Table 2 Fractional Atomic Coordinates (×10^4) and Equivalent Isotropic Displacement Parameters (Å^2×10^3) for cu_231126ZH_XJ_0m. U_{eq} is defined as 1/3 of the trace of the orthogonalised U_{IJ} tensor.

atom	x	y	z	U(eq)
S(1)	1236(2)	6674.4(15)	5348.0(2)	60.3(4)
O(1)	2605(6)	7648(4)	5520.0(4)	61.1(9)
C(1)	2660(8)	4817(6)	5315.7(6)	53.7(11)
O(2)	1461(6)	7731(4)	5172.7(5)	76.5(11)
C(2)	1885(8)	3349(6)	5401.6(7)	63.6(13)
O(3)	-832(5)	6326(4)	5429.1(5)	73.1(10)
C(3)	3062(9)	1894(6)	5383.2(8)	69.3(14)
O(4)	4043(5)	2411(3)	6636.5(4)	51.0(7)
C(4)	5024(9)	1893(6)	5281.2(7)	61.7(13)
O(5)	7271(6)	3520(4)	6712.0(5)	64.4(9)
C(5)	6304(10)	298(7)	5264.0(8)	84.6(17)
O(6)	8801(6)	-988(4)	7753.0(5)	73.2(10)
C(6)	5767(9)	3354(7)	5194.2(7)	67.6(13)
C(7)	4613(8)	4817(6)	5211.1(7)	60.9(12)
C(8)	2912(8)	6870(6)	5713.7(6)	53.1(11)
C(9)	1385(8)	6997(5)	5861.1(7)	53.9(11)
C(10)	1790(7)	6278(5)	6057.7(6)	50.8(11)
C(11)	269(8)	6358(6)	6218.0(7)	55.5(11)
C(12)	687(8)	5604(6)	6403.0(7)	55.9(12)
C(13)	2664(8)	4794(5)	6440.9(6)	48.7(10)
C(14)	4170(8)	4711(5)	6288.4(6)	52.7(11)
C(15)	3772(7)	5436(5)	6093.2(6)	50.5(10)
C(16)	5324(8)	5379(6)	5933.8(7)	60.2(12)
C(17)	4899(8)	6079(6)	5745.1(7)	60.6(13)

C(18)	3007(8)	4016(6)	6651.6(7)	55.1(11)
C(19)	6172(8)	2301(5)	6676.7(6)	48.9(10)
C(20)	6993(7)	563(5)	6667.0(6)	48.1(10)
C(21)	8749(8)	328(6)	6509.4(6)	54.9(11)
C(22)	8111(8)	634(5)	6288.3(6)	52.6(11)
C(23)	6130(9)	129(7)	6212.5(7)	68.9(13)
C(24)	5654(12)	355(8)	6005.3(9)	91(2)
C(25)	7120(15)	1076(9)	5876.9(9)	99(2)
C(26)	9077(13)	1593(9)	5949.9(9)	94(2)
C(27)	9577(9)	1375(7)	6154.8(7)	69.3(14)
C(28)	6276(7)	-589(5)	6799.9(6)	47.4(10)
C(29)	7054(8)	-2374(5)	6792.1(8)	58.3(12)
C(30)	4708(7)	-204(6)	6972.5(6)	51.8(11)
C(31)	2612(8)	-1181(7)	6946.0(8)	70.7(14)
C(32)	5834(7)	-473(5)	7180.2(6)	47.5(10)
C(33)	5077(8)	-1561(6)	7328.9(7)	56.9(11)
C(34)	6101(9)	-1710(6)	7517.5(7)	64.5(12)
C(35)	7927(8)	-772(6)	7559.6(6)	55.8(12)
C(36)	8739(8)	299(6)	7412.9(6)	55.6(11)
C(37)	7681(8)	429(5)	7226.2(6)	52.1(11)
C(38)	10712(9)	-62(7)	7800.2(8)	76.6(15)

10. DFT calculations

All density functional theory (DFT) calculations are carried out by Gaussian 09B program [2]. Geometry optimizations are conducted by B3LYP functional[3] and Grimme's dispersion correction with Becke-Johnson damping[4]. And all atoms are treated with the def2-SVP basis set[5]. The solvation effects of DMF are taken into consideration by the Integral Equation Formalism PCM (IEFPCM) [6]. Vibrational frequency analyses are conducted at the same level to ensure all stationary

points as local minima (zero imaginary frequencies). The single-point energy calculations are evaluated with M06-2X functional[7], and all atoms are treated with def2-TZVP basis set[5]. The solvent (DMF) effects are calculated with the Solvent Model Density (SMD) method[8]. All optimized geometric figures are plotted by CYLview [9].

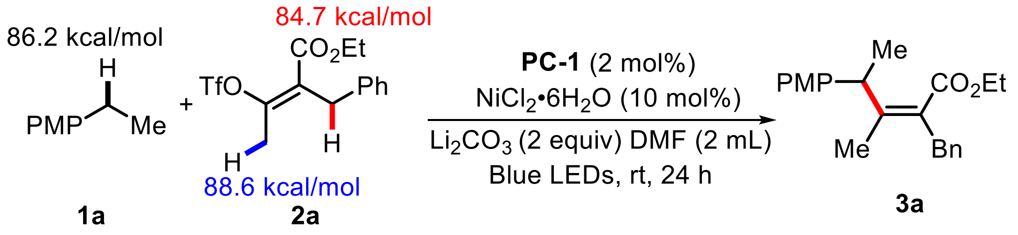

Upon completion of the geometry optimizations, BDE and BDFE calculations were performed for the target molecule (AB) and its dissociation products (A· and B·). The BDE was calculated using the following equation:

$$\mathrm{BDE}=E(\mathrm{A}\cdot)+E(\mathrm{B}\cdot)-E(\mathrm{AB})$$

where $E(\mathrm{A}\cdot)$,$E(\mathrm{B}\cdot)$,and $E(\mathrm{AB})$ represent the single-point energies of the A radical, B radical, and the AB molecule, respectively.

The BDFE was calculated using the following equation:

$$\mathrm{BDFE}=G(\mathrm{A}\cdot)+G(\mathrm{B}\cdot)-G(\mathrm{AB})$$

where $G(\mathrm{A}\cdot)$,$G(\mathrm{B}\cdot)$,and $G(\mathrm{AB})$ represent the Gibbs free energies of the A radical, B radical, and the AB molecule, respectively.

The calculated BDE and BDFE values for the bond dissociation process are summarized in Table 1.

Supplementary Table 1. The computational BDE and BDFE in **1a** and **2a**.

	Electronic energy (a.u.)		BDE (kcal/mol)	BDFE (kcal/mol)
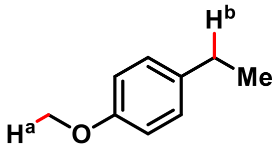 **1a**	-425.375065	C-H^a	98.29	88.20
		C-H^b	86.19	75.96
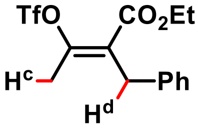 **2a**	-1616.368260	C-H^c	88.56	78.98
		C-H^d	84.73	75.06

Images of key structures

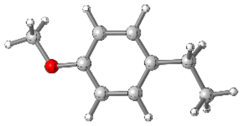

1a

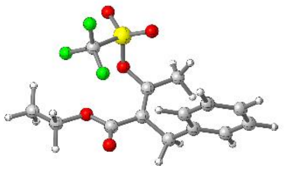

2a

Cartesian Coordinates of All the Optimized Geometries

1a

O	-2.713455000000	-1.484598000000	0.000203000000
C	-1.545989000000	-0.789280000000	0.000098000000
C	-0.364927000000	-1.546154000000	0.000132000000
C	0.879679000000	-0.916595000000	0.000036000000
C	0.992732000000	0.483289000000	-0.000098000000
C	-0.198904000000	1.223240000000	-0.000126000000
C	-1.455746000000	0.612153000000	-0.000032000000
C	2.325652000000	1.209036000000	-0.000223000000
C	-3.933139000000	-0.769833000000	0.000141000000
C	3.571265000000	0.327322000000	0.000009000000
H	-0.443764000000	-2.635468000000	0.000236000000
H	1.776123000000	-1.538735000000	0.000067000000
H	-0.148076000000	2.316244000000	-0.000224000000
H	-2.351182000000	1.233262000000	-0.000057000000
H	2.356424000000	1.880398000000	-0.876916000000
H	2.356371000000	1.880794000000	0.876167000000
H	-4.735888000000	-1.518509000000	0.000216000000
H	-4.034343000000	-0.134540000000	0.897376000000
H	-4.034351000000	-0.134711000000	-0.897213000000
H	4.481073000000	0.946787000000	-0.000066000000
H	3.610174000000	-0.321877000000	0.889418000000
H	3.610268000000	-0.322220000000	-0.889144000000

2a

C	0.267155000000	0.616814000000	1.325306000000
C	0.871603000000	-0.373359000000	0.635813000000
C	-0.562526000000	0.450324000000	2.556237000000
C	0.650773000000	-1.820642000000	1.038158000000
O	0.442665000000	1.951620000000	0.916108000000
S	-0.789314000000	2.861936000000	0.357399000000
O	-0.335542000000	4.232084000000	0.467473000000
O	-2.067696000000	2.397523000000	0.868994000000
C	-0.773341000000	2.417163000000	-1.472920000000
F	-0.807527000000	1.097263000000	-1.615243000000

F	-1.852490000000	2.951135000000	-2.020402000000
F	0.311155000000	2.904568000000	-2.051551000000
C	1.676473000000	-0.182432000000	-0.609217000000
O	1.815236000000	-1.066608000000	-1.432610000000
O	2.241993000000	1.017447000000	-0.724607000000
C	3.607492000000	2.663507000000	-1.772630000000
C	3.011816000000	1.280999000000	-1.916078000000
C	-0.752936000000	-2.309198000000	0.727147000000
C	-1.265778000000	-2.210597000000	-0.576996000000
C	-2.560773000000	-2.644400000000	-0.866658000000
C	-3.366299000000	-3.184557000000	0.142854000000
C	-2.864416000000	-3.288078000000	1.442470000000
C	-1.566743000000	-2.850722000000	1.730662000000
H	-0.464246000000	1.339326000000	3.194374000000
H	-0.251148000000	-0.434084000000	3.124870000000
H	-1.626823000000	0.332833000000	2.300213000000
H	1.379058000000	-2.437209000000	0.493866000000
H	0.855526000000	-1.950036000000	2.110817000000
H	4.184503000000	2.916145000000	-2.674821000000
H	2.815960000000	3.415711000000	-1.641316000000
H	4.282168000000	2.711172000000	-0.904369000000
H	3.782509000000	0.502084000000	-2.019341000000
H	2.342207000000	1.205468000000	-2.786738000000
H	-0.638085000000	-1.793081000000	-1.366705000000
H	-2.944463000000	-2.561099000000	-1.886597000000
H	-4.380516000000	-3.521768000000	-0.084006000000
H	-3.484680000000	-3.706807000000	2.238801000000
H	-1.182947000000	-2.930445000000	2.751243000000

1a-OMe-H[a]

O	-2.729915000000	-1.469860000000	0.111199000000
C	-1.545415000000	-0.781047000000	0.070297000000
C	-0.375776000000	-1.546859000000	0.050820000000
C	0.870075000000	-0.917479000000	0.021774000000
C	0.981561000000	0.481810000000	0.011289000000
C	-0.208600000000	1.227598000000	0.039870000000
C	-1.463608000000	0.618368000000	0.072091000000
C	2.313893000000	1.206236000000	-0.024904000000
C	-3.900596000000	-0.864395000000	-0.209274000000
C	3.560113000000	0.325734000000	-0.031791000000
H	-0.457676000000	-2.635573000000	0.054181000000
H	1.766181000000	-1.539251000000	0.003977000000
H	-0.153793000000	2.320088000000	0.045019000000
H	-2.367408000000	1.226621000000	0.118986000000

H	2.326681000000	1.862475000000	-0.913382000000
H	2.357619000000	1.893375000000	0.838755000000
H	-4.762070000000	-1.525330000000	-0.109428000000
H	-3.882596000000	-0.054631000000	-0.944971000000
H	4.468499000000	0.946634000000	-0.056040000000
H	3.614428000000	-0.309700000000	0.866651000000
H	3.586034000000	-0.336633000000	-0.911813000000

1a-H[b]

O	-2.727708000000	-1.473148000000	0.048947000000
C	-1.552690000000	-0.798653000000	0.004219000000
C	-0.380140000000	-1.582192000000	0.037003000000
C	0.869801000000	-0.989451000000	-0.003643000000
C	1.020762000000	0.430691000000	-0.079772000000
C	-0.181761000000	1.195118000000	-0.111674000000
C	-1.438796000000	0.603229000000	-0.071147000000
C	2.287290000000	1.062507000000	-0.122229000000
C	-3.939803000000	-0.743555000000	0.021710000000
C	3.591170000000	0.333309000000	-0.093128000000
H	-0.486593000000	-2.667721000000	0.095313000000
H	1.757124000000	-1.624640000000	0.023339000000
H	-0.109946000000	2.284553000000	-0.170016000000
H	-2.326320000000	1.235141000000	-0.098473000000
H	2.300267000000	2.154964000000	-0.180667000000
H	-4.750615000000	-1.481800000000	0.067029000000
H	-4.023776000000	-0.062364000000	0.886055000000
H	-4.039456000000	-0.155282000000	-0.906793000000
H	4.441654000000	1.028646000000	-0.132433000000
H	3.704664000000	-0.280788000000	0.820557000000
H	3.694965000000	-0.368255000000	-0.942824000000

2a-H[c]

C	0.109656000000	0.659184000000	1.258793000000
C	0.750414000000	-0.396137000000	0.573250000000
C	-0.574019000000	0.570820000000	2.442922000000
C	0.582739000000	-1.810374000000	1.066945000000
O	0.132221000000	1.948561000000	0.673281000000
S	-1.050716000000	2.270307000000	-0.409773000000
O	-2.188292000000	2.871276000000	0.264545000000
O	-1.211057000000	1.149760000000	-1.325403000000
C	-0.135680000000	3.651286000000	-1.316209000000
F	0.535333000000	3.129254000000	-2.330168000000
F	-1.053111000000	4.488867000000	-1.770713000000
F	0.690898000000	4.284376000000	-0.503575000000

C	1.499833000000	-0.236996000000	-0.695478000000
O	1.608572000000	-1.147377000000	-1.497395000000
O	2.079282000000	0.955718000000	-0.856087000000
C	3.707942000000	2.395436000000	-1.849777000000
C	2.880897000000	1.144139000000	-2.042389000000
C	-0.828127000000	-2.349096000000	0.887164000000
C	-1.494756000000	-2.199929000000	-0.339017000000
C	-2.788165000000	-2.698778000000	-0.508032000000
C	-3.434924000000	-3.354933000000	0.545422000000
C	-2.777629000000	-3.508120000000	1.769019000000
C	-1.482933000000	-3.005486000000	1.937561000000
H	-1.060816000000	1.455199000000	2.854347000000
H	-0.634685000000	-0.366706000000	2.991649000000
H	1.285756000000	-2.447278000000	0.512199000000
H	0.863301000000	-1.871226000000	2.130015000000
H	4.324133000000	2.571327000000	-2.744432000000
H	3.068141000000	3.275319000000	-1.692840000000
H	4.377996000000	2.290583000000	-0.982958000000
H	3.508008000000	0.252640000000	-2.188796000000
H	2.204006000000	1.221695000000	-2.907183000000
H	-0.996575000000	-1.683757000000	-1.161331000000
H	-3.295556000000	-2.572583000000	-1.467690000000
H	-4.447811000000	-3.742558000000	0.412878000000
H	-3.273929000000	-4.016983000000	2.599022000000
H	-0.975548000000	-3.125660000000	2.898739000000

2a-H[d]

C	-0.080835000000	0.635260000000	0.831335000000
C	0.612683000000	-0.330878000000	0.097070000000
C	-0.873041000000	0.444251000000	2.076263000000
C	0.332823000000	-1.710554000000	0.162197000000
O	0.036653000000	1.988395000000	0.471263000000
S	-1.159758000000	2.699427000000	-0.393114000000
O	-1.144733000000	4.103542000000	-0.039039000000
O	-2.364306000000	1.881788000000	-0.374223000000
C	-0.446735000000	2.567816000000	-2.128629000000
F	-0.252556000000	1.290625000000	-2.433541000000
F	-1.320876000000	3.097790000000	-2.968318000000
F	0.698670000000	3.226298000000	-2.197155000000
C	1.798917000000	0.003889000000	-0.773844000000
O	2.269697000000	-0.776175000000	-1.574651000000
O	2.306413000000	1.215417000000	-0.549197000000
C	3.826872000000	3.014165000000	-0.908462000000
C	3.419990000000	1.631148000000	-1.364770000000

C	-0.918992000000	-2.352984000000	0.488808000000
C	-2.173335000000	-1.713847000000	0.321993000000
C	-3.356896000000	-2.388165000000	0.606214000000
C	-3.327967000000	-3.712977000000	1.062973000000
C	-2.099093000000	-4.368994000000	1.210207000000
C	-0.911917000000	-3.704205000000	0.914638000000
H	-0.513770000000	1.173909000000	2.821779000000
H	-0.757677000000	-0.567333000000	2.479016000000
H	-1.945782000000	0.643047000000	1.920772000000
H	1.145537000000	-2.372810000000	-0.145322000000
H	4.670689000000	3.373659000000	-1.516359000000
H	2.991574000000	3.721325000000	-1.017463000000
H	4.139787000000	3.002408000000	0.146703000000
H	4.233926000000	0.898528000000	-1.253211000000
H	3.103415000000	1.618769000000	-2.419475000000
H	-2.212026000000	-0.695391000000	-0.066305000000
H	-4.314099000000	-1.881906000000	0.461059000000
H	-4.260044000000	-4.235970000000	1.288591000000
H	-2.070160000000	-5.406061000000	1.552416000000
H	0.045281000000	-4.220031000000	1.023303000000

H_2(anchor)

H	-0.371371000000	-0.006190000000	0.000000000000
H	0.371371000000	0.006190000000	0.000000000000

H • (radical anchor)

H	0.0000000000000	0.0000000000000	0.000000000000

11. Reference

[1] D. Babinski, O. Soltani, D. E. Frantz, *Org. Lett.* **2008**, *10*, 2901-2904.

[2] Gaussian 09, Revision B.01, M. J. Frisch, G. W. Trucks, H. B. Schlegel, G. E. Scuseria, M. A. Robb, J. R. Cheeseman, G. Scalmani, V. Barone, B. Mennucci, G. A. Petersson, H. Nakatsuji, M. Caricato, X. Li, H. P. Hratchian, A. F. Izmaylov, J. Bloino, G. Zheng, J. L. Sonnenberg, M. Hada, M. Ehara, K. Toyota, R. Fukuda, J. Hasegawa, M. Ishida, T. Nakajima, Y. Honda, O. Kitao, H. Nakai, T. Vreven, J. A. Montgomery, Jr., J. E. Peralta, F. Ogliaro, M. Bearpark, J. J. Heyd, E. Brothers, K. N. Kudin, V. N. Staroverov, T. Keith, R. Kobayashi, J. Normand, K. Raghavachari, A. Rendell, J. C. Burant, S. S. Iyengar, J. Tomasi, M. Cossi, N. Rega, J. M. Millam, M. Klene, J. E. Knox, J. B. Cross, V. Bakken, C. Adamo, J. Jaramillo, R. Gomperts, R. E. Stratmann, O. Yazyev, A. J. Austin, R. Cammi, C. Pomelli, J. W. Ochterski, R. L. Martin, K. Morokuma, V. G. Zakrzewski, G. A. Voth, P. Salvador, J. J. Dannenberg, S. Dapprich, A. D. Daniels, O. Farkas, J. B. Foresman, J. V. Ortiz, J. Cioslowski, and D. J. Fox, Gaussian, Inc.,

Wallingford CT, 2013.

[3] a) A. D. Becke, *J. Chem. Phys.* **1993**, *98*, 5648-5652; b) W. Y. C. Lee, R. G. Parr, *Phys. Rev. B: Condens. Matter.* **1988**, *37*, 785-789.

[4] S. Grimme, S. Ehrlich, L. Goerigk, *J. Comput. Chem.* **2011**, *32*, 1456-1465.

[5] F. Weigend, R. Ahlrichs, *Phys. Chem. Chem. Phys.* **2005**, *7*, 3297-3305.

[6] a) E. S. S. Miertus, *Chem. Phys.* **1981**, *55*, 13; b) J. T. S. Miertus, *Chem. Phys.* **1982**, *65*, 7; c)E. S. J. L. Pascual-Ahuir, I. Tunon, *J. Comput. Chem.* **1994**, *15*, 12.

[7] Zhao, Y., Truhlar, D.G. *Theor. Chem. Acc.* 120, 215–241 (2008).

[8] M. Aleksandr V, C. Christopher J, T. Donald G, *J. Phys. Chem. B*. 2009, 113, 6378-6396.

[9] CYLview 1.0b; Legault, C. Y. Université de Sherbrooke, (http://www.cylview.org). (2009).
